# Discovery of numerous novel small genes in the intergenic regions of the *Escherichia coli* O157:H7 Sakai genome

**DOI:** 10.1371/journal.pone.0184119

**Published:** 2017-09-13

**Authors:** Sarah M. Hücker, Zachary Ardern, Tatyana Goldberg, Andrea Schafferhans, Michael Bernhofer, Gisle Vestergaard, Chase W. Nelson, Michael Schloter, Burkhard Rost, Siegfried Scherer, Klaus Neuhaus

**Affiliations:** 1 Chair for Microbial Ecology, Technische Universität München, Freising, Germany; 2 ZIEL - Institute for Food & Health, Technische Universität München, Freising, Germany; 3 Department of Informatics—Bioinformatics & TUM-IAS, Technische Universität München, Garching, Germany; 4 Research Unit Environmental Genomics, Helmholtz Zentrum München, Neuherberg, Germany; 5 Sackler Institute for Comparative Genomics, American Museum of Natural History New York, New York, United States of America; 6 Core Facility Microbiome/NGS, ZIEL - Institute for Food & Health, Technische Universität München, Freising, Germany; Centre for Research and Technology-Hellas, GREECE

## Abstract

In the past, short protein-coding genes were often disregarded by genome annotation pipelines. Transcriptome sequencing (RNAseq) signals outside of annotated genes have usually been interpreted to indicate either ncRNA or pervasive transcription. Therefore, in addition to the transcriptome, the translatome (RIBOseq) of the enteric pathogen *Escherichia coli* O157:H7 strain Sakai was determined at two optimal growth conditions and a severe stress condition combining low temperature and high osmotic pressure. All intergenic open reading frames potentially encoding a protein of ≥ 30 amino acids were investigated with regard to coverage by transcription and translation signals and their translatability expressed by the ribosomal coverage value. This led to discovery of 465 unique, putative novel genes not yet annotated in this *E*. *coli* strain, which are evenly distributed over both DNA strands of the genome. For 255 of the novel genes, annotated homologs in other bacteria were found, and a machine-learning algorithm, trained on small protein-coding *E*. *coli* genes, predicted that 89% of these translated open reading frames represent *bona fide* genes. The remaining 210 putative novel genes without annotated homologs were compared to the 255 novel genes with homologs and to 250 short annotated genes of this *E*. *coli* strain. All three groups turned out to be similar with respect to their translatability distribution, fractions of differentially regulated genes, secondary structure composition, and the distribution of evolutionary constraint, suggesting that both novel groups represent legitimate genes. However, the machine-learning algorithm only recognized a small fraction of the 210 genes without annotated homologs. It is possible that these genes represent a novel group of genes, which have unusual features dissimilar to the genes of the machine-learning algorithm training set.

## Introduction

The pathogenic *E*. *coli* strain O157:H7 Sakai (EHEC) was first isolated in 1996 from an outbreak in Japan [1]. When contaminated food is consumed, EHEC can cause bloody diarrhea and the disease may progress to the life-threatening hemolytic uremic syndrome [2]. In addition to humans [3] and contaminated food, EHEC persists in many environments, such as soil [4], plants [5], invertebrates [6], and cattle [7]. These environments represent various challenges requiring expression of a different set of bacterial genes [8]. Since there is no vaccination or targeted therapy available [9], it is important to better understand the biology of this enteric pathogen in order to prevent infections.

In contrast to eukaryotic genomes, bacterial genomes are densely covered with annotated protein-coding genes, e.g., 88.1% of the EHEC Sakai genome consists of protein-coding genes according to the most recent genome annotation [1]. Nevertheless, it is still possible that intergenic regions harbor overlooked short genes [10, 11]. After sequencing a bacterial genome, bioinformatics tools, such as GLIMMER [12] or RAST [13] are used for gene prediction and annotation. Especially for short genes, these tools are biased in that open reading frames (ORFs) shorter than 150 bp are often rejected [14] and in some cases are not even permitted for database entry [15]. Thus, the sensitivity of automated annotation processes in predicting short genes is quite low [16]. Additionally, the experimental detection of small proteins in proteome studies is difficult: Many small proteins are lost during proteome purification and many more are not detectable by classic mass spectrometry, because they do not produce enough tryptic peptides of the proper size [17]. Therefore, small proteins have been largely ignored in the past and our knowledge of their structures and functions is very limited [15]. Although small proteins have recently come more into focus [18, 19], the majority of them still belong to the ‘dark proteome’ lacking known folds or domains, thus rendering putative functional assignments using bioinformatics tools impossible [20, 21].

The rise of next-generation sequencing technologies allows high-throughput investigation of the expression status of genomes without any restriction to gene length. RNAseq strand-specifically determines the global transcriptome and widespread transcription outside of annotated genes has become increasingly obvious [22–25]. In the past, these transcription signals were generally interpreted as ncRNAs [26, 27] or just pervasive transcription without any biological significance [28–30]. However, ribosomal footprinting (RIBOseq) can be used to determine the coverage of RNA with ribosomes, indicating translation into a peptide of the associated RNA, thus, facilitating the global investigation of the translatome [31, 32]. Even more, RIBOseq reads usually show a triplet periodicity reflecting the codon-wise movement of the ribosome during the translation process [31, 33]. Combining ribosomal footprinting with RNAseq allows estimation of the translatability of an ORF, expressed by the ribosomal coverage value (RCV), which is the ratio of the reads per kilobase (of gene) per million sequenced reads (RPKM) value for the translatome over the RPKM value for the transcriptome. The RCV can be used to distinguish ncRNA from translated mRNA, and RIBOseq allows the discovery of many non-annotated short translated ORFs [33–39]. In bacteria, RIBOseq is less frequently applied. However, Baek et al. [40] recently reported 130 novel short genes in *Salmonella*, the smallest gene encoding a peptide of only 7 amino acids (AA). The translatome of EHEC strain EDL933 under a single growth condition yielded 72 novel genes encoded in intergenic regions, 95% of them encoding proteins smaller than 100 AA [11].

In this study, RIBOseq and RNAseq analysis of *E*. *coli* O157:H7 Sakai was compared at three different growth conditions to identify translated ORFs in the intergenic regions. The resulting candidates for novel genes were further characterized using bioinformatics analysis.

## Results

### Translatome signals of putative novel genes

**Fig 1:**
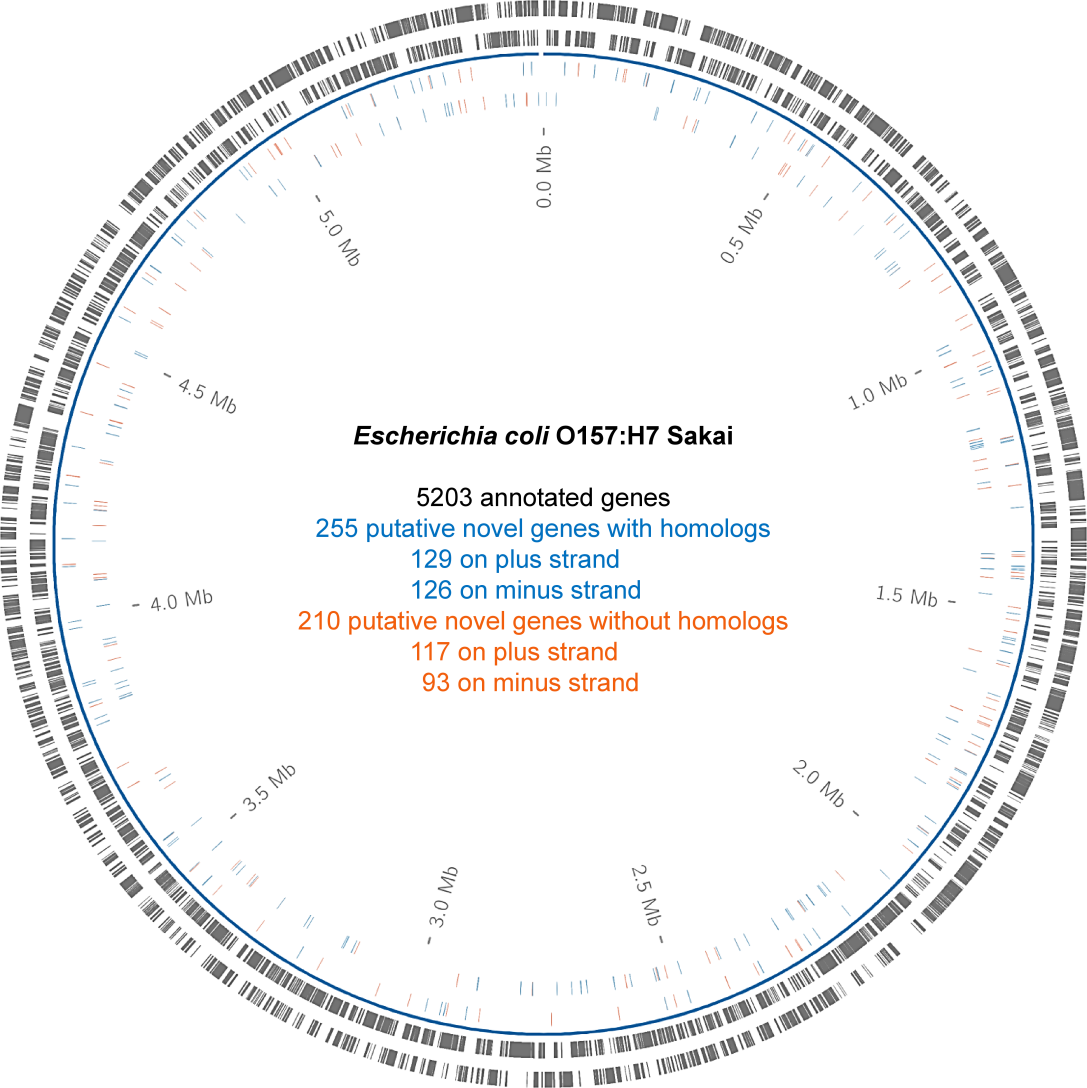
Distribution of 465 small novel genes within the EHEC genome. The circles from outside to inside show: annotated genes on the plus strand, annotated genes on the minus strand, novel genes on the plus strand and novel genes on minus strand. Novel genes with annotated homologs are colored in blue and novel genes without annotated homologs are colored in orange.

The transcriptome and the translatome of EHEC Sakai were determined at three different growth conditions. Two standard lab conditions (lysogeny broth (LB) at 37°C; Brain-heart-infusion (BHI) at 37°C) and combined cold and osmotic stress (COS; BHI supplemented with 4% NaCl at 14°C) in two biological replicates each. Details about total read number and amount of rRNA, tRNA, and mRNA are listed in S1 Table. All intergenic ORFs of at least 30 AA length were considered as potentially encoding a protein if significant RIBOseq signals were found. A RIBOseq signal was assumed significant at a threshold of at least 1 RPKM, at least 50% ORF coverage, and an RCV of at least 0.25. This analysis resulted in 1271 potentially translated intergenic ORFs, which were manually examined for the following additional criteria before consideration as candidate genes. First, ORFs with identical sequences to others were removed. Next, every ORF with its mapped RIBOseq reads was visualized in the Artemis viewer [41]. False positives were assumed if the signal could have been caused by neighboring annotated genes and not by the putative ORF of interest and, as such were excluded. In the case of same-strand overlapping ORFs in different reading frames, the ORF with the better fit to the RIBOseq signal was selected. After individual inspection in which 806 candidates were excluded, we arrived at a conservative estimate of 465 intergenic ORFs, which were considered to show convincing evidence of translation in the RIBOseq experiments. The novel putative genes were consecutively numbered in the order they appear in the EHEC genome (XECs001-XECs465). The novel genes were approximately uniformly distributed within the whole genome, occurring on both strands of the chromosome (Fig 1). Details about position on the genome, length, RPKM value, coverage, and RCV of all novel genes are found in S2 Table.

**Fig 2:**
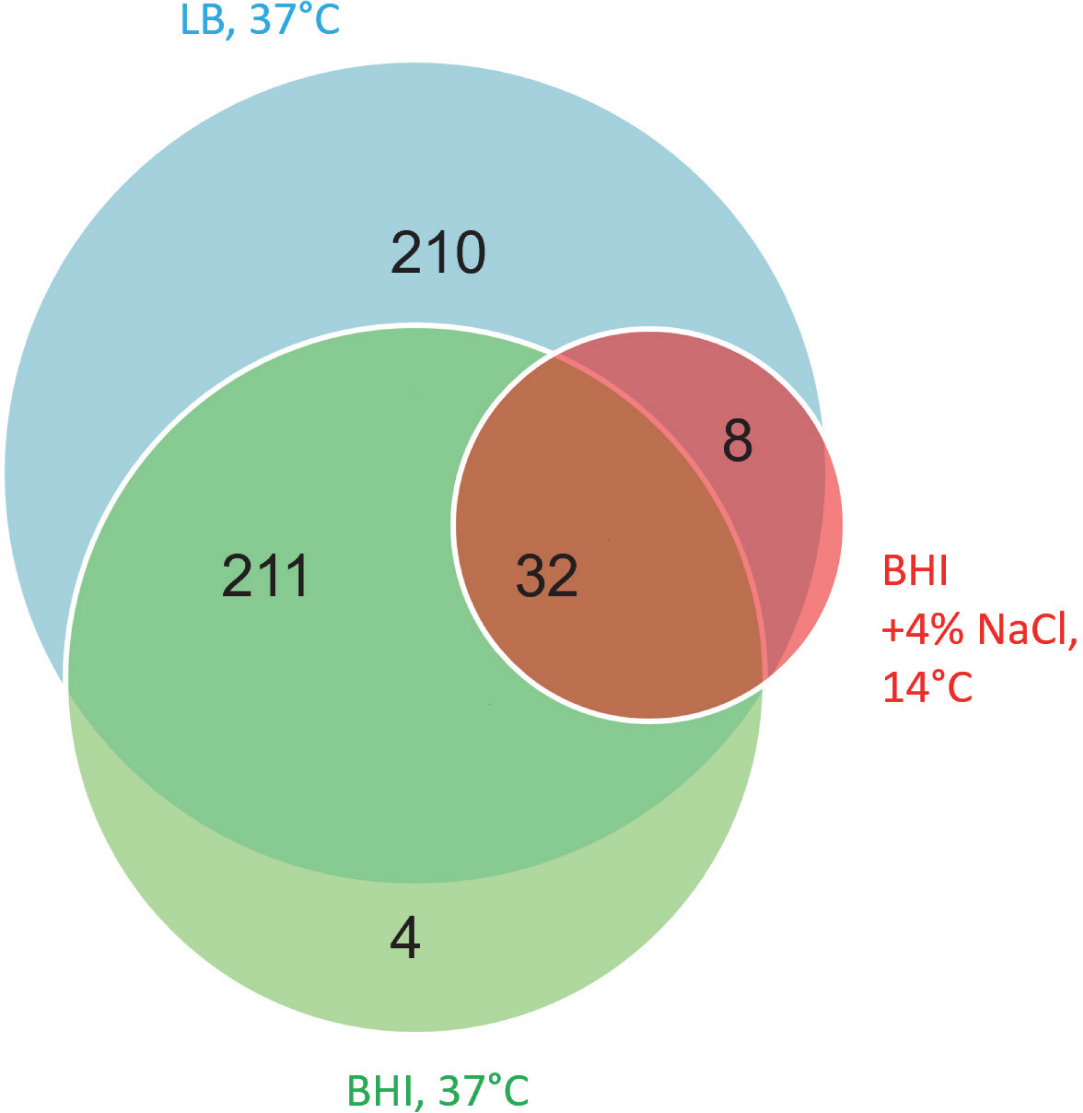
Growth conditions where the novel genes reach or exceed translation thresholds. The Venn-Diagram shows how many ORFs are translated under the three growth conditions investigated. The majority of novel genes are translated at optimal growth conditions leading to a large overlap between LB and BHI control. Blue: LB at 37°C, green: BHI at 37°C, red: BHI + 4% NaCl at 14°C.

**Fig 3:**
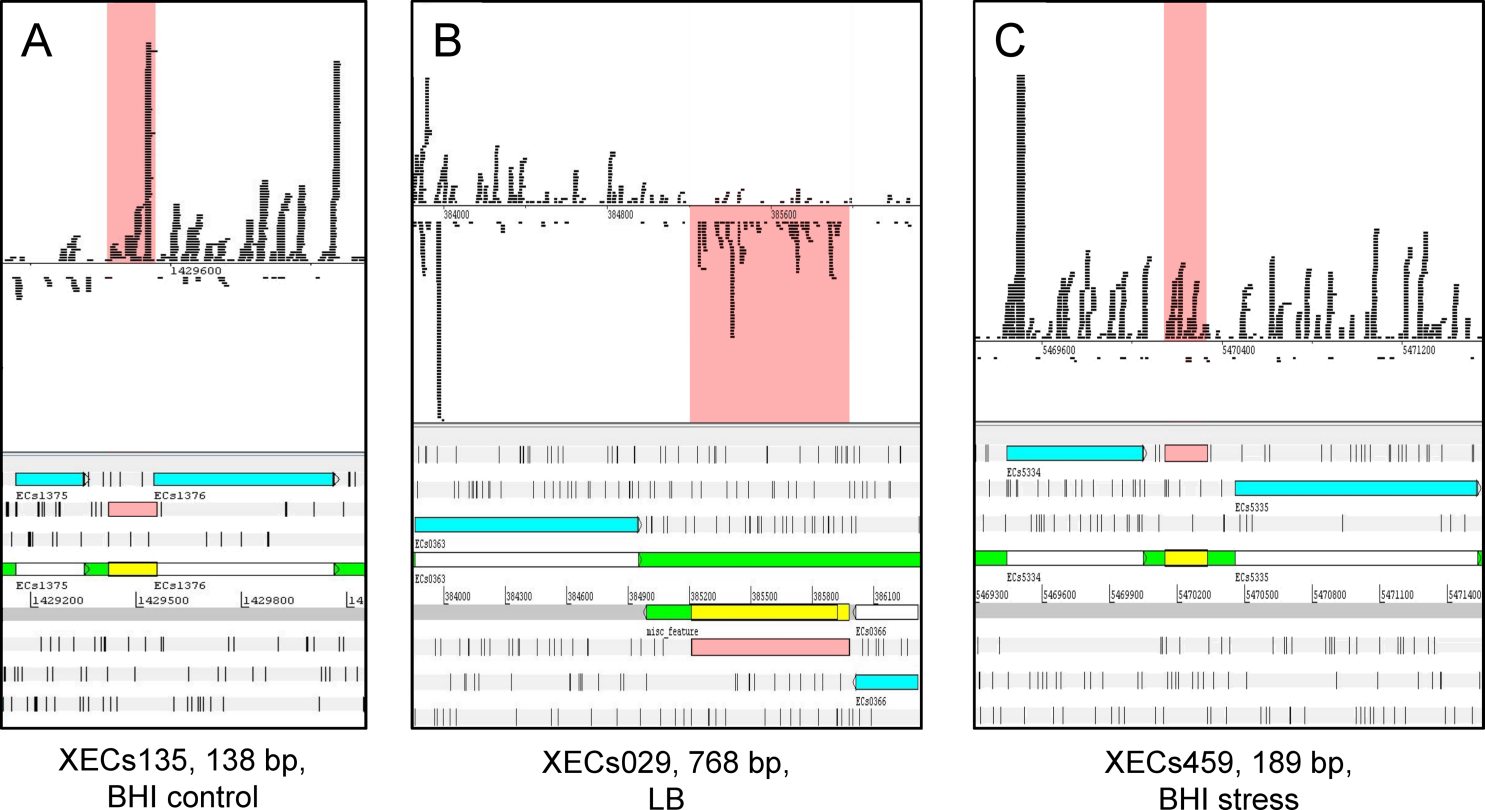
Three novel genes with RIBOseq signals as examples. In the lower part, the corresponding section of the genome is shown with the novel gene highlighted in pink. In the upper part, the strand-specifically mapped RIBOseq reads are displayed, whereby each black line represents a sequenced read.

Two-hundred-eleven (211) novel genes show translation at both optimal growth conditions (LB and BHI at 37°C), 210 novel genes are detected in LB only, and four are detected in BHI control only. RIBOseq signals of 32 novel genes are shared under all three conditions but no gene fulfills the criteria for candidate gene inclusion in BHI COS only (Fig 2 and S2 Table). One example of a translated intergenic ORF for each growth condition is visualized in Fig 3. The three novel gene candidates depicted are clearly covered by RIBOseq reads over their entire length and it is considered highly unlikely that the translation signals are caused by neighboring annotated genes. Additionally, the novel genes show sufficient RCVs of 0.51 (XECs135), 0.58 (XECs029) and 0.29 (XECs459), confirming translation.

### Annotated homologs of novel genes

The amino acid sequences of the novel genes were used as a query to find annotated homologous proteins in other bacteria with blastp using default parameters against the RefSeq database. With an e-value threshold of ≤ 10^−3^, 55% of the putative proteins encoded in the novel genes match an annotated homolog (Table 1). When a more stringent e-value threshold of ≤ 10^−10^ was applied, 42% of novel genes still possess annotated homologs. The hits with the lowest e-value for each novel gene are listed in S3 Table. Interestingly, 34 of the novel genes are annotated in other *E*. *coli* O157:H7 strains, of which twelve were found in the EHEC strain EDL933 [42], which is the closest relative to strain Sakai used in this study. Additionally, eleven of the novel genes detected in the intergenic regions of EHEC EDL933 in a previous study [11] were confirmed for EHEC Sakai, as well.

**Table 1:** Summary of the properties of the short annotated genes, novel genes with annotated homologs and novel genes without annotated homologs.

	(i) Short annotated genes (control group)	(ii) Translated ORFs with annotated homologs	(iii) Translated ORFs without annotated homologs
Number of ORFs analyzed	250	255	210
Mean length [bp]	192	172	127
Mean RCV LB	1.55	2.04	1.74
Mean RCV BHI control	0.55	0.44	0.5
Mean RCV BHI COS	0.12	0.11	0.1
Regulated genes (BHI control versus LB)	82 (32.8%)	103 (40.4%)	76 (36.2%)
Regulated genes (BHI control versus BHI COS)	90 (36%)	210 (82.4%)	170 (81%)
Promoter predicted	242 (96.8%)	242 (94.9%)	210 (100%)
Mean promoter localization (bp upstream start)	187	137	128
Mean promoter strength (LDF score)	3.43	3.44	3.86
Terminator predicted	55 (22%)	53 (20.8%)	32 (15.2%)
Mean terminator localization (bp downstream stop)	68	107	127
Mean terminator score	-16.86	-16.72	-15.87
Shine-Dalgarno (SD) motif predicted	200 (80%)	114 (44.7%)	74 (35.2%)
Mean ΔG° of the SD motifs	-5.17	-4.61	-4.47
Mean SD localization (bp upstream start)	7	8	11
Machine-learning algorithm prediction “real“	248 (99.2%)	226 (88.6%)	5 (2.4%)
Machine-learning algorithm prediction “pseudo”	2 (0.8%)	29 (11.4%)	205 (97.6%)
K_A_>K_S_	7 (2.8%)	0	0
K_A_<K_S_	5 (2%)	12 (4.7%)	5 (2.4%)

**Fig 4:**
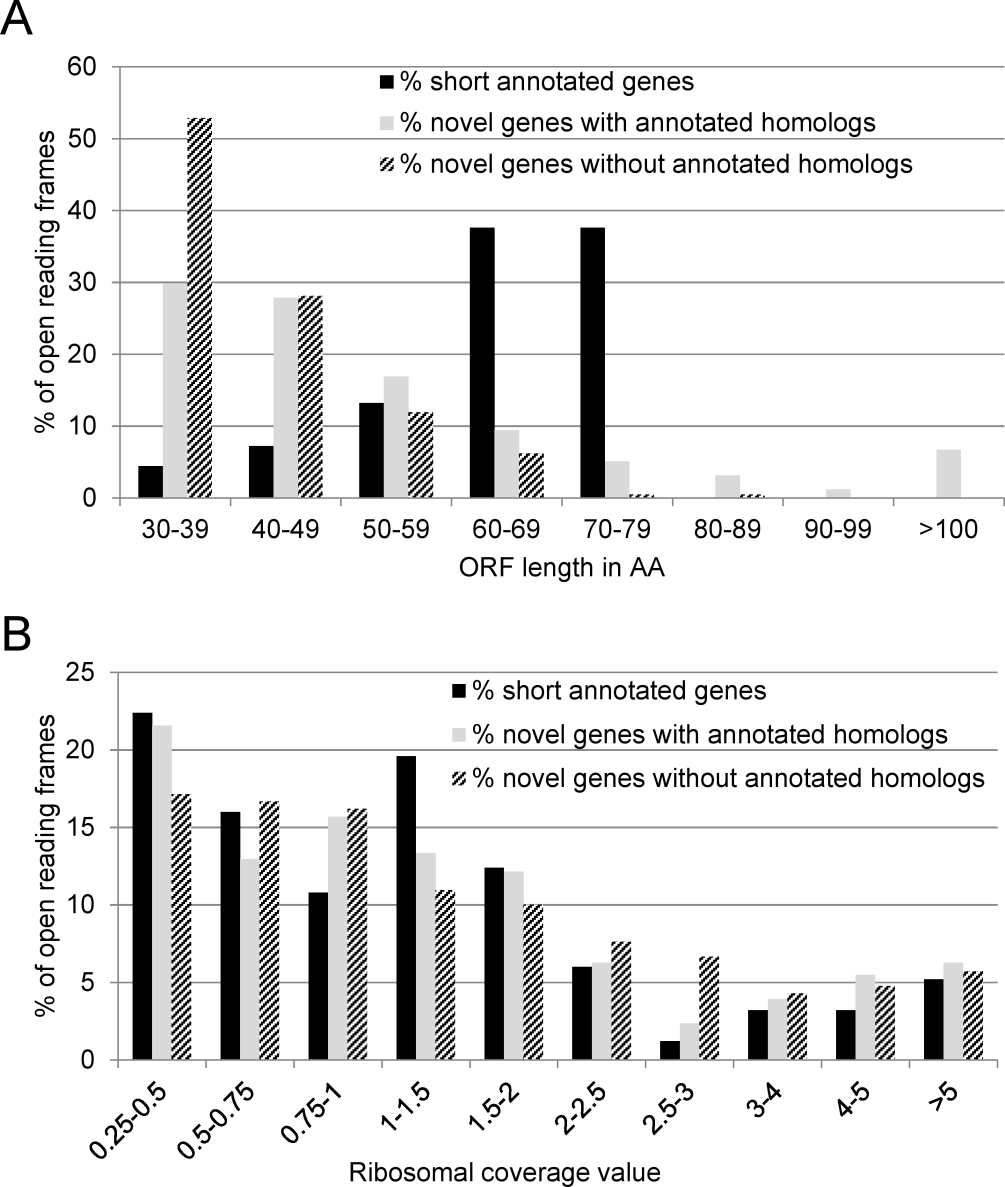
Length and RCV distribution of short annotated genes, novel genes with annotated homologs, and novel genes without annotated homologs. (A) The ORF length in AA was binned into eight categories and the number of ORFs for each gene group belonging to every category was determined. On average, the annotated genes are longer than the novel genes. The novel genes without annotated homologs have the shortest length. (B) The translatability expressed by the ribosomal coverage value (RCV) when growing in LB. The RCV was binned into ten groups. All three gene categories show a similar RCV distribution.

Based on the blastp analysis with an e-value threshold of ≤ 10^−3^, the 465 novel genes were divided into two groups: one group of 255 ORFs, which have annotated homologs in other bacteria (‘with annotated homolog’), and a second group of 210 ORFs for which no annotated homologs were found in the database (‘without annotated homolog’). Furthermore, the 250 shortest annotated genes of EHEC Sakai with an RCV of at least 0.25 in LB (S2 Table and S4 Table) were compared to the two groups of novel genes (see also below; S3 Table). Even though the shortest annotated genes were used, they are on average longer (mean 192 bp) than the novel genes (mean 172 bp). The novel genes without annotated homologs being the shortest, with a mean length of 127 bp (Table 1). More than 50% of the latter group would encode a protein of just 30–39 AA (Fig 4A). However, the largest novel gene would encode a protein of 425 AA. For the three groups, the RCV distribution is shown for LB in Fig 4B. All groups show a comparable pattern: the majority of genes have a moderate translatability and a subset of genes is translated with high efficiency. Growth in BHI control and in BHI COS also yield RCV distributions which are similar among the three gene groups (S1 Fig). Overall, translatability is somewhat decreased under BHI control, but there is a massive decline of translatability under BHI COS condition (Table 1). However, the decline is in a similar range for all three groups and attributable to the stress condition.

### Sequence conservation

**Fig 5:**
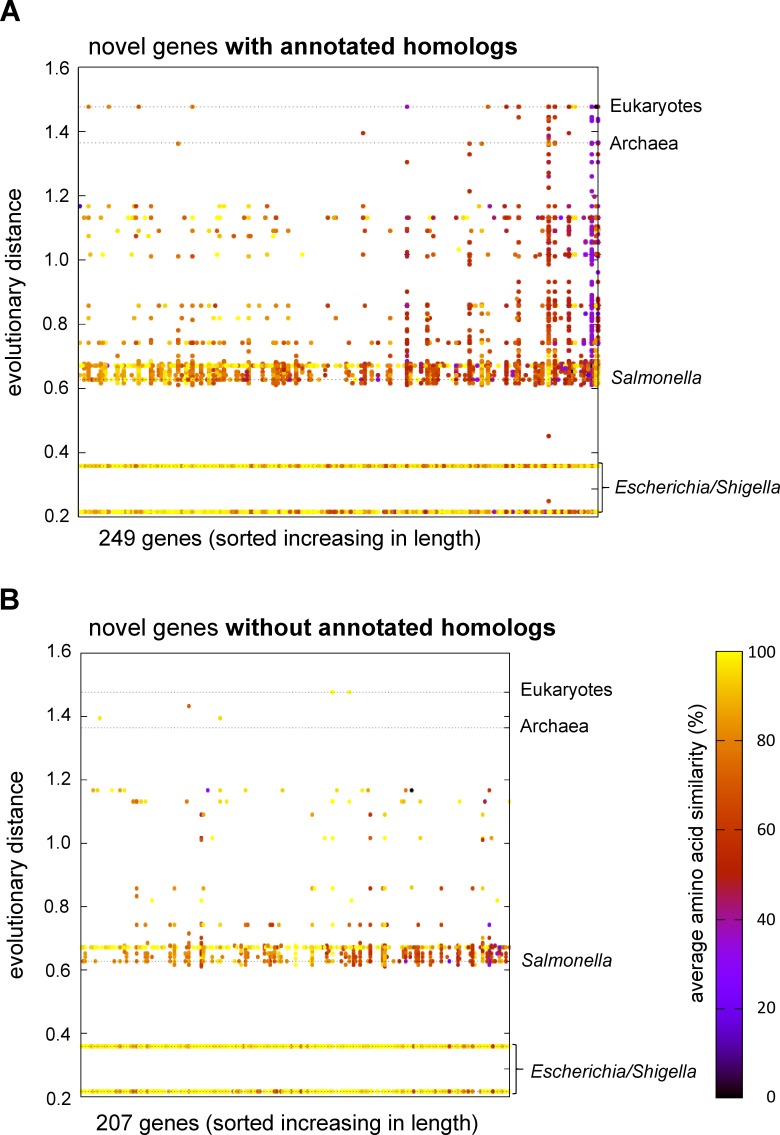
Conservation of novel genes with and without annotated homologs. Average AA sequence similarity (according to the color scale) for all target sequences from a tblastn search of the RefSeq genomic database, for each ORF is shown. Each dot represents a hit in the database for a given novel gene, with points combined and similarity averaged by genus. Novel genes are spread across the X-axis ordered by their length; the Y-axis shows the taxonomic distance of each genus, using the SILVA database 16S rRNA alignment guide tree. (A) Novel genes with at least one annotated homologous protein sequence. (B) Novel genes without annotated homologs. Those with annotated homologs tend to be found across more genera. Note that the number of homologs found in each genus is not indicated, with the vast majority being in *Escherichia* and *Shigella*. Data overview is provided in S5 Table.

A tblastn search for non-annotated homologs of the novel genes in other organisms, using the RefSeq genomic database, shows high conservation levels within the *Escherichia* genus and often more widely (Fig 5). Six novel genes with annotated homologs (blastp) and three putative novel genes without annotated homologs did not have tblastn hits. Thus, 249 and 207 genes with unique sequences are shown in Fig 5A and 5B, respectively. The novel genes with annotated homologs (blastp) show more unannotated homologs (tblastn) with greater average evolutionary distance and AA similarity compared to those novel genes without annotated homologs (blastp). A two-tailed t-test comparing the maximum distance of intact homologs (tblastn) for the novel genes with and without annotated homologs (blastp) gives a *p*-value of *p* = 0.002. Thus, the maximum evolutionary distance of the homologs found using tblastn is significantly different for both groups (i.e., genes with and without annotated homologs using blastp).

There is some evidence for horizontal gene transfer of some ORFs, with highly similar sequences found in distant bacterial genera, and even eukaryotes, for instance multiple matches between XECs029 and *Drosophila* genomes. The sequences in the RefSeq database might be misidentified. However, the phenomenon of transfer of bacterial genome regions to arthropods has been described [43].

Intergenic sequences upstream and downstream of the novel genes were analyzed as above. As expected, sequence similarity is less preserved in the upstream and downstream regions when compared to the ORF-sequence of the novel genes (S2 Fig). For intact homologs (i.e., no stop codon) of the novel genes, the average sequence similarity for intact tblastn hits outside of the *Escherichia/Shigella* genera is 69% (S5 Table). Average sequence similarity for all homologs of the sequences upstream and downstream of the novel genes is lower, at 47% (S2 Fig).

### Triplet periodicity of the RIBOseq signal

**Fig 6:**
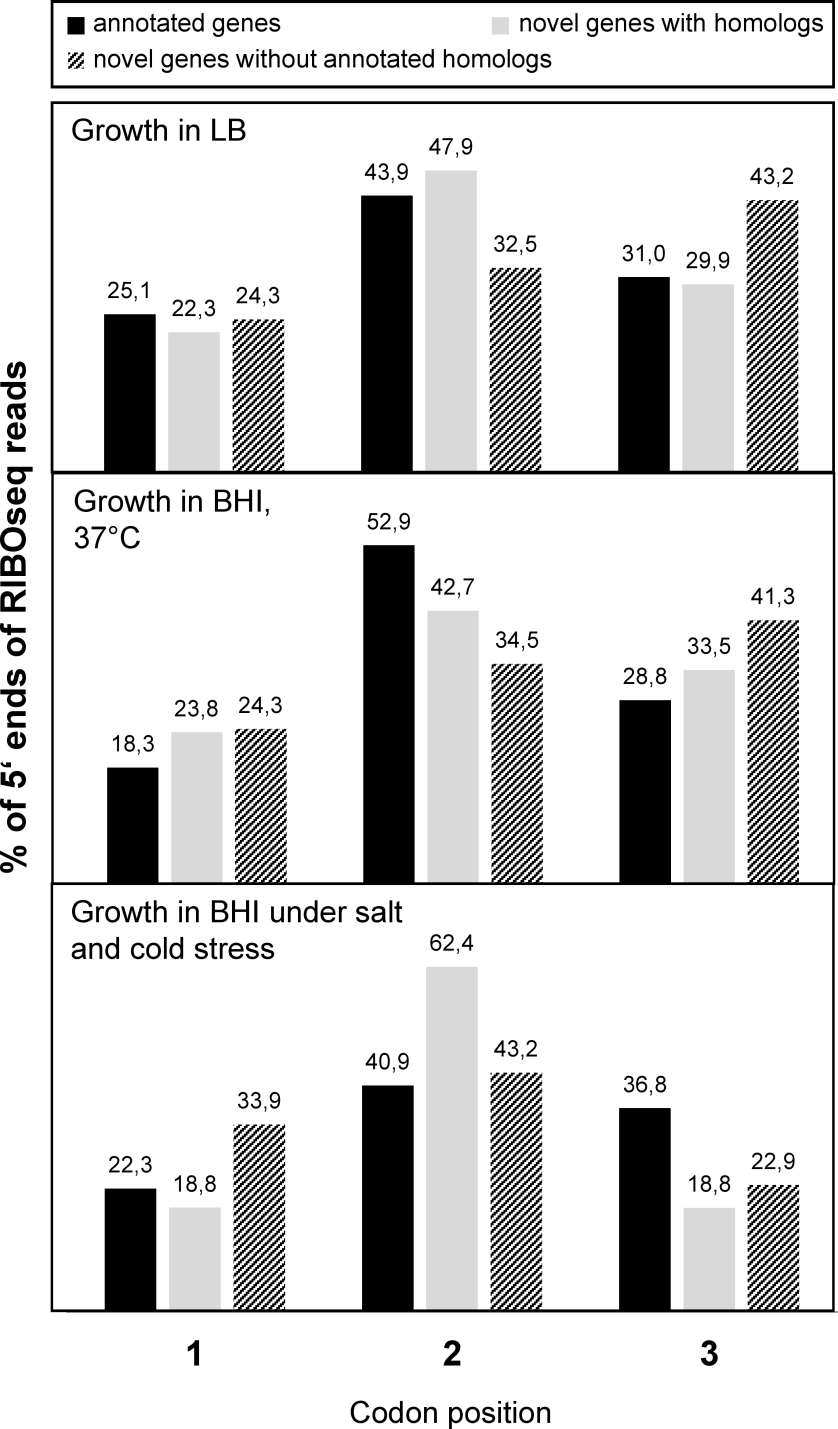
Reading frame in the sum signal of annotated genes, novel genes with annotated homologs, and without annotated homologs. The 5’ ends of RIBOseq reads of length 20 bp were investigated with regard to their codon position. The bar diagrams show the percentage of 5’ ends on every codon position for the three investigated growth conditions. Annotated genes and novel genes with annotated homologs have the majority of 5’ ends at position two for every condition. The novel genes without annotated homologs only show a reading frame at codon position two at the condition BHI + 4% NaCl at 14°C.

A characteristic of RIBOseq data, at least from eukaryotes, is that the reads show a triplet periodicity reflecting the codon-wise translation by the ribosome [31]. Thus, the codon positions of 5’ ends of all RIBOseq reads with read length 20 bp were determined in the sum signal of all annotated genes and of the novel genes with and without annotated homologs. Indeed, the annotated genes and the novel genes with annotated homologs show a reading frame signal at codon position two for all investigated growth conditions (Fig 6). However, the signal is weak and the novel genes without annotated homologs only show a reading frame at codon position two when grown in BHI COS.

### Differential regulation of the novel genes

**Fig 7:**
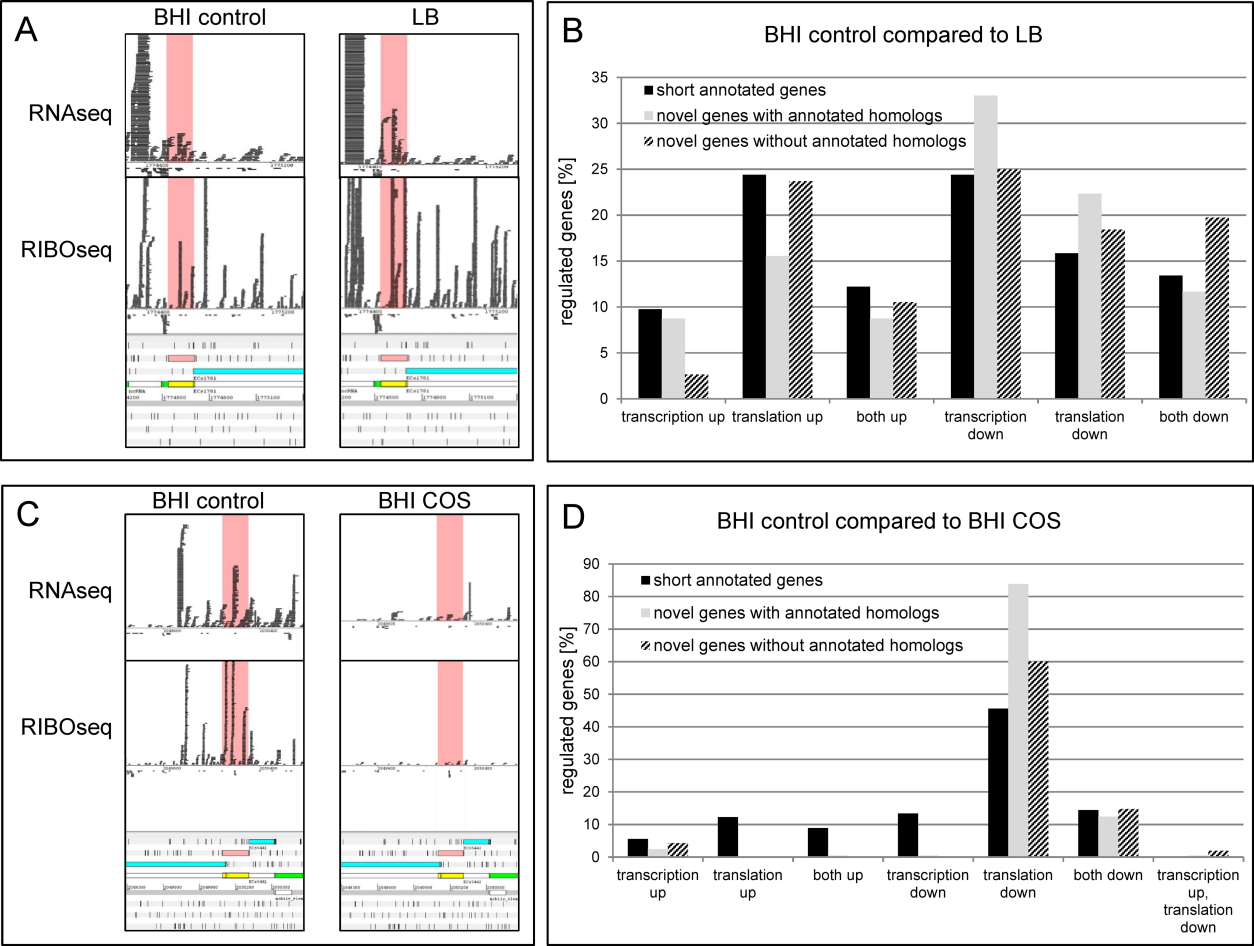
Differentially regulated genes under different growth conditions. (A) Example of a transcriptionally and translationally upregulated gene in LB compared to BHI control. The novel gene XECs170 is highlighted in pink. The transcription of XECs170 is increased 2.7-fold and translation 9.8-fold. (B) Summary of differentially regulated genes in LB compared to BHI control. For all three gene categories, downregulation dominates. (C) Example of a transcriptionally and translationally downregulated gene in BHI COS compared to BHI control. Transcription of XECs197 is 5.5-fold and translation is 129-fold reduced at the stress condition. (D) Summary of differentially regulated genes in BHI COS compared to BHI control. Downregulation at the translational level clearly dominates for all gene categories.

Differential expression at transcriptional and translational levels between growth conditions indicates regulation of gene expression, which implies functionality. Therefore, we investigated the novel genes for significantly changed transcription and translation using BHI control as the reference condition in comparison to LB and BHI COS. In addition, the 250 shortest annotated genes were analyzed as a control group. Comparing growth in BHI and LB medium at 37°C showed that about one third of the genes in each group is differentially expressed (Table 1). XECs170 is an example of a transcriptionally and translationally upregulated novel gene (Fig 7A): the transcription in LB is 2.7-fold increased and the translation is even 9.8-fold higher. For all groups, downregulation in LB is more frequent than upregulation. Downregulation occurs more often at the transcriptional level, whereas for upregulation translational changes are more frequent (Fig 7B). Fold changes, *p*-values and false discovery rates determined with edgeR [44] for all significantly regulated genes are listed in S6 Table.

When the two BHI conditions are compared, even more genes show differential regulation. For example, the novel gene XECs197 is clearly expressed at the control condition, but transcription and translation are almost switched off at BHI COS (Fig 7C). For the short annotated genes, 40% are regulated, but for the novel genes without annotated homologs and the novel genes with annotated homologs 81% and 82.4% are differentially expressed, respectively (Table 1, S7 Table). Although the absolute number of regulated genes is higher for the novel genes, all three gene groups show the same trend (Fig 7D): the majority are downregulated at BHI COS, where translational regulation clearly dominates.

### Bioinformatics analyses

#### Predicted protein characteristics

**Fig 8:**
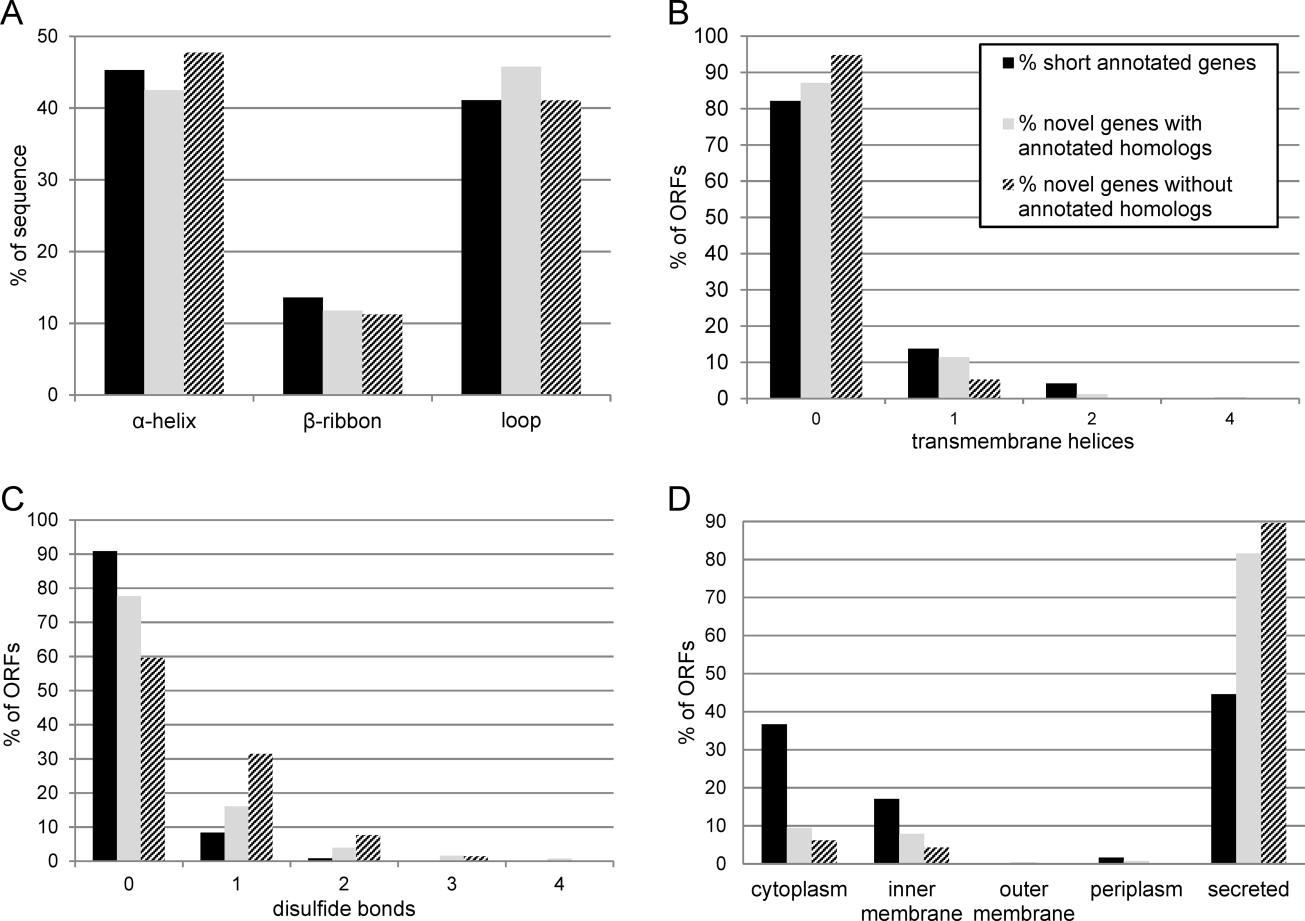
Selected results of PredictProtein for the short annotated genes, and the novel genes with and without annotated homologs. (A) Average secondary structure composition. (B) Number of predicted transmembrane helices. (C) Number of predicted disulfide bonds. (D) Predicted localization of the proteins within the *E*. *coli* cell. Percentage values for every gene separately can be found in S8 Table and S9 Table.

The software PredictProtein [45, 46] predicts many parameters of an amino acid sequence including composition, secondary structure, protein localization, disordered regions, as well as the number of DNA/RNA binding sites, disulfide bonds and transmembrane helices. Prediction of secondary structures is very similar for the three groups (Fig 8A). The proteins mainly fold into α-helices and loops, β-sheet-like structures are less common. Concerning disordered regions, the three groups contain a similar average portion of disorder of about 20% regarding the UCON prediction [47] (S8 Table and S9 Table). Forty-four (9.5%) novel genes show evidence of transmembrane helices (Fig 8B). The proportion of short annotated genes with predicted transmembrane helices is higher (18%). Novel genes with annotated homologs also more often contain a transmembrane helix than do novel genes without annotated homologs (12.9% compared to 5.2%, respectively). For the number of predicted disulfide bonds an opposite picture was obtained. The novel genes without annotated homologs more often have one or more disulfide bonds predicted, followed by the novel genes with annotated homologs, but 90% of the short annotated genes seem not to contain any disulfide bond (Fig 8C). The localization of the putative proteins was also predicted: 34 putative novel proteins should localize in the inner or outer membrane, while surprisingly, 85% are predicted to be secreted (Fig 8D). Whereas the localization prediction of the novel genes with and without annotated homologs is similar, the result for the short annotated genes is slightly different: Many of them should still be secreted (45%), but the number predicted to be cytoplasmic and inner membrane proteins is higher. Further details and additional properties of the novel genes and the short annotated genes are listed in S8 Table and S9 Table.

#### Machine learning trained on known EHEC proteins confirms blastp hits

The above-mentioned parameters were also predicted for a number of short annotated proteins of *Escherichia coli* O157:H7 EDL933 to obtain a positive control set. As a negative control set, these natural proteins were scrambled (for each positive control sequence, 100 randomly scrambled sequences were used) and submitted for PredictProtein analysis. A machine-learning algorithm was trained on the positive and negative control sets to distinguish between ‘real’ protein sequences and scrambled ones (‘pseudo’) [11]. This algorithm was used to investigate the 465 translated ORFs found in this study (S3 Table) and the 250 short annotated genes of EHEC Sakai (S4 Table). Again, every amino acid sequence was scrambled 10-times as a negative control. As expected, the algorithm recognized 99.4% of the scrambled proteins as ‘pseudo’ and 99.2% of the short annotated genes as ‘real’ based on predicted parameters of those sequences. Overall, 50% of the novel genes were recognized as ‘real’. However, the presence of an annotated homolog (found via blastp) correlates well with being predicted as ‘real’ by the machine-learning algorithm and *vice versa* (Table 1, S10 Table). Only five novel genes without annotated homologs were recognized by machine-learning algorithm as ‘real’ proteins. Conversely, 29 novel genes with annotated homologs were predicted as ‘pseudo’ proteins (Table 1 and S3 Table).

#### Promoter and terminator prediction

A promoter is required to initiate transcription of an ORF and is recognized by the σ-factor of the RNA polymerase holoenzyme. The housekeeping σ-factor in *E*. *coli* is σ^70^ (reviewed in [48]). Therefore, σ^70^ promoter sequences were searched in the regions 300 bp upstream of putative start codons of the novel genes using BPROM. Interestingly, all novel genes without annotated homologs have a predicted promoter in their upstream region and in the upstream regions for the novel genes with annotated homologs a promoter sequence appears to be present in 95% of the cases (Table 1 and S3 Table). On average, the predicted promoter sequence localizes 187 bp upstream of the start codon for the annotated genes. In the case of the novel genes, the distance to the start codon is slightly shorter. The LDF score is a measure of the promoter strength and a promoter is considered active with an LDF score of at least 0.2. The average LDF score of the predicted promoters for the three gene groups is similar: 3.43 for the short annotated genes, 3.44 for the novel genes with annotated homologs and 3.86 for the novel genes without annotated homologs, respectively (Table 1).

Transcription termination mediated by ρ-independent terminators [49] in the region 300 bp downstream of the stop codon was investigated using FindTerm. For 20.8% of the novel genes with annotated homologs a terminator was predicted. For those without annotated homologs, the fraction was slightly lower (Table 1).

#### Shine-Dalgarno sequence and start codons

The presence of a Shine-Dalgarno (SD) sequence upstream of the start codon promotes efficient translation initiation [50]. The consensus SD motif for *E*. *coli* is uaAGGAGGu and base pairing of this sequence with the anti-SD of the 16S rRNA results in a free energy of ΔG° -9.6 [51]. Within the region 30 bp upstream of the start codons 41% of the novel genes with annotated homologs and 35.2% without annotated homologs have a SD sequence (Table 1). A high proportion of the annotated genes have a SD sequence (80%). Additionally, the average free energy of the SD is lower for the annotated genes (-5.17 compared to -4.61 and -4.47, respectively). The upstream regions of XECs059 (novel gene with annotated homolog) and XECs428 (novel gene without annotated homolog) contain a perfect SD sequence (S3 Table).

ATG is the most common start codon, but also GTG, TTG, and the rare start codons CTG, ATT, ATA, and ATC can initiate translation in *E*. *coli* [52]. Genome annotation algorithms only search for the three most common start codons (ATG, GTG, and TTG, respectively) [12] and in accordance with this, the group of the annotated genes shows for 90% of genes an ATG start codon, for 7.2% a GTG start codon, and for 2.8% a TTG start codon, whereas rare start codons are not present at all. In case of the novel genes, the real start codon is unknown. Because of that the potential start codon farthest upstream of the coding region, but within the transcriptome signal, was chosen no matter whether it was a frequent or rare start codon. Therefore, only 42% of the novel genes with annotated homologs and 32.8% of the novel genes without annotated homologs start with either ATG, GTG, or TTG. All other genes, putatively, have rare start codons. However, it cannot be excluded that some of these genes possess an ATG, GTG, or TTG start codon further downstream of the open reading frame.

#### Evolutionary sequence analysis of novel genes

The rates of non-synonymous (amino acid changing) and synonymous (not amino acid changing) substitutions per site, k_A_ and k_S_ respectively, reflect the evolutionary processes underlying the divergence of related genes. In the absence of selection, it is expected that k_A_ ≈ k_S_, indicating neutrality. On the other hand, when purifying selection acts to eliminate disadvantageous mutations, the fact that most fitness-altering mutations are nonsynonymous implies that selection will disproportionately slow the rate of divergence at nonsynonymous sites, leading to k_A_ < k_S_. On the other hand, when positive selection acts to promote advantageous mutations, this will disproportionately increase the rate of divergence at non-synonymous sites, leading to k_A_ > k_S_. Although intergenic junk sequences are expected to evolve neutrally, functional genes can also exhibit k_A_ ≈ k_S_ because of near-neutrality or a balance between positive and purifying selective forces. We reasoned that only functional protein-coding sequences would show significant signs of positive or negative selection and, based on the hypothesis that our novel genes are functional, we predicted that the proportion of genes exhibiting significant signatures of selection should be similar between novel candidate genes and annotated genes.

To test this hypothesis, the most distant homologous sequences matching the genes, with 100% coverage and no gaps, were identified using tblastn. Due to the short size of most of the genes, many sequences were too similar for a k_A_/k_S_ comparison, leaving 175 of 250 annotated genes, 153 of 255 novel genes with annotated homologs, and 116 of 210 novel genes without annotated homologs available for analysis (S3 Table and S4 Table). Of these remaining genes, 12 (4.8%), 12 (4.7%), and 5 (2.4%) genes showed significant selection in the three respective classes using a Holm-Bonferroni multiple comparisons procedure, which was not a significant difference between classes (*p* = 0.335, Fisher’s Exact Test). However, only annotated genes exhibited any genes under significant positive selection (5 genes), which was a significant difference among classes (*p* = 0.001, Fisher’s Exact Test; Table 1).

## Discussion

### RIBOseq is a powerful tool to detect translated mRNA

Ribosomal footprinting has been used to detect translation of non-annotated ORFs previously. In eukaryotes, hundreds of non-annotated ORFs show evidence of translation, e.g., in yeast [53], in *Drosophila* [54], in zebrafish [34], in *Arabidopsis* [37], and even in humans [55]. Additionally, the translation of previously annotated ncRNAs was reported frequently [36, 39, 56]. In bacteria, 130 novel genes were detected in *Salmonella* [40] and 72 novel genes were detected in EHEC strain EDL933 [11]. For the latter strain, translation is also reported for a number of RNAs that were previously classified as ncRNA. For instance, the ncRNA *ryhB* encodes a nonamer peptide RyhP [39]. Although it was not the focus of their study, Jeong et al. [57] report translation signals for 31 annotated ncRNAs in *Streptomyces coelicolor*. Even the well-studied λ phage with a very small genome of 48.5 kB shows translation of 50 non-annotated ORFs [58].

RIBOseq experiments with eukaryotes allow reading frame determination for individual genes [33, 37, 38]. The reading frame resolution of prokaryotic RIBOseq data is lower such that we cannot determine a reading frame in the RIBOseq signal of single ORFs. This may be caused by bacterial ribosomes being more flexible and incorporating changing numbers of mRNA nucleotides [59]. In addition, the RIBOseq method, formerly developed for eukaryotes, has been adapted for bacteria and footprints of more variable read length are obtained [60]. Furthermore, the composition of ribosomal proteins and rRNAs can be heterogeneous dependent on the growth condition; especially at stress conditions, specialized ribosomes are responsible for the translation of a subset of mRNAs [61, 62]. Putatively, the specialized ribosomes protect an mRNA stretch of deviating length. Recent findings indicate that the usage of a translational inhibitor influences ribosome conformation, which weakens the reading frame signal [63]. For instance chloramphenicol, as used in this study, preferentially arrests translation at positions encoding alanine, serine, or threonine [64] which dilutes the triplet signal. Also, the choice of the ribonuclease used for digestion of mRNA not protected by ribosomes influences RIBOseq results [65]. To minimize the influence of any sequence specificity for a single RNase, we applied a mixture of five RNases (RNase I, MNase, XRN-1, RNase R, and RNase T). Here, we show a reading frame in the sum signal for all genes for the first time in bacteria using conventional RIBOseq. Very recently, the addition of the endonuclease RelE to the ribosome preparation has been reported to improve reading frame determination. The RelE toxin cuts the mRNA within the ribosome very precisely at a specific position in the codon [66]. However, as shown in Fig 6, under our three conditions a reading frame in the sum signal can be extracted from the data, at least for the group of novel genes that have annotated homologs in other bacterial strains or species.

### RIBOseq based evidence for translation of 465 intergenic ORFs

In this study, 465 intergenic ORFs have been detected, which show a clear RIBOseq signal (S2 Table). The average size of the novel-gene encoded proteins is only 50 AA. Standard genome annotation algorithms do usually not predict such very short genes or proteins [14, 16]. In this study, an arbitrary size minimum of 30 AA was applied to restrict the number of ORFs to be investigated and to reduce the possibility of false positives, but even smaller peptides can be functional [39, 40]. Knowledge about the functions of small proteins in bacteria is limited, but small proteins have recently achieved attention (reviewed in [15, 18]). For instance, Baumgartner et al. [67] confirmed five small proteins in *Synechocystis* by Western blot. Neuhaus et al. [11] detected 72 novel small genes in the intergenic regions of the *E*. *coli* strain EDL933 by evaluating RNAseq and RIBOseq data of a single growth condition (LB, 37°C). Compared to their work, this study on a different EHEC strain achieves a higher sequencing depth and two additional growth conditions including severe stress were investigated. Moreover, translated ORFs were not only selected by an RPKM-value threshold, but further conservative thresholds for coverage and RCV were applied. Translation of eleven novel small genes found in EHEC EDL933 by Neuhaus et al. [11] is present in EHEC Sakai and twelve translated ORFs of EHEC Sakai are annotated proteins in EDL933. Vice versa, 28 of the 72 novel EDL933 genes are annotated proteins in strain Sakai.

### The 255 translated ORFs with annotated homologs most likely represent protein-coding genes

Blastp analysis revealed that a group of 255 out of the 465 novel ORFs with a clear RIBOseq signal found in this work, have annotated homologs in other bacteria. In addition, many of these 255 genes display predicted protein structures (Fig 8), as well as σ^70^ promoters, and in some cases ρ-independent terminators and SD sites, like annotated short proteins. Even ORFs without these predicted extra features can encode proteins, because those genes could be part of an operon, the promoter could be recognized by an alternative σ-factor [68], termination could be ρ-dependent [69], and translation of leaderless mRNAs occurs [70]. Overall, these novel genes behave similarly in all parameters investigated when compared to 250 short annotated genes of EHEC Sakai. Both gene groups are transcribed and translated at the same magnitude and the RCV distributions of all growth conditions are comparable. A similar fraction of genes is differentially transcribed and/or translated, when BHI control is compared to BHI COS or LB. Even the directions of up/down regulation compare well (Fig 7). Additionally, active translation is supported by the presence of a reading frame on codon position two for every growth condition in the sum signal caused by codon-wise progression of the ribosome. Furthermore, a machine-learning algorithm trained with short annotated proteins of EHEC EDL933 predicted 88.6% of these genes with annotated homologs as being ‘real’ proteins. Finally, there is no significant difference between the number of genes under selection in this class as compared to either annotated genes or novel genes without annotated homologs. However, unlike annotated genes, for which the majority of selected genes showed evidence of positive selection, all selected genes in this class were under purifying selection. This is not unexpected under the hypothesis of functionality, because purifying selection is the most common form of selection in nature [52], and because this result was obtained despite choosing the most distant homolog. However, it is also likely that ascertainment bias plays a role in this result, as it is probable that more emphasis has historically been placed on the annotation of genes which are shared by more distantly related organisms. This would especially be true if many of the novel genes we identified are orphan genes, since such genes lack distantly related homologs by definition. Therefore, we conclude that these 255 translated intergenic ORFs indeed represent novel small protein-coding genes of EHEC strain Sakai.

### Unusual features of the 210 novel genes without annotated homologs

A second group, 210 out of 465 novel genes, had no annotated homologs when using blastp. However, homologs in other bacteria may be present but were missed during annotation of these genomes due to their unusual features. Indeed, a tblastn search confirmed that many non-annotated homologs in the *Escherichia* genus and, in some cases, in farther related species as well, exist (Fig 5B). The majority of these ORFs were not classified by the machine-learning algorithm to encode ‘real’ proteins. This appears to be more significant and raises the question whether these ORFs indeed code for proteins. The following analysis is based on a comparison between three groups: (i) 250 annotated small genes, (ii) 255 novel small genes with annotated homologs and (iii) the group of 210 ORFs without annotated homologs, which may or may not code for proteins (Table 1). Several arguments support the hypothesis that these ORFs are functional and not residues due to pervasive transcription [29]: first, their expression obviously does not lead to a fitness disadvantage, as in misfolded proteins, which are cytotoxic [71]. Second, a promoter is present upstream of all 210 ORFs, and thirdly, the same fraction of these ORFs is differentially transcribed, compared to both control groups (i) and (ii) (Fig 7). However, these data would fit the hypothesis either that these ORFs represent ncRNA or that they are protein-coding genes. The following observations are in favor of the hypothesis that these novel ORFs are protein-coding genes and not ncRNAs: most significantly, RIBOseq signals, and hence significant RCVs, are in the same order of magnitude as those of short annotated genes, many ORFs without homologs are differentially regulated at the translational level, SD sequences are present upstream of one third of the ORFs, and the number of predicted protein structures is very similar to that of annotated protein-coding genes. Finally, a similar proportion of genes appear to be under selection as among the annotated genes and novel genes with annotated homologs, with the caveat that ascertainment bias has likely favored the detection of genes under purifying selection.

Why, then, does the machine-learning algorithm not recognize these ORFs as protein-coding genes? A first explanation is that the algorithm will only predict sequences as ‘real’, which are within the known parameter space of the training set. Proteins of unknown structure and folds may reside outside the parameter space of ‘established’ proteins and, thus, will fail to be classified as ‘real’ and inevitability binned as ‘pseudo’. The majority of all established proteins belong to a protein family with known secondary structure or which contains characterized domains. But 25% of all protein sequences do not match to any family and, therefore, belong to the ‘dark proteome’ [72]. In prokaryotes, 13% of all proteins are ‘dark’ [20]. Their properties are different when compared to known proteins: They are shorter, they are often secreted, contain more disulfide bonds, have a lower evolutionary reuse [20], are more disordered, have a different hydrophobic amino acid topology, and have a higher energy [21]. Many of these properties fit well with the PredictProtein data of the proteins encoded by the novel genes without annotated homologs: accordingly, the majority of putative proteins without annotated homologs are very short, are predicted to be secreted, and more often contain disulfide bonds. Thus, these properties render it unlikely that the machine-learning algorithm will predict these unusual proteins correctly.

A second possibility is that the novel genes without annotated homologs may represent very young taxonomically restricted or ‘orphan’ genes. Yomtovian et al. [73] reported that orphan genes of EHEC show an amino acid composition more comparable to random sequences than to annotated genes, since they may not yet have a fully adapted function, which makes it difficult for any annotation program, including our machine-learning algorithm, to distinguish them from scrambled proteins. Also, young genes without annotated homologs are shorter [74], which is true for our data set. Additionally, evolutionary young genes often use uncommon start codons [75], which is also true for our data set. This hypothesis is further supported by the evolutionary distances of the non-annotated homologs detected using tblastn, when comparing the novel genes without annotated homologs to the novel genes with annotated homologs (Fig 5). The genes with annotated homologs show intact tblastn hits (i.e., ORFs without stop codons) with a significantly greater evolutionary distance compared to the genes without annotated homologs.

In summary, we believe that our data provide evidence supporting the hypothesis that most of these 210 ORFs are evolutionarily young genes coding for proteins with unusual features. The data set may contain some false positives, since in a few cases, ribosome binding of the RNA may exert a regulatory function, comparable to a translation regulating riboswitch instead of translation into protein [76, 77]; however, this will not invalidate our general findings.

## Conclusion

This study supports the fact, that, in contrast to earlier beliefs, bacterial genomes are probably under-annotated due to small genes having been overlooked. In *E*. *coli* O157:H7 Sakai, at least 465 non-annotated short ORFs are covered with significant RIBOseq reads indicating active translation and the majority of these ORFs show features of protein-coding genes. Since the EHEC Sakai genome harbors about 5200 annotated protein-coding genes, these additional genes would significantly increase the number of protein-coding genes in this bacterium. Obviously, much further work is required for functional characterization of the novel genes. It would not be surprising if other bacterial genomes also harbor many overlooked short genes in their intergenic regions, which could be investigated by combined RNAseq and RIBOseq. In addition, the high-throughput discovery of small proteins in proteome analysis requires modified or improved methods since these proteins likely escape attention with most currently available methods [17, 78, 79]. Our study supports the notion that it is advisable to improve genome annotation algorithms in order to reduce bias against annotation of short genes [16, 75].

## Material and methods

### Transcriptome and translatome sequencing

Strand-specific RNAseq and RIBOseq of *Escherichia coli* O157:H7 Sakai (GenBank accession number BA000007.2 and RefSeq accession NC_002695.1, version from February 2014) [1] were performed at three different growth conditions in two biological replicates each. An overnight culture of EHEC was inoculated 1:100 in lysogeny broth (LB medium) and incubated at 37°C and 150 rpm until an OD_600_ of 0.4 was reached. Additionally, two conditions using brain-heart infusion broth (BHI; Merck KGaA) were investigated. For the BHI control condition, an overnight culture of EHEC was inoculated 1:100 and incubated at 37°C and 150 rpm until an OD_600_ of 0.1 was reached. For the stress condition of combined cold and osmotic stress (COS), 4% NaCl were added to the BHI medium and incubation was performed at 14°C until an OD_600_ of 0.1 was reached.

RNAseq was performed as described by Landstorfer et al. [8] for the Illumina system. For ribosomal footprinting, the method published by Ingolia et al. [31] was adapted to bacteria as described [11] with the following further modifications: mRNA not protected by ribosomes was digested with a mixture of five RNases to exclude sequence specificity. Buffer NEB 4 plus 1 mM CaCl_2_ was added to 1 ml cell extract and the solution was incubated for 1 h at RT with 250 U MNase (Roche), 5 U XRN-1 (NEB), 250 U RNase I (Thermo Fisher Scientific), 50 U RNase R (Biozym) and 12 U RNase T (NEB). The monosome fraction was harvested by sucrose density gradient centrifugation and unprotected mRNA digestion was repeated once. For the LB condition, rRNA was depleted using the MICROBExpress kit (Thermo Fisher Scientific) and for the BHI conditions rRNA depletion was performed using the RiboZero kit for Gram-negative bacteria (Illumina). All libraries were prepared using the TruSeq Small RNA Sample Preparation Kit (Illumina) and sequenced on a HiSeq 2500 machine according to the manufacturer. The sequencing raw data is available at the Sequence Read Archive (SRA, NCBI) under the accession SRP113660.

### Read mapping and RCV calculation

For processing and mapping of the sequencing raw data, the Galaxy platform was used [80] as described [11]. The data were visualized using BamView [81] implemented in Artemis 16.0 [41]. The RPKM values for all intergenic non-annotated ORFs in EHEC which would encode a peptide of ≥ 30 AA (~12,000 ORFs) were calculated in R, whereas reads mapping to rRNA or tRNA were excluded [82]. Besides the canonical DTG start codons, the rare start codons CTG, ATT, ATA and ATC were allowed according to genetic code table 11 (https://www.ncbi.nlm.nih.gov/Taxonomy/Utils/wprintgc.cgi). The ratio of RPKM translatome over RPKM transcriptome gives the ribosomal coverage value (RCV), which is a measure for the translatability of a certain ORF [39]. Novel gene candidates had to fulfill the following criteria for at least one growth condition in both biological replicates to be considered translated: RPKM translatome at least 1 read per million mapped reads, coverage translatome ≥ 0.5 and RCV ≥ 0.25. To exclude false positives, all novel gene candidates were manually inspected in Artemis.

### Reading frame determination

Adapter removal and quality trimming were performed using AdapterRemoval v2.1.7 [83] and non-rRNA reads longer than 18 bp were extracted using sortMeRNA v2.0 [84]. Extracted reads were mapped to previously annotated genes, novel genes with annotated homologs and novel genes without annotated homologs, in *Escherichia coli* O157:H7 Sakai using Vsearch v2.1.2 [85]. The reading frame of the 5’ end of each mapped read of length 20 bp (maximum of read length distribution) was determined using a custom script (S1 File), which counts the number of 5’ ends for the three codon positions and sums the values for the three gene groups (annotated genes, novel genes with annotated homologs, and novel genes without annotated homologs).

### Differential gene expression

The condition ‘BHI at 37°C’ was used as the reference data set and for the LB and BHI COS conditions significant changes on transcriptional and translational level were determined. Read counts were normalized to the smallest library and differential expression was analyzed by an exact test implemented in the *Bioconductor* package *edgeR* (version 3.2.4) [44]. A *p*-value ≤ 0.05 and a false discovery rate (FDR) ≤ 0.1 were used to delineate significant expression changes.

### Prediction of σ^70^ promoters

The region 300 bp upstream of the start codon was searched for the presence and strength of a σ^70^ promoter with the program BPROM (Softberry [86]). It searches for the -35 and -10 consensus motif and recognition sequences for transcription factors. With this data, an LDF score (linear discriminant function) is calculated, whereupon increasing values indicate growing promoter strength. An LDF score of 0.2 gives the threshold for promoter prediction with 80% accuracy and specificity.

### Prediction of ρ-independent terminators

The region 300 bp downstream of the stop codon was searched for the presence and strength of a ρ-independent terminator using FindTerm (Softberry [86]). This program searches thymidine-rich regions, and calculates the energy of possible terminator structures. Low energy values indicate strong terminators.

### Prediction of Shine-Dalgarno sequence

The region 30 bp upstream of the start codon was examined for the presence of a Shine-Dalgarno sequence (optimum uaAGGAGGu). ΔG° was calculated according to Ma et al. [51] with a threshold of ≤ -2.9 kcal/mol.

### Calculation of k_A_/k_S_

The most distantly related homologs of the short annotated genes and the novel genes were determined with tblastn by selecting the hit with the highest e-value which still has 100% coverage and no gaps. In case the sequence pairs were too similar, meaningful k_A_/k_S_ calculation was not possible. The ratio of synonymous to non-synonymous substitutions between those gene pairs was computed using the KaKs_Calculator 2.0 [87]. The “bacterial and plant plastid code” was selected and the method model selection (MS) was used. The ORF is assumed to be under positive selection when k_A_/k_S_ is significantly greater than 1 and under purifying selection when k_A_/k_S_ is significantly less than 1. Significance was determined using a Holm-Bonferroni multiple comparisons procedure with respect to the family, an error rate of 0.05. A Fisher’s Exact Test was performed in R version 3.3.2. Unless otherwise noted, all *p*-values refer to two-sided tests.

### Detection of annotated homologs

Novel gene sequences were translated into the corresponding proteins sequences, which were used to query the GenBank database using blastp with default parameters [88]. An e-value cutoff of 10^-3^ was applied.

### Sequence conservation

Sequences of the novel genes were aligned against the full RefSeq genomic database downloaded on 5 April 2017, using a tblastn search in the local BLAST utilities 2.6.0+ from the NCBI [89] with a maximum e-value of 0.001. The putative homologues were extracted from the database and those without stop codons were retained as ‘intact’. The amino acid similarity of each intact subject sequence with the query ORF was calculated using the Needle-Wunsch algorithm “Needleall” from EMBOSS [90]. The *Achromobacter* sp. ATCC35328 sequences with names beginning NZ_CYUC010 were removed from the analysis, due to abnormally high similarity with *E*. *coli* for a very large number of genes. Thus, we assumed this species to be mislabeled. To map the results gained using NCBI databases to the SILVA taxonomy, hits were conflated to genus level, which allowed inclusion of over 90% of genera with hits in each case. To obtain approximate relative evolutionary distances, the average distance from EHEC Sakai to the last common ancestor with each genus was calculated from the 16S rRNA SILVA reference NR99 guide tree [91], release 128, using Newick Utilities [92]. A custom shell script for these tasks, ORFage, was used (S2 File). A similar pipeline was used to check the conservation of intergenic sequences upstream and downstream of the novel genes. For the upstream regions, the sequences between the stop codon of the nearest annotated gene upstream of the start codon of the novel gene was taken. Similarly, for downstream regions, the sequence between the stop codon of the novel gene and the start codon of the next annotated gene downstream was taken. Some of the regions were too short to obtain (meaningful) tblastn hits and were excluded. Further regions were excluded, when containing another of the novel genes before an annotated gene was reached. One downstream sequence was abnormally long and could not be processed (tblastn search > 1 day), hence, this region was also excluded. Within the upstream and downstream sequences, stop codons were allowed. The shell script used for preparation of the intergenic sequences including the use of ENTREZ DIRECT [93] is included in S3 File.

### Predicted protein characteristics

The amino acid sequences encoded in the 250 short annotated genes and the 465 novel genes were submitted to PredictProtein [46] using default parameters. This software predicts structural and functional features of the putative proteins. The results of PROFphd (secondary structure) [94], TMSEG (transmembrane helices) [95], DISULFIND (disulfide bonds) [96], UCON (disordered regions) [47] and LocTree3 (subcellular localization) [97] were analyzed in further detail.

### Machine learning based protein recognition

A machine-learning algorithm, as described by Neuhaus et al. [11], was used to classify the novel proteins based on predicted protein parameters. Briefly, about 279 short annotated proteins were picked from EHEC EDL933 and these sequences shuffled 100-times. All sequences, natural and shuffled, were submitted to a PredictProtein analysis [45, 46]. The machine-learning algorithm was trained using the predicted parameters for the annotated proteins (positive control) and their shuffled counterparts (negative control). Both strains, EDL933 and Sakai are very closely related to each other [98] and, thus, the trained algorithm was used here, as well. We not only examined the protein sequences of the novel genes in Sakai, but also shuffled those 10-times to detect false positives.

### Localization of novel genes

Visualization of the gene’s localization was created using Circos [99].

## Supporting information

S1 Fig Distribution of RCV for the short annotated genes, novel genes with and without annotated homologs. (A) RCV distribution at BHI control. (B) RCV distribution at BHI COS.
(PPTX)

S2 Fig Conservation of intergenic sequences. A similar process as used for Fig 5 was repeated on unannotated sequences upstream and downstream of the novel genes, but without removing sequences with stop codons. Many of the sequences had no tblastn hits (too short) and some others were excluded as more than one novel gene was situated between two annotated genes; one was excluded as abnormally long. Thus, 136 sequence remained for upstream and 122 for downstream. Most homologs have low similarity. The custom shell script used is provided in S3 File. (A) Analysis of the sequences upstream of the novel genes without annotated homologs. (B) Analysis of the sequences downstream of the novel genes without annotated homologs.
The average evolutionary distance to the tblastn hits (of at least 80% similarity) of the novel proteins without annotated homologs (blastp) is 0.643. Average distance for their downstream sequences is 0.535, which is significantly lower (*p* = 0.0024, two-tailed t-test). Average in evolutionary distance for upstream regions is 0.596, not significantly different compared to distances for genes (*p* = 0.1421). The upstream region may be more conserved (e.g., due to regulatory sequences contained).
(PPTX)

S1 Table Summary of NGS results. The total number of reads, the number of reads mapping to the *E*. *coli* O157:H7 Sakai genome and the distribution of mapped reads to rRNA, tRNA and mRNA are shown. Only the reads mapping to mRNA were used for further analysis. Every library contains between 1.5–9.7 m. mRNA reads.
(DOCX)

S2 Table RNAseq and RIBOseq results of three different growth conditions for the 465 novel genes and the 250 short annotated genes. The novel genes are consecutively numbered after their appearance in the EHEC Sakai genome. The RPKM transcriptome, RPKM translatome, RCV, and coverage values represent mean values of the two biological replicates.
(DOCX)

S3 Table Properties of the novel genes. Annotated homologs in other strains/species were searched using blastp. Only the best hit is listed. The fourth column illustrates annotated homologs in other *E*. *coli* O157:H7 strains or duplications of annotated genes in EHEC Sakai. With bioinformatics methods the presence of a σ^70^ promoter, a ρ-independent terminator, a Shine-Dalgarno sequence, and selection pressure (k_A_/k_S_) were predicted or estimated. The last column gives the classification of the putative novel protein by the machine-learning algorithm trained with short annotated *E*. *coli* O157:H7 EDL933 genes.
(DOCX)

S4 Table Properties of the 250 short annotated genes. With bioinformatics methods the presence of a σ^70^ promoter, a ρ-independent terminator, a Shine-Dalgarno sequence and selection pressure (k_A_/k_S_) were predicted or estimated. The last column gives the classification of the short genes by the machine-learning algorithm.
(DOCX)

S5 Table Conservation of the novel genes. Summary of ORF conservation as represented in Fig 5.
(XLSX)

S6 Table Significant transcriptional and translational regulation in LB compared to BHI control of the novel genes and the short annotated genes. The mean value of the two biological replicates of transcriptome and translatome counts of the BHI control and the LB condition are shown. The log-fold change was calculated and differential gene expression was determined using *edgeR*. Transcriptional or translational changes are considered significant, when they show a *p*-value of ≤ 0.05 and a false discovery rate (FDR) of ≤ 0.1. Significant changes in LB compared to BHI control are highlighted in gray. Only genes with significant changes on transcriptional and/or translational level are listed.
(DOCX)

S7 Table Transcriptional and translational regulation at BHI COS compared to BHI control of the novel genes and the short annotated genes. The mean value of the two biological replicates of transcriptome and translatome counts of the BHI control and the stress condition COS are shown. The log-fold change was calculated and differential gene expression was determined using the software *edgeR*. Transcriptional or translational changes are considered significant, when they show a *p*-value of ≤ 0.05 and an FDR of ≤ 0.1. Significant changes in BHI COS compared to control are highlighted in gray. Only genes with significant changes on transcriptional and/or translational level are listed.
(DOCX)

S8 Table Summary of the Predict Protein results for the putative proteins encoded by the novel genes. The first columns show the AA composition, followed by predicted cellular localization, number of transmembrane helices, disulfide bonds and binding motives. Additionally, secondary structures, disordered regions and domains are predicted.
(XLSX)

S9 Table Summary of the Predict Protein results for the short annotated genes. The first columns show the AA composition, followed by predicted cellular localization, number of transmembrane helices, disulfide bonds and binding motives. Additionally, secondary structures, disordered regions and domains are predicted.
(XLSX)

S10 Table Classification into 'real' and 'pseudo' proteins by the machine-learning algorithm. The upper part of the table shows the results for the novel genes and the lower part for the scrambled sequences.
(XLSX)

S1 File Custom script used for reading frame determination in the sum signal of gene groups. (TXT)

S2 File Custom script used for detecting sequence conservation. (BASH)

S3 File Custom script used for extracting intergenic sequences—for comparative conservation analysis. (BASH)
