# Supplementary material for: Discovery of numerous novel small genes in the intergenic regions of the Escherichia coli O157:H7 Sakai genome
Source: PLoS One. 2017 Sep 13;12(9):e0184119. doi: 10.1371/journal.pone.0184119 (PMC5597208; doi:10.1371/journal.pone.0184119)
Supplement: S1 Fig — (A) RCV distribution at BHI control. (B) RCV distribution at BHI COS. (PPTX) [file pone.0184119.s001.pptx]

## Slide 1
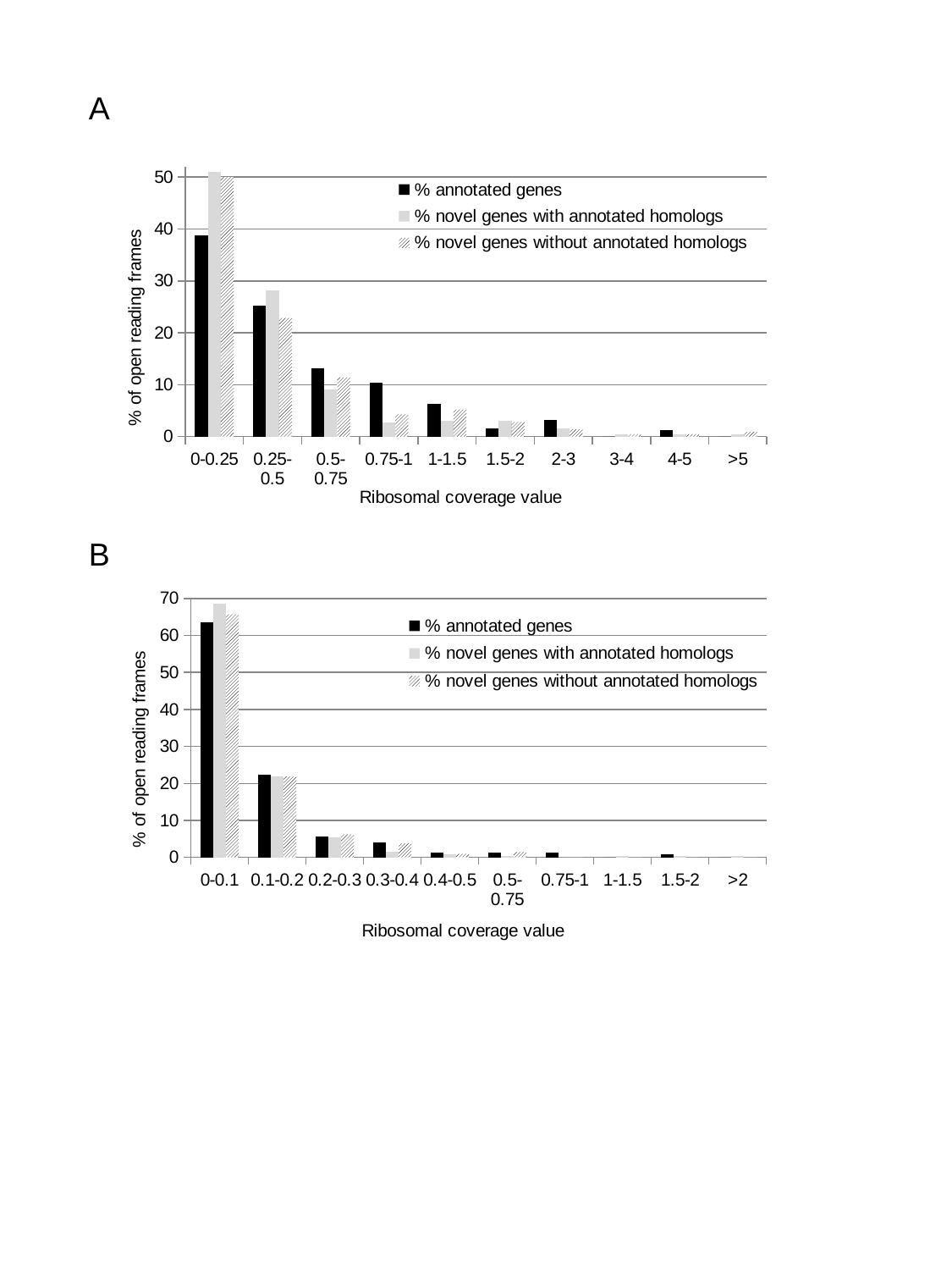

A
### Chart
| Category | % annotated genes | % novel genes with annotated homologs | % novel genes without annotated homologs |
|---|---|---|---|
| 0-0.25 | 38.8 | 50.98039215686274 | 50.0 |
| 0.25-0.5 | 25.2 | 28.235294117647058 | 22.857142857142858 |
| 0.5-0.75 | 13.2 | 9.019607843137255 | 11.428571428571429 |
| 0.75-1 | 10.4 | 2.7450980392156863 | 4.285714285714286 |
| 1-1.5 | 6.4 | 3.1372549019607843 | 5.238095238095238 |
| 1.5-2 | 1.6 | 3.1372549019607843 | 2.857142857142857 |
| 2-3 | 3.2 | 1.5686274509803921 | 1.4285714285714286 |
| 3-4 | 0.0 | 0.39215686274509803 | 0.47619047619047616 |
| 4-5 | 1.2 | 0.39215686274509803 | 0.47619047619047616 |
| >5 | 0.0 | 0.39215686274509803 | 0.9523809523809523 |B
### Chart
| Category | % annotated genes | % novel genes with annotated homologs | % novel genes without annotated homologs |
|---|---|---|---|
| 0-0.1 | 63.6 | 68.62745098039215 | 65.71428571428571 |
| 0.1-0.2 | 22.4 | 21.96078431372549 | 21.904761904761905 |
| 0.2-0.3 | 5.6 | 5.490196078431373 | 6.190476190476191 |
| 0.3-0.4 | 4.0 | 1.5686274509803921 | 3.8095238095238093 |
| 0.4-0.5 | 1.2 | 0.7843137254901961 | 0.9523809523809523 |
| 0.5-0.75 | 1.2 | 0.39215686274509803 | 1.4285714285714286 |
| 0.75-1 | 1.2 | 0.0 | 0.0 |
| 1-1.5 | 0.0 | 0.39215686274509803 | 0.0 |
| 1.5-2 | 0.8 | 0.39215686274509803 | 0.0 |
| >2 | 0.0 | 0.39215686274509803 | 0.0 |
