# Supplementary material for: Discovery of numerous novel small genes in the intergenic regions of the Escherichia coli O157:H7 Sakai genome
Source: PLoS One. 2017 Sep 13;12(9):e0184119. doi: 10.1371/journal.pone.0184119 (PMC5597208; doi:10.1371/journal.pone.0184119)
Supplement: S2 Fig — A similar process as used for Fig 5 was repeated on unannotated sequences upstream and downstream of the novel genes, but without removing sequences with stop codons. Many of the sequences had no tblastn hits (too short) and some others were excluded as more than one novel gene was situated between two annotated genes; one was excluded as abnormally long. Thus, 136 sequence remained for upstream and 122 for downstream. Most homologs have low similarity. The custom shell script used is provided in S3 File. (A) Analysis of the sequences upstream of the novel genes without annotated homologs. (B) Analysis of the sequences downstream of the novel genes without annotated homologs. The average evolutionary distance to the tblastn hits (of at least 80% similarity) of the novel proteins without annotated homologs (blastp) is 0.643. Average distance for their downstream sequences is 0.535, which is significantly lower (p = 0.0024, two-tailed t-test). Average in evolutionary distance for upstream regions is 0.596, not significantly different compared to distances for genes (p = 0.1421). The upstream region may be more conserved (e.g., due to regulatory sequences contained). (PPTX) [file pone.0184119.s002.pptx]

## Slide 1
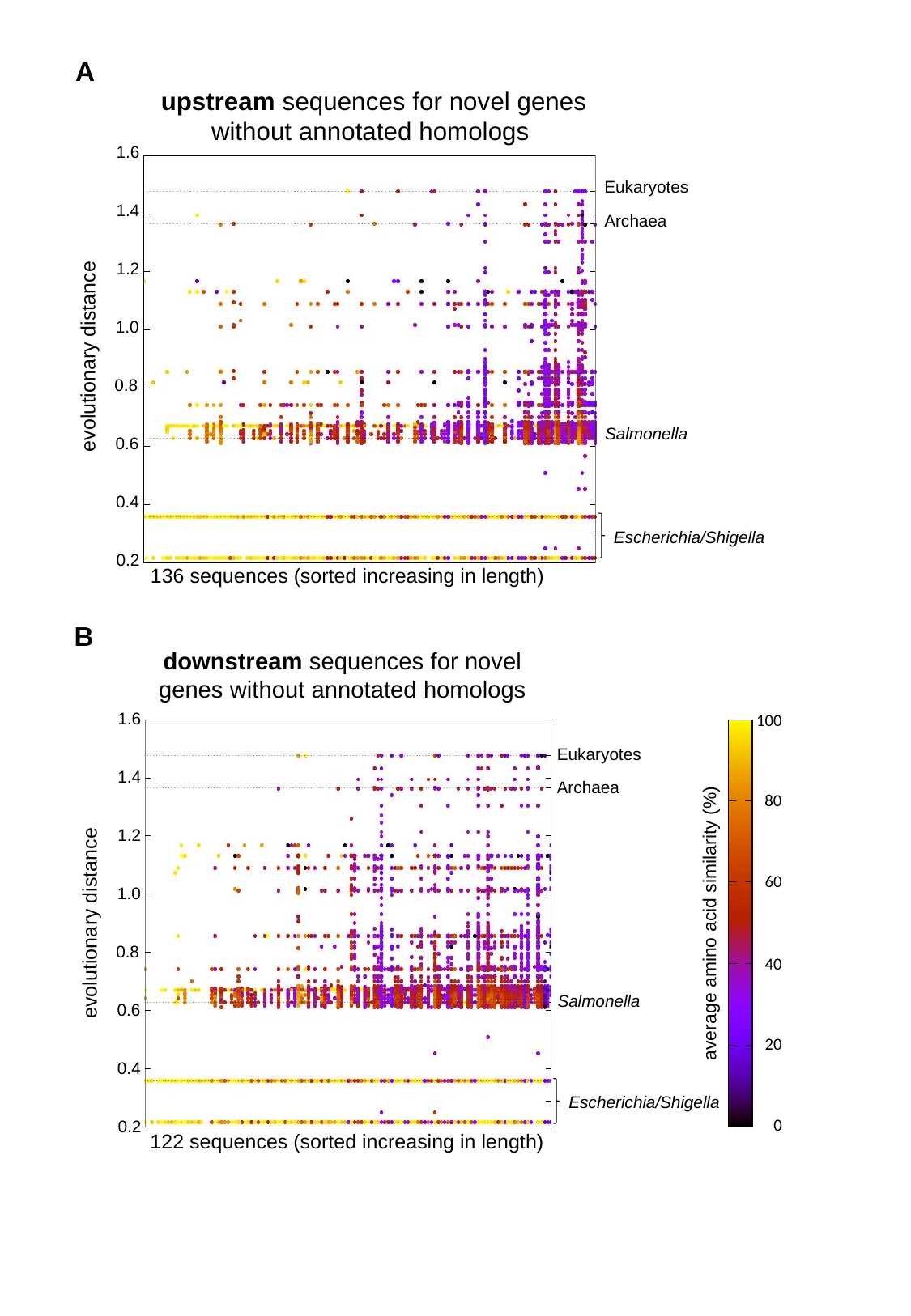

A
upstream sequences for novel genes without annotated homologs
1.6
Eukaryotes
1.4
Archaea
1.2
1.0
evolutionary distance
0.8
Salmonella
0.6
0.4
Escherichia/Shigella
0.2
136 sequences (sorted increasing in length)
B
downstream sequences for novel genes without annotated homologs
1.6
100
Eukaryotes
1.4
Archaea
80
1.2
60
1.0
evolutionary distance
average amino acid similarity (%)
0.8
40
Salmonella
0.6
20
0.4
Escherichia/Shigella
 0
0.2
122 sequences (sorted increasing in length)
