# Supplementary material for: Discovery of numerous novel small genes in the intergenic regions of the Escherichia coli O157:H7 Sakai genome
Source: PLoS One. 2017 Sep 13;12(9):e0184119. doi: 10.1371/journal.pone.0184119 (PMC5597208; doi:10.1371/journal.pone.0184119)
Supplement: S1 Table — The total number of reads, the number of reads mapping to the E. coli O157:H7 Sakai genome and the distribution of mapped reads to rRNA, tRNA and mRNA are shown. Only the reads mapping to mRNA were used for further analysis. Every library contains between 1.5–9.7 m. mRNA reads. (DOCX) [file pone.0184119.s003.docx]

| **Sample** | **Number of reads** | **Number of mapped reads** | **% rRNA** | **% tRNA** | **% mRNA** |
| --- | --- | --- | --- | --- | --- |
| LB, 37°C, transcriptome, replicate I | 23,759,146 | 15,393,472 | 77 | 10 | 13 |
| LB, 37°C, transcriptome, replicate II | 30,900,878 | 21,307864 | 79 | 8 | 13 |
| LB, 37°C, translatome, replicate I | 59,430,090 | 44,349,713 | 81 | 1 | 18 |
| LB, 37°C, translatome, replicate II | 47,331,178 | 31,181,934 | 82 | 1 | 17 |
| BHI, 37°C, transcriptome, replicate I | 94,103,438 | 23,476,896 | 60 | 17 | 23 |
| BHI, 37°C, transcriptome, replicate II | 19,483,193 | 9,676,571 | 65 | 13 | 22 |
| BHI, 37°C translatome, replicate I | 20,798,009 | 16,202,990 | 54 | 1 | 45 |
| BHI, 37°C, translatome, replicate II | 126,116,194 | 51,141,734 | 77 | 4 | 19 |
| BHI, 14°C, 4% NaCl, transcriptome, replicate I | 82,220,302 | 14,244,849 | 53 | 24 | 24 |
| BHI, 14°C, 4% NaCl, transcriptome, replicate II | 26,464,898 | 7,044,519 | 66 | 15 | 19 |
| BHI, 14°C, 4% NaCl translatome, replicate I | 172,487,790 | 55,940,877 | 92 | 1 | 7 |
| BHI, 14°C, 4% NaCl, translatome replicate II | 98,240,630 | 22,038,870 | 91 | 1 | 8 |
