# Supplementary material for: Discovery of numerous novel small genes in the intergenic regions of the Escherichia coli O157:H7 Sakai genome
Source: PLoS One. 2017 Sep 13;12(9):e0184119. doi: 10.1371/journal.pone.0184119 (PMC5597208; doi:10.1371/journal.pone.0184119)
Supplement: S2 Table — The novel genes are consecutively numbered after their appearance in the EHEC Sakai genome. The RPKM transcriptome, RPKM translatome, RCV, and coverage values represent mean values of the two biological replicates. (DOCX) [file pone.0184119.s004.docx]

| description | | | | LB, 37°C | | | | BHI control | | | | BHI stress | | | |
| --- | --- | --- | --- | --- | --- | --- | --- | --- | --- | --- | --- | --- | --- | --- | --- |
| gene name | start | stop | length [bp] | RPKM transcriptome | RPKM translatome | RCV | coverage | RPKM transcriptome | RPKM translatome | RCV | coverage | RPKM transcriptome | RPKM translatome | RCV | coverage |
| XECs001 | 5580 | 5681 | 102 | 48.2 | 20.0 | 0.42 | 0.67 | 60.4 | 10.1 | 0.19 | 0.77 | 126.4 | 9.9 | 0.07 | 0.72 |
| XECs002 | 25051 | 25146 | 96 | 8.7 | 14.2 | 1.67 | 0.72 | 61.5 | 14.9 | 0.24 | 0.69 | 40.2 | 1.1 | 0.03 | 0.41 |
| XECs003 | 39073 | 39267 | 195 | 25.2 | 32.9 | 1.31 | 0.70 | 11.8 | 2.3 | 0.20 | 0.46 | 5.3 | 0.3 | 0.04 | 0.09 |
| XECs004 | 39110 | 39277 | 168 | 16.0 | 22.6 | 1.41 | 0.64 | 11.2 | 2.4 | 0.22 | 0.36 | 6.2 | 0.3 | 0.04 | 0.11 |
| XECs005 | 64466 | 64588 | 123 | 73.7 | 22.8 | 0.31 | 0.65 | 66.3 | 5.4 | 0.08 | 0.72 | 22.3 | 0.5 | 0.02 | 0.20 |
| XECs006 | 93946 | 94089 | 144 | 105.7 | 37.3 | 0.35 | 0.59 | 275.9 | 29.1 | 0.11 | 0.77 | 163.5 | 1.2 | 0.01 | 0.38 |
| XECs007 | 94094 | 94228 | 135 | 254.1 | 429.9 | 1.88 | 0.75 | 539.9 | 35.9 | 0.07 | 0.97 | 911.0 | 17.5 | 0.02 | 0.65 |
| XECs008 | 135872 | 136003 | 132 | 194.2 | 842.2 | 4.40 | 0.88 | 314.8 | 584.1 | 1.78 | 0.83 | 676.0 | 123.5 | 0.19 | 0.90 |
| XECs009 | 148822 | 148923 | 102 | 20.9 | 87.9 | 8.24 | 0.69 | 34.9 | 6.4 | 0.20 | 0.54 | 30.5 | 3.2 | 0.11 | 0.38 |
| XECs010 | 152257 | 152370 | 114 | 22.4 | 12.5 | 0.65 | 0.64 | 39.5 | 8.8 | 0.21 | 0.71 | 27.9 | 1.2 | 0.12 | 0.42 |
| XECs011 | 188466 | 188609 | 144 | 65.7 | 51.3 | 0.77 | 0.59 | 59.1 | 10.7 | 0.18 | 0.56 | 95.2 | 1.6 | 0.02 | 0.30 |
| XECs012 | 193115 | 193306 | 192 | 1487.6 | 507.4 | 0.34 | 0.62 | 2863.1 | 253.7 | 0.08 | 0.80 | 2697.7 | 34.1 | 0.02 | 0.56 |
| XECs013 | 194119 | 194214 | 96 | 2007.6 | 871.6 | 0.43 | 1.00 | 2234.9 | 259.5 | 0.11 | 1.00 | 2513.5 | 27.8 | 0.01 | 0.94 |
| XECs014 | 194233 | 194367 | 135 | 20735.2 | 7542.2 | 0.36 | 1.00 | 15140.0 | 2954.7 | 0.21 | 0.98 | 21257.8 | 338.6 | 0.02 | 0.96 |
| XECs015 | 223357 | 223449 | 93 | 135.7 | 121.0 | 0.88 | 0.76 | 210.6 | 17.0 | 0.08 | 0.81 | 119.3 | 1.1 | 0.01 | 0.33 |
| XECs016 | 226647 | 226766 | 120 | 57.0 | 19.6 | 0.37 | 0.60 | 88.6 | 5.5 | 0.08 | 0.48 | 74.0 | 2.7 | 0.03 | 0.45 |
| XECs017 | 240840 | 240947 | 108 | 35.3 | 24.0 | 0.74 | 0.70 | 186.8 | 49.2 | 0.26 | 0.82 | 44.0 | 0.4 | 0.01 | 0.19 |
| XECs018 | 240883 | 241683 | 801 | 10.3 | 16.8 | 1.76 | 0.64 | 104.6 | 21.0 | 0.22 | 0.81 | 19.4 | 0.6 | 0.03 | 0.20 |
| XECs019 | 240890 | 241024 | 135 | 12.4 | 23.8 | 1.98 | 0.63 | 78.0 | 35.6 | 0.51 | 0.87 | 13.2 | 0.5 | 0.08 | 0.21 |
| XECs020 | 241037 | 241132 | 96 | 26.1 | 64.6 | 2.49 | 0.98 | 205.2 | 71.4 | 0.39 | 1.00 | 28.6 | 1.3 | 0.06 | 0.46 |
| XECs021 | 241139 | 241243 | 105 | 8.9 | 12.9 | 0.66 | 0.77 | 159.9 | 17.7 | 0.12 | 0.91 | 12.0 | 1.3 | 0.16 | 0.27 |
| XECs022 | 241146 | 241250 | 105 | 11.1 | 12.1 | 0.53 | 0.73 | 174.0 | 17.5 | 0.11 | 0.88 | 12.0 | 1.3 | 0.16 | 0.27 |
| XECs023 | 284624 | 284767 | 144 | 6.4 | 21.4 | 1.21 | 0.61 | 18.6 | 11.6 | 0.59 | 0.73 | 31.3 | 0.9 | 0.06 | 0.15 |
| XECs024 | 286096 | 286233 | 138 | 12.5 | 11.4 | 0.98 | 0.74 | 17.4 | 1.5 | 0.09 | 0.42 | 14.0 | 0.3 | 0.02 | 0.12 |
| XECs025 | 291082 | 291210 | 129 | 1.8 | 41.2 | 6.85 | 0.52 | 15.1 | 28.8 | 1.85 | 0.64 | 10.9 | 1.9 | 0.26 | 0.41 |
| XECs026 | 303307 | 303432 | 126 | 960.5 | 468.2 | 0.49 | 0.66 | 1871.3 | 191.5 | 0.09 | 0.73 | 1187.3 | 8.5 | 0.01 | 0.54 |
| XECs027 | 308434 | 308526 | 93 | 371.8 | 141.4 | 0.38 | 0.81 | 320.4 | 60.5 | 0.25 | 0.92 | 301.8 | 13.0 | 0.04 | 0.59 |
| XECs028 | 308435 | 308527 | 93 | 4.5 | 92.6 | 20.39 | 0.81 | 12.7 | 1.1 | 0.09 | 0.26 | 9.6 | 1.7 | 0.17 | 0.60 |
| XECs029 | 385213 | 385980 | 768 | 23.5 | 16.8 | 0.74 | 0.58 | 18.4 | 6.5 | 0.37 | 0.47 | 33.3 | 4.0 | 0.10 | 0.23 |
| XECs030 | 385377 | 385520 | 144 | 9.4 | 32.2 | 8.35 | 0.76 | 16.2 | 7.3 | 0.55 | 0.48 | 4.6 | 0.8 | 0.19 | 0.32 |
| XECs031 | 401948 | 402130 | 183 | 244.1 | 72.2 | 0.30 | 0.90 | 618.1 | 56.4 | 0.10 | 0.79 | 44.9 | 7.8 | 0.26 | 0.56 |
| XECs032 | 402072 | 402251 | 180 | 134.0 | 69.8 | 0.54 | 0.91 | 486.8 | 70.8 | 0.15 | 0.95 | 28.0 | 7.2 | 0.29 | 0.36 |
| XECs033 | 402139 | 402345 | 207 | 35.0 | 53.4 | 1.53 | 0.87 | 208.9 | 64.5 | 0.35 | 0.95 | 19.0 | 0.3 | 0.02 | 0.13 |
| XECs034 | 402291 | 402386 | 96 | 16.4 | 29.9 | 2.10 | 0.80 | 188.3 | 51.0 | 0.37 | 0.79 | 17.0 | 0.9 | 0.06 | 0.16 |
| XECs035 | 435484 | 435606 | 123 | 31.7 | 16.8 | 0.53 | 0.58 | 88.3 | 9.5 | 0.11 | 0.68 | 101.2 | 11.6 | 0.11 | 0.57 |
| XECs036 | 435516 | 435680 | 165 | 29.2 | 17.9 | 0.68 | 0.66 | 31.6 | 8.3 | 0.31 | 0.61 | 24.7 | 1.7 | 0.07 | 0.39 |
| XECs037 | 472088 | 472201 | 114 | 50.5 | 177.4 | 3.52 | 0.58 | 13.1 | 11.3 | 0.99 | 0.62 | 7.2 | 1.5 | 0.22 | 0.32 |
| XECs038 | 481393 | 481491 | 99 | 21.6 | 131.4 | 7.00 | 0.49 | 66.7 | 35.1 | 0.51 | 0.62 | 20.9 | 2.3 | 0.11 | 0.34 |
| XECs039 | 481436 | 481534 | 99 | 31.9 | 137.3 | 4.57 | 0.64 | 64.5 | 35.4 | 0.54 | 0.68 | 19.4 | 3.3 | 0.17 | 0.54 |
| XECs040 | 496201 | 496344 | 144 | 144.0 | 766.1 | 5.10 | 0.95 | 117.1 | 153.5 | 1.33 | 0.86 | 49.9 | 9.2 | 0.23 | 0.49 |
| XECs041 | 496268 | 496366 | 99 | 99.1 | 60.1 | 0.62 | 0.88 | 111.3 | 32.8 | 0.34 | 0.72 | 54.6 | 3.2 | 0.06 | 0.25 |
| XECs042 | 499801 | 499914 | 114 | 9.0 | 24.9 | 2.76 | 0.74 | 9.1 | 11.9 | 1.30 | 0.71 | 7.8 | 2.2 | 0.17 | 0.21 |
| XECs043 | 503155 | 503316 | 162 | 2.6 | 5.1 | 1.94 | 0.35 | 16.1 | 19.8 | 1.54 | 0.59 | 1.8 | 0.3 | 0.18 | 0.08 |
| XECs044 | 507354 | 507563 | 210 | 149.0 | 83.7 | 0.56 | 0.72 | 334.1 | 31.7 | 0.09 | 0.73 | 256.7 | 3.3 | 0.02 | 0.49 |
| XECs045 | 507510 | 507602 | 93 | 36.0 | 103.2 | 2.84 | 0.74 | 146.5 | 40.1 | 0.28 | 0.80 | 120.2 | 5.2 | 0.05 | 0.71 |
| XECs046 | 520776 | 521036 | 261 | 77.9 | 147.3 | 1.91 | 0.78 | 104.4 | 31.6 | 0.31 | 0.84 | 54.2 | 4.7 | 0.10 | 0.59 |
| XECs047 | 521053 | 521145 | 93 | 2453.8 | 1292.3 | 0.54 | 0.99 | 3156.2 | 225.8 | 0.08 | 0.99 | 2606.7 | 68.0 | 0.03 | 0.94 |
| XECs048 | 523342 | 523485 | 144 | 849.4 | 373.5 | 0.44 | 1.00 | 1142.6 | 98.1 | 0.09 | 0.96 | 1393.3 | 36.3 | 0.03 | 0.93 |
| XECs049 | 523349 | 523471 | 123 | 957.4 | 385.6 | 0.40 | 1.00 | 1306.5 | 96.1 | 0.07 | 0.95 | 1591.4 | 41.6 | 0.03 | 0.91 |
| XECs050 | 541175 | 541318 | 144 | 231.1 | 114.7 | 0.50 | 0.85 | 208.5 | 58.9 | 0.33 | 0.72 | 123.5 | 5.9 | 0.05 | 0.40 |
| XECs051 | 557871 | 557987 | 117 | 80.1 | 41.6 | 0.54 | 0.76 | 79.6 | 12.1 | 0.15 | 0.75 | 127.8 | 7.2 | 0.05 | 0.69 |
| XECs052 | 572265 | 572390 | 126 | 15.5 | 13.2 | 1.00 | 0.57 | 11.4 | 4.9 | 0.58 | 0.76 | 12.9 | 1.2 | 0.10 | 0.26 |
| XECs053 | 608044 | 608214 | 171 | 13.3 | 17.9 | 1.35 | 0.55 | 23.8 | 4.9 | 0.20 | 0.62 | 23.4 | 2.6 | 0.18 | 0.49 |
| XECs054 | 631719 | 631916 | 198 | 8.2 | 11.4 | 1.37 | 0.58 | 6.1 | 8.2 | 1.29 | 0.83 | 5.2 | 0.9 | 0.15 | 0.12 |
| XECs055 | 649039 | 649176 | 138 | 169.0 | 464.5 | 2.86 | 0.97 | 129.9 | 150.7 | 1.20 | 0.96 | 82.1 | 46.9 | 0.71 | 0.97 |
| XECs056 | 657201 | 657368 | 168 | 22.4 | 53.3 | 2.41 | 0.55 | 98.7 | 46.8 | 0.53 | 0.59 | 22.9 | 2.6 | 0.12 | 0.26 |
| XECs057 | 657224 | 657337 | 114 | 25.7 | 55.0 | 2.21 | 0.59 | 101.2 | 26.7 | 0.30 | 0.60 | 27.3 | 3.9 | 0.14 | 0.39 |
| XECs058 | 690459 | 690662 | 204 | 103.3 | 83.7 | 0.81 | 0.67 | 486.8 | 75.9 | 0.16 | 0.73 | 231.7 | 2.2 | 0.01 | 0.36 |
| XECs059 | 713487 | 713636 | 150 | 53.5 | 99.5 | 1.95 | 0.73 | 31.4 | 29.6 | 0.96 | 0.60 | 91.5 | 5.6 | 0.06 | 0.70 |
| XECs060 | 720171 | 720317 | 147 | 110.2 | 298.8 | 2.71 | 0.94 | 120.4 | 131.7 | 1.06 | 0.89 | 159.1 | 35.9 | 0.24 | 0.69 |
| XECs061 | 720232 | 720354 | 123 | 134.4 | 254.8 | 1.80 | 0.87 | 90.4 | 27.9 | 0.43 | 0.74 | 256.0 | 3.5 | 0.05 | 0.55 |
| XECs062 | 740203 | 740307 | 105 | 428.4 | 380.8 | 0.89 | 0.88 | 173.2 | 110.2 | 0.63 | 0.98 | 136.7 | 13.5 | 0.20 | 0.87 |
| XECs063 | 750505 | 750618 | 114 | 6.5 | 32.8 | 2.39 | 0.64 | 71.9 | 13.3 | 0.19 | 0.72 | 122.0 | 9.8 | 0.11 | 0.70 |
| XECs064 | 756188 | 756283 | 96 | 382.1 | 479.3 | 1.22 | 0.82 | 383.5 | 98.4 | 0.25 | 0.88 | 271.4 | 1.5 | 0.01 | 0.28 |
| XECs065 | 756819 | 756959 | 141 | 60.9 | 28.3 | 0.46 | 0.78 | 30.8 | 4.2 | 0.14 | 0.68 | 18.9 | 1.6 | 0.08 | 0.41 |
| XECs066 | 783609 | 783746 | 138 | 577.6 | 774.7 | 1.34 | 0.78 | 145.3 | 51.9 | 0.50 | 0.62 | 45.2 | 5.4 | 0.15 | 0.50 |
| XECs067 | 790666 | 790899 | 234 | 510.7 | 238.0 | 0.49 | 0.84 | 659.6 | 87.8 | 0.13 | 0.82 | 1259.6 | 41.6 | 0.04 | 0.85 |
| XECs068 | 790769 | 790879 | 111 | 72.0 | 63.5 | 0.94 | 0.86 | 197.2 | 36.0 | 0.21 | 0.89 | 526.7 | 23.8 | 0.08 | 0.89 |
| XECs069 | 799046 | 799150 | 105 | 328.4 | 416.8 | 1.24 | 0.80 | 52.6 | 36.5 | 1.04 | 0.70 | 13.4 | 1.1 | 0.09 | 0.42 |
| XECs070 | 799107 | 799208 | 102 | 577.8 | 855.6 | 1.39 | 1.00 | 55.0 | 104.4 | 2.94 | 0.99 | 5.8 | 0.5 | 0.08 | 0.25 |
| XECs071 | 831178 | 831288 | 111 | 164.8 | 159.4 | 1.00 | 0.82 | 90.9 | 21.5 | 0.23 | 0.67 | 332.8 | 44.7 | 0.14 | 0.67 |
| XECs072 | 831189 | 831284 | 96 | 158.2 | 183.1 | 1.20 | 0.89 | 95.0 | 24.5 | 0.25 | 0.70 | 336.2 | 51.5 | 0.16 | 0.75 |
| XECs073 | 831200 | 831319 | 120 | 199.7 | 150.0 | 0.79 | 0.95 | 116.3 | 20.6 | 0.17 | 0.77 | 415.2 | 43.6 | 0.10 | 0.85 |
| XECs074 | 831692 | 831793 | 102 | 72.7 | 47.3 | 0.66 | 0.99 | 51.6 | 6.3 | 0.12 | 0.68 | 93.7 | 12.9 | 0.13 | 0.79 |
| XECs075 | 855717 | 855809 | 93 | 97.8 | 167.1 | 1.71 | 0.44 | 292.9 | 230.8 | 0.75 | 0.76 | 176.5 | 6.5 | 0.19 | 0.42 |
| XECs076 | 882228 | 882413 | 186 | 9.7 | 19.4 | 1.97 | 0.66 | 25.6 | 5.2 | 0.21 | 0.67 | 24.7 | 3.5 | 0.14 | 0.33 |
| XECs077 | 942222 | 942329 | 108 | 961.8 | 1267.3 | 1.43 | 1.00 | 460.6 | 278.9 | 0.57 | 1.00 | 74.1 | 11.7 | 0.19 | 0.81 |
| XECs078 | 961855 | 961986 | 132 | 53.8 | 31.2 | 0.61 | 0.74 | 44.4 | 9.8 | 0.23 | 0.81 | 169.9 | 3.5 | 0.01 | 0.26 |
| XECs079 | 976913 | 977044 | 132 | 8.8 | 44.7 | 6.84 | 0.66 | 32.2 | 10.4 | 0.33 | 0.64 | 25.2 | 6.8 | 0.29 | 0.51 |
| XECs080 | 976954 | 977178 | 225 | 9.1 | 33.1 | 3.59 | 0.69 | 37.7 | 9.7 | 0.26 | 0.67 | 50.6 | 5.7 | 0.16 | 0.64 |
| XECs081 | 977117 | 977221 | 105 | 23.4 | 22.1 | 0.92 | 0.70 | 51.4 | 10.5 | 0.25 | 0.61 | 117.0 | 5.3 | 0.06 | 0.83 |
| XECs082 | 977178 | 977333 | 156 | 36.3 | 673.9 | 18.03 | 0.95 | 207.6 | 523.7 | 3.95 | 0.89 | 241.3 | 107.7 | 1.38 | 0.96 |
| XECs083 | 988556 | 988726 | 171 | 19.3 | 19.3 | 1.32 | 0.75 | 22.7 | 6.3 | 0.31 | 0.75 | 29.9 | 4.3 | 0.14 | 0.56 |
| XECs084 | 1013202 | 1013330 | 129 | 173.5 | 270.9 | 1.70 | 0.74 | 193.6 | 114.1 | 0.70 | 0.76 | 154.7 | 18.0 | 0.40 | 0.60 |
| XECs085 | 1015250 | 1015351 | 102 | 562.6 | 157.1 | 0.28 | 0.94 | 347.1 | 140.3 | 0.40 | 0.99 | 367.3 | 2.8 | 0.01 | 0.50 |
| XECs086 | 1036840 | 1036935 | 96 | 39.2 | 12.5 | 0.33 | 0.55 | 42.7 | 6.4 | 0.15 | 0.70 | 37.8 | 3.0 | 0.08 | 0.44 |
| XECs087 | 1046003 | 1046095 | 93 | 12.0 | 21.0 | 4.08 | 0.62 | 10.1 | 3.4 | 0.34 | 0.39 | 5.6 | 1.3 | 0.18 | 0.15 |
| XECs088 | 1046126 | 1046410 | 285 | 17.6 | 36.8 | 2.07 | 0.64 | 57.4 | 6.4 | 0.16 | 0.53 | 22.6 | 2.2 | 0.12 | 0.26 |
| XECs089 | 1053586 | 1053696 | 111 | 62.3 | 60.8 | 1.05 | 0.62 | 78.8 | 28.2 | 0.36 | 0.67 | 153.7 | 4.7 | 0.04 | 0.63 |
| XECs090 | 1053770 | 1053886 | 117 | 71.5 | 60.4 | 0.85 | 0.69 | 71.2 | 5.0 | 0.07 | 0.69 | 126.7 | 0.7 | 0.01 | 0.11 |
| XECs091 | 1057652 | 1057804 | 153 | 30.4 | 64.8 | 2.24 | 0.90 | 41.8 | 32.2 | 1.04 | 0.86 | 33.4 | 25.4 | 0.74 | 0.60 |
| XECs092 | 1080678 | 1080803 | 126 | 321.9 | 128.1 | 0.40 | 1.00 | 204.8 | 60.0 | 0.27 | 0.90 | 198.8 | 13.3 | 0.07 | 0.85 |
| XECs093 | 1087204 | 1087341 | 138 | 184.3 | 529.4 | 2.89 | 0.80 | 122.5 | 205.3 | 1.78 | 0.67 | 121.2 | 32.8 | 0.35 | 0.61 |
| XECs094 | 1093345 | 1093452 | 108 | 526.7 | 733.6 | 1.41 | 0.88 | 712.6 | 148.7 | 0.22 | 0.92 | 789.9 | 8.4 | 0.01 | 0.59 |
| XECs095 | 1149544 | 1149732 | 189 | 6760.8 | 9788.7 | 1.44 | 0.79 | 4059.7 | 3560.1 | 0.95 | 0.82 | 4060.1 | 160.9 | 0.05 | 0.81 |
| XECs096 | 1149708 | 1149869 | 162 | 105.5 | 37.4 | 0.36 | 0.64 | 67.6 | 12.0 | 0.17 | 0.69 | 109.8 | 14.1 | 0.13 | 0.78 |
| XECs097 | 1169201 | 1169362 | 162 | 15.2 | 13.3 | 0.89 | 0.57 | 10.7 | 4.8 | 0.42 | 0.51 | 10.1 | 0.4 | 0.04 | 0.21 |
| XECs098 | 1174303 | 1174512 | 210 | 16.8 | 37.6 | 2.24 | 0.60 | 149.9 | 24.8 | 0.15 | 0.67 | 20.5 | 2.6 | 0.16 | 0.42 |
| XECs099 | 1178680 | 1178832 | 153 | 31.9 | 14.1 | 0.47 | 0.62 | 34.9 | 11.0 | 0.36 | 0.69 | 7.3 | 0.6 | 0.12 | 0.21 |
| XECs100 | 1179839 | 1180039 | 201 | 83.9 | 63.2 | 0.81 | 0.62 | 83.6 | 6.0 | 0.08 | 0.51 | 29.1 | 0.7 | 0.03 | 0.19 |
| XECs101 | 1180026 | 1180217 | 192 | 159.5 | 55.8 | 0.37 | 0.74 | 184.7 | 22.8 | 0.12 | 0.84 | 108.5 | 1.4 | 0.01 | 0.45 |
| XECs102 | 1180030 | 1180158 | 129 | 138.4 | 36.8 | 0.26 | 0.61 | 100.1 | 5.2 | 0.05 | 0.65 | 111.6 | 1.1 | 0.01 | 0.41 |
| XECs103 | 1183291 | 1183467 | 177 | 88.2 | 86.1 | 0.96 | 0.71 | 113.6 | 34.2 | 0.32 | 0.79 | 36.9 | 0.8 | 0.02 | 0.16 |
| XECs104 | 1183367 | 1183678 | 312 | 221.1 | 352.6 | 1.59 | 0.93 | 187.9 | 208.2 | 1.19 | 0.95 | 47.8 | 1.4 | 0.03 | 0.43 |
| XECs105 | 1183603 | 1183695 | 93 | 491.4 | 904.1 | 1.81 | 1.00 | 373.9 | 584.3 | 1.51 | 0.99 | 81.2 | 3.0 | 0.04 | 0.86 |
| XECs106 | 1184893 | 1184994 | 102 | 5.9 | 34.5 | 6.08 | 0.72 | 60.1 | 17.2 | 0.30 | 0.75 | 15.3 | 1.6 | 0.15 | 0.27 |
| XECs107 | 1186394 | 1186486 | 93 | 52.5 | 35.2 | 0.68 | 0.74 | 44.4 | 4.0 | 0.12 | 0.48 | 19.9 | 0.7 | 0.02 | 0.16 |
| XECs108 | 1186569 | 1186724 | 156 | 226.3 | 73.6 | 0.33 | 0.66 | 75.2 | 18.6 | 0.25 | 0.59 | 151.6 | 9.5 | 0.06 | 0.37 |
| XECs109 | 1210036 | 1210149 | 114 | 12.2 | 25.2 | 1.95 | 0.54 | 18.4 | 23.1 | 1.79 | 0.87 | 12.3 | 1.4 | 0.10 | 0.43 |
| XECs110 | 1210044 | 1210136 | 93 | 14.9 | 30.9 | 1.95 | 0.59 | 15.3 | 26.1 | 2.32 | 0.92 | 15.1 | 1.7 | 0.10 | 0.50 |
| XECs111 | 1228889 | 1229020 | 132 | 4799.9 | 5680.8 | 1.23 | 1.00 | 3436.3 | 587.8 | 0.16 | 0.98 | 497.8 | 34.6 | 0.12 | 0.95 |
| XECs112 | 1229624 | 1229734 | 111 | 192.3 | 83.7 | 0.45 | 0.66 | 1468.1 | 60.4 | 0.06 | 0.75 | 682.5 | 34.4 | 0.34 | 0.65 |
| XECs113 | 1230459 | 1230590 | 132 | 283.3 | 307.3 | 1.09 | 0.94 | 224.7 | 524.0 | 2.35 | 0.98 | 121.8 | 13.5 | 0.12 | 0.64 |
| XECs114 | 1255538 | 1255645 | 108 | 452.2 | 134.6 | 0.30 | 0.74 | 807.0 | 88.0 | 0.09 | 0.68 | 234.5 | 2.6 | 0.01 | 0.57 |
| XECs115 | 1266493 | 1266585 | 93 | 46.0 | 45.2 | 1.17 | 0.58 | 131.0 | 9.3 | 0.11 | 0.48 | 113.9 | 12.9 | 0.12 | 0.70 |
| XECs116 | 1268318 | 1268440 | 123 | 416.4 | 172.6 | 0.41 | 0.89 | 290.5 | 9.1 | 0.04 | 0.63 | 124.4 | 4.1 | 0.04 | 0.69 |
| XECs117 | 1309075 | 1309245 | 171 | 7.3 | 18.1 | 2.52 | 0.61 | 12.4 | 10.4 | 0.90 | 0.58 | 17.8 | 1.4 | 0.07 | 0.07 |
| XECs118 | 1390321 | 1390437 | 117 | 128.3 | 110.8 | 0.87 | 0.76 | 229.9 | 99.2 | 0.36 | 0.68 | 115.8 | 1.4 | 0.01 | 0.25 |
| XECs119 | 1396044 | 1396169 | 126 | 48.6 | 49.8 | 1.02 | 0.77 | 66.1 | 36.6 | 0.55 | 0.83 | 44.1 | 2.3 | 0.08 | 0.47 |
| XECs120 | 1396051 | 1396185 | 135 | 57.1 | 49.2 | 0.88 | 0.81 | 60.6 | 35.6 | 0.57 | 0.84 | 43.3 | 2.0 | 0.07 | 0.42 |
| XECs121 | 1396908 | 1397045 | 138 | 7711.4 | 3596.2 | 0.46 | 1.00 | 5647.1 | 485.2 | 0.08 | 1.00 | 2703.7 | 42.8 | 0.02 | 0.98 |
| XECs122 | 1396976 | 1397086 | 111 | 8747.0 | 4310.1 | 0.49 | 0.99 | 6659.5 | 572.3 | 0.08 | 1.00 | 3714.4 | 56.5 | 0.02 | 1.00 |
| XECs123 | 1399649 | 1399786 | 138 | 16.1 | 78.9 | 4.78 | 0.88 | 217.6 | 79.6 | 0.37 | 0.95 | 238.0 | 2.9 | 0.01 | 0.67 |
| XECs124 | 1404328 | 1404453 | 126 | 51.9 | 38.6 | 0.77 | 0.93 | 84.9 | 15.6 | 0.18 | 0.92 | 58.8 | 5.0 | 0.07 | 0.46 |
| XECs125 | 1404870 | 1405196 | 327 | 31.0 | 26.1 | 0.94 | 0.72 | 65.6 | 17.5 | 0.26 | 0.90 | 131.2 | 4.1 | 0.03 | 0.73 |
| XECs126 | 1404992 | 1405294 | 303 | 31.5 | 28.4 | 1.02 | 0.78 | 68.5 | 15.0 | 0.22 | 0.82 | 129.8 | 2.5 | 0.02 | 0.57 |
| XECs127 | 1417358 | 1417465 | 108 | 31.4 | 25.7 | 0.80 | 0.50 | 25.1 | 15.4 | 0.54 | 0.67 | 23.3 | 0.6 | 0.03 | 0.25 |
| XECs128 | 1417455 | 1417775 | 321 | 61.9 | 58.3 | 0.94 | 0.83 | 58.1 | 45.0 | 0.73 | 0.81 | 26.3 | 6.1 | 0.23 | 0.63 |
| XECs129 | 1417525 | 1417698 | 174 | 25.4 | 70.9 | 2.98 | 0.87 | 27.6 | 38.8 | 1.26 | 0.88 | 10.2 | 4.0 | 0.39 | 0.67 |
| XECs130 | 1417820 | 1417963 | 144 | 125.4 | 94.7 | 0.77 | 0.95 | 170.6 | 16.1 | 0.10 | 0.97 | 103.0 | 2.7 | 0.02 | 0.24 |
| XECs131 | 1418045 | 1418149 | 105 | 32.8 | 39.4 | 1.33 | 0.77 | 73.8 | 5.9 | 0.08 | 0.61 | 22.6 | 1.5 | 0.08 | 0.47 |
| XECs132 | 1422400 | 1422543 | 144 | 116.9 | 96.1 | 0.76 | 0.58 | 47.8 | 33.5 | 0.95 | 0.48 | 64.4 | 10.4 | 0.17 | 0.41 |
| XECs133 | 1424859 | 1425050 | 192 | 25.4 | 52.1 | 2.04 | 0.76 | 52.5 | 14.5 | 0.29 | 0.65 | 10.0 | 1.2 | 0.12 | 0.15 |
| XECs134 | 1425376 | 1425594 | 219 | 6.6 | 19.9 | 3.05 | 0.68 | 9.9 | 2.0 | 0.20 | 0.34 | 5.1 | 0.2 | 0.05 | 0.05 |
| XECs135 | 1429421 | 1429558 | 138 | 11.1 | 6.9 | 1.01 | 0.43 | 36.5 | 16.9 | 0.51 | 0.86 | 9.7 | 0.8 | 0.07 | 0.14 |
| XECs136 | 1436583 | 1436693 | 111 | 13.8 | 62.8 | 5.57 | 0.62 | 35.4 | 12.0 | 0.33 | 0.68 | 6.0 | 0.5 | 0.09 | 0.12 |
| XECs137 | 1440648 | 1440749 | 102 | 445.2 | 1643.6 | 3.92 | 0.93 | 469.7 | 72.0 | 0.16 | 0.85 | 188.9 | 2.6 | 0.01 | 0.40 |
| XECs138 | 1440718 | 1440870 | 153 | 212.2 | 1340.1 | 6.37 | 1.00 | 419.2 | 221.5 | 0.53 | 1.00 | 183.6 | 5.1 | 0.03 | 0.57 |
| XECs139 | 1442058 | 1442225 | 168 | 37.3 | 12.3 | 0.33 | 0.53 | 78.9 | 2.6 | 0.04 | 0.37 | 7.9 | 0.4 | 0.05 | 0.14 |
| XECs140 | 1474524 | 1474655 | 132 | 38.7 | 49.8 | 1.30 | 0.92 | 53.1 | 15.9 | 0.34 | 0.72 | 25.3 | 4.8 | 0.35 | 0.69 |
| XECs141 | 1493706 | 1493870 | 165 | 154.0 | 134.0 | 0.90 | 0.90 | 3.5 | 10.1 | 6.76 | 0.78 | 0.9 | 0.5 | 0.59 | 0.30 |
| XECs142 | 1505005 | 1505145 | 141 | 14.2 | 13.4 | 1.45 | 0.58 | 25.9 | 5.9 | 0.21 | 0.54 | 23.1 | 3.5 | 0.15 | 0.44 |
| XECs143 | 1516843 | 1516968 | 126 | 154.6 | 53.0 | 0.34 | 0.84 | 247.4 | 112.4 | 0.38 | 0.93 | 366.3 | 2.6 | 0.01 | 0.73 |
| XECs144 | 1539498 | 1539617 | 120 | 29.8 | 16.0 | 0.55 | 0.53 | 14.7 | 1.3 | 0.10 | 0.46 | 53.6 | 1.2 | 0.05 | 0.39 |
| XECs145 | 1550761 | 1550922 | 162 | 79.1 | 346.1 | 4.35 | 0.99 | 121.3 | 150.1 | 1.14 | 0.84 | 73.3 | 1.1 | 0.01 | 0.37 |
| XECs146 | 1554306 | 1554482 | 177 | 47.5 | 19.9 | 0.41 | 0.62 | 225.9 | 12.9 | 0.06 | 0.60 | 151.9 | 3.7 | 0.02 | 0.52 |
| XECs147 | 1561115 | 1561276 | 162 | 37.9 | 19.2 | 0.52 | 0.60 | 51.7 | 5.8 | 0.11 | 0.65 | 22.0 | 1.1 | 0.06 | 0.21 |
| XECs148 | 1561141 | 1561245 | 105 | 50.0 | 17.9 | 0.37 | 0.72 | 72.5 | 6.1 | 0.09 | 0.86 | 27.5 | 0.8 | 0.04 | 0.17 |
| XECs149 | 1576747 | 1576884 | 138 | 23.5 | 14.3 | 0.59 | 0.61 | 34.1 | 9.1 | 0.26 | 0.72 | 23.6 | 3.3 | 0.15 | 0.18 |
| XECs150 | 1587711 | 1587815 | 105 | 30.6 | 19.7 | 0.71 | 0.69 | 65.3 | 3.2 | 0.06 | 0.69 | 7.8 | 0.2 | 0.03 | 0.11 |
| XECs151 | 1596342 | 1596530 | 189 | 29.3 | 28.3 | 1.04 | 0.85 | 30.6 | 11.7 | 0.36 | 0.91 | 11.0 | 0.9 | 0.09 | 0.28 |
| XECs152 | 1596460 | 1596585 | 126 | 27.3 | 20.9 | 0.94 | 0.73 | 26.5 | 11.6 | 0.44 | 0.60 | 15.3 | 2.9 | 0.18 | 0.29 |
| XECs153 | 1602366 | 1602473 | 108 | 29.3 | 11.7 | 0.42 | 0.64 | 33.8 | 3.6 | 0.10 | 0.60 | 9.6 | 0.2 | 0.04 | 0.08 |
| XECs154 | 1628272 | 1628388 | 117 | 5.2 | 10.6 | 2.20 | 0.64 | 47.4 | 9.9 | 0.26 | 0.74 | 1.3 | 0.0 | 0.00 | 0.00 |
| XECs155 | 1659312 | 1659443 | 132 | 30.6 | 38.4 | 1.31 | 0.76 | 165.8 | 38.6 | 0.22 | 0.85 | 86.3 | 3.7 | 0.12 | 0.63 |
| XECs156 | 1668452 | 1668562 | 111 | 51.9 | 131.2 | 2.62 | 0.77 | 62.1 | 6.9 | 0.11 | 0.58 | 26.0 | 1.7 | 0.22 | 0.25 |
| XECs157 | 1685334 | 1685501 | 168 | 14.3 | 23.3 | 1.72 | 0.64 | 48.6 | 3.5 | 0.07 | 0.63 | 45.4 | 3.2 | 0.07 | 0.45 |
| XECs158 | 1704965 | 1705066 | 102 | 66.5 | 36.6 | 0.56 | 0.81 | 45.2 | 5.9 | 0.42 | 0.75 | 16.7 | 2.1 | 0.16 | 0.69 |
| XECs159 | 1704990 | 1705085 | 96 | 41.6 | 37.0 | 0.90 | 0.86 | 48.0 | 6.2 | 0.42 | 0.79 | 14.6 | 2.0 | 0.16 | 0.61 |
| XECs160 | 1707771 | 1707887 | 117 | 568.5 | 199.8 | 0.35 | 0.98 | 461.0 | 66.1 | 0.14 | 0.95 | 1144.6 | 45.3 | 0.04 | 0.96 |
| XECs161 | 1714965 | 1715171 | 207 | 29.3 | 27.6 | 0.96 | 0.67 | 25.1 | 5.5 | 0.22 | 0.61 | 71.3 | 1.6 | 0.02 | 0.43 |
| XECs162 | 1717718 | 1717852 | 135 | 19.9 | 55.1 | 2.78 | 0.60 | 21.7 | 5.7 | 0.33 | 0.57 | 111.9 | 2.6 | 0.03 | 0.48 |
| XECs163 | 1732157 | 1732537 | 381 | 87.8 | 151.0 | 1.72 | 0.70 | 232.4 | 96.5 | 0.38 | 0.70 | 100.5 | 2.1 | 0.02 | 0.48 |
| XECs164 | 1742550 | 1742657 | 108 | 34.0 | 42.0 | 1.34 | 0.68 | 39.7 | 51.5 | 1.15 | 0.86 | 26.0 | 0.0 | 0.00 | 0.00 |
| XECs165 | 1757510 | 1757623 | 114 | 955.1 | 496.0 | 0.52 | 0.86 | 62.4 | 26.8 | 0.43 | 0.63 | 3.9 | 1.2 | 0.30 | 0.25 |
| XECs166 | 1768946 | 1769215 | 270 | 166.5 | 246.9 | 1.50 | 0.61 | 533.5 | 34.0 | 0.07 | 0.80 | 637.2 | 8.4 | 0.02 | 0.39 |
| XECs167 | 1768993 | 1769091 | 99 | 12.2 | 67.8 | 5.46 | 0.57 | 60.9 | 25.7 | 0.46 | 0.80 | 26.1 | 6.0 | 0.20 | 0.36 |
| XECs168 | 1774126 | 1774281 | 156 | 33.9 | 29.3 | 0.88 | 0.61 | 86.1 | 55.5 | 0.90 | 0.57 | 35.6 | 1.5 | 0.04 | 0.22 |
| XECs169 | 1774140 | 1774238 | 99 | 47.8 | 39.0 | 0.82 | 0.54 | 101.7 | 80.1 | 1.12 | 0.47 | 44.2 | 2.3 | 0.06 | 0.18 |
| XECs170 | 1774538 | 1774705 | 168 | 162.0 | 719.6 | 4.30 | 0.82 | 71.5 | 15.7 | 0.23 | 0.73 | 32.6 | 1.8 | 0.06 | 0.45 |
| XECs171 | 1779408 | 1779509 | 102 | 19.2 | 20.9 | 1.32 | 0.63 | 10.7 | 4.4 | 0.55 | 0.64 | 8.0 | 0.0 | 0.00 | 0.00 |
| XECs172 | 1812519 | 1812632 | 114 | 20.8 | 35.2 | 1.85 | 0.56 | 78.4 | 11.7 | 0.15 | 0.47 | 24.0 | 0.4 | 0.02 | 0.39 |
| XECs173 | 1812523 | 1812717 | 195 | 24.5 | 37.0 | 1.49 | 0.63 | 107.9 | 12.5 | 0.11 | 0.50 | 20.1 | 0.3 | 0.02 | 0.31 |
| XECs174 | 1812683 | 1813117 | 435 | 15.0 | 33.0 | 2.27 | 0.58 | 81.8 | 8.1 | 0.10 | 0.66 | 10.0 | 0.6 | 0.06 | 0.24 |
| XECs175 | 1812783 | 1812893 | 111 | 3.8 | 13.1 | 3.48 | 0.66 | 60.7 | 4.4 | 0.08 | 0.75 | 5.3 | 0.9 | 0.18 | 0.45 |
| XECs176 | 1813050 | 1813247 | 198 | 14.1 | 25.1 | 1.97 | 0.62 | 71.1 | 5.6 | 0.08 | 0.62 | 10.1 | 0.1 | 0.01 | 0.04 |
| XECs177 | 1813101 | 1813196 | 96 | 43.2 | 2309.0 | 68.37 | 1.00 | 21.2 | 163.3 | 7.20 | 0.96 | 13.1 | 20.7 | 2.36 | 0.40 |
| XECs178 | 1815562 | 1815675 | 114 | 3.2 | 19.2 | 2.27 | 0.68 | 19.2 | 2.9 | 0.13 | 0.56 | 11.7 | 0.7 | 0.06 | 0.25 |
| XECs179 | 1824008 | 1824109 | 102 | 13.6 | 22.1 | 1.68 | 0.78 | 2.4 | 6.5 | 2.74 | 0.68 | 20.3 | 3.2 | 0.19 | 0.71 |
| XECs180 | 1831619 | 1831744 | 126 | 35.0 | 39.2 | 1.18 | 0.86 | 212.4 | 21.6 | 0.13 | 0.85 | 264.8 | 10.0 | 0.07 | 0.73 |
| XECs181 | 1835831 | 1835992 | 162 | 78.9 | 119.2 | 1.62 | 0.91 | 110.1 | 17.3 | 0.15 | 0.89 | 22.0 | 1.0 | 0.05 | 0.39 |
| XECs182 | 1836002 | 1836103 | 102 | 59.1 | 45.3 | 0.87 | 0.60 | 145.3 | 18.1 | 0.12 | 0.74 | 36.3 | 1.0 | 0.03 | 0.36 |
| XECs183 | 1836279 | 1836371 | 93 | 256.3 | 168.1 | 0.69 | 0.97 | 249.9 | 83.2 | 0.31 | 0.99 | 37.4 | 1.5 | 0.04 | 0.43 |
| XECs184 | 1842478 | 1842627 | 150 | 113.9 | 43.1 | 0.39 | 0.66 | 44.4 | 28.7 | 0.62 | 0.53 | 78.5 | 30.3 | 0.46 | 0.68 |
| XECs185 | 1845035 | 1845127 | 93 | 14.9 | 72.2 | 4.39 | 0.67 | 40.5 | 10.1 | 0.25 | 0.58 | 36.6 | 7.0 | 0.21 | 0.53 |
| XECs186 | 1867113 | 1867277 | 165 | 28.7 | 15.8 | 0.55 | 0.81 | 47.5 | 4.3 | 0.09 | 0.50 | 60.2 | 1.9 | 0.03 | 0.37 |
| XECs187 | 1918780 | 1918947 | 168 | 11.1 | 17.5 | 1.58 | 0.67 | 62.8 | 11.5 | 0.17 | 0.58 | 190.7 | 3.4 | 0.02 | 0.11 |
| XECs188a | 1940772 | 1940927 | 156 | 21.8 | 34.9 | 1.91 | 0.54 | 53.3 | 11.6 | 0.23 | 0.43 | 15.7 | 1.2 | 0.08 | 0.34 |
| XECs189 | 1940786 | 1940905 | 120 | 26.7 | 41.5 | 2.00 | 0.51 | 54.6 | 10.7 | 0.23 | 0.39 | 8.6 | 1.4 | 0.16 | 0.37 |
| XECs190 | 1941866 | 1941967 | 102 | 34.6 | 13.6 | 0.40 | 0.69 | 17.6 | 2.8 | 0.28 | 0.30 | 29.1 | 2.6 | 0.09 | 0.50 |
| XECs191 | 1945984 | 1946130 | 147 | 30.0 | 12.6 | 0.43 | 0.55 | 26.4 | 1.7 | 0.13 | 0.46 | 5.0 | 0.8 | 0.16 | 0.32 |
| XECs192 | 1972292 | 1972405 | 114 | 61.1 | 81.3 | 1.32 | 0.86 | 206.2 | 53.4 | 0.25 | 0.89 | 72.1 | 2.9 | 0.06 | 0.39 |
| XECs193 | 1972321 | 1972422 | 102 | 34.1 | 74.6 | 2.16 | 0.82 | 169.1 | 52.0 | 0.29 | 0.98 | 55.1 | 3.0 | 0.10 | 0.50 |
| XECs194 | 1972547 | 1972669 | 123 | 46.2 | 157.9 | 5.17 | 0.70 | 99.5 | 128.0 | 1.29 | 0.82 | 48.2 | 4.4 | 0.09 | 0.44 |
| XECs195 | 1972617 | 1972781 | 165 | 40.6 | 126.7 | 4.16 | 0.78 | 143.9 | 99.3 | 0.69 | 0.79 | 71.9 | 3.9 | 0.05 | 0.48 |
| XECs196 | 1987225 | 1987374 | 150 | 25.7 | 14.6 | 0.66 | 0.70 | 39.8 | 9.5 | 0.28 | 0.67 | 18.8 | 1.5 | 0.07 | 0.16 |
| XECs197 | 2050091 | 2050309 | 219 | 41.2 | 30.3 | 0.76 | 0.78 | 83.5 | 43.5 | 0.59 | 0.87 | 12.5 | 0.7 | 0.06 | 0.29 |
| XECs198 | 2113764 | 2113856 | 93 | 18.0 | 21.2 | 1.19 | 0.67 | 323.5 | 73.3 | 0.22 | 0.77 | 73.4 | 0.7 | 0.01 | 0.28 |
| XECs199 | 2148538 | 2148633 | 96 | 6650.1 | 4407.1 | 0.69 | 1.00 | 7946.0 | 4139.7 | 0.50 | 1.00 | 2876.5 | 180.3 | 0.16 | 0.98 |
| XECs200 | 2148636 | 2148737 | 102 | 2448.1 | 12340.0 | 5.45 | 1.00 | 3919.3 | 2280.9 | 0.63 | 1.00 | 1851.4 | 181.3 | 0.16 | 0.68 |
| XECs201 | 2148657 | 2148758 | 102 | 397.8 | 221.0 | 0.58 | 0.88 | 418.0 | 75.2 | 0.19 | 0.91 | 654.3 | 25.8 | 0.04 | 0.47 |
| XECs202 | 2148704 | 2148838 | 135 | 397.0 | 190.2 | 0.49 | 0.74 | 390.6 | 71.4 | 0.18 | 0.92 | 640.0 | 20.9 | 0.03 | 0.62 |
| XECs203a | 2158465 | 2158557 | 93 | 66.5 | 34.2 | 0.60 | 0.66 | 503.2 | 8.0 | 0.02 | 0.76 | 48.6 | 9.5 | 0.21 | 0.54 |
| XECs204 | 2161106 | 2161381 | 276 | 29.1 | 16.2 | 0.61 | 0.57 | 21.1 | 4.6 | 0.34 | 0.45 | 20.2 | 1.0 | 0.05 | 0.34 |
| XECs205 | 2188753 | 2188917 | 165 | 6.5 | 15.9 | 3.01 | 0.56 | 46.2 | 6.1 | 0.14 | 0.63 | 16.6 | 1.3 | 0.05 | 0.10 |
| XECs188b | 2189408 | 2189563 | 156 | 12.5 | 34.3 | 3.79 | 0.59 | 46.4 | 13.5 | 0.42 | 0.62 | 20.9 | 1.2 | 0.05 | 0.30 |
| XECs206 | 2189430 | 2189549 | 120 | 12.8 | 41.0 | 5.12 | 0.59 | 45.2 | 14.3 | 0.79 | 0.63 | 14.2 | 1.5 | 0.10 | 0.34 |
| XECs203b | 2205556 | 2205648 | 93 | 52.4 | 22.9 | 0.46 | 0.67 | 43.0 | 6.8 | 0.20 | 0.70 | 12.7 | 9.2 | 0.72 | 0.52 |
| XECs207 | 2209407 | 2209508 | 102 | 204.6 | 144.4 | 0.73 | 0.66 | 537.6 | 176.5 | 0.32 | 0.66 | 467.4 | 2.3 | 0.01 | 0.51 |
| XECs208 | 2211317 | 2211496 | 180 | 29.9 | 54.8 | 1.80 | 0.62 | 24.1 | 11.6 | 0.79 | 0.59 | 16.5 | 0.9 | 0.04 | 0.14 |
| XECs209 | 2217275 | 2217652 | 378 | 105.6 | 36.9 | 0.34 | 0.57 | 59.1 | 7.1 | 0.12 | 0.47 | 64.3 | 5.2 | 0.10 | 0.23 |
| XECs210 | 2237296 | 2237448 | 153 | 213.5 | 81.3 | 0.38 | 0.66 | 67.6 | 16.2 | 0.23 | 0.79 | 177.4 | 9.6 | 0.06 | 0.42 |
| XECs211 | 2237349 | 2237444 | 96 | 44.1 | 18.3 | 0.41 | 0.56 | 41.8 | 3.1 | 0.07 | 0.71 | 27.7 | 0.6 | 0.02 | 0.41 |
| XECs212 | 2240263 | 2240520 | 258 | 8.4 | 34.9 | 3.88 | 0.59 | 48.4 | 13.8 | 0.28 | 0.81 | 23.9 | 2.1 | 0.09 | 0.44 |
| XECs213 | 2240294 | 2240512 | 219 | 8.3 | 39.8 | 4.52 | 0.62 | 37.9 | 15.1 | 0.40 | 0.82 | 18.0 | 2.3 | 0.14 | 0.43 |
| XECs214 | 2255489 | 2255611 | 123 | 235.8 | 66.3 | 0.31 | 0.58 | 118.7 | 77.8 | 0.59 | 0.80 | 92.7 | 4.1 | 0.06 | 0.41 |
| XECs215 | 2257331 | 2257444 | 114 | 84.3 | 69.9 | 0.84 | 0.92 | 28.3 | 28.6 | 0.99 | 0.90 | 90.3 | 13.3 | 0.15 | 0.91 |
| XECs216 | 2273412 | 2273579 | 168 | 181.3 | 272.0 | 1.48 | 0.92 | 325.6 | 225.6 | 0.73 | 0.98 | 101.0 | 5.5 | 0.05 | 0.72 |
| XECs217 | 2277916 | 2278014 | 99 | 11.7 | 13.4 | 1.51 | 0.63 | 6.7 | 1.9 | 0.25 | 0.42 | 3.8 | 0.8 | 0.22 | 0.10 |
| XECs218 | 2337170 | 2337286 | 117 | 3374.5 | 1182.1 | 0.35 | 1.00 | 953.6 | 343.9 | 0.35 | 1.00 | 251.7 | 17.6 | 0.11 | 0.98 |
| XECs219 | 2337340 | 2337564 | 225 | 196.3 | 508.1 | 2.76 | 0.62 | 164.5 | 44.8 | 0.26 | 0.60 | 264.3 | 35.1 | 0.12 | 0.40 |
| XECs220 | 2346305 | 2346442 | 138 | 210.2 | 614.5 | 3.15 | 0.88 | 341.7 | 1354.0 | 3.67 | 0.95 | 121.6 | 8.5 | 0.19 | 0.61 |
| XECs221 | 2358393 | 2358566 | 174 | 20.6 | 86.7 | 4.16 | 0.88 | 15.2 | 44.2 | 2.63 | 0.78 | 68.5 | 9.0 | 0.20 | 0.86 |
| XECs222 | 2358436 | 2358543 | 108 | 27.5 | 77.1 | 2.71 | 0.91 | 19.8 | 29.3 | 1.43 | 0.69 | 95.2 | 9.0 | 0.15 | 0.88 |
| XECs223 | 2398848 | 2398979 | 132 | 144.9 | 126.4 | 0.87 | 0.80 | 299.9 | 35.1 | 0.11 | 0.75 | 200.2 | 2.9 | 0.02 | 0.49 |
| XECs224 | 2402387 | 2402512 | 126 | 152.5 | 40.4 | 0.27 | 0.60 | 158.2 | 11.6 | 0.08 | 0.65 | 122.2 | 1.4 | 0.01 | 0.47 |
| XECs225 | 2404908 | 2405033 | 126 | 14.3 | 20.8 | 1.70 | 0.64 | 32.3 | 7.4 | 0.25 | 0.54 | 30.0 | 0.0 | 0.00 | 0.00 |
| XECs226 | 2421162 | 2421269 | 108 | 37.3 | 15.1 | 0.44 | 0.62 | 29.0 | 3.5 | 0.12 | 0.64 | 21.3 | 0.5 | 0.02 | 0.12 |
| XECs227 | 2480342 | 2480473 | 132 | 110.7 | 174.3 | 1.61 | 0.72 | 111.6 | 86.7 | 0.78 | 0.85 | 42.6 | 6.3 | 0.16 | 0.59 |
| XECs228 | 2480428 | 2480550 | 123 | 168.6 | 203.9 | 1.23 | 1.00 | 135.1 | 112.1 | 0.86 | 1.00 | 60.9 | 8.8 | 0.15 | 0.83 |
| XECs229 | 2480875 | 2481000 | 126 | 50.5 | 26.0 | 0.63 | 0.50 | 95.3 | 31.2 | 0.33 | 0.77 | 103.4 | 11.1 | 0.13 | 0.69 |
| XECs230 | 2507310 | 2507423 | 114 | 1557.9 | 5007.2 | 3.21 | 1.00 | 7063.8 | 1774.4 | 0.25 | 0.99 | 700.9 | 70.5 | 0.14 | 0.96 |
| XECs231 | 2508912 | 2509034 | 123 | 87.7 | 51.9 | 0.57 | 0.87 | 107.5 | 6.4 | 0.06 | 0.70 | 101.7 | 2.6 | 0.03 | 0.57 |
| XECs232 | 2524904 | 2525092 | 189 | 10.1 | 16.6 | 1.58 | 0.54 | 41.9 | 3.2 | 0.08 | 0.55 | 125.2 | 1.0 | 0.01 | 0.26 |
| XECs233 | 2524953 | 2525051 | 99 | 141.8 | 43.9 | 0.31 | 0.80 | 195.9 | 8.4 | 0.04 | 0.66 | 773.2 | 4.3 | 0.01 | 0.66 |
| XECs234 | 2525029 | 2525142 | 114 | 92.2 | 50.8 | 0.53 | 0.90 | 122.1 | 5.7 | 0.05 | 0.65 | 385.4 | 3.7 | 0.01 | 0.68 |
| XECs235 | 2526833 | 2526940 | 108 | 129.1 | 40.9 | 0.33 | 0.77 | 781.5 | 72.5 | 0.09 | 0.97 | 328.6 | 3.8 | 0.01 | 0.71 |
| XECs236 | 2531652 | 2531858 | 207 | 98.5 | 79.2 | 0.80 | 0.68 | 190.0 | 34.3 | 0.19 | 0.72 | 148.3 | 6.6 | 0.05 | 0.72 |
| XECs237 | 2531680 | 2531901 | 222 | 134.0 | 138.2 | 1.03 | 0.73 | 219.7 | 62.8 | 0.29 | 0.80 | 164.9 | 8.0 | 0.05 | 0.79 |
| XECs238 | 2580128 | 2580268 | 141 | 60.9 | 261.8 | 4.50 | 0.78 | 38.4 | 24.2 | 0.56 | 0.70 | 2.1 | 0.9 | 0.41 | 0.24 |
| XECs239 | 2590291 | 2590395 | 105 | 24.4 | 57.9 | 2.50 | 0.86 | 70.7 | 14.9 | 0.31 | 0.75 | 7.1 | 0.3 | 0.04 | 0.07 |
| XECs240 | 2613270 | 2613671 | 402 | 26.1 | 47.3 | 1.83 | 0.66 | 29.0 | 18.0 | 0.56 | 0.78 | 22.7 | 0.5 | 0.02 | 0.15 |
| XECs241 | 2613302 | 2613436 | 135 | 25.1 | 113.3 | 5.53 | 0.84 | 37.0 | 37.0 | 0.96 | 0.92 | 21.4 | 0.5 | 0.05 | 0.15 |
| XECs242 | 2613791 | 2613931 | 141 | 5.0 | 42.9 | 4.13 | 0.78 | 24.5 | 6.7 | 0.32 | 0.70 | 13.7 | 0.6 | 0.03 | 0.19 |
| XECs243 | 2667727 | 2667876 | 150 | 17.4 | 21.6 | 1.67 | 0.51 | 80.3 | 31.9 | 0.39 | 0.77 | 42.0 | 0.4 | 0.01 | 0.10 |
| XECs244 | 2679320 | 2679541 | 222 | 8.1 | 14.5 | 1.81 | 0.57 | 6.2 | 1.0 | 0.17 | 0.26 | 13.4 | 1.2 | 0.09 | 0.32 |
| XECs245 | 2697539 | 2697790 | 252 | 17.1 | 59.6 | 3.46 | 0.71 | 115.8 | 32.3 | 0.28 | 0.84 | 50.9 | 5.8 | 0.11 | 0.47 |
| XECs246 | 2697570 | 2697782 | 213 | 16.3 | 69.2 | 4.19 | 0.77 | 119.1 | 37.1 | 0.31 | 0.85 | 50.8 | 6.4 | 0.12 | 0.44 |
| XECs247 | 2697649 | 2697804 | 156 | 16.9 | 81.0 | 4.72 | 0.66 | 131.1 | 37.7 | 0.29 | 0.67 | 58.4 | 6.5 | 0.11 | 0.29 |
| XECs248 | 2727227 | 2727352 | 126 | 20.2 | 52.3 | 2.68 | 0.75 | 298.5 | 139.4 | 0.47 | 0.63 | 34.7 | 1.0 | 0.03 | 0.24 |
| XECs249 | 2738682 | 2738810 | 129 | 28.5 | 17.2 | 0.72 | 0.71 | 142.9 | 9.8 | 0.07 | 0.74 | 21.2 | 0.2 | 0.00 | 0.09 |
| XECs250 | 2738728 | 2738820 | 93 | 46.0 | 22.2 | 0.53 | 0.84 | 219.2 | 11.7 | 0.05 | 0.79 | 32.6 | 0.6 | 0.07 | 0.03 |
| XECs251 | 2774570 | 2774728 | 159 | 184.0 | 268.7 | 1.46 | 0.90 | 197.6 | 115.6 | 0.52 | 0.89 | 356.1 | 1.6 | 0.01 | 0.47 |
| XECs252 | 2774938 | 2775144 | 207 | 192.9 | 121.4 | 0.63 | 0.86 | 207.8 | 50.4 | 0.24 | 0.87 | 349.2 | 14.9 | 0.07 | 0.84 |
| XECs253 | 2775102 | 2775275 | 174 | 125.2 | 120.4 | 1.00 | 0.72 | 151.1 | 252.2 | 1.58 | 0.77 | 235.5 | 14.5 | 0.08 | 0.72 |
| XECs254 | 2775197 | 2775409 | 213 | 103.4 | 80.6 | 0.80 | 0.57 | 110.4 | 194.2 | 1.58 | 0.70 | 201.4 | 6.4 | 0.05 | 0.58 |
| XECs255 | 2798333 | 2798440 | 108 | 22.8 | 23.2 | 1.01 | 0.69 | 64.4 | 20.7 | 0.36 | 0.70 | 2449.5 | 17.2 | 0.01 | 0.65 |
| XECs256 | 2872220 | 2872318 | 99 | 22.1 | 9.3 | 0.51 | 0.67 | 16.0 | 3.5 | 0.25 | 0.82 | 19.5 | 1.0 | 0.06 | 0.23 |
| XECs257 | 2883244 | 2883423 | 180 | 386.6 | 543.6 | 1.39 | 0.89 | 778.2 | 41.5 | 0.05 | 0.89 | 247.9 | 2.2 | 0.02 | 0.38 |
| XECs258 | 2883764 | 2883895 | 132 | 135.9 | 57.0 | 0.40 | 0.69 | 230.6 | 15.0 | 0.07 | 0.82 | 39.3 | 1.3 | 0.04 | 0.39 |
| XECs259 | 2883853 | 2884056 | 204 | 297.5 | 394.9 | 1.30 | 0.81 | 440.3 | 59.0 | 0.13 | 0.75 | 104.5 | 3.5 | 0.05 | 0.33 |
| XECs260 | 2883905 | 2884027 | 123 | 432.1 | 621.4 | 1.41 | 0.88 | 636.9 | 88.6 | 0.14 | 0.83 | 150.4 | 5.8 | 0.06 | 0.55 |
| XECs261 | 2896556 | 2896753 | 198 | 159.9 | 55.2 | 0.35 | 0.86 | 294.4 | 17.3 | 0.06 | 0.84 | 145.3 | 3.3 | 0.02 | 0.42 |
| XECs262 | 2896566 | 2896697 | 132 | 77.0 | 27.6 | 0.36 | 0.84 | 119.0 | 12.5 | 0.10 | 0.81 | 79.7 | 1.1 | 0.01 | 0.39 |
| XECs263 | 2924348 | 2924452 | 105 | 830.7 | 646.2 | 0.78 | 0.89 | 624.3 | 170.6 | 0.26 | 0.78 | 187.6 | 6.9 | 0.05 | 0.83 |
| XECs264 | 2932278 | 2932385 | 108 | 58.1 | 33.7 | 0.58 | 0.63 | 103.6 | 24.6 | 0.33 | 0.86 | 37.1 | 3.0 | 0.08 | 0.31 |
| XECs265 | 2936766 | 2937089 | 324 | 121.6 | 119.7 | 1.04 | 0.82 | 62.9 | 27.4 | 0.50 | 0.79 | 38.5 | 1.7 | 0.04 | 0.24 |
| XECs266 | 2936806 | 2937135 | 330 | 173.7 | 128.4 | 0.76 | 0.85 | 92.3 | 31.5 | 0.44 | 0.83 | 41.8 | 2.3 | 0.05 | 0.33 |
| XECs267 | 2944526 | 2944633 | 108 | 169.9 | 161.1 | 0.97 | 0.89 | 337.4 | 139.8 | 0.41 | 0.98 | 486.1 | 36.5 | 0.07 | 1.00 |
| XECs268 | 2944587 | 2944694 | 108 | 9.5 | 49.2 | 5.16 | 0.85 | 49.3 | 15.6 | 0.35 | 0.92 | 191.6 | 8.6 | 0.04 | 0.94 |
| XECs269 | 2971507 | 2971656 | 150 | 63.4 | 36.8 | 0.58 | 0.76 | 14.2 | 4.6 | 0.33 | 0.59 | 18.3 | 3.0 | 0.15 | 0.28 |
| XECs270 | 3004814 | 3004930 | 117 | 693.4 | 625.4 | 0.92 | 0.97 | 446.0 | 257.8 | 0.65 | 0.91 | 652.9 | 14.6 | 0.07 | 0.65 |
| XECs271 | 3066056 | 3066154 | 99 | 8.0 | 25.9 | 4.22 | 0.76 | 22.1 | 7.3 | 0.34 | 0.60 | 32.9 | 2.1 | 0.07 | 0.36 |
| XECs272 | 3133048 | 3133182 | 135 | 540.8 | 435.7 | 0.79 | 0.79 | 807.2 | 100.7 | 0.13 | 0.66 | 61.9 | 3.5 | 0.06 | 0.54 |
| XECs273 | 3139310 | 3139408 | 99 | 194.1 | 97.9 | 0.58 | 0.90 | 255.3 | 44.8 | 0.18 | 0.89 | 147.2 | 1.7 | 0.02 | 0.28 |
| XECs274 | 3157141 | 3157272 | 132 | 161.7 | 59.4 | 0.38 | 0.63 | 179.7 | 16.2 | 0.09 | 0.70 | 228.0 | 3.9 | 0.04 | 0.64 |
| XECs275 | 3157232 | 3157375 | 144 | 247.3 | 69.9 | 0.29 | 0.99 | 200.4 | 19.7 | 0.10 | 0.88 | 357.1 | 2.9 | 0.01 | 0.63 |
| XECs276 | 3160269 | 3160376 | 108 | 644.8 | 295.2 | 0.46 | 0.88 | 593.2 | 164.6 | 0.28 | 0.90 | 954.6 | 80.1 | 0.11 | 0.71 |
| XECs277 | 3182567 | 3182668 | 102 | 15.9 | 13.6 | 0.85 | 0.68 | 85.4 | 12.1 | 0.16 | 0.72 | 11.6 | 1.1 | 0.09 | 0.19 |
| XECs278 | 3225006 | 3225122 | 117 | 1245.6 | 860.7 | 0.69 | 0.99 | 416.2 | 221.4 | 0.48 | 0.99 | 91.8 | 3.9 | 0.06 | 0.68 |
| XECs279 | 3237560 | 3237676 | 117 | 921.6 | 793.9 | 0.89 | 0.73 | 332.6 | 653.2 | 1.89 | 0.71 | 384.3 | 74.9 | 0.25 | 0.67 |
| XECs280 | 3244732 | 3244893 | 162 | 16.3 | 32.0 | 2.00 | 0.53 | 49.9 | 27.6 | 0.59 | 0.51 | 71.2 | 1.0 | 0.02 | 0.24 |
| XECs281 | 3306749 | 3306931 | 183 | 817.6 | 388.4 | 0.47 | 0.67 | 970.8 | 175.4 | 0.18 | 0.70 | 348.3 | 2.3 | 0.01 | 0.38 |
| XECs282 | 3306769 | 3306927 | 159 | 941.0 | 447.1 | 0.47 | 0.74 | 1090.2 | 201.9 | 0.18 | 0.77 | 349.8 | 2.7 | 0.01 | 0.41 |
| XECs283 | 3307074 | 3307166 | 93 | 896.3 | 1523.7 | 1.77 | 0.87 | 289.3 | 80.0 | 0.29 | 0.72 | 994.5 | 61.0 | 0.11 | 0.91 |
| XECs284 | 3312923 | 3313039 | 117 | 10.3 | 104.0 | 10.54 | 0.82 | 16.6 | 20.2 | 1.40 | 0.70 | 26.0 | 1.6 | 0.04 | 0.11 |
| XECs285 | 3374320 | 3374502 | 183 | 56.2 | 47.7 | 0.93 | 0.60 | 96.1 | 18.3 | 0.20 | 0.70 | 84.7 | 0.9 | 0.01 | 0.42 |
| XECs286 | 3377018 | 3377122 | 105 | 102.7 | 232.3 | 2.48 | 0.88 | 96.8 | 19.9 | 0.22 | 0.85 | 432.0 | 29.1 | 0.07 | 0.90 |
| XECs287 | 3377073 | 3377180 | 108 | 104.0 | 39.2 | 0.38 | 0.86 | 103.2 | 16.3 | 0.17 | 0.89 | 438.1 | 64.8 | 0.16 | 0.99 |
| XECs288 | 3382798 | 3382890 | 93 | 221.7 | 183.9 | 0.83 | 0.85 | 446.0 | 68.7 | 0.14 | 0.70 | 1507.2 | 43.1 | 0.05 | 0.91 |
| XECs289 | 3409894 | 3410064 | 171 | 63.3 | 29.4 | 0.47 | 0.61 | 77.2 | 9.8 | 0.13 | 0.50 | 69.3 | 6.4 | 0.10 | 0.60 |
| XECs290 | 3416203 | 3416322 | 120 | 5.4 | 12.9 | 2.87 | 0.65 | 30.1 | 6.4 | 0.21 | 0.85 | 9.3 | 1.8 | 0.20 | 0.50 |
| XECs291 | 3420580 | 3420774 | 195 | 436.6 | 155.7 | 0.36 | 0.69 | 382.8 | 43.5 | 0.13 | 0.57 | 122.0 | 1.0 | 0.01 | 0.25 |
| XECs292 | 3420627 | 3420719 | 93 | 452.5 | 297.2 | 0.74 | 0.80 | 417.3 | 76.0 | 0.18 | 0.68 | 179.2 | 1.2 | 0.01 | 0.28 |
| XECs293 | 3424517 | 3424615 | 99 | 35.6 | 15.6 | 0.44 | 0.69 | 40.0 | 4.3 | 0.11 | 0.68 | 80.1 | 0.6 | 0.01 | 0.19 |
| XECs294 | 3441535 | 3442809 | 1275 | 21.2 | 27.1 | 1.30 | 0.62 | 16.0 | 2.3 | 0.15 | 0.42 | 22.6 | 1.2 | 0.08 | 0.25 |
| XECs295 | 3442511 | 3442612 | 102 | 41.5 | 31.1 | 0.73 | 0.86 | 72.9 | 4.6 | 0.07 | 0.68 | 30.6 | 2.7 | 0.09 | 0.72 |
| XECs296 | 3475616 | 3475720 | 105 | 51351.5 | 239574.3 | 4.62 | 1.00 | 64638.8 | 30560.9 | 0.49 | 1.00 | 152717.1 | 11910.3 | 0.08 | 1.00 |
| XECs297 | 3476652 | 3476855 | 204 | 75.5 | 47.3 | 0.63 | 0.61 | 187.2 | 24.2 | 0.14 | 0.61 | 92.0 | 2.0 | 0.02 | 0.48 |
| XECs298 | 3476662 | 3476760 | 99 | 113.9 | 79.5 | 0.71 | 0.81 | 302.6 | 37.0 | 0.14 | 0.86 | 149.1 | 3.1 | 0.02 | 0.69 |
| XECs299 | 3476684 | 3476806 | 123 | 90.2 | 73.3 | 0.82 | 0.80 | 203.1 | 38.1 | 0.21 | 0.79 | 102.5 | 2.3 | 0.02 | 0.52 |
| XECs300 | 3476859 | 3476999 | 141 | 156.6 | 136.6 | 0.88 | 0.88 | 408.2 | 58.6 | 0.16 | 0.85 | 142.1 | 3.3 | 0.03 | 0.72 |
| XECs301 | 3476877 | 3476993 | 117 | 25.4 | 23.2 | 1.00 | 0.75 | 189.6 | 9.6 | 0.05 | 0.81 | 42.4 | 1.4 | 0.04 | 0.50 |
| XECs302 | 3535478 | 3535585 | 108 | 67.5 | 27.2 | 0.41 | 0.79 | 144.9 | 18.1 | 0.13 | 0.82 | 128.3 | 0.7 | 0.01 | 0.38 |
| XECs303 | 3538917 | 3539015 | 99 | 158.1 | 55.1 | 0.34 | 0.75 | 114.2 | 6.2 | 0.05 | 0.52 | 196.7 | 3.7 | 0.02 | 0.45 |
| XECs304 | 3538925 | 3539101 | 177 | 668.3 | 327.0 | 0.49 | 0.57 | 509.9 | 214.7 | 0.41 | 0.67 | 193.3 | 3.3 | 0.02 | 0.48 |
| XECs305 | 3549704 | 3549856 | 153 | 1430.3 | 2059.4 | 1.37 | 0.92 | 340.4 | 364.0 | 1.29 | 0.86 | 89.1 | 2.0 | 0.02 | 0.60 |
| XECs306 | 3549889 | 3549999 | 111 | 89.9 | 609.3 | 6.67 | 1.00 | 39.1 | 33.8 | 0.92 | 0.94 | 24.0 | 3.4 | 0.18 | 0.55 |
| XECs307 | 3549968 | 3550066 | 99 | 25.3 | 129.3 | 4.98 | 1.00 | 31.6 | 17.5 | 0.67 | 0.93 | 9.7 | 0.6 | 0.07 | 0.06 |
| XECs308 | 3550070 | 3550312 | 243 | 46.2 | 77.6 | 1.73 | 0.85 | 33.5 | 11.0 | 0.33 | 0.57 | 16.1 | 2.1 | 0.11 | 0.39 |
| XECs309 | 3550084 | 3550254 | 171 | 46.7 | 65.1 | 1.42 | 0.87 | 26.7 | 4.5 | 0.17 | 0.58 | 14.7 | 2.8 | 0.17 | 0.42 |
| XECs310 | 3550254 | 3550361 | 108 | 52.8 | 160.3 | 3.12 | 0.94 | 42.4 | 17.4 | 0.45 | 0.69 | 32.3 | 1.3 | 0.04 | 0.40 |
| XECs311 | 3550309 | 3550515 | 207 | 49.4 | 146.7 | 2.94 | 1.00 | 34.8 | 16.3 | 0.47 | 0.95 | 35.4 | 1.8 | 0.05 | 0.57 |
| XECs312 | 3550319 | 3550465 | 147 | 56.2 | 158.3 | 2.81 | 1.00 | 34.7 | 9.7 | 0.30 | 0.94 | 43.9 | 1.7 | 0.04 | 0.55 |
| XECs313 | 3550425 | 3551270 | 846 | 29.9 | 29.7 | 0.98 | 0.72 | 12.7 | 4.2 | 0.35 | 0.50 | 13.1 | 1.5 | 0.12 | 0.41 |
| XECs314 | 3550907 | 3551284 | 378 | 43.9 | 32.9 | 0.76 | 0.81 | 6.3 | 3.8 | 0.67 | 0.44 | 6.5 | 1.7 | 0.37 | 0.45 |
| XECs315 | 3551044 | 3551202 | 159 | 16.1 | 41.3 | 2.60 | 0.88 | 1.2 | 4.9 | 4.27 | 0.43 | 4.2 | 2.8 | 0.71 | 0.58 |
| XECs316 | 3628920 | 3629045 | 126 | 616.0 | 1322.1 | 2.14 | 0.68 | 299.6 | 110.9 | 0.39 | 0.71 | 203.9 | 10.9 | 0.07 | 0.49 |
| XECs317 | 3646617 | 3646718 | 102 | 4141.6 | 2997.7 | 0.69 | 1.00 | 512.9 | 215.6 | 0.54 | 1.00 | 355.8 | 47.5 | 0.18 | 1.00 |
| XECs318 | 3646763 | 3646975 | 213 | 5759.3 | 3235.5 | 0.57 | 0.91 | 831.4 | 778.1 | 1.53 | 0.83 | 583.0 | 49.9 | 0.09 | 0.77 |
| XECs319 | 3662413 | 3662574 | 162 | 26.7 | 49.2 | 2.43 | 0.67 | 66.4 | 11.1 | 0.17 | 0.82 | 47.5 | 5.0 | 0.16 | 0.70 |
| XECs320 | 3665572 | 3665694 | 123 | 18.5 | 23.6 | 1.26 | 0.64 | 130.6 | 21.4 | 0.17 | 0.78 | 43.3 | 1.4 | 0.04 | 0.46 |
| XECs321 | 3706677 | 3706793 | 117 | 19.4 | 35.6 | 1.83 | 0.61 | 35.6 | 38.1 | 1.00 | 0.69 | 18.4 | 0.9 | 0.03 | 0.21 |
| XECs322 | 3792484 | 3792789 | 306 | 57.7 | 97.4 | 1.75 | 0.60 | 197.1 | 50.9 | 0.25 | 0.68 | 152.6 | 11.9 | 0.08 | 0.43 |
| XECs323 | 3804724 | 3804846 | 123 | 35.8 | 73.6 | 2.22 | 0.61 | 52.4 | 35.3 | 0.66 | 0.68 | 13.8 | 1.6 | 0.18 | 0.24 |
| XECs324 | 3823205 | 3823309 | 105 | 100.9 | 51.5 | 0.57 | 0.92 | 285.7 | 21.5 | 0.08 | 0.80 | 321.7 | 9.3 | 0.03 | 0.79 |
| XECs325 | 3823278 | 3823442 | 165 | 72.4 | 30.3 | 0.47 | 0.87 | 162.1 | 15.6 | 0.10 | 0.80 | 145.1 | 8.5 | 0.06 | 0.65 |
| XECs326 | 3827548 | 3827712 | 165 | 245.5 | 380.9 | 1.56 | 0.99 | 252.3 | 140.4 | 0.56 | 0.98 | 138.3 | 30.3 | 0.22 | 0.92 |
| XECs327 | 3827627 | 3827737 | 111 | 342.8 | 557.0 | 1.63 | 1.00 | 305.4 | 201.7 | 0.68 | 1.00 | 174.3 | 43.7 | 0.25 | 0.94 |
| XECs328 | 3828045 | 3828161 | 117 | 17.9 | 25.7 | 1.57 | 0.70 | 226.8 | 11.8 | 0.05 | 0.71 | 165.8 | 3.4 | 0.02 | 0.47 |
| XECs329 | 3835469 | 3835633 | 165 | 14.7 | 24.0 | 2.48 | 0.65 | 35.4 | 9.8 | 0.28 | 0.70 | 192.3 | 14.9 | 0.08 | 0.73 |
| XECs330 | 3843241 | 3843378 | 138 | 288.1 | 231.7 | 0.84 | 0.83 | 448.6 | 63.1 | 0.15 | 0.87 | 216.4 | 4.0 | 0.02 | 0.53 |
| XECs331 | 3853749 | 3853883 | 135 | 8.3 | 25.6 | 4.68 | 0.64 | 63.3 | 7.3 | 0.13 | 0.64 | 12.1 | 0.7 | 0.06 | 0.33 |
| XECs332 | 3867433 | 3867579 | 147 | 28.1 | 21.6 | 0.77 | 0.50 | 14.5 | 27.8 | 2.09 | 0.55 | 42.5 | 0.2 | 0.00 | 0.05 |
| XECs333 | 3872707 | 3872937 | 231 | 19.9 | 11.4 | 0.59 | 0.55 | 53.5 | 4.7 | 0.09 | 0.65 | 22.2 | 0.2 | 0.02 | 0.12 |
| XECs334 | 3879941 | 3880051 | 111 | 32.2 | 16.4 | 0.54 | 0.60 | 25.2 | 11.0 | 0.46 | 0.77 | 21.3 | 3.0 | 0.11 | 0.37 |
| XECs335 | 3901529 | 3901621 | 93 | 44.5 | 32.9 | 1.01 | 0.86 | 128.3 | 10.1 | 0.08 | 0.76 | 145.0 | 5.8 | 0.05 | 0.77 |
| XECs336 | 3929536 | 3929634 | 99 | 1041.8 | 452.4 | 0.44 | 0.96 | 455.5 | 150.5 | 0.48 | 0.96 | 121.2 | 3.3 | 0.03 | 0.43 |
| XECs337 | 3934135 | 3934236 | 102 | 6.4 | 109.0 | 21.66 | 0.67 | 48.2 | 20.5 | 0.46 | 0.79 | 52.4 | 4.9 | 0.18 | 0.73 |
| XECs338 | 3942704 | 3942820 | 117 | 34.2 | 27.5 | 0.87 | 0.73 | 97.4 | 14.0 | 0.15 | 0.66 | 81.0 | 5.1 | 0.06 | 0.50 |
| XECs339 | 3974212 | 3974307 | 96 | 3.8 | 19.9 | 1.51 | 0.72 | 24.0 | 11.6 | 0.49 | 0.78 | 7.0 | 0.7 | 0.09 | 0.23 |
| XECs340 | 3987091 | 3987213 | 123 | 11.0 | 12.8 | 2.16 | 0.58 | 22.0 | 4.1 | 0.18 | 0.63 | 41.5 | 2.2 | 0.37 | 0.25 |
| XECs341 | 4009263 | 4009397 | 135 | 277.3 | 126.7 | 0.50 | 0.60 | 348.3 | 51.4 | 0.15 | 0.75 | 272.9 | 6.0 | 0.03 | 0.51 |
| XECs342 | 4009283 | 4009393 | 111 | 337.3 | 141.4 | 0.47 | 0.69 | 421.5 | 51.1 | 0.12 | 0.83 | 331.9 | 7.3 | 0.03 | 0.58 |
| XECs343 | 4060536 | 4060679 | 144 | 495.9 | 264.6 | 0.53 | 0.90 | 1807.0 | 468.9 | 0.22 | 0.95 | 803.6 | 7.7 | 0.01 | 0.82 |
| XECs344 | 4060768 | 4060899 | 132 | 1334.9 | 757.1 | 0.59 | 0.59 | 2757.5 | 276.0 | 0.10 | 0.62 | 944.1 | 43.3 | 0.04 | 0.35 |
| XECs345 | 4064405 | 4064551 | 147 | 33.5 | 13.6 | 0.43 | 0.55 | 246.2 | 13.3 | 0.05 | 0.90 | 193.5 | 3.5 | 0.02 | 0.39 |
| XECs346 | 4071182 | 4071343 | 162 | 360.1 | 632.4 | 1.76 | 0.99 | 709.9 | 344.1 | 0.46 | 0.99 | 566.4 | 25.1 | 0.04 | 0.82 |
| XECs347 | 4088813 | 4088923 | 111 | 151.4 | 130.2 | 0.89 | 0.89 | 119.5 | 32.7 | 0.29 | 0.86 | 159.5 | 25.3 | 0.17 | 0.69 |
| XECs348 | 4092849 | 4093049 | 201 | 257.0 | 737.8 | 3.02 | 0.62 | 63.3 | 42.6 | 0.73 | 0.63 | 77.3 | 14.4 | 0.38 | 0.33 |
| XECs349 | 4136136 | 4136252 | 117 | 243.4 | 82.1 | 0.34 | 0.95 | 330.1 | 74.8 | 0.21 | 1.00 | 176.5 | 2.7 | 0.02 | 0.53 |
| XECs350 | 4139972 | 4140064 | 93 | 32.5 | 9.0 | 0.40 | 0.48 | 114.9 | 38.2 | 0.34 | 0.94 | 30.2 | 2.6 | 0.09 | 0.13 |
| XECs351 | 4140142 | 4140420 | 279 | 633.5 | 1613.3 | 2.56 | 0.96 | 1340.6 | 593.2 | 0.48 | 0.97 | 895.1 | 52.4 | 0.09 | 0.82 |
| XECs352 | 4140224 | 4140370 | 147 | 556.6 | 498.0 | 0.90 | 0.97 | 1311.6 | 182.5 | 0.15 | 0.99 | 913.0 | 49.0 | 0.07 | 0.87 |
| XECs353 | 4166836 | 4167003 | 168 | 289.8 | 94.2 | 0.32 | 0.78 | 486.4 | 40.6 | 0.08 | 0.88 | 169.3 | 2.7 | 0.02 | 0.65 |
| XECs354 | 4196382 | 4196516 | 135 | 80.8 | 83.5 | 1.03 | 0.65 | 62.3 | 10.7 | 0.18 | 0.66 | 136.1 | 5.1 | 0.04 | 0.54 |
| XECs355 | 4197385 | 4197513 | 129 | 152.9 | 42.4 | 0.28 | 0.77 | 258.2 | 19.0 | 0.07 | 0.83 | 204.2 | 3.5 | 0.03 | 0.43 |
| XECs356 | 4205633 | 4205830 | 198 | 288.2 | 269.1 | 0.93 | 0.89 | 167.3 | 71.7 | 0.47 | 0.88 | 229.6 | 10.9 | 0.05 | 0.59 |
| XECs357 | 4205777 | 4206016 | 240 | 312.6 | 285.7 | 0.93 | 0.62 | 304.0 | 49.1 | 0.16 | 0.60 | 201.6 | 11.1 | 0.07 | 0.45 |
| XECs358 | 4213608 | 4213718 | 111 | 21.4 | 18.4 | 1.04 | 0.80 | 9.3 | 2.7 | 0.30 | 0.59 | 22.1 | 0.0 | 0.00 | 0.00 |
| XECs359 | 4238544 | 4238663 | 120 | 157.5 | 53.0 | 0.34 | 0.91 | 244.6 | 30.5 | 0.12 | 0.88 | 104.2 | 2.6 | 0.05 | 0.47 |
| XECs360 | 4256141 | 4256302 | 162 | 90.9 | 238.0 | 2.59 | 0.72 | 85.2 | 39.5 | 0.58 | 0.81 | 18.3 | 1.3 | 0.13 | 0.34 |
| XECs361 | 4256145 | 4256336 | 192 | 79.8 | 201.1 | 2.50 | 0.61 | 75.5 | 33.3 | 0.54 | 0.68 | 20.1 | 1.4 | 0.09 | 0.30 |
| XECs362 | 4289286 | 4289420 | 135 | 63.3 | 53.5 | 0.86 | 0.80 | 60.4 | 6.4 | 0.12 | 0.65 | 49.9 | 1.6 | 0.04 | 0.45 |
| XECs363 | 4294607 | 4294714 | 108 | 15.9 | 74.8 | 4.87 | 0.93 | 0.8 | 4.7 | 4.65 | 0.70 | 3.4 | 0.5 | 0.13 | 0.19 |
| XECs364 | 4310086 | 4310181 | 96 | 16.9 | 12.9 | 0.79 | 0.65 | 42.1 | 15.1 | 0.35 | 0.55 | 35.6 | 1.1 | 0.02 | 0.18 |
| XECs365 | 4348074 | 4348247 | 174 | 23.5 | 32.8 | 1.41 | 0.60 | 122.5 | 18.9 | 0.16 | 0.77 | 98.4 | 2.1 | 0.02 | 0.22 |
| XECs366 | 4365075 | 4365203 | 129 | 93.7 | 76.5 | 0.83 | 0.89 | 218.1 | 35.9 | 0.20 | 1.00 | 95.6 | 4.2 | 0.08 | 0.95 |
| XECs367 | 4365145 | 4365282 | 138 | 81.7 | 54.0 | 0.66 | 0.86 | 217.6 | 59.9 | 0.31 | 0.91 | 72.6 | 3.4 | 0.06 | 0.65 |
| XECs368 | 4374032 | 4374160 | 129 | 68.1 | 100.2 | 1.54 | 0.99 | 61.9 | 38.0 | 0.67 | 0.94 | 21.9 | 6.2 | 0.35 | 0.55 |
| XECs369 | 4374213 | 4374362 | 150 | 740.8 | 216.2 | 0.29 | 0.77 | 547.3 | 47.3 | 0.09 | 0.78 | 86.9 | 3.4 | 0.05 | 0.63 |
| XECs370 | 4374958 | 4375056 | 99 | 82.0 | 47.2 | 0.56 | 0.78 | 215.6 | 19.6 | 0.10 | 0.78 | 218.2 | 1.2 | 0.00 | 0.35 |
| XECs371 | 4405602 | 4405955 | 354 | 19.7 | 23.4 | 1.21 | 0.66 | 32.4 | 48.8 | 1.51 | 0.68 | 14.7 | 2.5 | 0.15 | 0.33 |
| XECs372 | 4405867 | 4405965 | 99 | 28.2 | 69.8 | 2.62 | 0.82 | 55.0 | 161.8 | 2.73 | 0.95 | 20.2 | 2.3 | 0.11 | 0.72 |
| XECs373 | 4424147 | 4424245 | 99 | 23.5 | 34.9 | 1.44 | 0.69 | 42.7 | 4.4 | 0.10 | 0.59 | 38.2 | 3.3 | 0.09 | 0.26 |
| XECs374 | 4437937 | 4438131 | 195 | 73.1 | 121.7 | 1.66 | 0.93 | 127.2 | 22.3 | 0.17 | 0.87 | 130.8 | 8.5 | 0.07 | 0.70 |
| XECs375 | 4437989 | 4438159 | 171 | 57.3 | 119.9 | 2.09 | 0.96 | 111.5 | 16.6 | 0.15 | 0.90 | 127.5 | 7.7 | 0.06 | 0.66 |
| XECs376 | 4449728 | 4449850 | 123 | 26.5 | 41.4 | 1.67 | 0.80 | 46.5 | 20.3 | 0.39 | 0.73 | 67.4 | 1.0 | 0.02 | 0.24 |
| XECs377 | 4467911 | 4468075 | 165 | 15.8 | 40.1 | 3.52 | 0.60 | 253.5 | 68.6 | 0.28 | 0.88 | 48.5 | 3.2 | 0.09 | 0.25 |
| XECs378 | 4512752 | 4512862 | 111 | 7.1 | 24.2 | 4.03 | 0.64 | 15.1 | 9.5 | 0.58 | 0.60 | 4.7 | 9.8 | 1.51 | 0.27 |
| XECs379 | 4516986 | 4517198 | 213 | 49.2 | 75.4 | 1.53 | 0.87 | 66.1 | 69.9 | 1.34 | 0.92 | 35.1 | 2.7 | 0.08 | 0.68 |
| XECs380 | 4561396 | 4561536 | 141 | 38.9 | 16.0 | 0.43 | 0.69 | 40.1 | 7.8 | 0.19 | 0.73 | 37.8 | 3.1 | 0.08 | 0.49 |
| XECs381 | 4561412 | 4561513 | 102 | 38.7 | 16.0 | 0.42 | 0.70 | 31.6 | 4.5 | 0.16 | 0.75 | 45.7 | 3.4 | 0.08 | 0.51 |
| XECs382 | 4580806 | 4580910 | 105 | 6.7 | 18.0 | 0.97 | 0.85 | 43.3 | 1.6 | 0.04 | 0.53 | 17.7 | 2.0 | 0.10 | 0.50 |
| XECs383 | 4580962 | 4581075 | 114 | 27.7 | 165.5 | 6.10 | 0.94 | 140.9 | 29.7 | 0.22 | 0.87 | 65.0 | 2.9 | 0.04 | 0.47 |
| XECs384 | 4580964 | 4581065 | 102 | 26.9 | 184.9 | 7.06 | 0.95 | 154.8 | 33.0 | 0.22 | 0.92 | 64.7 | 3.2 | 0.05 | 0.48 |
| XECs385 | 4585224 | 4585322 | 99 | 8.4 | 19.0 | 2.31 | 0.77 | 23.0 | 5.0 | 0.21 | 0.57 | 21.7 | 3.0 | 0.14 | 0.50 |
| XECs386 | 4613400 | 4613498 | 99 | 8.0 | 14.0 | 2.17 | 0.60 | 41.1 | 6.8 | 0.18 | 0.67 | 8.2 | 0.0 | 0.00 | 0.00 |
| XECs387 | 4643862 | 4644008 | 147 | 102.6 | 90.8 | 0.88 | 0.68 | 130.6 | 10.1 | 0.10 | 0.66 | 122.9 | 2.4 | 0.02 | 0.61 |
| XECs388 | 4643918 | 4644028 | 111 | 127.2 | 186.5 | 1.43 | 0.72 | 148.4 | 23.9 | 0.18 | 0.82 | 132.7 | 2.8 | 0.02 | 0.72 |
| XECs389 | 4654580 | 4654702 | 123 | 48.4 | 26.7 | 0.64 | 0.59 | 28.7 | 3.7 | 0.13 | 0.48 | 10.3 | 4.4 | 0.31 | 0.25 |
| XECs390 | 4669919 | 4670107 | 189 | 280.6 | 202.6 | 0.72 | 0.88 | 511.4 | 143.2 | 0.27 | 0.93 | 402.1 | 14.8 | 0.04 | 0.66 |
| XECs391 | 4670014 | 4670139 | 126 | 249.3 | 195.9 | 0.79 | 0.85 | 502.1 | 159.7 | 0.31 | 0.92 | 378.4 | 6.4 | 0.02 | 0.60 |
| XECs392 | 4670275 | 4670376 | 102 | 64.2 | 59.0 | 0.93 | 0.56 | 154.7 | 26.4 | 0.20 | 0.59 | 118.2 | 1.3 | 0.02 | 0.47 |
| XECs393 | 4670309 | 4670476 | 168 | 847.0 | 249.7 | 0.29 | 0.88 | 2596.9 | 97.2 | 0.04 | 0.87 | 1710.6 | 16.9 | 0.02 | 0.60 |
| XECs394 | 4670437 | 4670553 | 117 | 1910.3 | 1159.1 | 0.60 | 1.00 | 5891.3 | 990.7 | 0.16 | 1.00 | 3355.7 | 47.7 | 0.03 | 1.00 |
| XECs395 | 4671046 | 4671303 | 258 | 118.9 | 447.2 | 3.79 | 0.93 | 546.8 | 237.5 | 0.52 | 0.93 | 801.7 | 113.9 | 0.20 | 0.91 |
| XECs396 | 4719231 | 4719344 | 114 | 59.9 | 20.3 | 0.34 | 0.76 | 220.6 | 50.4 | 0.27 | 0.75 | 371.4 | 33.6 | 0.13 | 0.74 |
| XECs397 | 4734916 | 4735008 | 93 | 1605.6 | 718.0 | 0.48 | 0.93 | 7586.2 | 378.7 | 0.05 | 0.95 | 4960.8 | 87.9 | 0.02 | 0.91 |
| XECs398 | 4735086 | 4735202 | 117 | 17588.8 | 4559.0 | 0.26 | 1.00 | 46123.4 | 2271.8 | 0.05 | 1.00 | 25889.3 | 434.9 | 0.02 | 1.00 |
| XECs399 | 4743477 | 4743599 | 123 | 6.4 | 18.1 | 2.70 | 0.54 | 79.2 | 21.2 | 0.31 | 0.60 | 56.0 | 1.6 | 0.05 | 0.20 |
| XECs400 | 4753259 | 4753378 | 120 | 493.3 | 168.2 | 0.34 | 0.98 | 243.8 | 48.6 | 0.21 | 0.99 | 169.0 | 4.4 | 0.03 | 0.68 |
| XECs401 | 4757644 | 4757763 | 120 | 211.7 | 78.0 | 0.38 | 0.90 | 159.9 | 40.3 | 0.25 | 0.83 | 235.4 | 13.7 | 0.06 | 0.62 |
| XECs402 | 4776425 | 4776559 | 135 | 186.7 | 123.1 | 0.81 | 0.75 | 945.9 | 27.3 | 0.05 | 0.43 | 1142.0 | 2.4 | 0.00 | 0.38 |
| XECs403 | 4795776 | 4795877 | 102 | 1027.7 | 775.9 | 0.78 | 0.89 | 669.6 | 130.7 | 0.19 | 0.97 | 747.0 | 113.9 | 0.22 | 0.49 |
| XECs404 | 4802031 | 4802168 | 138 | 128.4 | 43.0 | 0.34 | 0.75 | 119.3 | 21.0 | 0.16 | 0.75 | 139.6 | 23.8 | 0.16 | 0.65 |
| XECs405 | 4802053 | 4802211 | 159 | 123.1 | 57.7 | 0.47 | 0.85 | 124.5 | 20.8 | 0.15 | 0.84 | 147.2 | 20.8 | 0.13 | 0.66 |
| XECs406 | 4849561 | 4849671 | 111 | 1020.9 | 1571.4 | 1.55 | 1.00 | 263.9 | 171.1 | 0.64 | 0.99 | 234.9 | 23.2 | 0.10 | 0.91 |
| XECs407 | 4908505 | 4908633 | 129 | 146.9 | 64.9 | 0.44 | 0.75 | 206.8 | 36.3 | 0.17 | 0.83 | 112.5 | 5.2 | 0.07 | 0.39 |
| XECs408 | 4908564 | 4908656 | 93 | 124.8 | 107.9 | 0.87 | 0.76 | 190.2 | 48.5 | 0.23 | 0.81 | 93.1 | 1.9 | 0.02 | 0.38 |
| XECs409 | 4918345 | 4918479 | 135 | 19.6 | 14.9 | 0.77 | 0.61 | 38.9 | 3.8 | 0.10 | 0.57 | 29.1 | 0.5 | 0.02 | 0.24 |
| XECs410 | 4928782 | 4928877 | 96 | 138.2 | 164.3 | 1.20 | 0.88 | 517.1 | 90.3 | 0.20 | 0.95 | 407.8 | 27.8 | 0.12 | 0.74 |
| XECs411 | 4933659 | 4933856 | 198 | 24.9 | 68.8 | 3.57 | 0.76 | 99.4 | 14.3 | 0.15 | 0.91 | 47.2 | 3.6 | 0.08 | 0.34 |
| XECs412 | 4933666 | 4933878 | 213 | 28.6 | 68.4 | 3.66 | 0.77 | 93.7 | 14.5 | 0.16 | 0.91 | 43.9 | 3.8 | 0.08 | 0.42 |
| XECs413 | 4933878 | 4934147 | 270 | 19.6 | 115.3 | 6.20 | 0.89 | 56.2 | 63.5 | 1.08 | 0.91 | 16.2 | 1.8 | 0.12 | 0.35 |
| XECs414 | 4955248 | 4955421 | 174 | 23.5 | 17.8 | 0.76 | 0.49 | 38.3 | 2.9 | 0.09 | 0.39 | 50.6 | 1.2 | 0.02 | 0.33 |
| XECs415 | 4972701 | 4972853 | 153 | 21.0 | 47.8 | 2.24 | 0.91 | 96.6 | 11.1 | 0.11 | 0.72 | 98.7 | 2.6 | 0.03 | 0.63 |
| XECs416 | 4972795 | 4972908 | 114 | 22.0 | 92.1 | 4.24 | 0.97 | 103.8 | 11.8 | 0.13 | 0.89 | 135.1 | 4.5 | 0.04 | 0.66 |
| XECs417 | 4987337 | 4987450 | 114 | 587.1 | 495.6 | 0.84 | 0.79 | 1109.5 | 749.1 | 0.58 | 0.86 | 615.0 | 3.7 | 0.01 | 0.51 |
| XECs418 | 4988677 | 4988811 | 135 | 14141.8 | 4505.4 | 0.32 | 1.00 | 24303.5 | 1532.0 | 0.07 | 0.99 | 29835.8 | 597.3 | 0.02 | 1.00 |
| XECs419 | 4990025 | 4990129 | 105 | 1358.7 | 1079.4 | 0.82 | 0.91 | 1036.7 | 360.2 | 0.35 | 0.97 | 3032.1 | 358.4 | 0.13 | 0.76 |
| XECs420 | 5008943 | 5009125 | 183 | 5965.8 | 2146.0 | 0.36 | 1.00 | 3975.9 | 724.4 | 0.19 | 1.00 | 1991.3 | 58.2 | 0.06 | 0.95 |
| XECs421 | 5008948 | 5009082 | 135 | 3293.6 | 2294.6 | 0.72 | 1.00 | 2167.8 | 581.6 | 0.27 | 1.00 | 1677.0 | 56.6 | 0.07 | 0.94 |
| XECs422 | 5040127 | 5040270 | 144 | 21.6 | 72.8 | 3.42 | 0.93 | 65.5 | 55.9 | 0.82 | 0.93 | 126.4 | 14.6 | 0.16 | 0.88 |
| XECs423 | 5040280 | 5040714 | 435 | 13.4 | 27.8 | 2.13 | 0.74 | 58.5 | 13.4 | 0.23 | 0.84 | 69.5 | 14.7 | 0.21 | 0.79 |
| XECs424 | 5040575 | 5040703 | 129 | 7.9 | 42.4 | 5.37 | 0.94 | 63.6 | 21.2 | 0.33 | 0.97 | 91.4 | 28.8 | 0.35 | 0.95 |
| XECs425 | 5040725 | 5040838 | 114 | 28.5 | 13.3 | 0.47 | 0.58 | 100.8 | 5.8 | 0.06 | 0.83 | 99.4 | 4.8 | 0.05 | 0.65 |
| XECs426 | 5077280 | 5077447 | 168 | 2027.5 | 1074.3 | 0.55 | 0.63 | 1473.9 | 289.0 | 0.18 | 0.69 | 144.5 | 2.9 | 0.02 | 0.50 |
| XECs427 | 5086499 | 5086657 | 159 | 153.2 | 130.1 | 0.84 | 0.84 | 99.4 | 13.0 | 0.16 | 0.66 | 113.7 | 42.0 | 0.38 | 0.81 |
| XECs428 | 5086524 | 5086694 | 171 | 128.6 | 110.9 | 0.84 | 0.85 | 84.8 | 11.9 | 0.18 | 0.70 | 87.1 | 39.3 | 0.46 | 0.77 |
| XECs429 | 5127815 | 5127940 | 126 | 104.9 | 26.5 | 0.25 | 0.83 | 231.2 | 29.6 | 0.12 | 0.61 | 184.4 | 7.7 | 0.07 | 0.42 |
| XECs430 | 5127861 | 5127989 | 129 | 20.9 | 14.8 | 0.71 | 0.63 | 89.0 | 11.2 | 0.13 | 0.70 | 50.0 | 1.2 | 0.02 | 0.37 |
| XECs431 | 5131100 | 5131357 | 258 | 51.6 | 46.4 | 0.92 | 0.64 | 29.6 | 6.7 | 0.23 | 0.60 | 31.6 | 2.1 | 0.07 | 0.50 |
| XECs432 | 5136349 | 5136447 | 99 | 14.0 | 15.3 | 1.18 | 0.60 | 30.3 | 9.8 | 0.40 | 0.82 | 10.5 | 0.3 | 0.02 | 0.04 |
| XECs433 | 5140794 | 5140913 | 120 | 215.1 | 98.6 | 0.46 | 0.78 | 439.0 | 29.3 | 0.07 | 0.75 | 33.9 | 1.8 | 0.09 | 0.47 |
| XECs434 | 5180683 | 5180823 | 141 | 116.5 | 45.7 | 0.40 | 0.95 | 319.5 | 11.9 | 0.04 | 0.90 | 64.6 | 2.2 | 0.04 | 0.54 |
| XECs435 | 5184772 | 5184954 | 183 | 101.0 | 352.7 | 4.11 | 0.75 | 2176.5 | 512.1 | 0.56 | 0.98 | 647.3 | 53.4 | 0.24 | 0.79 |
| XECs436 | 5186523 | 5186651 | 129 | 2976.8 | 1807.9 | 0.61 | 0.96 | 454.9 | 112.5 | 0.27 | 0.96 | 296.4 | 17.8 | 0.06 | 0.70 |
| XECs437 | 5216828 | 5216941 | 114 | 8.5 | 52.8 | 8.13 | 0.77 | 142.5 | 34.3 | 0.23 | 0.86 | 65.7 | 3.4 | 0.05 | 0.51 |
| XECs438 | 5216838 | 5216936 | 99 | 9.8 | 59.0 | 8.01 | 0.81 | 156.1 | 38.5 | 0.24 | 0.89 | 74.2 | 4.0 | 0.06 | 0.54 |
| XECs439 | 5216845 | 5217000 | 156 | 14.6 | 67.0 | 4.62 | 0.76 | 261.2 | 44.0 | 0.18 | 0.88 | 333.8 | 3.2 | 0.01 | 0.44 |
| XECs440 | 5230525 | 5230653 | 129 | 258.4 | 236.0 | 0.91 | 0.98 | 440.0 | 105.8 | 0.24 | 1.00 | 243.7 | 37.0 | 0.19 | 0.94 |
| XECs441 | 5230571 | 5230720 | 150 | 76.1 | 246.6 | 3.36 | 0.98 | 215.3 | 135.3 | 0.61 | 1.00 | 66.6 | 13.8 | 0.23 | 0.95 |
| XECs442 | 5262853 | 5262999 | 147 | 119.1 | 86.8 | 0.73 | 0.80 | 279.8 | 75.3 | 0.27 | 0.77 | 636.7 | 30.2 | 0.06 | 0.83 |
| XECs443 | 5269595 | 5269720 | 126 | 30.2 | 229.1 | 8.30 | 0.76 | 26.0 | 10.6 | 0.47 | 0.58 | 5.3 | 2.0 | 0.41 | 0.42 |
| XECs444 | 5278683 | 5278829 | 147 | 804.5 | 1695.3 | 2.10 | 0.74 | 1574.8 | 572.0 | 0.36 | 0.59 | 1096.8 | 188.0 | 0.17 | 0.52 |
| XECs445 | 5295279 | 5295377 | 99 | 30.1 | 15.9 | 0.51 | 0.77 | 37.5 | 6.5 | 0.17 | 0.71 | 212.3 | 0.8 | 0.00 | 0.32 |
| XECs446 | 5297713 | 5297889 | 177 | 59.3 | 30.2 | 0.51 | 0.61 | 111.2 | 27.1 | 0.24 | 0.68 | 76.9 | 2.8 | 0.11 | 0.30 |
| XECs447 | 5312600 | 5312794 | 195 | 78.3 | 66.8 | 0.85 | 0.97 | 530.5 | 28.0 | 0.06 | 0.95 | 534.1 | 14.3 | 0.03 | 0.87 |
| XECs448 | 5318228 | 5318368 | 141 | 294.1 | 159.0 | 0.53 | 0.91 | 75.1 | 17.2 | 0.21 | 0.83 | 22.1 | 0.2 | 0.01 | 0.05 |
| XECs449 | 5322904 | 5323005 | 102 | 4321.1 | 7001.4 | 1.62 | 1.00 | 5219.9 | 2706.9 | 0.50 | 1.00 | 2848.2 | 84.3 | 0.03 | 1.00 |
| XECs450 | 5325943 | 5326065 | 123 | 226.3 | 343.3 | 1.55 | 0.78 | 336.3 | 118.7 | 0.33 | 0.82 | 197.1 | 9.5 | 0.05 | 0.50 |
| XECs451 | 5341530 | 5341652 | 123 | 374.5 | 746.0 | 2.01 | 0.91 | 505.1 | 207.4 | 0.41 | 0.91 | 275.9 | 34.3 | 0.14 | 0.80 |
| XECs452 | 5351801 | 5351911 | 111 | 3.8 | 14.2 | 3.85 | 0.62 | 17.2 | 8.0 | 0.70 | 0.69 | 12.7 | 3.0 | 0.26 | 0.46 |
| XECs453 | 5352365 | 5352484 | 120 | 52.9 | 35.2 | 0.79 | 0.69 | 117.2 | 9.6 | 0.08 | 0.72 | 132.0 | 2.0 | 0.02 | 0.33 |
| XECs454 | 5373639 | 5373785 | 147 | 129.0 | 66.0 | 0.52 | 0.79 | 91.9 | 11.2 | 0.13 | 0.72 | 41.9 | 2.0 | 0.05 | 0.35 |
| XECs455 | 5390006 | 5390131 | 126 | 16.6 | 8.6 | 0.54 | 0.42 | 39.3 | 18.3 | 0.48 | 0.68 | 13.0 | 2.2 | 0.09 | 0.16 |
| XECs456 | 5433143 | 5433301 | 159 | 39.4 | 18.2 | 0.46 | 0.54 | 65.9 | 8.5 | 0.13 | 0.68 | 41.4 | 0.9 | 0.05 | 0.36 |
| XECs457 | 5448991 | 5449152 | 162 | 67.1 | 36.0 | 0.54 | 0.65 | 97.8 | 40.7 | 0.41 | 0.81 | 15.5 | 2.1 | 0.14 | 0.36 |
| XECs458 | 5470119 | 5470265 | 147 | 15.5 | 32.8 | 2.11 | 0.72 | 16.4 | 19.7 | 1.68 | 0.60 | 39.8 | 7.4 | 0.23 | 0.71 |
| XECs459 | 5470144 | 5470332 | 189 | 17.4 | 32.4 | 1.86 | 0.72 | 15.0 | 23.2 | 1.85 | 0.73 | 34.1 | 7.7 | 0.29 | 0.84 |
| XECs460 | 5473718 | 5473810 | 93 | 11.5 | 20.6 | 2.08 | 0.78 | 14.0 | 16.2 | 6.36 | 0.93 | 12.0 | 2.9 | 0.24 | 0.37 |
| XECs461 | 5473866 | 5473979 | 114 | 49.0 | 77.4 | 1.67 | 0.97 | 41.8 | 46.0 | 1.15 | 1.00 | 5.9 | 0.5 | 0.07 | 0.10 |
| XECs462 | 5474208 | 5474333 | 126 | 51.2 | 240.6 | 4.70 | 0.95 | 53.4 | 57.7 | 1.52 | 1.00 | 10.6 | 1.0 | 0.09 | 0.32 |
| XECs463 | 5474604 | 5474732 | 129 | 35.4 | 51.4 | 1.56 | 0.85 | 16.7 | 10.9 | 0.64 | 0.96 | 6.9 | 0.9 | 0.17 | 0.32 |
| XECs464 | 5485379 | 5485543 | 165 | 54.8 | 19.5 | 0.36 | 0.84 | 41.4 | 12.4 | 0.31 | 0.78 | 67.8 | 4.3 | 0.06 | 0.52 |
| XECs465 | 5497286 | 5497402 | 117 | 837.5 | 817.7 | 1.00 | 0.92 | 1941.9 | 246.3 | 0.13 | 0.85 | 454.7 | 12.7 | 0.03 | 0.58 |
| ECs0001 | 190 | 273 | 84 | 352.3 | 459.2 | 1.38 | 1.00 | 873.4 | 97.0 | 0.11 | 1.00 | 437.0 | 29.8 | 0.10 | 0.96 |
| ECs0079 | 88229 | 88315 | 87 | 141.7 | 138.7 | 1.12 | 0.99 | 248.6 | 26.0 | 0.10 | 0.97 | 288.5 | 13.6 | 0.06 | 0.72 |
| ECs0105 | 116138 | 116335 | 198 | 67.5 | 141.6 | 2.10 | 0.96 | 56.0 | 47.8 | 0.85 | 0.94 | 40.8 | 5.8 | 0.19 | 0.43 |
| ECs0232 | 262677 | 262901 | 225 | 11.1 | 9.1 | 0.82 | 0.35 | 15.2 | 3.6 | 0.35 | 0.40 | 7.9 | 1.0 | 0.14 | 0.43 |
| ECs0239 | 271928 | 272110 | 183 | 17.7 | 12.5 | 0.70 | 0.34 | 102.8 | 26.9 | 0.22 | 0.69 | 27.1 | 0.4 | 0.02 | 0.31 |
| ECs0243 | 275535 | 275705 | 171 | 11.4 | 3.8 | 0.49 | 0.26 | 33.5 | 6.6 | 0.25 | 0.36 | 10.8 | 0.9 | 0.08 | 0.22 |
| ECs0275 | 302753 | 302953 | 201 | 87.7 | 35.0 | 0.40 | 0.44 | 28.8 | 20.4 | 0.62 | 0.59 | 5.9 | 0.4 | 0.06 | 0.39 |
| ECs0301 | 319000 | 319221 | 222 | 6.5 | 47.5 | 7.37 | 0.48 | 23.5 | 16.5 | 0.71 | 0.54 | 9.7 | 3.8 | 0.38 | 0.45 |
| ECs0326 | 344521 | 344712 | 192 | 8.9 | 16.7 | 4.19 | 0.49 | 55.5 | 14.1 | 0.26 | 0.56 | 66.1 | 0.8 | 0.01 | 0.23 |
| ECs0439 | 466758 | 466949 | 192 | 48.9 | 63.7 | 1.43 | 0.77 | 190.8 | 47.0 | 0.24 | 0.87 | 606.9 | 15.8 | 0.04 | 0.75 |
| ECs0513 | 546155 | 546373 | 219 | 119.1 | 151.9 | 1.42 | 0.92 | 552.5 | 164.1 | 0.28 | 0.95 | 299.4 | 13.9 | 0.06 | 0.76 |
| ECs0519 | 556002 | 556166 | 165 | 20.3 | 156.8 | 8.11 | 0.87 | 37.2 | 40.6 | 1.05 | 1.00 | 13.9 | 3.3 | 0.58 | 0.36 |
| ECs0590 | 649113 | 649325 | 213 | 332.4 | 579.0 | 1.78 | 1.00 | 183.3 | 140.1 | 0.77 | 1.00 | 180.8 | 29.9 | 0.15 | 0.68 |
| ECs0662 | 738647 | 738856 | 210 | 2614.4 | 2344.6 | 0.89 | 1.00 | 1477.9 | 1187.9 | 0.85 | 1.00 | 5728.8 | 513.7 | 0.09 | 0.71 |
| ECs0665 | 740303 | 740506 | 204 | 1714.5 | 2103.6 | 1.23 | 1.00 | 569.3 | 522.1 | 0.91 | 0.99 | 955.2 | 167.8 | 0.24 | 0.57 |
| ECs0728 | 807561 | 807767 | 207 | 4393.3 | 11166.5 | 2.52 | 1.00 | 2452.2 | 1783.4 | 0.76 | 1.00 | 1190.2 | 261.0 | 0.38 | 0.48 |
| ECs0790 | 878646 | 878795 | 150 | 385.5 | 914.3 | 2.44 | 1.00 | 346.1 | 341.6 | 0.95 | 1.00 | 649.0 | 157.3 | 0.30 | 0.76 |
| ECs0805 | 893721 | 893942 | 222 | 23.4 | 30.7 | 1.31 | 0.44 | 13.5 | 2.3 | 0.25 | 0.48 | 9.4 | 0.6 | 0.05 | 0.13 |
| ECs0808 | 894497 | 894679 | 183 | 7.6 | 12.3 | 1.48 | 0.30 | 5.5 | 2.1 | 0.50 | 0.21 | 2.0 | 0.0 | 0.00 | 0.00 |
| ECs0818 | 901806 | 902021 | 216 | 17.4 | 12.7 | 0.73 | 0.50 | 24.6 | 3.7 | 0.15 | 0.53 | 6.9 | 0.4 | 0.05 | 0.06 |
| ECs0822 | 902970 | 903122 | 153 | 16.7 | 23.5 | 1.80 | 0.38 | 14.4 | 12.5 | 0.72 | 0.35 | 18.9 | 5.7 | 0.17 | 0.38 |
| ECs0826 | 906387 | 906599 | 213 | 10.2 | 4.8 | 0.48 | 0.42 | 7.7 | 3.1 | 0.65 | 0.40 | 20.9 | 2.4 | 0.11 | 0.13 |
| ECs0849 | 928398 | 928532 | 135 | 20.0 | 12.6 | 1.74 | 0.54 | 22.7 | 8.3 | 0.39 | 0.58 | 9.9 | 0.6 | 0.06 | 0.08 |
| ECs0966 | 1053441 | 1053665 | 225 | 214.2 | 404.3 | 1.88 | 1.00 | 87.5 | 42.2 | 0.52 | 0.90 | 39.6 | 2.2 | 0.06 | 0.38 |
| ECs0969 | 1057302 | 1057520 | 219 | 1587.4 | 1813.7 | 1.14 | 1.00 | 1331.4 | 778.6 | 0.57 | 1.00 | 1373.1 | 232.4 | 0.22 | 0.91 |
| ECs1000 | 1100315 | 1100497 | 183 | 842.6 | 317.8 | 0.39 | 0.99 | 331.4 | 131.6 | 0.40 | 0.99 | 409.0 | 33.0 | 0.10 | 0.58 |
| ECs1037 | 1145186 | 1145353 | 168 | 323.3 | 240.6 | 0.74 | 1.00 | 100.1 | 11.2 | 0.11 | 1.00 | 898.8 | 22.3 | 0.03 | 0.78 |
| ECs1058 | 1165169 | 1165360 | 192 | 79.3 | 41.8 | 0.53 | 0.85 | 43.5 | 43.1 | 0.94 | 0.94 | 15.0 | 1.8 | 0.10 | 0.09 |
| ECs1059 | 1165357 | 1165545 | 189 | 80.5 | 55.4 | 0.69 | 0.70 | 48.3 | 25.8 | 0.49 | 0.80 | 18.8 | 0.6 | 0.05 | 0.36 |
| ECs1061 | 1166119 | 1166304 | 186 | 12.2 | 68.2 | 5.58 | 0.51 | 33.8 | 30.8 | 1.04 | 0.69 | 9.6 | 0.7 | 0.07 | 0.10 |
| ECs1065 | 1167022 | 1167177 | 156 | 41.9 | 61.7 | 1.49 | 0.67 | 29.8 | 27.0 | 1.08 | 0.66 | 15.7 | 2.4 | 0.17 | 0.30 |
| ECs1068 | 1167742 | 1167933 | 192 | 421.0 | 352.4 | 0.89 | 0.78 | 333.4 | 178.7 | 0.55 | 0.96 | 222.8 | 20.5 | 0.10 | 0.73 |
| ECs1098 | 1185513 | 1185707 | 195 | 24.2 | 13.6 | 0.60 | 0.45 | 29.6 | 3.8 | 0.14 | 0.45 | 6.5 | 1.0 | 0.24 | 0.22 |
| ECs1100 | 1186168 | 1186374 | 207 | 18.2 | 28.3 | 1.54 | 0.62 | 13.6 | 2.6 | 0.19 | 0.45 | 9.3 | 0.7 | 0.05 | 0.15 |
| ECs1119 | 1202439 | 1202645 | 207 | 20.0 | 5.1 | 0.25 | 0.35 | 9.7 | 0.3 | 0.03 | 0.14 | 2.9 | 0.1 | 0.03 | 0.19 |
| ECs1125 | 1209796 | 1209978 | 183 | 4.3 | 3.4 | 0.86 | 0.24 | 13.1 | 0.6 | 0.05 | 0.30 | 19.9 | 0.2 | 0.02 | 0.17 |
| ECs1127 | 1210459 | 1210641 | 183 | 84.8 | 97.3 | 1.16 | 0.78 | 499.0 | 78.8 | 0.15 | 0.89 | 271.7 | 9.7 | 0.03 | 0.38 |
| ECs1144 | 1229317 | 1229529 | 213 | 5.7 | 10.9 | 1.79 | 0.47 | 98.6 | 12.2 | 0.12 | 0.88 | 24.7 | 1.2 | 0.05 | 0.61 |
| ECs1145 | 1229815 | 1230027 | 213 | 192.3 | 468.9 | 2.49 | 1.00 | 1604.7 | 1352.0 | 0.92 | 1.00 | 838.6 | 63.4 | 0.09 | 0.75 |
| ECs1159 | 1245217 | 1245444 | 228 | 31.2 | 47.4 | 1.55 | 0.70 | 27.2 | 23.9 | 0.86 | 0.77 | 82.5 | 14.4 | 0.18 | 0.47 |
| ECs1170 | 1250884 | 1251105 | 222 | 46.9 | 46.6 | 1.08 | 0.68 | 14.8 | 3.7 | 0.36 | 0.62 | 20.1 | 0.4 | 0.01 | 0.18 |
| ECs1172 | 1251496 | 1251687 | 192 | 41.7 | 42.4 | 1.36 | 0.29 | 26.6 | 8.1 | 0.34 | 0.33 | 31.3 | 0.3 | 0.01 | 0.29 |
| ECs1173 | 1251660 | 1251842 | 183 | 40.6 | 26.4 | 0.78 | 0.62 | 28.9 | 6.5 | 0.24 | 0.61 | 17.8 | 0.1 | 0.01 | 0.04 |
| ECs1178 | 1253790 | 1253954 | 165 | 214.6 | 61.3 | 0.32 | 0.57 | 177.4 | 25.5 | 0.16 | 0.72 | 82.2 | 0.6 | 0.01 | 0.22 |
| ECs1186 | 1257683 | 1257898 | 216 | 451.7 | 177.1 | 0.40 | 0.87 | 176.4 | 132.9 | 1.05 | 0.88 | 41.2 | 2.6 | 0.06 | 0.69 |
| ECs1188 | 1258369 | 1258515 | 147 | 118.0 | 43.1 | 0.36 | 0.94 | 112.5 | 15.7 | 0.17 | 0.98 | 27.3 | 0.6 | 0.01 | 0.40 |
| ECs1193 | 1261584 | 1261799 | 216 | 42.2 | 24.6 | 0.64 | 0.64 | 31.1 | 15.2 | 0.45 | 0.69 | 4.1 | 0.1 | 0.02 | 0.04 |
| ECs1210 | 1272078 | 1272257 | 180 | 40.8 | 22.5 | 0.67 | 0.85 | 35.2 | 14.9 | 0.51 | 0.82 | 11.5 | 0.8 | 0.09 | 0.41 |
| ECs1212 | 1272647 | 1272862 | 216 | 14.4 | 7.9 | 0.55 | 0.40 | 9.3 | 2.6 | 0.27 | 0.32 | 11.7 | 0.7 | 0.05 | 0.30 |
| ECs1248 | 1306798 | 1307016 | 219 | 112.2 | 43.2 | 0.41 | 0.64 | 68.5 | 12.6 | 0.20 | 0.65 | 69.8 | 1.4 | 0.02 | 0.21 |
| ECs1357 | 1417073 | 1417276 | 204 | 14.2 | 11.7 | 1.60 | 0.24 | 6.4 | 1.0 | 0.21 | 0.15 | 19.3 | 0.6 | 0.03 | 0.47 |
| ECs1359 | 1417912 | 1418136 | 225 | 19.2 | 13.1 | 0.68 | 0.45 | 18.2 | 4.8 | 0.36 | 0.43 | 12.5 | 0.8 | 0.04 | 0.13 |
| ECs1367 | 1424971 | 1425165 | 195 | 6.4 | 5.7 | 0.90 | 0.47 | 25.4 | 2.7 | 0.11 | 0.46 | 5.3 | 0.1 | 0.02 | 0.09 |
| ECs1379 | 1431113 | 1431328 | 216 | 96.4 | 40.8 | 0.44 | 0.50 | 178.7 | 13.6 | 0.08 | 0.74 | 123.9 | 2.7 | 0.02 | 0.52 |
| ECs1392 | 1441434 | 1441655 | 222 | 17.4 | 5.3 | 0.34 | 0.28 | 43.9 | 1.9 | 0.04 | 0.31 | 14.0 | 0.0 | 0.00 | 0.00 |
| ECs1436 | 1478448 | 1478588 | 141 | 7.2 | 5.3 | 0.70 | 0.51 | 4.3 | 0.7 | 0.20 | 0.16 | 7.9 | 2.1 | 0.36 | 0.51 |
| ECs1467 | 1506462 | 1506635 | 174 | 5228.0 | 6050.8 | 1.21 | 1.00 | 10023.6 | 3692.0 | 0.36 | 1.00 | 6636.0 | 455.5 | 0.07 | 0.99 |
| ECs1505 | 1544196 | 1544426 | 231 | 61.3 | 21.0 | 0.34 | 0.64 | 74.6 | 5.8 | 0.09 | 0.45 | 36.9 | 1.5 | 0.05 | 0.14 |
| ECs1517 | 1549924 | 1550115 | 192 | 25.1 | 49.0 | 2.13 | 0.72 | 19.2 | 18.6 | 1.39 | 0.57 | 22.7 | 1.5 | 0.30 | 0.26 |
| ECs1530 | 1557757 | 1557972 | 216 | 8.4 | 8.4 | 1.03 | 0.49 | 6.6 | 0.8 | 0.13 | 0.29 | 5.1 | 0.3 | 0.07 | 0.14 |
| ECs1536 | 1560390 | 1560614 | 225 | 10.1 | 6.7 | 0.68 | 0.35 | 9.3 | 3.1 | 0.41 | 0.51 | 16.5 | 0.5 | 0.03 | 0.22 |
| ECs1537 | 1560611 | 1560829 | 219 | 3.0 | 2.1 | 0.95 | 0.18 | 1.9 | 0.3 | 0.11 | 0.09 | 0.7 | 0.1 | 0.14 | 0.09 |
| ECs1538 | 1560971 | 1561111 | 141 | 57.6 | 26.1 | 0.46 | 0.98 | 32.7 | 7.3 | 0.23 | 0.81 | 2.1 | 0.6 | 0.29 | 0.13 |
| ECs1539 | 1561241 | 1561426 | 186 | 45.6 | 11.1 | 0.25 | 0.65 | 22.8 | 6.0 | 0.44 | 0.57 | 4.8 | 0.1 | 0.08 | 0.00 |
| ECs1565 | 1587113 | 1587274 | 162 | 53.6 | 23.1 | 0.43 | 0.67 | 53.4 | 8.3 | 0.19 | 0.65 | 50.9 | 3.4 | 0.05 | 0.28 |
| ECs1569 | 1589630 | 1589842 | 213 | 5.7 | 3.3 | 0.65 | 0.19 | 13.5 | 16.6 | 1.03 | 0.40 | 15.3 | 0.6 | 0.09 | 0.16 |
| ECs1577 | 1597360 | 1597557 | 198 | 10.3 | 10.1 | 1.03 | 0.33 | 12.7 | 3.8 | 0.40 | 0.56 | 4.5 | 0.7 | 0.16 | 0.15 |
| ECs1579 | 1597735 | 1597926 | 192 | 6.5 | 4.7 | 0.73 | 0.35 | 6.5 | 6.7 | 2.10 | 0.45 | 1.9 | 0.1 | 0.05 | 0.10 |
| ECs1596 | 1606591 | 1606764 | 174 | 3.5 | 1.1 | 0.25 | 0.05 | 3.0 | 1.8 | 0.60 | 0.18 | 7.3 | 0.0 | 0.00 | 0.00 |
| ECs1624 | 1627575 | 1627757 | 183 | 5.8 | 6.9 | 1.32 | 0.25 | 37.3 | 7.0 | 0.31 | 0.51 | 3.6 | 0.5 | 0.13 | 0.13 |
| ECs1627 | 1629129 | 1629335 | 207 | 205.4 | 141.5 | 0.75 | 0.72 | 195.2 | 86.5 | 0.49 | 0.74 | 9.3 | 0.2 | 0.02 | 0.05 |
| ECs1631 | 1632525 | 1632731 | 207 | 10.3 | 7.0 | 0.79 | 0.51 | 21.1 | 2.4 | 0.12 | 0.41 | 8.9 | 1.4 | 0.27 | 0.22 |
| ECs1655 | 1655162 | 1655344 | 183 | 8.1 | 6.8 | 1.00 | 0.45 | 12.1 | 2.6 | 0.51 | 0.56 | 44.5 | 5.1 | 0.11 | 0.49 |
| ECs1673 | 1668988 | 1669188 | 201 | 54.8 | 19.7 | 0.39 | 0.77 | 49.9 | 1.8 | 0.04 | 0.54 | 32.1 | 0.9 | 0.03 | 0.14 |
| ECs1722 | 1717461 | 1717691 | 231 | 11.4 | 15.0 | 1.28 | 0.65 | 17.9 | 8.8 | 0.47 | 0.70 | 19.9 | 3.3 | 0.32 | 0.63 |
| ECs1764 | 1763025 | 1763243 | 219 | 18.2 | 15.5 | 0.84 | 0.58 | 34.2 | 9.4 | 0.33 | 0.80 | 26.8 | 0.8 | 0.04 | 0.22 |
| ECs1766 | 1764363 | 1764590 | 228 | 10.2 | 6.9 | 0.79 | 0.50 | 7.3 | 1.5 | 0.23 | 0.40 | 3.9 | 0.7 | 0.17 | 0.34 |
| ECs1782 | 1776701 | 1776916 | 216 | 9.7 | 7.6 | 0.77 | 0.49 | 7.0 | 0.9 | 0.16 | 0.21 | 11.3 | 0.1 | 0.00 | 0.24 |
| ECs1788 | 1779673 | 1779900 | 228 | 27.7 | 21.3 | 0.75 | 0.55 | 20.7 | 4.5 | 0.22 | 0.43 | 6.8 | 0.3 | 0.04 | 0.23 |
| ECs1856 | 1843816 | 1844034 | 219 | 118.1 | 315.2 | 3.24 | 0.96 | 244.1 | 431.7 | 2.07 | 0.97 | 1600.8 | 681.3 | 0.56 | 0.83 |
| ECs1882 | 1876620 | 1876844 | 225 | 72.0 | 99.1 | 1.39 | 0.98 | 70.9 | 45.4 | 0.65 | 1.00 | 153.9 | 9.8 | 0.10 | 0.79 |
| ECs1884 | 1877212 | 1877433 | 222 | 27.0 | 104.3 | 3.86 | 0.84 | 45.3 | 20.5 | 0.46 | 0.86 | 112.0 | 5.9 | 0.07 | 0.66 |
| ECs1939 | 1928627 | 1928782 | 156 | 15.2 | 6.1 | 0.54 | 0.37 | 12.1 | 2.5 | 0.36 | 0.55 | 7.1 | 0.2 | 0.04 | 0.05 |
| ECs1940 | 1928793 | 1928972 | 180 | 15.5 | 6.0 | 0.52 | 0.40 | 23.0 | 3.1 | 0.15 | 0.61 | 11.9 | 1.7 | 0.40 | 0.13 |
| ECs1949 | 1933334 | 1933546 | 213 | 21.4 | 10.5 | 0.69 | 0.46 | 22.5 | 3.0 | 0.13 | 0.46 | 10.1 | 0.7 | 0.07 | 0.29 |
| ECs1960 | 1941418 | 1941585 | 168 | 10.8 | 17.5 | 1.63 | 0.60 | 39.2 | 5.6 | 0.14 | 0.60 | 22.0 | 2.5 | 0.10 | 0.30 |
| ECs1962 | 1943999 | 1944214 | 216 | 27.1 | 10.9 | 0.48 | 0.42 | 12.2 | 2.2 | 0.18 | 0.35 | 18.2 | 1.8 | 0.07 | 0.43 |
| ECs1967 | 1946971 | 1947198 | 228 | 37.2 | 26.0 | 0.68 | 0.57 | 30.3 | 5.4 | 0.25 | 0.58 | 5.2 | 0.1 | 0.02 | 0.20 |
| ECs1988 | 1963300 | 1963506 | 207 | 16.6 | 6.3 | 0.41 | 0.41 | 11.8 | 0.7 | 0.07 | 0.21 | 7.2 | 0.2 | 0.03 | 0.27 |
| ECs2004 | 1983189 | 1983374 | 186 | 34.2 | 21.4 | 0.62 | 0.85 | 17.3 | 7.4 | 0.49 | 0.73 | 9.6 | 1.3 | 0.16 | 0.38 |
| ECs2024 | 2009186 | 2009359 | 174 | 65.4 | 62.7 | 0.96 | 0.65 | 18.5 | 12.1 | 0.78 | 0.56 | 32.9 | 0.7 | 0.03 | 0.36 |
| ECs2025 | 2009471 | 2009638 | 168 | 150.6 | 65.1 | 0.53 | 0.76 | 22.2 | 5.9 | 0.28 | 0.63 | 18.1 | 2.0 | 0.12 | 0.21 |
| ECs2031 | 2016160 | 2016384 | 225 | 53.1 | 84.5 | 1.57 | 0.73 | 28.5 | 6.3 | 0.20 | 0.43 | 70.4 | 1.2 | 0.03 | 0.14 |
| ECs2040 | 2024597 | 2024827 | 231 | 21.9 | 24.5 | 1.17 | 0.68 | 34.3 | 19.9 | 0.70 | 0.81 | 27.2 | 0.1 | 0.00 | 0.12 |
| ECs2049 | 2033104 | 2033325 | 222 | 13.0 | 17.2 | 1.30 | 0.44 | 41.9 | 6.0 | 0.23 | 0.42 | 90.1 | 9.0 | 0.11 | 0.32 |
| ECs2059 | 2042705 | 2042917 | 213 | 5.0 | 3.8 | 1.05 | 0.12 | 7.0 | 3.2 | 0.63 | 0.26 | 5.9 | 1.9 | 0.18 | 0.10 |
| ECs2084 | 2077069 | 2077206 | 138 | 89.3 | 145.6 | 1.72 | 0.83 | 73.6 | 59.0 | 0.79 | 0.79 | 904.5 | 156.1 | 0.21 | 0.82 |
| ECs2085 | 2077308 | 2077523 | 216 | 9.9 | 2.3 | 0.30 | 0.19 | 13.2 | 6.5 | 0.51 | 0.62 | 243.5 | 26.7 | 0.10 | 0.97 |
| ECs2105 | 2105007 | 2105204 | 198 | 7.0 | 4.7 | 0.50 | 0.17 | 14.7 | 1.7 | 0.12 | 0.18 | 13.1 | 1.5 | 0.11 | 0.14 |
| ECs2139 | 2144401 | 2144619 | 219 | 82.8 | 75.7 | 1.08 | 0.95 | 213.9 | 42.1 | 0.19 | 0.89 | 66.6 | 5.0 | 0.10 | 0.64 |
| ECs2178 | 2179423 | 2179629 | 207 | 9.4 | 7.5 | 1.06 | 0.47 | 20.8 | 1.7 | 0.08 | 0.30 | 10.7 | 1.5 | 0.15 | 0.25 |
| ECs2181 | 2182458 | 2182682 | 225 | 65.9 | 17.2 | 0.28 | 0.40 | 94.6 | 15.6 | 0.23 | 0.48 | 13.9 | 0.7 | 0.06 | 0.18 |
| ECs2188 | 2185931 | 2186137 | 207 | 9.2 | 10.5 | 1.30 | 0.48 | 4.4 | 1.8 | 0.83 | 0.48 | 5.0 | 0.1 | 0.04 | 0.20 |
| ECs2192 | 2190645 | 2190842 | 198 | 19.4 | 8.4 | 0.41 | 0.40 | 54.2 | 4.7 | 0.10 | 0.64 | 15.7 | 0.1 | 0.01 | 0.24 |
| ECs2202 | 2195566 | 2195793 | 228 | 4.4 | 4.5 | 1.06 | 0.33 | 6.4 | 1.0 | 0.28 | 0.27 | 0.0 | 0.9 | 0.00 | 0.15 |
| ECs2212 | 2200897 | 2201106 | 210 | 46.2 | 34.0 | 0.74 | 0.71 | 23.9 | 24.8 | 0.95 | 0.73 | 13.8 | 0.4 | 0.03 | 0.21 |
| ECs2214 | 2201671 | 2201859 | 189 | 6.9 | 1.5 | 0.38 | 0.07 | 8.7 | 3.6 | 0.38 | 0.15 | 2.7 | 0.1 | 0.04 | 0.05 |
| ECs2253 | 2233397 | 2233540 | 144 | 10.9 | 39.9 | 4.53 | 0.70 | 15.0 | 6.0 | 0.39 | 0.63 | 17.0 | 1.9 | 0.15 | 0.27 |
| ECs2255 | 2233910 | 2234137 | 228 | 49.9 | 28.0 | 0.57 | 0.59 | 25.5 | 8.1 | 0.54 | 0.62 | 4.6 | 0.1 | 0.04 | 0.29 |
| ECs2261 | 2236894 | 2237100 | 207 | 6.7 | 6.6 | 1.16 | 0.43 | 11.2 | 1.6 | 0.14 | 0.49 | 9.7 | 0.2 | 0.02 | 0.16 |
| ECs2271 | 2243902 | 2244006 | 105 | 73.0 | 532.3 | 7.20 | 0.91 | 153.3 | 226.6 | 1.59 | 0.87 | 68.4 | 1.6 | 0.03 | 0.40 |
| ECs2280 | 2248879 | 2249106 | 228 | 786.7 | 2181.6 | 2.84 | 0.98 | 854.7 | 1106.4 | 1.34 | 0.99 | 250.1 | 9.4 | 0.04 | 0.67 |
| ECs2282 | 2249591 | 2249755 | 165 | 9.0 | 7.3 | 0.95 | 0.44 | 54.1 | 4.4 | 0.08 | 0.50 | 37.3 | 0.3 | 0.01 | 0.43 |
| ECs2284 | 2250897 | 2251085 | 189 | 5.7 | 2.3 | 0.43 | 0.24 | 5.8 | 3.7 | 0.86 | 0.22 | 0.8 | 0.2 | 0.25 | 0.13 |
| ECs2333 | 2304182 | 2304307 | 126 | 550.9 | 180.9 | 0.36 | 0.97 | 1001.8 | 243.9 | 0.22 | 1.00 | 363.4 | 11.8 | 0.07 | 0.90 |
| ECs2334 | 2304580 | 2304795 | 216 | 292.2 | 126.9 | 0.44 | 1.00 | 681.9 | 184.4 | 0.26 | 1.00 | 225.0 | 12.5 | 0.05 | 0.89 |
| ECs2382 | 2354644 | 2354853 | 210 | 88.6 | 43.8 | 0.49 | 0.49 | 104.9 | 34.9 | 0.41 | 0.77 | 68.2 | 1.6 | 0.02 | 0.22 |
| ECs2424 | 2399515 | 2399712 | 198 | 1289.9 | 2906.0 | 2.31 | 1.00 | 2471.1 | 2673.4 | 1.12 | 1.00 | 1358.5 | 580.5 | 0.43 | 1.00 |
| ECs2497 | 2475683 | 2475835 | 153 | 58.3 | 64.7 | 1.12 | 0.49 | 25.1 | 7.2 | 0.50 | 0.76 | 48.9 | 0.8 | 0.03 | 0.51 |
| ECs2505 | 2481007 | 2481189 | 183 | 29.0 | 104.0 | 3.92 | 0.58 | 248.2 | 140.2 | 0.56 | 0.97 | 278.3 | 44.2 | 0.22 | 0.87 |
| ECs2520 | 2496381 | 2496560 | 180 | 256.1 | 236.7 | 0.93 | 0.94 | 165.2 | 70.7 | 0.42 | 1.00 | 221.9 | 12.1 | 0.06 | 0.49 |
| ECs2526 | 2503402 | 2503611 | 210 | 12.4 | 11.2 | 1.24 | 0.37 | 30.7 | 6.2 | 0.19 | 0.49 | 22.3 | 0.3 | 0.01 | 0.16 |
| ECs2533 | 2509056 | 2509265 | 210 | 2991.8 | 1938.6 | 0.64 | 1.00 | 781.2 | 406.4 | 0.54 | 1.00 | 788.8 | 39.7 | 0.06 | 0.87 |
| ECs2534 | 2509278 | 2509421 | 144 | 1552.3 | 844.6 | 0.54 | 1.00 | 547.6 | 80.0 | 0.15 | 0.97 | 740.0 | 97.9 | 0.13 | 1.00 |
| ECs2536 | 2510452 | 2510595 | 144 | 611.9 | 481.9 | 0.80 | 0.87 | 747.8 | 577.9 | 0.77 | 0.97 | 464.1 | 10.8 | 0.03 | 0.75 |
| ECs2622 | 2595881 | 2596084 | 204 | 23.7 | 41.3 | 1.94 | 0.47 | 54.8 | 4.6 | 0.09 | 0.34 | 22.5 | 0.5 | 0.06 | 0.29 |
| ECs2627 | 2597642 | 2597845 | 204 | 15.7 | 16.1 | 1.18 | 0.34 | 22.5 | 4.4 | 0.18 | 0.47 | 12.0 | 0.2 | 0.04 | 0.04 |
| ECs2653 | 2618089 | 2618313 | 225 | 134.5 | 663.2 | 4.93 | 0.99 | 327.3 | 270.1 | 0.78 | 1.00 | 144.1 | 10.9 | 0.11 | 0.71 |
| ECs2691 | 2649181 | 2649369 | 189 | 149.2 | 202.3 | 1.39 | 0.97 | 84.1 | 64.3 | 0.75 | 0.99 | 84.7 | 6.8 | 0.09 | 0.96 |
| ECs2695 | 2652734 | 2652916 | 183 | 85.5 | 352.6 | 4.23 | 0.90 | 53.7 | 78.8 | 1.67 | 0.74 | 64.1 | 3.8 | 0.05 | 0.46 |
| ECs2734 | 2687036 | 2687242 | 207 | 2.9 | 19.7 | 7.55 | 0.55 | 9.4 | 3.1 | 0.38 | 0.58 | 8.6 | 0.6 | 0.16 | 0.17 |
| ECs2743 | 2693005 | 2693211 | 207 | 9.4 | 12.5 | 1.96 | 0.50 | 6.0 | 0.9 | 0.24 | 0.29 | 7.2 | 0.2 | 0.03 | 0.20 |
| ECs2748 | 2696990 | 2697157 | 168 | 21.6 | 26.7 | 1.36 | 0.72 | 170.2 | 22.6 | 0.13 | 0.67 | 56.8 | 2.4 | 0.04 | 0.36 |
| ECs2755 | 2701170 | 2701274 | 105 | 132.6 | 885.0 | 6.79 | 0.95 | 167.7 | 116.7 | 0.76 | 0.99 | 164.6 | 3.8 | 0.02 | 0.65 |
| ECs2758 | 2702382 | 2702570 | 189 | 7.4 | 24.3 | 3.79 | 0.50 | 7.9 | 5.9 | 1.61 | 0.71 | 6.3 | 1.0 | 0.15 | 0.35 |
| ECs2760 | 2702920 | 2703144 | 225 | 9.3 | 16.6 | 1.80 | 0.54 | 6.6 | 5.3 | 0.79 | 0.46 | 6.3 | 1.0 | 0.25 | 0.29 |
| ECs2765 | 2705881 | 2706108 | 228 | 5.3 | 55.1 | 10.79 | 0.56 | 16.5 | 27.6 | 1.68 | 0.61 | 10.7 | 2.1 | 0.18 | 0.40 |
| ECs2769 | 2707943 | 2708131 | 189 | 4.7 | 0.5 | 0.25 | 0.04 | 11.7 | 0.4 | 0.04 | 0.03 | 4.3 | 0.0 | 0.00 | 0.00 |
| ECs2772 | 2710758 | 2710961 | 204 | 10.2 | 11.9 | 1.31 | 0.15 | 7.1 | 2.2 | 0.31 | 0.50 | 4.4 | 1.6 | 0.22 | 0.16 |
| ECs2804 | 2749324 | 2749545 | 222 | 1.9 | 2.5 | 1.51 | 0.13 | 6.4 | 0.4 | 0.07 | 0.11 | 1.3 | 0.5 | 0.38 | 0.30 |
| ECs2814 | 2756733 | 2756960 | 228 | 6.7 | 10.7 | 1.82 | 0.51 | 43.4 | 13.6 | 0.34 | 0.89 | 41.0 | 2.4 | 0.06 | 0.54 |
| ECs2833 | 2774427 | 2774591 | 165 | 166.5 | 48.1 | 0.32 | 0.87 | 150.5 | 19.0 | 0.12 | 0.94 | 275.2 | 6.2 | 0.02 | 0.78 |
| ECs2890 | 2842627 | 2842806 | 180 | 7.2 | 7.9 | 1.52 | 0.61 | 14.5 | 10.7 | 0.77 | 0.67 | 7.0 | 0.8 | 0.18 | 0.48 |
| ECs2969 | 2921137 | 2921352 | 216 | 13.8 | 9.4 | 0.79 | 0.34 | 12.1 | 1.3 | 0.09 | 0.26 | 4.1 | 0.9 | 0.13 | 0.15 |
| ECs2971 | 2921716 | 2921895 | 180 | 47.3 | 21.1 | 0.53 | 0.69 | 49.7 | 13.7 | 0.34 | 0.72 | 11.1 | 0.8 | 0.06 | 0.39 |
| ECs2978 | 2927439 | 2927648 | 210 | 2.0 | 0.6 | 0.26 | 0.06 | 4.0 | 0.7 | 0.16 | 0.29 | 1.8 | 0.2 | 0.11 | 0.08 |
| ECs2980 | 2928012 | 2928188 | 177 | 3.7 | 2.6 | 1.26 | 0.24 | 3.4 | 1.8 | 0.44 | 0.18 | 1.7 | 0.2 | 0.12 | 0.04 |
| ECs2989 | 2932423 | 2932641 | 219 | 635.0 | 364.7 | 0.57 | 1.00 | 353.0 | 143.2 | 0.67 | 1.00 | 228.3 | 9.3 | 0.04 | 0.68 |
| ECs2997 | 2936444 | 2936608 | 165 | 196.1 | 66.2 | 0.34 | 0.84 | 150.1 | 29.1 | 0.23 | 0.92 | 80.5 | 0.6 | 0.01 | 0.49 |
| ECs3003 | 2938551 | 2938739 | 189 | 161.7 | 184.8 | 1.13 | 0.84 | 43.2 | 53.5 | 1.30 | 0.70 | 57.7 | 4.2 | 0.07 | 0.36 |
| ECs3004 | 2938712 | 2938903 | 192 | 173.1 | 192.3 | 1.10 | 0.63 | 67.9 | 58.0 | 1.02 | 0.68 | 71.9 | 4.9 | 0.06 | 0.45 |
| ECs3006 | 2939294 | 2939515 | 222 | 176.4 | 74.9 | 0.43 | 0.75 | 41.8 | 13.7 | 0.45 | 0.71 | 38.4 | 4.9 | 0.12 | 0.28 |
| ECs3079 | 3019059 | 3019286 | 228 | 64.9 | 55.7 | 1.05 | 0.88 | 86.3 | 28.7 | 0.31 | 0.77 | 115.9 | 16.7 | 0.16 | 0.83 |
| ECs3087 | 3028930 | 3029139 | 210 | 38.8 | 16.9 | 0.49 | 0.57 | 46.6 | 21.4 | 0.86 | 0.80 | 41.8 | 7.5 | 0.11 | 0.21 |
| ECs3239 | 3200272 | 3200448 | 177 | 367.7 | 254.6 | 0.69 | 0.99 | 375.3 | 148.3 | 0.46 | 1.00 | 162.0 | 13.2 | 0.08 | 0.42 |
| ECs3366 | 3345432 | 3345623 | 192 | 55.8 | 64.1 | 1.15 | 0.79 | 117.9 | 44.3 | 0.39 | 0.96 | 66.8 | 1.5 | 0.02 | 0.11 |
| ECs3372 | 3351741 | 3351956 | 216 | 178.1 | 276.5 | 1.56 | 0.89 | 158.8 | 62.9 | 0.36 | 0.97 | 218.3 | 18.6 | 0.09 | 0.62 |
| ECs3390 | 3377191 | 3377391 | 201 | 315.9 | 83.8 | 0.26 | 0.93 | 343.6 | 64.1 | 0.21 | 0.98 | 1766.4 | 48.7 | 0.03 | 0.96 |
| ECs3479 | 3473803 | 3473955 | 153 | 116.0 | 92.3 | 0.77 | 0.94 | 166.8 | 39.0 | 0.21 | 0.97 | 100.5 | 11.9 | 0.11 | 0.54 |
| ECs3497 | 3483887 | 3484102 | 216 | 9.2 | 8.0 | 0.87 | 0.44 | 6.2 | 0.5 | 0.12 | 0.24 | 5.1 | 0.6 | 0.15 | 0.20 |
| ECs3513 | 3498740 | 3498946 | 207 | 2.0 | 2.2 | 1.26 | 0.09 | 8.8 | 0.6 | 0.08 | 0.17 | 3.6 | 0.0 | 0.00 | 0.00 |
| ECs3527 | 3519057 | 3519215 | 159 | 47.4 | 81.7 | 1.92 | 0.82 | 65.8 | 152.3 | 2.01 | 1.00 | 86.6 | 17.8 | 0.24 | 0.74 |
| ECs3553 | 3542889 | 3543074 | 186 | 2215.3 | 1920.8 | 0.87 | 1.00 | 1755.3 | 921.6 | 0.49 | 1.00 | 1557.0 | 79.1 | 0.07 | 0.75 |
| ECs3690 | 3693671 | 3693889 | 219 | 536.9 | 1218.8 | 2.25 | 0.85 | 435.8 | 1134.1 | 2.55 | 0.89 | 980.2 | 81.7 | 0.11 | 0.68 |
| ECs3710 | 3716277 | 3716495 | 219 | 2.8 | 1.1 | 0.38 | 0.13 | 4.7 | 1.5 | 0.32 | 0.29 | 2.0 | 0.2 | 0.10 | 0.04 |
| ECs3815 | 3827406 | 3827552 | 147 | 172.7 | 385.9 | 2.31 | 0.72 | 182.4 | 104.9 | 0.57 | 0.95 | 108.2 | 6.8 | 0.09 | 0.59 |
| ECs3852 | 3858950 | 3859150 | 201 | 5.6 | 2.8 | 1.51 | 0.15 | 3.5 | 0.1 | 0.03 | 0.05 | 9.2 | 0.5 | 0.05 | 0.10 |
| ECs3854 | 3860922 | 3861149 | 228 | 8.4 | 4.3 | 0.61 | 0.34 | 9.1 | 2.4 | 0.26 | 0.39 | 6.2 | 0.8 | 0.26 | 0.15 |
| ECs3864 | 3870594 | 3870710 | 117 | 8.3 | 13.7 | 2.08 | 0.59 | 17.9 | 10.6 | 0.87 | 0.33 | 24.7 | 1.1 | 0.05 | 0.37 |
| ECs3891 | 3892334 | 3892489 | 156 | 41.7 | 17.1 | 0.45 | 0.40 | 129.8 | 6.4 | 0.06 | 0.46 | 1362.8 | 5.1 | 0.00 | 0.66 |
| ECs3931 | 3931268 | 3931468 | 201 | 251.7 | 433.7 | 1.69 | 0.94 | 126.2 | 40.8 | 0.31 | 0.83 | 85.8 | 4.4 | 0.06 | 0.69 |
| ECs3948 | 3950312 | 3950527 | 216 | 4937.2 | 3470.1 | 0.70 | 1.00 | 7054.0 | 3779.4 | 0.51 | 1.00 | 7561.7 | 1082.4 | 0.14 | 0.93 |
| ECs3989 | 3994666 | 3994836 | 171 | 179.9 | 692.7 | 3.88 | 1.00 | 174.1 | 125.9 | 0.73 | 1.00 | 150.2 | 18.5 | 0.22 | 0.66 |
| ECs4115 | 4124368 | 4124571 | 204 | 17.1 | 4.7 | 0.26 | 0.37 | 13.3 | 4.0 | 0.28 | 0.63 | 10.6 | 0.2 | 0.04 | 0.16 |
| ECs4135 | 4147580 | 4147759 | 180 | 37.9 | 29.4 | 0.93 | 0.67 | 15.9 | 6.4 | 0.38 | 0.59 | 3.3 | 1.5 | 0.45 | 0.33 |
| ECs4140 | 4153394 | 4153615 | 222 | 272.3 | 220.8 | 0.79 | 0.98 | 60.5 | 26.7 | 0.44 | 0.98 | 229.4 | 15.9 | 0.08 | 0.42 |
| ECs4164 | 4177630 | 4177746 | 117 | 2201.0 | 9901.3 | 4.58 | 1.00 | 4133.6 | 5342.3 | 1.35 | 1.00 | 3581.6 | 2291.1 | 0.94 | 1.00 |
| ECs4167 | 4179555 | 4179734 | 180 | 5273.3 | 6443.8 | 1.23 | 1.00 | 6925.6 | 4974.5 | 0.69 | 1.00 | 7700.9 | 1094.6 | 0.14 | 1.00 |
| ECs4177 | 4183580 | 4183771 | 192 | 11093.0 | 20098.5 | 1.82 | 1.00 | 18307.2 | 3508.1 | 0.19 | 1.00 | 15407.9 | 575.6 | 0.04 | 1.00 |
| ECs4199 | 4197546 | 4197764 | 219 | 377.7 | 1213.3 | 3.22 | 0.99 | 412.8 | 583.7 | 1.34 | 0.99 | 360.7 | 36.0 | 0.10 | 0.96 |
| ECs4205 | 4204124 | 4204342 | 219 | 45.6 | 72.1 | 1.55 | 0.82 | 39.7 | 37.2 | 0.86 | 0.92 | 65.3 | 10.0 | 0.16 | 0.47 |
| ECs4220 | 4219362 | 4219529 | 168 | 428.7 | 280.6 | 0.65 | 0.97 | 267.9 | 137.3 | 0.75 | 1.00 | 241.6 | 31.4 | 0.12 | 0.71 |
| ECs4250 | 4252338 | 4252565 | 228 | 29.9 | 69.7 | 2.39 | 0.95 | 53.9 | 24.4 | 0.59 | 0.92 | 63.0 | 8.5 | 0.14 | 0.69 |
| ECs4381 | 4391722 | 4391928 | 207 | 2.0 | 1.6 | 0.89 | 0.16 | 6.0 | 0.8 | 0.17 | 0.18 | 19.6 | 0.6 | 0.11 | 0.42 |
| ECs4415 | 4438143 | 4438331 | 189 | 155.3 | 216.7 | 1.41 | 0.66 | 226.7 | 40.8 | 0.19 | 0.70 | 281.7 | 43.2 | 0.16 | 0.45 |
| ECs4441 | 4467623 | 4467835 | 213 | 3452.0 | 7967.4 | 2.32 | 1.00 | 16407.4 | 20602.9 | 1.21 | 1.00 | 10736.1 | 881.3 | 0.17 | 0.58 |
| ECs4511 | 4555109 | 4555276 | 168 | 2653.9 | 2538.2 | 0.98 | 0.99 | 5153.6 | 2436.6 | 0.49 | 1.00 | 4962.4 | 235.4 | 0.10 | 0.83 |
| ECs4536 | 4582702 | 4582884 | 183 | 11.4 | 16.9 | 1.53 | 0.49 | 39.5 | 3.9 | 0.10 | 0.63 | 38.9 | 1.7 | 0.04 | 0.63 |
| ECs4587 | 4619553 | 4619771 | 219 | 13.4 | 16.5 | 4.05 | 0.47 | 84.7 | 17.1 | 0.20 | 0.59 | 15.6 | 0.2 | 0.01 | 0.11 |
| ECs4613 | 4643365 | 4643463 | 99 | 677.1 | 1139.7 | 1.71 | 1.00 | 378.2 | 242.0 | 0.61 | 1.00 | 512.4 | 60.4 | 0.32 | 0.78 |
| ECs4638 | 4670569 | 4670709 | 141 | 4628.3 | 19361.2 | 4.17 | 1.00 | 8839.4 | 21331.8 | 2.15 | 1.00 | 4640.5 | 1474.7 | 0.37 | 0.91 |
| ECs4644 | 4677144 | 4677218 | 75 | 506.6 | 541.0 | 1.04 | 1.00 | 206.1 | 13.2 | 0.07 | 0.69 | 26.6 | 2.3 | 0.08 | 0.07 |
| ECs4659 | 4693425 | 4693604 | 180 | 53.7 | 29.6 | 0.54 | 0.59 | 35.3 | 5.0 | 0.14 | 0.49 | 157.5 | 2.5 | 0.03 | 0.23 |
| ECs4701 | 4743766 | 4743864 | 99 | 1093.3 | 360.4 | 0.33 | 1.00 | 1396.7 | 147.9 | 0.11 | 1.00 | 525.4 | 23.2 | 0.05 | 0.57 |
| ECs4715 | 4759637 | 4759738 | 102 | 509.3 | 130.9 | 0.25 | 0.96 | 710.3 | 78.9 | 0.11 | 1.00 | 376.5 | 27.7 | 0.09 | 0.94 |
| ECs4863 | 4928505 | 4928717 | 213 | 2541.8 | 30068.9 | 11.79 | 1.00 | 4766.0 | 23950.0 | 4.56 | 1.00 | 4141.8 | 6801.6 | 1.73 | 0.70 |
| ECs4947 | 5045160 | 5045381 | 222 | 12.4 | 3.8 | 0.35 | 0.26 | 14.6 | 2.3 | 0.28 | 0.37 | 12.4 | 2.0 | 0.12 | 0.20 |
| ECs4958 | 5049833 | 5050030 | 198 | 8.2 | 10.5 | 1.21 | 0.49 | 33.3 | 9.9 | 0.28 | 0.69 | 9.0 | 0.3 | 0.04 | 0.14 |
| ECs4965 | 5052564 | 5052791 | 228 | 7.9 | 19.3 | 2.12 | 0.66 | 24.8 | 5.9 | 0.24 | 0.65 | 6.8 | 1.1 | 0.16 | 0.16 |
| ECs4991 | 5074226 | 5074429 | 204 | 5.9 | 8.0 | 1.42 | 0.23 | 11.6 | 7.4 | 0.72 | 0.54 | 7.3 | 1.6 | 0.22 |  |
| ECs4994 | 5077497 | 5077679 | 183 | 30.8 | 54.1 | 2.32 | 0.71 | 44.7 | 8.9 | 0.20 | 0.75 | 23.1 | 2.3 | 0.11 | 0.16 |
| ECs4997 | 5078578 | 5078778 | 201 | 81.7 | 727.1 | 8.94 | 0.98 | 178.0 | 405.5 | 2.20 | 1.00 | 143.8 | 9.2 | 0.06 | 0.58 |
| ECs5028 | 5112419 | 5112628 | 210 | 119.1 | 52.6 | 0.45 | 0.82 | 79.2 | 34.1 | 0.42 | 0.78 | 105.1 | 2.8 | 0.03 | 0.51 |
| ECs5152 | 5258112 | 5258309 | 198 | 27.0 | 80.9 | 3.60 | 0.75 | 43.1 | 55.0 | 1.32 | 0.87 | 92.2 | 20.9 | 0.20 | 0.81 |
| ECs5178 | 5279565 | 5279792 | 228 | 3527.0 | 7340.4 | 2.08 | 1.00 | 4537.3 | 7174.2 | 1.47 | 1.00 | 3125.4 | 1518.6 | 0.48 | 1.00 |
| ECs5224 | 5327937 | 5328071 | 135 | 181.8 | 212.8 | 1.17 | 0.97 | 96.3 | 31.1 | 0.36 | 0.97 | 48.3 | 9.7 | 0.19 | 0.71 |
| ECs5292 | 5421500 | 5421700 | 201 | 5.3 | 3.2 | 0.64 | 0.14 | 6.8 | 0.8 | 0.12 | 0.13 | 6.3 | 0.0 | 0.00 | 0.06 |
| ECs5303 | 5434891 | 5435055 | 165 | 7.9 | 9.7 | 1.47 | 0.30 | 4.0 | 4.1 | 1.00 | 0.31 | 2.3 | 0.7 | 0.30 | 0.32 |
| ECs5312 | 5446540 | 5446743 | 204 | 34.4 | 62.9 | 1.83 | 0.70 | 48.0 | 28.3 | 0.55 | 0.80 | 28.3 | 2.7 | 0.10 | 0.53 |
| ECs5360 | 5497200 | 5497340 | 141 | 18.8 | 5.9 | 0.32 | 0.22 | 55.9 | 3.5 | 0.08 | 0.10 | 47.4 | 0.3 | 0.01 | 0.55 |
| ECs5363 | 122063 | 122284 | 222 | 6.7 | 3.6 | 0.64 | 0.20 | 7.5 | 0.6 | 0.06 | 0.19 | 0.7 | 0.2 | 0.29 | 0.09 |
| ECs5364 | 168859 | 169065 | 207 | 98.0 | 44.9 | 0.46 | 0.83 | 130.3 | 7.8 | 0.06 | 0.78 | 140.3 | 1.7 | 0.01 | 0.07 |
| ECs5365 | 217269 | 217487 | 219 | 426.1 | 1762.6 | 4.18 | 0.98 | 209.8 | 560.2 | 2.84 | 0.99 | 219.5 | 105.5 | 0.59 | 0.67 |
| ECs5374 | 317658 | 317828 | 171 | 15.0 | 2.7 | 0.25 | 0.10 | 40.3 | 0.9 | 0.02 | 0.18 | 17.8 | 0.6 | 0.05 | 0.54 |
| ECs5375 | 344782 | 344922 | 141 | 17.4 | 133.6 | 7.82 | 0.83 | 17.0 | 82.0 | 4.83 | 0.71 | 22.1 | 8.1 | 0.37 | 0.30 |
| ECs5380 | 665952 | 666173 | 222 | 6.7 | 11.0 | 1.68 | 0.41 | 21.7 | 4.5 | 0.23 | 0.53 | 18.4 | 1.3 | 0.07 | 0.63 |
| ECs5382 | 713651 | 713848 | 198 | 58.6 | 117.7 | 2.06 | 0.84 | 27.9 | 32.3 | 1.08 | 0.72 | 98.7 | 8.3 | 0.16 | 0.74 |
| ECs5391 | 858702 | 858815 | 114 | 1037.1 | 455.6 | 0.44 | 1.00 | 398.1 | 203.1 | 0.51 | 1.00 | 217.6 | 26.9 | 0.12 | 0.53 |
| ECs5406 | 1191964 | 1192170 | 207 | 5.4 | 21.0 | 5.77 | 0.61 | 6.2 | 1.8 | 0.31 | 0.31 | 4.3 | 1.0 | 0.22 | 0.38 |
| ECs5411 | 1319757 | 1319966 | 210 | 8.8 | 12.1 | 1.37 | 0.26 | 15.4 | 36.0 | 4.25 | 0.40 | 18.0 | 0.1 | 0.01 | 0.17 |
| ECs5412 | 1375471 | 1375668 | 198 | 2.1 | 3.6 | 1.79 | 0.39 | 6.3 | 2.6 | 0.31 | 0.23 | 3.8 | 1.0 | 0.26 | 0.18 |
| ECs5414 | 1425231 | 1425428 | 198 | 9.9 | 4.7 | 0.64 | 0.35 | 9.8 | 4.1 | 0.47 | 0.50 | 20.2 | 0.5 | 0.02 | 0.56 |
| ECs5418 | 1566390 | 1566611 | 222 | 11.7 | 4.7 | 0.51 | 0.28 | 6.2 | 0.8 | 0.13 | 0.32 | 4.7 | 0.7 | 0.11 | 0.14 |
| ECs5420 | 1597034 | 1597165 | 132 | 59.1 | 104.6 | 1.82 | 0.65 | 115.4 | 64.8 | 0.55 | 0.66 | 236.1 | 5.1 | 0.03 | 0.40 |
| ECs5422 | 1702104 | 1702307 | 204 | 9.3 | 6.4 | 0.69 | 0.36 | 10.6 | 1.1 | 0.11 | 0.23 | 5.1 | 0.2 | 0.04 | 0.04 |
| ECs5432 | 1845142 | 1845315 | 174 | 114.3 | 46.8 | 0.41 | 0.83 | 116.2 | 20.7 | 0.21 | 0.68 | 145.3 | 9.0 | 0.06 | 0.26 |
| ECs5433 | 1927309 | 1927485 | 177 | 10.2 | 3.0 | 0.25 | 0.19 | 8.1 | 1.7 | 0.15 | 0.13 | 4.6 | 3.4 | 0.81 | 0.55 |
| ECs5438 | 1952162 | 1952383 | 222 | 9.2 | 8.2 | 0.90 | 0.32 | 5.1 | 1.2 | 0.25 | 0.33 | 8.4 | 0.5 | 0.05 | 0.05 |
| ECs5440 | 2009431 | 2009580 | 150 | 278.5 | 81.0 | 0.32 | 0.78 | 59.3 | 5.9 | 0.10 | 0.68 | 29.1 | 2.6 | 0.09 | 0.47 |
| ECs5441 | 2050312 | 2050521 | 210 | 16.8 | 8.7 | 0.55 | 0.17 | 66.3 | 7.1 | 0.14 | 0.63 | 79.3 | 0.5 | 0.02 | 0.45 |
| ECs5442 | 2051029 | 2051208 | 180 | 7.0 | 1.9 | 0.30 | 0.11 | 38.8 | 9.9 | 0.28 | 0.40 | 10.7 | 0.2 | 0.02 | 0.14 |
| ECs5451 | 2228725 | 2228946 | 222 | 18.0 | 8.2 | 0.48 | 0.27 | 5.2 | 0.9 | 0.29 | 0.34 | 11.7 | 0.7 | 0.05 | 0.38 |
| ECs5458 | 2250110 | 2250328 | 219 | 11.9 | 17.4 | 2.05 | 0.55 | 38.5 | 21.2 | 0.65 | 0.69 | 22.0 | 1.7 | 0.08 | 0.37 |
| ECs5460 | 2312013 | 2312213 | 201 | 3.2 | 0.5 | 0.25 | 0.05 | 0.9 | 0.2 | 0.22 | 0.16 | 0.7 | 0.3 | 0.43 | 0.33 |
| ECs5463 | 2510779 | 2510994 | 216 | 33.3 | 15.0 | 0.45 | 0.59 | 34.2 | 18.1 | 0.50 | 0.83 | 73.0 | 9.7 | 0.13 | 0.31 |
| ECs5465 | 2595658 | 2595864 | 207 | 36.4 | 56.1 | 1.66 | 0.59 | 114.3 | 15.0 | 0.12 | 0.72 | 40.0 | 1.5 | 0.06 | 0.65 |
| ECs5482 | 2942020 | 2942154 | 135 | 118.6 | 38.0 | 0.32 | 0.83 | 51.9 | 15.8 | 0.38 | 0.77 | 47.2 | 3.7 | 0.08 | 0.39 |
| ECs5484 | 3043126 | 3043344 | 219 | 4.0 | 3.0 | 1.39 | 0.13 | 3.0 | 1.3 | 0.35 | 0.29 | 0.0 | 0.6 | 0.00 | 0.57 |
| ECs5495 | 3252320 | 3252538 | 219 | 19.8 | 73.4 | 5.56 | 0.74 | 35.7 | 8.6 | 0.23 | 0.56 | 40.0 | 9.0 | 0.20 | 0.24 |
| ECs5496 | 3308886 | 3309086 | 201 | 554.1 | 749.8 | 1.39 | 1.00 | 432.6 | 328.2 | 0.75 | 0.99 | 342.7 | 22.4 | 0.08 | 0.69 |
| ECs5498 | 3396071 | 3396295 | 225 | 10.3 | 2.3 | 0.27 | 0.25 | 6.3 | 0.8 | 0.15 | 0.34 | 0.7 | 0.1 | 0.14 | 0.03 |
| ECs5530 | 4173443 | 4173661 | 219 | 1409.7 | 4224.4 | 3.03 | 0.88 | 1024.2 | 691.0 | 0.62 | 0.91 | 468.0 | 8.4 | 0.02 | 0.88 |
| ECs5531 | 4189674 | 4189868 | 195 | 20.7 | 34.8 | 1.50 | 0.54 | 89.0 | 12.3 | 0.15 | 0.53 | 748.5 | 100.9 | 0.18 | 0.82 |
| ECs5532 | 4198498 | 4198698 | 201 | 62.3 | 52.1 | 0.84 | 0.89 | 71.1 | 13.4 | 0.19 | 0.86 | 70.0 | 3.2 | 0.05 | 0.70 |
| ECs5556 | 4788180 | 4788383 | 204 | 493.9 | 379.7 | 0.86 | 0.96 | 210.9 | 129.3 | 0.68 | 0.93 | 155.5 | 37.1 | 0.23 | 0.57 |
| ECs5575 | 5001462 | 5001662 | 201 | 7.4 | 17.7 | 2.83 | 0.49 | 7.1 | 4.9 | 0.82 | 0.49 | 5.5 | 8.6 | 1.80 | 0.45 |
| ECs5580 | 5049284 | 5049469 | 186 | 4.8 | 2.3 | 0.68 | 0.17 | 7.8 | 1.7 | 0.22 | 0.14 | 2.8 | 0.0 | 0.00 | 0.16 |
| ECs5586 | 5230834 | 5230980 | 147 | 83.7 | 21.0 | 0.26 | 0.82 | 90.0 | 37.6 | 0.40 | 0.83 | 46.8 | 1.9 | 0.07 | 0.67 |
| ECs5591 | 5358407 | 5358574 | 168 | 31.4 | 10.5 | 0.40 | 0.29 | 44.0 | 4.3 | 0.10 | 0.40 | 20.3 | 0.6 | 0.04 | 0.52 |
| ECs5595 | 5476071 | 5476202 | 132 | 16.2 | 102.0 | 6.03 | 0.99 | 25.1 | 48.2 | 2.20 | 0.88 | 10.1 | 3.7 | 0.38 | 0.21 |
