# Supplementary material for: Discovery of numerous novel small genes in the intergenic regions of the Escherichia coli O157:H7 Sakai genome
Source: PLoS One. 2017 Sep 13;12(9):e0184119. doi: 10.1371/journal.pone.0184119 (PMC5597208; doi:10.1371/journal.pone.0184119)
Supplement: S3 Table — Annotated homologs in other strains/species were searched using blastp. Only the best hit is listed. The fourth column illustrates annotated homologs in other E. coli O157:H7 strains or duplications of annotated genes in EHEC Sakai. With bioinformatics methods the presence of a σ70 promoter, a ρ-independent terminator, a Shine-Dalgarno sequence, and selection pressure (kA/kS) were predicted or estimated. The last column gives the classification of the putative novel protein by the machine-learning algorithm trained with short annotated E. coli O157:H7 EDL933 genes. (DOCX) [file pone.0184119.s005.docx]

|  | BLASTP | | | | | K_A_/K_s_ | | | promoter | | | Shine Dalgarno Sequence | | terminator | |  |
| --- | --- | --- | --- | --- | --- | --- | --- | --- | --- | --- | --- | --- | --- | --- | --- | --- |
| gene name | e-value | organism | | hit in EHEC | | value | p-value | | bps upstream start codon | | LDF score | bps upstream start codon | ∆G° | bps downstream stop codon | score | machine learning algorithm |
| XECs001 | 4E-07 | *Escherichia coli* |  | | - | - | | - | 188 | 3.32 | | - | - | - | - | real |
| XECs002 | 1E-04 | *Escherichia coli* |  | | - | - | | - | 45 | 3.39 | | - | - | - | - | real |
| XECs003 | 3E-33 | *Shigella sonnei* |  | | - | - | | - | 219 | 3.43 | | - | - | - | - | real |
| XECs004 | - | - |  | | - | - | | - | 202 | 2.37 | | - | - | - | - | real |
| XECs005 | - | - |  | | - | - | | - | 43 | 3.01 | | 16 | -3.3 | - | - | pseudo |
| XECs006 | 6E-21 | *Escherichia coli* |  | | - | - | | - | 115 | 4.83 | | 14 | -5.1 | - | - | real |
| XECs007 | 4E-11 | *Salmonella enterica* |  | | - | - | | - | 90 | 2.73 | | - | - | - | - | real |
| XECs008 | 1E-17 | *Escherichia coli* |  | | - | - | | - | 34 | 5.76 | | - | - | - | - | real |
| XECs009 | - | - |  | | - | - | | - | 36 | 1.83 | | - | - | - | - | pseudo |
| XECs010 | - | *-* |  | | - | - | | - | 40 | 4.33 | | - | - | - | - | pseudo |
| XECs011 | 1E-04 | *Escherichia coli* |  | | - | - | | - | 143 | 3.5 | | 9 | -6.1 | - | - | real |
| XECs012 | - | *-* |  | | - | - | | - | 214 | 1.88 | | 2 | -4.2 | - | - | pseudo |
| XECs013 | 7E-08 | *Salmonella enterica* |  | | - | - | | - | 42 | 1.53 | | - | - | 38 | -20.4 | pseudo |
| XECs014 | 7E-04 | *Salmonella enterica* |  | | - | - | | - | 156 | 1.53 | | 8 | -8.7 | - | - | real |
| XECs015 | 2E-06 | *Salmonella enterica* |  | | - | - | | - | 154 | 1.7 | | 11 | -5 | - | - | pseudo |
| XECs016 | 6E-13 | *Escherichia coli* |  | | - | - | | - | 203 | 4.35 | | 15 | -3.4 | - | - | real |
| XECs017 | - | - |  | | - | - | | - | 34 | 7.94 | | 21 | -3.5 | - | - | pseudo |
| XECs018 | 0 | *Escherichia coli* |  | | - | - | | - | 77 | 7.94 | | 5 | -5.9 | - | - | real |
| XECs019 | 2E-06 | *Escherichia coli* |  | | - | - | | - | 84 | 7.94 | | 12 | -5.9 | - | - | real |
| XECs020 | - | - |  | | - | - | | - | 231 | 7.94 | | - | - | - | - | pseudo |
| XECs021 | - | *-* |  | | - | - | | - | 214 | 1.98 | | - | - | - | - | pseudo |
| XECs022 | - | - |  | | - | - | | - | 221 | 1.98 | | - | - | - | - | pseudo |
| XECs023 | - | *-* |  | | - | - | | - | 53 | 2.99 | | 17 | -4.2 | - | - | pseudo |
| XECs024 | 6E-15 | *Escherichia coli* |  | | - | - | | - | 147 | 3.97 | | 12 | -3.3 | - | - | real |
| XECs025 | 2E-16 | *Escherichia coli* |  | | - | - | | - | 246 | 2.8 | | - | - | - | - | real |
| XECs026 | - | *-* |  | | - | - | | - | 111 | 3.07 | | - | - | - | - | pseudo |
| XECs027 | 7E-10 | *Escherichia coli* |  | | - | - | | - | 162 | 4.25 | | - | - | - | - | real |
| XECs028 | 3E-05 | *Escherichia coli* |  | | hit in EDL933 | - | | - | 50 | 2.6 | | 14 | -3.8 | - | - | real |
| XECs029 | 4E-172 | *Escherichia coli* |  | | annotated in EDL933 as Z0405 | - | | - | 90 | 2.59 | | - | - | - | - | real |
| XECs030 | - | - |  | | - | - | | - | 197 | 3.04 | | - | - | - | - | pseudo |
| XECs031 | 3E-07 | *Escherichia coli* |  | | - | 0.144 | | 2E-04 | 227 | 3.24 | | - | - | 74 | -13.3 | real |
| XECs032 | 5E-32 | *Escherichia coli* |  | | - | - | | - | 128 | 3.68 | | 16 | -3.4 | - | - | real |
| XECs033 | 1E-40 | *Escherichia coli* |  | | - | - | | - | 195 | 3.68 | | - | - | - | - | real |
| XECs034 | - | - |  | | - | - | | - | 140 | 2.24 | | 3 | -4.8 | - | - | pseudo |
| XECs035 | 5E-20 | *Escherichia coli* |  | | - | - | | - | 91 | 3.07 | | 4 | -2.9 | 244 | -14.1 | real |
| XECs036 | 3E-11 | *Escherichia coli* |  | | - | - | | - | 119 | 2.17 | | 8 | -3.2 | - | - | real |
| XECs037 | - | - |  | | - | - | | - | 172 | 2.19 | | 10 | -4.1 | - | - | pseudo |
| XECs038 | - | - |  | | - | - | | - | 77 | 3.9 | | - | - | 125 | -13.4 | pseudo |
| XECs039 | - | - |  | | - | - | | - | 42 | 5.27 | | 2 | -3.5 | 82 | -13.4 | pseudo |
| XECs040 | - | - |  | | - | - | | - | 31 | 4.73 | | 19 | -4.1 | - | - | pseudo |
| XECs041 | - | - |  | | - | - | | - | 83 | 4.63 | | - | - | - | - | pseudo |
| XECs042 | - | - |  | | - | - | | - | 245 | 1.41 | | - | - | - | - | pseudo |
| XECs043 | 4E-21 | *Escherichia coli* |  | | - | - | | - | 202 | 3.98 | | 0 | -4.9 | - | - | real |
| XECs044 | - | - |  | | - | - | | - | 43 | 3.21 | | - | - | - | - | pseudo |
| XECs045 | - | *-* |  | | - | - | | - | 111 | 3.61 | | 8 | -3.2 | - | - | pseudo |
| XECs046 | 7E-57 | *Escherichia coli* |  | | - | - | | - | 130 | 1.75 | | 0 | -4 | - | - | real |
| XECs047 | - | - |  | | - | - | | - | 173 | 3.05 | | - | - | - | - | pseudo |
| XECs048 | - | - |  | | - | - | | - | 240 | 2.26 | | 19 | -4.9 | 144 | -14.2 | pseudo |
| XECs049 | 6E-06 | *Salmonella enterica* |  | | - | - | | - | 247 | 2.26 | | - | - | 157 | -14.2 | real |
| XECs050 | 6E-10 | *Escherichia coli* |  | | - | - | | - | 232 | 2.71 | | - | - | - | - | real |
| XECs051 | - | - |  | | - | - | | - | 98 | 1.17 | | - | - | - | - | pseudo |
| XECs052 | - | - |  | | - | - | | - | 106 | 1.07 | | - | - | - | - | pseudo |
| XECs053 | 5E-13 | *Escherichia albertii* |  | | - | - | | - | - | - | | 13 | -6.7 | - | - | real |
| XECs054 | 3E-09 | *Shigella sonnei* |  | | - | - | | - | 186 | 3.06 | | - | - | - | - | real |
| XECs055 | - | - |  | | - | - | | - | 148 | 3.57 | | - | - | - | - | pseudo |
| XECs056 | - | - |  | | - | - | | - | 51 | 6.66 | | - | - | - | - | pseudo |
| XECs057 | - | - |  | | - | - | | - | 74 | 6.66 | | - | - | - | - | pseudo |
| XECs058 | 9E-34 | *Escherichia coli* |  | | - | - | | - | 56 | 2.81 | | - | - | - | - | real |
| XECs059 | 1E-25 | *Escherichia coli* |  | | is X005 in EDL 933 (Neuhaus et al., 2016) | - | | - | 247 | 3.37 | | 0 | -9.6 | - | - | real |
| XECs060 | - | - |  | | - | - | | - | 88 | 2.74 | | 21 | -6.2 | - | - | pseudo |
| XECs061 | - | - |  | | - | - | | - | 77 | 3.24 | | 5 | -5.3 | - | - | pseudo |
| XECs062 | 3E-08 | *Salmonella enterica* |  | | - | - | | - | 28 | 4.01 | | - | - | 232 | -15.7 | real |
| XECs063 | 4E-09 | *Citrobacter* |  | | - | - | | - | 97 | 2.58 | | 12 | -4.2 | - | - | real |
| XECs064 | 2E-14 | *Shigella sonnei* |  | | - | - | | - | 60 | 3.87 | | - | - | - | - | real |
| XECs065 | 6E-23 | *Escherichia coli* |  | | - | - | | - | - | - | | - | - | - | - | real |
| XECs066 | 1E-21 | *Escherichia coli* |  | | - | - | | - | 33 | 5.42 | | - | - | - | - | real |
| XECs067 | 6E-15 | *Citrobacter koseri* |  | | - | - | | - | 213 | 2.96 | | - | - | - | - | real |
| XECs068 | - | - |  | | - | 0.018 | | 2E-04 | 233 | 2.96 | | - | - | - | - | pseudo |
| XECs069 | - | - |  | | - | - | | - | 98 | 5.45 | | 18 | -8.3 | - | - | pseudo |
| XECs070 | 1E-14 | *Escherichia coli* |  | | - | - | | - | 231 | 6.12 | | 5 | -3.6 | - | - | real |
| XECs071 | - | - |  | | - | - | | - | 160 | 4.55 | | 18 | -3.5 | - | - | pseudo |
| XECs072 | - | - |  | | - | - | | - | 164 | 4.55 | | - | - | - | - | pseudo |
| XECs073 | - | - |  | | - | - | | - | 230 | 4.74 | | - | - | - | - | pseudo |
| XECs074 | - | - |  | | - | - | | - | 99 | 5.54 | | 19 | -5.1 | - | - | pseudo |
| XECs075 | - | - |  | | - | - | | - | 191 | 7.85 | | - | - | - | - | pseudo |
| XECs076 | - | *-* |  | | - | - | | - | 144 | 3.47 | | 2 | -4.9 | - | - | real |
| XECs077 | - | - |  | | - | - | | - | 219 | 2.41 | | - | - | - | - | pseudo |
| XECs078 | - | - |  | | - | - | | - | 238 | 2.19 | | 0 | -3.4 | - | - | pseudo |
| XECs079 | 3E-07 | *Escherichia coli* |  | | - | - | | - | 183 | 3.13 | | 8 | -2,9 | - | - | pseudo |
| XECs080 | 4E-05 | *Salmonella enterica* |  | | - | - | | - | 200 | 4.04 | | - | - | - | - | real |
| XECs081 | - | - |  | | - | 0.33 | | 2E-04 | 157 | 4.04 | | - | - | - | - | pseudo |
| XECs082 | 6E-21 | *Escherichia coli* |  | | - | - | | - | 45 | 4.04 | | - | - | - | - | real |
| XECs083 | 2E-05 | *Escherichia coli* |  | | - | - | | - | 106 | 1.31 | | 6 | -5.7 | - | - | real |
| XECs084 | - | - |  | | - | - | | - | 93 | 2.14 | | 13 | -4.2 | - | - | pseudo |
| XECs085 | - | - |  | | - | - | | - | 147 | 4.18 | | - | - | - | - | pseudo |
| XECs086 | - | - |  | | - | - | | - | 132 | 2.24 | | 21 | -3.5 | - | - | pseudo |
| XECs087 | 8E-13 | *Escherichia coli* |  | | - | - | | - | 247 | 2.6 | | - | - | - | - | real |
| XECs088 | 3E-42 | *Escherichia coli* |  | | - | - | | - | 48 | 1.68 | | - | - | - | - | real |
| XECs089 | 0.001 | *Burkholderia mallei* |  | | - | - | | - | 178 | 2.72 | | 13 | -4.9 | - | - | real |
| XECs090 | 2E-06 | *Escherichia coli* |  | | - | - | | - | 68 | 3.69 | | - | - | - | - | pseudo |
| XECs091 | 5E-04 | *Salmonella enterica* |  | | is X011 in EDL 933 (Neuhaus et al., 2016) | - | | - | 186 | 2.86 | | - | - | - | - | real |
| XECs092 | - | *-* |  | | - | - | | - | 73 | 4.38 | | - | - | - | - | pseudo |
| XECs093 | - | - |  | | - | - | | - | 55 | 2.55 | | - | - | - | - | pseudo |
| XECs094 | - | - |  | | - | - | | - | 30 | 2.67 | | 13 | -4 | - | - | pseudo |
| XECs095 | 1E-32 | *Escherichia coli* |  | | - | - | | - | 31 | 6.02 | | - | - | - | - | real |
| XECs096 | 3E-25 | *Escherichia coli* |  | | - | - | | - | 88 | 1.87 | | - | - | - | - | real |
| XECs097 | - | - |  | | - | - | | - | 187 | 1.19 | | - | - | - | - | pseudo |
| XECs098 | 4E-40 | *Escherichia coli* |  | | - | - | | - | 134 | 3.25 | | 21 | -3.7 | - | - | real |
| XECs099 | - | - |  | | - | - | | - | 40 | 7.14 | | - | - | - | - | pseudo |
| XECs100 | 4E-40 | *Escherichia coli* |  | | - | - | | - | 94 | 3.41 | | 4 | -3.8 | 129 | -14.5 | real |
| XECs101 | 4E-25 | *Escherichia coli* |  | | - | - | | - | 29 | 1.06 | | 18 | -3.3 | - | - | real |
| XECs102 | 3E-21 | *Escherichia coli* phage |  | | - | - | | - | - | - | | - | - | 65 | -18.6 | real |
| XECs103 | 3E-34 | *Escherichia coli* |  | | - | - | | - | 247 | 6.34 | | - | - | - | - | real |
| XECs104 | 4E-69 | *Escherichia coli* |  | | annotated as transcriptional regulator in EHEC | - | | - | 85 | 6.99 | | 5 | -5.5 | 42 | -11.8 | real |
| XECs105 | 2E-08 | *Escherichia coli* |  | | - | - | | - | 43 | 6.99 | | 20 | -3.7 | - | - | real |
| XECs106 | 1E-04 | *Escherichia coli* |  | | - | - | | - | 135 | 3.78 | | 13 | -3.8 | - | - | real |
| XECs107 | 5E-11 | *Escherichia coli* |  | | - | - | | - | 96 | 3.89 | | - | - | - | - | real |
| XECs108 | 3E-12 | *Escherichia coli* |  | | - | - | | - | 28 | 2.12 | | - | - | - | - | real |
| XECs109 | - | - |  | | - | - | | - | 49 | 7.39 | | 1 | -3.3 | 121 | -13 | pseudo |
| XECs110 | - | - |  | | - | - | | - | 62 | 7.39 | | 11 | -4.8 | 129 | -13 | pseudo |
| XECs111 | - | - |  | | - | - | | - | 62 | 6.99 | | 16 | -5.2 | - | - | pseudo |
| XECs112 | - | - |  | | - | - | | - | 116 | 3.2 | | - | - | - | - | pseudo |
| XECs113 | 6E-22 | *Escherichia coli* |  | | - | 1E-06 | | 1E-06 | 167 | 7.85 | | - | - | - | - | real |
| XECs114 | 6E-12 | *Escherichia coli* |  | | - | - | |  | 115 | 3.83 | | 0 | -3.6 | - | - | pseudo |
| XECs115 | - | - |  | | - | - | | - | 208 | 3.25 | | - | - | 28 | -14.8 | pseudo |
| XECs116 | - | - |  | | - | - | | - | 32 | 5.18 | | - | - | 121 | -21.1 | pseudo |
| XECs117 | 3E-30 | *Escherichia coli* |  | | annotated in EDL933 as Z2553 | - | | - | 105 | 4.68 | | - | - | 56 | -17.3 | real |
| XECs118 | 6E-18 | *Escherichia coli* |  | | annotated as hypothetical protein in other EHEC strain | - | | - | 101 | 6.02 | | 7 | -3.5 | - | - | real |
| XECs119 | 5E-19 | *Escherichia coli* |  | | - | - | | - | 160 | 2.63 | | 6 | -6.2 | - | - | real |
| XECs120 | - | - |  | | - | - | | - | 144 | 2.63 | | - | - | - | - | pseudo |
| XECs121 | - | - |  | | - | - | | - | 133 | 3.1 | | - | - | 247 | -20.5 | pseudo |
| XECs122 | - | - |  | | - | - | | - | 201 | 3.1 | | - | - | 206 | -20.5 | pseudo |
| XECs123 | - | - |  | | - | - | | - | 67 | 2.27 | | - | - | - | - | pseudo |
| XECs124 | - | - |  | | - | - | | - | 38 | 2.17 | | - | - | - | - | pseudo |
| XECs125 | 2E-74 | *Escherichia coli* |  | | - | - | | - | 189 | 3.44 | | - | - | - | - | real |
| XECs126 | 5E-34 | *Escherichia coli* |  | | - | - | | - | 91 | 3.44 | | 14 | -3.6 | - | - | real |
| XECs127 | 6E-10 | *Escherichia coli* |  | | - | - | | - | 128 | 1.42 | | 21 | -3.5 | - | - | real |
| XECs128 | 7E-67 | *Escherichia coli* |  | | annotated as tellurium resistance protein in other EHEC strain | - | | - | 88 | 1.51 | | - | - | - | - | real |
| XECs129 | - | - |  | | - | - | | - | 54 | 6.23 | | 3 | -3.6 | - | - | pseudo |
| XECs130 | 2E-26 | *Escherichia coli* |  | | annotated as tellurium resistance protein in other EHEC strain | - | | - | 186 | 4.96 | | 3 | -6 | - | - | real |
| XECs131 | 2E-15 | *Escherichia coli* |  | | - | - | | - | 244 | 1.62 | | - | - | - | - | real |
| XECs132 | 5E-06 | *Escherichia coli* |  | | - | - | | - | 54 | 5.24 | | - | - | - | - | real |
| XECs133 | 1E-29 | *Escherichia coli* |  | | annotated as hypothetical protein in other EHEC strain | - | | - | 109 | 5.65 | | 2 | -3.4 | - | - | real |
| XECs134 | 1E-26 | *Escherichia coli* |  | | - | - | | - | 125 | 2.5 | | - | - | - | - | real |
| XECs135 | - | - |  | | - | - | | - | 31 | 5.9 | | - | - | - | - | pseudo |
| XECs136 | - | - |  | | - | - | | - | 227 | 6.89 | | - | - | - | - | pseudo |
| XECs137 | - | - |  | | - | - | | - | 35 | 6.06 | | - | - | - | - | pseudo |
| XECs138 | 1E-29 | *Escherichia coli* |  | | annotated in EDL933 as Z1206 | - | | - | 105 | 6.06 | | 3 | -6.5 | - | - | real |
| XECs139 | - | *-* |  | | - | - | | - | 70 | 6.36 | | 11 | -4.7 | - | - | pseudo |
| XECs140 | 2E-23 | *Escherichia coli* |  | | - | - | | - | 37 | 2.47 | | - | - | - | - | real |
| XECs141 | - | - |  | | - | - | | - | 78 | 1.57 | | - | - | - | - | pseudo |
| XECs142 | 1E-10 | *Escherichia coli* |  | | - | - | | - | 245 | 1.18 | | - | - | - | - | real |
| XECs143 | - | - |  | | - | - | | - | 62 | 3.57 | | - | - | - | - | pseudo |
| XECs144 | - | - |  | | - | - | | - | 197 | 1.34 | | 17 | -3.2 | - | - | pseudo |
| XECs145 | 2E-28 | *Escherichia coli* |  | | annotated as hypothetical protein in other EHEC strain | - | | - | 173 | 2.13 | | - | - | - | - | real |
| XECs146 | 4E-28 | *Escherichia coli* |  | | - | - | | - | 245 | 4.83 | | 2 | -4.1 | - | - | real |
| XECs147 | 3E-28 | *Escherichia coli* |  | | - | - | | - | 205 | 8 | | - | - | - | - | real |
| XECs148 | - | - |  | | - | - | | . | 231 | 8 | | - | - | - | - | pseudo |
| XECs149 | 2E-24 | *Escherichia coli* |  | | annotated as hypothetical protein in other EHEC strain | - | | - | 197 | 2.21 | | - | - | - | - | real |
| XECs150 | - | - |  | | - | - | | - | 215 | 2.6 | | - | - | - | - | pseudo |
| XECs151 | 5E-37 | *Escherichia coli* |  | | - | 0.061 | | 2E-04 | 204 | 7.61 | | 1 | -8.7 | 141 | -19.2 | real |
| XECs152 | - | - |  | | - | - | | - | 234 | 6.84 | | - | - | 87 | -19.2 | pseudo |
| XECs153 | - | - |  | | - | - | | - | 31 | 5.22 | | - | - | 35 | -14.9 | pseudo |
| XECs154 | 1E-17 | *Escherichia coli* |  | | - | - | | - | 89 | 4.58 | | 6 | -3.1 | - | - | real |
| XECs155 | 2E-20 | *Escherichia coli* |  | | - | - | | - | 30 | 2.86 | | 2 | -4.3 | - | - | real |
| XECs156 | - | - |  | | - | - | | - | 144 | 2.81 | | - | - | - | - | pseudo |
| XECs157 | - | *-* |  | | - | - | | - | 207 | 1.77 | | - | - | - | - | pseudo |
| XECs158 | - | - |  | | - | 0.093 | | 2E-04 | 180 | 2.71 | | - | - | - | - | pseudo |
| XECs159 | - | - |  | | - | - | | - | 205 | 2.71 | | - | - | - | - | pseudo |
| XECs160 | - | - |  | | - | - | | - | 179 | 5.56 | | 14 | -4.7 | - | - | pseudo |
| XECs161 | - | - |  | | - | - | | - | 140 | 2.63 | | 3 | -3.1 | - | - | real |
| XECs162 | - | - |  | | - | - | | - | 50 | 2.42 | | - | - | - | - | pseudo |
| XECs163 | 2E-38 | *Salmonella enterica* |  | | - | - | | - | 181 | 5.14 | | - | - | - | - | real |
| XECs164 | 3E-04 | *Shigella sonnei* |  | | - | - | | - | 104 | 7.52 | | 5 | -4.3 | - | - | pseudo |
| XECs165 | - | - |  | | - | - | | - | 128 | 2.83 | | 18 | -3.3 | - | - | pseudo |
| XECs166 | 9E-58 | *Escherichia coli* |  | | partial hit to annotated protein ECs1079 | - | | - | 56 | 2.8 | | 6 | -4.7 | - | - | real |
| XECs167 | 4E-10 | *Escherichia coli* |  | | - | - | | - | 180 | 2.8 | | 16 | -4.9 | 62 | -13.3 | pseudo |
| XECs168 | - | *-* |  | | - | - | | - | 194 | 4.51 | | 8 | -3.3 | 209 | -13.7 | pseudo |
| XECs169 | - | - |  | | - | - | | - | 208 | 4.51 | | - | - | 252 | -13.7 | pseudo |
| XECs170 | 3E-08 | *Escherichia coli* |  | | - | - | | - | 192 | 4.52 | | - | - | - | - | real |
| XECs171 | 8E-12 | *Escherichia coli* |  | | - | - | | - | 79 | 5.5 | | - | - | - | - | real |
| XECs172 | - | - |  | | - | - | | - | 189 | 5.08 | | 20 | -4.2 | - | - | pseudo |
| XECs173 | - | - |  | | - | - | | - | 193 | 5.08 | | 21 | -3 | - | - | pseudo |
| XECs174 | 2E-76 | *Shigella sonnei* |  | | - | - | | - | 148 | 3.65 | | - | - | - | - | pseudo |
| XECs175 | - | - |  | | - | - | | - | 248 | 3.25 | | - | - | - | - | pseudo |
| XECs176 | 6E-12 | *Escherichia coli* |  | | - | - | | - | 39 | 2.86 | | - | - | - | - | real |
| XECs177 | 0.001 | *Escherichia coli* |  | | - | - | | - | 148 | 3.63 | | - | - | - | - | real |
| XECs178 | 1E-19 | *Escherichia coli* |  | | - | - | | - | 29 | 4.76 | | 12 | -3.3 | 6 | -18.5 | real |
| XECs179 | - | - |  | | - | - | | - | 27 | 6.07 | | 1 | -4.8 | - | - | pseudo |
| XECs180 | - | - |  | | - | - | | - | 33 | 3.75 | | 19 | -2.9 | - | - | pseudo |
| XECs181 | 2E-29 | *Escherichia coli* |  | | annotated in EDL933 as Z2534 | 1E-06 | | 1E-06 | 208 | 4.61 | | - | - | - | - | real |
| XECs182 | 4E-06 | *Shigella dysenteriae* |  | | - | - | | - | 196 | 4.76 | | 7 | -3.7 | - | - | real |
| XECs183 | 4E-12 | *Escherichia coli* |  | | - | 1E-06 | | 1E-06 | 84 | 4.81 | | 5 | -6.4 | - | - | pseudo |
| XECs184 | - | - |  | | - | - | | - | 33 | 3.07 | | - | - | - | - | pseudo |
| XECs185 | 2E-04 | *Escherichia coli* |  | | - | - | | - | 32 | 3.1 | | - | - | 216 | -13.1 | pseudo |
| XECs186 | 5E-31 | *Shigella sonnei* |  | | - | - | | - | 215 | 3.32 | | 6 | -4.6 | - | - | real |
| XECs187 | 1E-27 | *Shigella flexneri* |  | | - | 1E-06 | | 1E-06 | 32 | 4.3 | | 1 | -5.9 | 71 | -13.6 | real |
| XECs188a | 9E-29 | *Escherichia coli* |  | | partial hit to annotated protein ECs2266; duplicate of XECs188b | - | | - | 214 | 5.19 | | 8 | -3.3 | - | - | real |
| XECs188b | 6E-28 | *Escherichia coli* |  | | partial hit to annotated protein ECs2266; duplicate of XECs188a | - | | - | 215 | 5.47 | | 8 | -3.3 | - | - | real |
| XECs189 | - | - |  | | - | - | | - | 228 | 5.19 | | - | - | - | - | pseudo |
| XECs190 | - | - |  | | - | - | | - | 240 | 2.79 | | 0 | -4.0 | - | - | pseudo |
| XECs191 | 4E-22 | *Escherichia coli* |  | | annotated as hypothetical protein in other EHEC strain | - | | - | 120 | 1.7 | | 0 | -7.2 | - | - | real |
| XECs192 | 2E-17 | *Escherichia coli* |  | | annotated as hypothetical protein in other EHEC strain | - | | - | 133 | 3.52 | | - | - | 264 | -16 | real |
| XECs193 | 1E-06 | *Escherichia coli* |  | | - | - | | - | 162 | 3.52 | | 18 | -3.1 | 248 | -16 | real |
| XECs194 | 3E-18 | *Escherichia coli* |  | | - | - | | - | 207 | 3.67 | | - | - | - | - | real |
| XECs195 | - | - |  | | - | - | | - | 117 | 3.1 | | - | - | - | - | pseudo |
| XECs196 | - | - |  | | - | - | | - | 35 | 4.15 | | - | - | - | - | pseudo |
| XECs197 | 1E-45 | *Escherichia coli* |  | | is X027 in EDL 933 (Neuhaus et al., 2016) | - | | - | 96 | 3.52 | | 6 | -3.7 | - | - | real |
| XECs198 | - | - |  | | - | - | | - | 122 | 2.9 | | - | - | - | - | pseudo |
| XECs199 | 5E-12 | *Escherichia coli* |  | | - | - | | - | 149 | 5.84 | | 2 | -6.3 | 75 | -15.7 | real |
| XECs200 | 7E-11 | *Escherichia coli* |  | | - | - | | - | 247 | 5.84 | | 4 | -6.1 | - | - | real |
| XECs201 | 2E-05 | *Escherichia coli* |  | | - | - | | - | 83 | 3.01 | | - | - | - | - | real |
| XECs202 | 2E-22 | *Escherichia coli* |  | | - | - | | - | 35 | 1.46 | | - | - | - | - | real |
| XECs203a | 7E-05 | *Escherichia coli* |  | | duplicate of XECs203b | - | | - | 166 | 3.77 | | - | - | - | - | pseudo |
| XECs203b | 7E-05 | *Escherichia coli* |  | | duplicate of XECs203a | - | | - | 83 | 0.61 | | 13 | -4.4 | - | - | pseudo |
| XECs204 | 5E-60 | *Escherichia coli* |  | | annotated in EDL933 as Z3307 | 0.206 | | 1E-04 | - | - | | 18 | -5.1 | - | - | real |
| XECs205 | 9E-28 | *Escherichia coli* |  | | - | - | | - | - | - | | 18 | -2.9 | 204 | -16 | real |
| XECs206 | - | - |  | | - | - | | - | 229 | 5.47 | | - | - | - | - | pseudo |
| XECs207 | 1E-05 | *Escherichia coli* |  | | - | - | | - | 34 | 5.22 | | - | - | 139 | -18.9 | real |
| XECs208 | - | - |  | | - | - | | - | 192 | 2.62 | | 0 | -3 | - | - | pseudo |
| XECs209 | 4E-54 | *Escherichia coli* |  | | - | - | | - | - | - | | - | - | - | - | real |
| XECs210 | 9E-12 | *Escherichia coli* |  | | - | - | | - | 29 | 1.66 | | - | - | 57 | -16.4 | real |
| XECs211 | 1E-11 | *Escherichia coli* |  | | - | - | | - | 104 | 2.38 | | - | - | 160 | -22 | real |
| XECs212 | 8E-17 | *Escherichia coli* |  | | annotated in EDL933 as Z2105 | - | | - | 225 | 4.98 | | - | - | - | - | real |
| XECs213 | 2E-14 | *Escherichia coli* |  | | - | - | | - | 233 | 4.98 | | - | - | - | - | pseudo |
| XECs214 | - | - |  | | - | - | | - | 58 | 1.4 | | - | - | - | - | pseudo |
| XECs215 | - | - |  | | - | 0.041 | | 2E-04 | 213 | 2.18 | | - | - | - | - | pseudo |
| XECs216 | 6E-32 | *Escherichia coli* |  | | - | - | | - | 101 | 2.57 | | 0 | -3,7 | - | - | real |
| XECs217 | - | - |  | | - | 0.178 | | 2E-04 | 116 | 4.18 | | - | - | 13 | -14.9 | pseudo |
| XECs218 | 3E-17 | *Escherichia coli* |  | | - | 0.053 | | 2E-04 | 67 | 3.75 | | - | - | - | - | real |
| XECs219 | 4E-47 | *Escherichia coli* |  | | - | - | | - | 226 | 4.15 | | - | - | 52 | -12.4 | real |
| XECs220 | - | - |  | | - | - | | - | 186 | 4.11 | | - | - | - | - | pseudo |
| XECs221 | 2E-23 | *Shigella sonnei* |  | | - | - | | - | 173 | 2.12 | | 6 | -6 | - | - | pseudo |
| XECs222 | - | - |  | | - | - | | - | 196 | 2.12 | | - | - | - | - | pseudo |
| XECs223 | 7E-21 | *Shigella flexneri* |  | | - | - | | - | 43 | 3.08 | | 11 | -3.7 | - | - | real |
| XECs224 | - | - |  | | - | - | | - | 183 | 5.82 | | - | - | - | - | pseudo |
| XECs225 | - | - |  | | - | - | | - | 160 | 8.05 | | - | - | - | - | pseudo |
| XECs226 | - | - |  | | - | - | | - | 81 | 4.56 | | 9 | -4.8 | - | - | pseudo |
| XECs227 | 5E-20 | *Escherichia coli* |  | | - | - | | - | 144 | 2.82 | | - | - | 83 | -18.3 | real |
| XECs228 | 2E-18 | *Escherichia coli* |  | | - | 0.079 | | 1E-04 | 34 | 3.81 | | - | - | 7 | -18.3 | real |
| XECs229 | 6E-13 | *Escherichia coli* |  | | - | - | | - | 77 | 1.22 | | - | - | - | - | real |
| XECs230 | - | - |  | | - | - | | - | 128 | 9.35 | | 4 | -4.8 | 251 | -17.6 | pseudo |
| XECs231 | - | - |  | | - | - | | - | 135 | 1.8 | | - | - | - | - | pseudo |
| XECs232 | 5E-21 | *Escherichia coli* |  | | - | - | | - | 93 | 4.58 | | - | - | - | - | real |
| XECs233 | 3E-13 | *Escherichia coli* |  | | - | - | | - | 36 | 3.9 | | - | - | 73 | -18.6 | real |
| XECs234 | - | - |  | | - | - | | - | 112 | 3.9 | | 1 | -3,6 | - | - | pseudo |
| XECs235 | - | *-* |  | | - | - | | - | 219 | 3.29 | | - | - | - | - | pseudo |
| XECs236 | - | *-* |  | | - | - | | - | 211 | 2.72 | | - | - | - | - | pseudo |
| XECs237 | 9E-35 | *Shigella flexneri* |  | | - | - | | - | 168 | 2.72 | | 9 | -3.3 | - | - | pseudo |
| XECs238 | 3E-18 | *Escherichia coli* |  | | - | - | | - | 62 | 4.38 | | 0 | -5.3 | - | - | real |
| XECs239 | - | *-* |  | | - | - | | - | 214 | 2.72 | | 16 | -3 | - | - | pseudo |
| XECs240 | 3E-90 | *Escherichia coli* |  | | annotated in EDL933 as Z2994 | - | | - | 118 | 4.44 | | - | - | - | - | real |
| XECs241 | 7E-19 | *Escherichia coli* |  | | - | - | | - | 203 | 4.93 | | - | - | - | - | real |
| XECs242 | - | - |  | | - | - | | - | 146 | 5.59 | | - | - | 81 | -12.3 | pseudo |
| XECs243 | 3E-27 | *Escherichia coli* |  | | - | - | | - | 214 | 6.06 | | - | - | 6 | -13.2 | real |
| XECs244 | 2E-16 | *Escherichia coli* |  | | - | - | | - | 35 | 0.64 | | 11 | -4.8 | - | - | real |
| XECs245 | 5E-17 | *Escherichia coli* |  | | annotated in EDL933 as Z2105 | - | | - | 225 | 4.98 | | - | - | - | - | real |
| XECs246 | 1E-13 | *Escherichia coli* |  | | partial hit to annotated protein ECs2266 | - | | - | 233 | 4.98 | | - | - | - | - | real |
| XECs247 | 1E-09 | *Escherichia coli* |  | | partial hit to annotated protein ECs2266 | - | | - | 211 | 4.98 | | 8 | -3.3 | - | - | real |
| XECs248 | 3E-08 | *Escherichia coli* |  | | - | - | | - | 100 | 6.34 | | - | - | - | - | real |
| XECs249 | - | - |  | | - | - | | - | 209 | 4.02 | | - | - | - | - | pseudo |
| XECs250 | - | - |  | | - | - | | - | 39 | 5.31 | | - | - | - | - | pseudo |
| XECs251 | 7E-09 | *Escherichia coli* |  | | - | - | | - | 165 | 5.77 | | 20 | -6 | - | - | real |
| XECs252 | 5E-36 | *Escherichia coli* |  | | is X045 in EDL 933 (Neuhaus et al., 2016) | - | | - | 52 | 7.39 | | - | - | - | - | real |
| XECs253 | - | - |  | | - | - | | - | 152 | 6.2 | | - | - | - | - | pseudo |
| XECs254 | - | - |  | | - | - | | - | 40 | 4.17 | | 2 | -3.1 | - | - | pseudo |
| XECs255 | 5E-07 | *Escherichia coli* |  | | partial hit to annotated protein ECs0550 | - | | - | 191 | 1.16 | | 18 | -3.2 | 4 | -14.1 | real |
| XECs256 | - | - |  | | - | - | | - | 111 | 3.45 | | 20 | -7.4 | - | - | pseudo |
| XECs257 | 1E-23 | *Escherichia coli* |  | | - | - | | - | 206 | 8.39 | | - | - | - | - | pseudo |
| XECs258 | - | *-* |  | | - | - | | - | 124 | 6.26 | | - | - | 205 | -15.6 | pseudo |
| XECs259 | 8E-28 | *Escherichia coli* |  | | - | - | | - | 63 | 6.24 | | - | - | - | - | real |
| XECs260 | - | - |  | | - | - | | - | 92 | 6.24 | | - | - | - | - | pseudo |
| XECs261 | 4E-40 | *Escherichia coli* |  | | is X047 in EDL 933 (Neuhaus et al., 2016) | - | | - | 44 | 2.8 | | - | - | - | - | real |
| XECs262 | 3E-19 | *Escherichia coli* |  | | annotated as hypothetical protein in other EHEC strain | - | | - | 54 | 2.8 | | - | - | 22 | -15.2 | real |
| XECs263 | - | *-* |  | | - | - | | - | 177 | 5.07 | | 5 | -6.3 | 115 | -14.8 | pseudo |
| XECs264 | - | - |  | | - | - | | - | 187 | 3.21 | | - | - | - | - | pseudo |
| XECs265 | 2E-69 | *Escherichia coli* |  | | - | - | | - | 213 | 3.01 | | - | - | - | - | real |
| XECs266 | 4E-61 | *Escherichia coli* |  | | annotated as hypothetical protein in other EHEC strain | - | | - | 134 | 1.39 | | 15 | -5.8 | - | - | real |
| XECs267 | 4E-12 | *Escherichia coli* |  | | - | 1E-06 | | 1E-06 | 73 | 2.21 | | 1 | -9 | - | - | pseudo |
| XECs268 | 0.001 | *Salmonella enterica* |  | | - | - | | - | 85 | 3.93 | | 1 | -6.8 | - | - | real |
| XECs269 | 4E-24 | *Escherichia coli* |  | | annotated as hypothetical protein in other EHEC strain | - | | - | 193 | 3.38 | | - | - | - | - | real |
| XECs270 | - | - |  | | - | - | | - | 151 | 4.04 | | - | - | - | - | pseudo |
| XECs271 | - | *-* |  | | - | - | | - | 46 | 3.68 | | - | - | - | - | pseudo |
| XECs272 | - | - |  | | - | - | | - | 29 | 7 | | 17 | -4.2 | - | - | pseudo |
| XECs273 | 4E-05 | *Salmonella enterica* |  | | - | - | | - | 60 | 4.74 | | 17 | -4 | - | - | real |
| XECs274 | 2E-07 | *Escherichia coli* |  | | - | - | | - | 218 | 5.03 | | - | - | 75 | -17.8 | real |
| XECs275 | 1E-08 | *Escherichia coli* |  | | - | - | | - | 115 | 5.03 | | 12 | -3.8 | 166 | -17.8 | real |
| XECs276 | 7E-17 | *Escherichia coli* |  | | - | - | | - | 27 | 4.86 | | 10 | -5.1 | - | - | pseudo |
| XECs277 | - | - |  | | - | - | | - | 93 | 11.23 | | - | - | 16 | -22.8 | pseudo |
| XECs278 | 1E-18 | *Escherichia coli* |  | | annotated as hypothetical protein in other EHEC strain | - | | - | 50 | 3.24 | | - | - | 13 | -17.6 | real |
| XECs279 | 1E-19 | *Escherichia coli* |  | | - | - | | - | 202 | 1.16 | | - | - | - | - | real |
| XECs280 | 4E-19 | *Escherichia coli* |  | | - | - | | - | 154 | 3.61 | | 1 | -2.9 | - | - | real |
| XECs281 | 2E-14 | *Escherichia coli* |  | | - | - | | - | 85 | 5.88 | | - | - | - | - | real |
| XECs282 | - | - |  | | - | - | | - | 89 | 5.88 | | - | - | - | - | pseudo |
| XECs283 | - | - |  | | - | - | | - | 202 | 4.23 | | - | - | 203 | -17.5 | pseudo |
| XECs284 | - | - |  | | - | - | | - | 71 | 5.27 | | 13 | -6.1 | - | - | pseudo |
| XECs285 | - | - |  | | - | - | | - | 213 | 5.41 | | 6 | -5.3 | 104 | -14 | pseudo |
| XECs286 | - | - |  | | - | - | | - | 68 | 2.12 | | - | - | - | - | pseudo |
| XECs287 | 3E-10 | *Escherichia coli* |  | | - | - | | - | 210 | 1.06 | | 14 | -3.7 | 15 | -21 | real |
| XECs288 | - | - |  | | - | - | | - | 28 | 1.61 | | - | - | - | - | pseudo |
| XECs289 | 3E-24 | *Shigella sonnei* |  | | - | - | | - | 204 | 2.81 | | - | - | - | - | real |
| XECs290 | - | - |  | | - | - | | - | 174 | 4.02 | | - | - | - | - | pseudo |
| XECs291 | 9E-31 | *Escherichia coli* |  | | - | - | | - | 204 | 3.39 | | - | - | - | - | real |
| XECs292 | - | - |  | | - | - | | - | 212 | 3.04 | | - | - | - | - | pseudo |
| XECs293 | - | - |  | | - | - | | - | 55 | 2.6 | | - | - | - | - | pseudo |
| XECs294 | 0 | *Escherichia coli* |  | | - | - | | - | 87 | 1.08 | | - | - | - | - | real |
| XECs295 | - | - |  | | - | - | | - | 202 | 2.22 | | - | - | - | - | pseudo |
| XECs296 | - | *-* |  | | - | - | | - | 29 | 5.78 | | 7 | -5.1 | - | - | pseudo |
| XECs297 | 7E-05 | *Escherichia coli* |  | | - | - | | - | 164 | 1.77 | | - | - | 245 | -12.7 | real |
| XECs298 | - | - |  | | - | - | | - | 42 | 3.21 | | - | - | - | - | pseudo |
| XECs299 | 1E-20 | *Escherichia coli* |  | | annotated in EDL933 as Z3917 | - | | - | 213 | 1.77 | | - | - | - | - | real |
| XECs300 | 1E-08 | *Escherichia coli* |  | | annotated as hypothetical protein in other EHEC strain | - | | - | 194 | 2.98 | | - | - | 4 | -13.5 | real |
| XECs301 | 4E-19 | *Escherichia coli* |  | | annotated as ferredoxin in other EHEC strain | - | | - | 78 | 6.87 | | - | - | - | - | real |
| XECs302 | 1E-04 | *Escherichia coli* |  | | - | - | | - | 190 | 0.38 | | - | - | 35 | -13.1 | pseudo |
| XECs303 | 2E-13 | *Escherichia coli* |  | | - | - | | - | 216 | 3.51 | | 6 | -3.8 | - | - | real |
| XECs304 | 2E-06 | *Salmonella enterica* |  | | - | - | | - | 233 | 2.83 | | - | - | - | - | real |
| XECs305 | - | - |  | | - | - | | - | 76 | 6.15 | | - | - | - | - | pseudo |
| XECs306 | - | - |  | | - | - | | - | 236 | 5.22 | | - | - | - | - | pseudo |
| XECs307 | - | - |  | | - | - | | - | 94 | 4.32 | | - | - | - | - | pseudo |
| XECs308 | 3E-35 | *Shigella sonnei* |  | | - | - | | - | 196 | 4.32 | | 9 | -4.2 | - | - | real |
| XECs309 | - | - |  | | - | - | | - | 210 | 4.32 | | - | - | - | - | pseudo |
| XECs310 | 3E-17 | *Escherichia coli* |  | | - | - | | - | 125 | 0.45 | | - | - | - | - | real |
| XECs311 | 5E-39 | *Escherichia coli* |  | | annotated in EDL933 as Z4007 | - | | - | 55 | 0.9 | | 2 | -4.8 | 130 | -12.8 | real |
| XECs312 | - | - |  | | - | - | | - | 65 | 0.9 | | 12 | -4.8 | 180 | -12.8 | pseudo |
| XECs313 | 0 | *Escherichia coli* |  | | - | - | | - | 171 | 0.9 | | 2 | -3.1 | - | - | real |
| XECs314 | 2E-42 | *Shigella dysenteriae* |  | | - | - | | - | 68 | 2.08 | | 12 | -3.3 | - | - | real |
| XECs315 | - | *-* |  | | - | - | | - | 205 | 2.08 | | - | - | - | - | pseudo |
| XECs316 | - | - |  | | - | - | | - | 201 | 1.69 | | 2 | -3.8 | - | - | pseudo |
| XECs317 | 3E-11 | *Shigella flexneri* |  | | - | - | | - | 233 | 2.48 | | 19 | -6.1 | 20 | -22.8 | pseudo |
| XECs318 | 4E-31 | *Escherichia coli* |  | | - | - | | - | 62 | 5.44 | | - | - | 166 | -22.8 | real |
| XECs319 | - | - |  | | - | - | | - | 63 | 2.48 | | - | - | - | - | pseudo |
| XECs320 | - | - |  | | - | - | | - | 234 | 2.23 | | - | - | - | - | pseudo |
| XECs321 | - | - |  | | - | - | | . | 24 | 3.6 | | 6 | -5.5 | - | - | pseudo |
| XECs322 | 8E-37 | *Escherichia coli* |  | | - | - | | - | 191 | 0.63 | | - | - | - | - | real |
| XECs323 | 1E-20 | *Escherichia coli* |  | | annotated as hypothetical protein in other EHEC strain | - | | - | 87 | 3.96 | | - | - | - | - | real |
| XECs324 | - | - |  | | - | - | | - | 78 | 2.9 | | 10 | -4.6 | - | - | pseudo |
| XECs325 | 1E-20 | *Escherichia coli* |  | | - | - | | - | 194 | 2.2 | | 19 | -3.1 | - | - | real |
| XECs326 | 3E-04 | *Cronobacter sakazakii* |  | | is X054 in EDL 933 (Neuhaus et al., 2016) | - | | - | 191 | 6.12 | | - | - | 78 | -15.2 | real |
| XECs327 | - | - |  | | - | - | | - | 166 | 6.12 | | - | - | 155 | -15.2 | pseudo |
| XECs328 | - | - |  | | - | - | | - | 135 | 3.4 | | - | - | - | - | pseudo |
| XECs329 | 6E-15 | *Escherichia coli* |  | | partial hit to annotated protein ECs0550 | - | | - | 85 | 2.64 | | - | - | - | - | real |
| XECs330 | 4E-25 | *Escherichia coli* |  | | - | - | | - | 210 | 2.62 | | 6 | -4.2 | - | - | real |
| XECs331 | 1E-15 | *Escherichia coli* |  | | - | - | | - | - | - | | 17 | -4.2 | 219 | -37.7 | real |
| XECs332 | 1E-06 | *Escherichia coli* |  | | - | - | | - | 213 | 4.55 | | 5 | -4.8 | - | - | pseudo |
| XECs333 | 3E-42 | *Escherichia coli* |  | | annotated as hypothetical protein in other EHEC strain | - | | - | 215 | 0.23 | | - | - | - | - | real |
| XECs334 | 2E-16 | *Escherichia coli* |  | | annotated as hypothetical protein in other EHEC strain | - | | - | 170 | 1.28 | | 0 | -3.1 | 163 | -14.1 | real |
| XECs335 | - | - |  | | - | - | | - | 94 | 1.51 | | - | - | - | - | pseudo |
| XECs336 | - | - |  | | - | - | | - | 206 | 5.2 | | 15 | -6.4 | 74 | -15.3 | pseudo |
| XECs337 | 1E-12 | *Escherichia coli* |  | | - | - | | - | 163 | 6.7 | | 19 | -6.1 | 29 | -19.3 | real |
| XECs338 | 2E-09 | *Escherichia coli* |  | | - | - | | - | 73 | 1.55 | | 7 | -5.3 | - | - | real |
| XECs339 | 2E-14 | *Shigella dysenteriae* |  | | - | - | | - | 85 | 2.71 | | - | - | - | - | pseudo |
| XECs340 | 2E-20 | *Escherichia coli* |  | | - | - | | - | 229 | 1.5 | | 9 | -3.5 | - | - | real |
| XECs341 | 3E-13 | *Escherichia coli* |  | | - | - | | - | 195 | 2.91 | | 8 | -6 | - | - | real |
| XECs342 | 5E-13 | *Escherichia coli* |  | | annotated in EDL933 as Z4474 | - | | - | 199 | 2.91 | | 12 | -6 | - | - | real |
| XECs343 | 7E-25 | *Escherichia coli* |  | | - | - | | - | 124 | 6.17 | | - | - | - | - | pseudo |
| XECs344 | - | - |  | | - | - | | - | 99 | 5.6 | | - | - | 178 | -15 | real |
| XECs345 | - | - |  | | - | - | | - | 235 | 3.7 | | - | - | - | - | pseudo |
| XECs346 | - | - |  | | - | - | | - | 24 | 4.34 | | - | - | - | - | pseudo |
| XECs347 | 2E-11 | *Shigella flexneri* |  | | - | - | | - | 208 | 2.08 | | - | - | - | - | pseudo |
| XECs348 | - | - |  | | - | - | | - | 176 | 1.81 | | - | - | - | - | pseudo |
| XECs349 | 8E-19 | *Escherichia coli* |  | | - | - | | - | 97 | 2.67 | | - | - | - | - | real |
| XECs350 | 3E-13 | *Escherichia coli* |  | | - | - | | - | 190 | 2.82 | | - | - | - | - | real |
| XECs351 | 1E-37 | *Escherichia coli* |  | | is X054 in EDL 933 (Neuhaus et al., 2016) | - | | - | 69 | 2.97 | | - | - | - | - | real |
| XECs352 | 1E-22 | *Escherichia coli* |  | | - | - | | - | 100 | 4.55 | | 9 | -5.5 | - | - | real |
| XECs353 | - | *-* |  | | - | - | | - | 175 | 2.52 | | 8 | -6 | - | - | pseudo |
| XECs354 | 2E-14 | *Escherichia coli* |  | | - | - | | - | 166 | 3.22 | | - | - | - | - | real |
| XECs355 | - | - |  | | - | - | | - | 244 | 1.97 | | - | - | - | - | real |
| XECs356 | - | - |  | | - | - | | - | 183 | 5.35 | | - | - | - | - | pseudo |
| XECs357 | 1E-44 | *Escherichia coli* |  | | - | - | | - | 119 | 2.39 | | - | - | - | - | real |
| XECs358 | - | - |  | | - | - | | - | 94 | 7.22 | | 10 | -3.6 | - | - | pseudo |
| XECs359 | 3E-19 | *Escherichia coli* |  | | - | - | | - | 107 | 5.4 | | 19 | -5.7 | - | - | real |
| XECs360 | - | - |  | | - | - | | - | 158 | 3.69 | | - | - | - | - | pseudo |
| XECs361 | - | - |  | | - | - | | - | 162 | 3.69 | | - | - | - | - | pseudo |
| XECs362 | - | - |  | | - | - | | - | 44 | 3.09 | | - | - | - | - | pseudo |
| XECs363 | 3E-08 | *Escherichia coli* |  | | - | - | | - | 150 | 1.94 | | - | - | - | - | real |
| XECs364 | 2E-05 | *Escherichia coli* |  | | - | - | | - | 115 | 4.73 | | - | - | 50 | -17.2 | real |
| XECs365 | 2E-33 | *Escherichia coli* |  | | - | - | | - | 169 | 0.35 | | - | - | - | - | real |
| XECs366 | 2E-20 | *Escherichia coli* |  | | annotated as hypothetical protein in other EHEC strain | - | | - | 91 | 3.69 | | - | - | - | - | real |
| XECs367 | 1E-22 | *Escherichia coli* |  | | - | - | | - | 131 | 3.02 | | - | - | - | - | real |
| XECs368 | - | *-* |  | | - | - | | - | 37 | 2.14 | | - | - | 108 | -12.4 | pseudo |
| XECs369 | - | - |  | | - | - | | - | 201 | 1.94 | | 12 | -4.2 | - | - | pseudo |
| XECs370 | - | *-* |  | | - | - | | - | 27 | 5.56 | | 17 | -3.3 | 46 | -19.6 | pseudo |
| XECs371 | 1E-57 | *Staphylococcus epidermidis* |  | | - | - | | - | 251 | 2.7 | | - | - | - | - | real |
| XECs372 | - | - |  | | - | - | | - | 241 | 4.56 | | - | - | 150 | -20.4 | pseudo |
| XECs373 | - | *-* |  | | - | - | | - | 73 | 1.3 | | 10 | -3.3 | - | - | pseudo |
| XECs374 | 9E-38 | *Escherichia coli* |  | | - | - | | - | 215 | 2.3 | | 9 | -5.6 | - | - | real |
| XECs375 | 6E-11 | *Escherichia coli* |  | | - | - | | - | 187 | 2.3 | | - | - | - | - | real |
| XECs376 | - | - |  | | - | - | | - | 163 | 0.92 | | - | - | - | - | pseudo |
| XECs377 | - | - |  | | - | - | | - | 47 | 4.74 | | 2 | -3.4 | 164 | -17.6 | pseudo |
| XECs378 | 2E-08 | *Escherichia coli* |  | | - | - | | - | 77 | 5.11 | | - | - | 200 | -14.2 | real |
| XECs379 | 2E-40 | *Escherichia coli* |  | | is X061 in EDL 933 (Neuhaus et al., 2016) | - | | - | 172 | 3.94 | | 0 | -4 | - | - | real |
| XECs380 | 3E-21 | *Escherichia coli* |  | | - | - | | - | 124 | 0.75 | | - | - | - | - | real |
| XECs381 | 2E-06 | *Escherichia coli* |  | | - | - | | - | 28 | 1.26 | | - | - | - | - | real |
| XECs382 | - | *-* |  | | - | - | | - | 162 | 2.2 | | - | - | - | - | pseudo |
| XECs383 | - | - |  | | - | - | | - | 52 | 5.52 | | 13 | -4.6 | - | - | pseudo |
| XECs384 | - | - |  | | - | - | | - | 54 | 5.52 | | 15 | -4.6 | - | - | pseudo |
| XECs385 | - | - |  | | - | - | | - | 126 | 2.16 | | - | - | - | - | pseudo |
| XECs386 | - | - |  | | - | - | | - | 219 | 8.23 | | - | - | - | - | pseudo |
| XECs387 | 8E-10 | *Enterobacter aerogenes* |  | | - | - | | - | 57 | 4.05 | | - | - | 131 | -17.4 | real |
| XECs388 | 2E-16 | *Escherichia coli* |  | | - | - | | - | 113 | 4.05 | | - | - | 111 | -17.4 | real |
| XECs389 | - | - |  | | - | - | | - | 212 | 1.29 | | - | - | - | - | pseudo |
| XECs390 | - | - |  | | - | - | | - | 137 | 3.22 | | 14 | -3.4 | - | - | pseudo |
| XECs391 | - | - |  | | - | - | | - | 105 | 3.22 | | - | - | - | - | pseudo |
| XECs392 | - | *-* |  | | - | - | | - | 142 | 0.89 | | - | - | - | - | pseudo |
| XECs393 | 9E-08 | *Escherichia coli* |  | | - | - | | - | 33 | 0.91 | | 2 | -2.9 | - | - | pseudo |
| XECs394 | 2E-15 | *Escherichia coli* |  | | - | - | | - | 97 | 3.75 | | 3 | -4.8 | - | - | real |
| XECs395 | 2E-53 | *Escherichia coli* |  | | annotated as membrane protein insertion efficiency factor in other EHEC strain | - | | - | 169 | 0.43 | | - | - | - | - | real |
| XECs396 | - | - |  | | - | - | | - | 105 | 4.83 | | 11 | -3.8 | - | - | pseudo |
| XECs397 | 3E-07 | *Klebsiella oxytoca* |  | | - | 0.092 | | 2E-04 | 107 | 5.84 | | - | - | - | - | real |
| XECs398 | 6E-18 | *Escherichia coli* |  | | - | - | | - | 135 | 5.27 | | - | - | - | - | real |
| XECs399 | 1E-18 | *Escherichia coli* |  | | - | - | | - | 29 | 3.21 | | - | - | - | - | real |
| XECs400 | - | - |  | | - | - | | - | 30 | 3.67 | | - | - | 141 | -15.8 | pseudo |
| XECs401 | 5E-20 | *Escherichia coli* |  | | - | - | | - | 149 | 2.27 | | 7 | -3.4 | - | - | real |
| XECs402 | 2E-21 | *Escherichia coli* |  | | - | - | | - | 163 | 0.62 | | 4 | -5,5 | - | - | real |
| XECs403 | - | - |  | | - | - | | - | 176 | 5.38 | | - | - | - | - | pseudo |
| XECs404 | 9E-23 | *Shigella flexneri* |  | | - | - | | - | 204 | 3.29 | | 11 | -6.7 | - | - | real |
| XECs405 | 5E-28 | *Escherichia coli* |  | | is X065 in EDL 933 (Neuhaus et al., 2016) | - | | - | 226 | 3.29 | | - | - | - | - | real |
| XECs406 | 3E-15 | *Escherichia coli* |  | | - | 1E-06 | | 1E-06 | 57 | 3.81 | | 5 | -3.9 | - | - | real |
| XECs407 | 1E-15 | *Escherichia coli* |  | | - | - | | - | 131 | 0.9 | | 10 | -3.8 | 25 | -11.8 | real |
| XECs408 | 1E-10 | *Escherichia coli* |  | | - | - | | - | 34 | 2.05 | | 5 | -4.2 | 2 | -11.8 | real |
| XECs409 | 3E-13 | *Escherichia coli* |  | | - | - | | - | 58 | 4.67 | | - | - | - | - | real |
| XECs410 | - | - |  | | - | - | | - | 81 | 2.17 | | - | - | - | - | pseudo |
| XECs411 | 3E-27 | *Escherichia coli* |  | | - | - | | - | 88 | 3.52 | | - | - | - | - | real |
| XECs412 | 9E-43 | *Shigella dysenteriae* |  | | - | - | | - | 66 | 3.52 | | - | - | - | - | real |
| XECs413 | 6E-60 | *Escherichia coli* |  | | - | - | | - | 137 | 2.49 | | 3 | -4.8 | - | - | real |
| XECs414 | 6E-33 | *Escherichia coli* |  | | - | - | | - | 119 | 1.79 | | 5 | -3.6 | - | - | real |
| XECs415 | - | - |  | | - | - | | - | 44 | 3.36 | | 11 | -5.1 | - | - | pseudo |
| XECs416 | - | - |  | | - | - | | - | 138 | 3.36 | | - | - | - | - | pseudo |
| XECs417 | - | - |  | | - | - | | - | 180 | 1.2 | | - | - | - | - | pseudo |
| XECs418 | - | - |  | | - | - | | - | 44 | 3.88 | | - | - | - | - | pseudo |
| XECs419 | - | - |  | | - | - | | - | 48 | 2.6 | | - | - | - | - | pseudo |
| XECs420 | - | - |  | | - | - | | - | 37 | 3.68 | | - | - | - | - | pseudo |
| XECs421 | - | - |  | | is X068 in EDL 933 (Neuhaus et al., 2016) | - | | - | 42 | 3.68 | | - | - | - | - | pseudo |
| XECs422 | - | - |  | | - | - | | - | 100 | 0.39 | | - | - | - | - | pseudo |
| XECs423 | 1E-67 | *Escherichia coli* |  | | - | - | | - | 235 | 3.75 | | 0 | -3.8 | - | - | real |
| XECs424 | 1E-13 | *Escherichia coli* |  | | - | - | | - | 246 | 3.75 | | 11 | -3.8 | - | - | real |
| XECs425 | - | - |  | | - | - | | - | 149 | 4.83 | | - | - | - | - | pseudo |
| XECs426 | - | - |  | | - | - | | - | 138 | 4.95 | | - | - | - | - | pseudo |
| XECs427 | - | - |  | | - | - | | - | 144 | 1.95 | | - | - | - | - | pseudo |
| XECs428 | - | - |  | | - | - | | - | 216 | 2.61 | | 15 | -9.6 | - | - | pseudo |
| XECs429 | 9E-12 | *Escherichia coli* |  | | - | - | | - | - | - | | - | - | - | - | real |
| XECs430 | - | - |  | | - | - | | - | 26 | 3.83 | | 12 | -8.2 | - | - | pseudo |
| XECs431 | - | - |  | | - | - | | - | 230 | 1.46 | | 18 | -3.7 | 0 | -11.8 | pseudo |
| XECs432 | - | - |  | | - | - | | - | 38 | 9.72 | | 9 | -4.9 | - | - | pseudo |
| XECs433 | 1E-19 | *Escherichia coli* |  | | - | - | | - | 165 | 3.32 | | - | - | - | - | real |
| XECs434 | 1E-17 | *Escherichia coli* |  | | - | - | | - | 76 | 2.9 | | - | - | 201 | -12.4 | real |
| XECs435 | - | - |  | | - | - | | - | 93 | 2.4 | | 1 | -4.8 | - | - | pseudo |
| XECs436 | 2E-09 | *Escherichia coli* |  | | - | - | | - | 164 | 2.03 | | 8 | -4.8 | - | - | pseudo |
| XECs437 | - | - |  | | - | - | | - | 147 | 3.33 | | 10 | -3.4 | - | - | pseudo |
| XECs438 | - | *-* |  | | - | - | | - | 152 | 3.33 | | 15 | -3.4 | - | - | pseudo |
| XECs439 | 2E-23 | *Shigella sonnei* |  | | - | - | | - | 88 | 3.33 | | 3 | -2.9 | - | - | pseudo |
| XECs440 | 3E-16 | *Shigella dysenteriae* |  | | - | - | | - | 55 | 1.17 | | - | - | - | - | pseudo |
| XECs441 | 7E-26 | uncultured bacterium |  | | - | - | | - | 101 | 1.17 | | 4 | -4.1 | 266 | -17.8 | real |
| XECs442 | 3E-23 | *Escherichia coli* |  | | - | - | | - | 187 | 2.19 | | 1 | -5.9 | 175 | -15.7 | real |
| XECs443 | 1E-07 | *Shigella dysenteriae* |  | | - | - | | - | 178 | 1.58 | | - | - | - | - | real |
| XECs444 | 5E-23 | *Shigella dysenteriae* |  | | - | - | | - | 119 | 3.13 | | - | - | - | - | real |
| XECs445 | - | - |  | | - | - | | - | 114 | 2.87 | | - | - | - | - | pseudo |
| XECs446 | 1E-35 | *Escherichia coli* |  | | - | - | | - | 168 | 1.74 | | 1 | -4.9 | - | - | real |
| XECs447 | 1E-16 | *Escherichia coli* |  | | - | - | | - | 199 | 0.66 | | 12 | -4.2 | - | - | real |
| XECs448 | 3E-26 | *Escherichia coli* |  | | annotated as hypothetical protein in other EHEC strain | - | | - | 32 | 0.3 | | - | - | - | - | real |
| XECs449 | 1E-05 | *Escherichia coli* |  | | - | - | | - | 48 | 4.86 | | 15 | -4.8 | 115 | -17.2 | real |
| XECs450 | 4E-17 | *Escherichia coli* |  | | - | - | | - | 166 | 1.22 | | - | - | - | - | real |
| XECs451 | - | - |  | | - | - | | - | 56 | 5.33 | | - | - | - | - | pseudo |
| XECs452 | - | - |  | | - | - | | - | 64 | 2.25 | | 6 | -3.1 | - | - | pseudo |
| XECs453 | - | - |  | | - | - | | - | 29 | 2.25 | | - | - | - | - | pseudo |
| XECs454 | - | - |  | | - | - | | - | 94 | 6.14 | | - | - | - | - | pseudo |
| XECs455 | - | - |  | | - | - | | - | 236 | 7.45 | | - | - | - | - | pseudo |
| XECs456 | 7E-18 | *Escherichia coli* |  | | - | - | | - | 198 | 1.58 | | 19 | -3.7 | - | - | real |
| XECs457 | 3E-25 | *Escherichia coli* |  | | annotated as hypothetical protein in other EHEC strain | - | | - | 74 | 6.38 | | - | - | - | - | real |
| XECs458 | - | - |  | | - | - | | - | 163 | 1.2 | | - | - | 92 | -16.9 | pseudo |
| XECs459 | 4E-32 | *Shigella flexneri* |  | | - | - | | - | 188 | 1.2 | | - | - | 28 | -16.9 | real |
| XECs460 | - | - |  | | - | - | | - | 115 | 1.57 | | 21 | -3.1 | - | - | pseudo |
| XECs461 | - | - |  | | - | - | | - | 53 | 0.68 | | 7 | -7.6 | - | - | pseudo |
| XECs462 | - | *-* |  | | - | - | | - | 180 | 6.86 | | - | - | - | - | pseudo |
| XECs463 | - | - |  | | - | - | | - | 209 | 2.1 | | 7 | -4.9 | - | - | pseudo |
| XECs464 | 5E-30 | *Escherichia coli* |  | | is X072 in EDL 933 (Neuhaus et al., 2016) | - | | - | 236 | 0.35 | | 2 | -4.3 | 68 | -21.3 | real |
| XECs465 | 5E-04 | *Yersinia pestis* |  | | - | - | | - | 60 | 6.95 | | - | - | - | - | pseudo |
