# Supplementary material for: Discovery of numerous novel small genes in the intergenic regions of the Escherichia coli O157:H7 Sakai genome
Source: PLoS One. 2017 Sep 13;12(9):e0184119. doi: 10.1371/journal.pone.0184119 (PMC5597208; doi:10.1371/journal.pone.0184119)
Supplement: S4 Table — With bioinformatics methods the presence of a σ70 promoter, a ρ-independent terminator, a Shine-Dalgarno sequence and selection pressure (kA/kS) were predicted or estimated. The last column gives the classification of the short genes by the machine-learning algorithm. (DOCX) [file pone.0184119.s006.docx]

|  | K_A_/K_s_ | | promoter | | Shine Dalgarno Sequence | | terminator | |  |
| --- | --- | --- | --- | --- | --- | --- | --- | --- | --- |
| gene name | value | p-value | bps upstream start codon | LDF score | bps upstream start codon | ∆G° | bps downstream stop codon | score | machine learning algorithm |
| ECs0001 | - | - | 224 | 3.54 | - | - | 5 | -25.3 | pseudo |
| ECs0079 | - | - | 110 | 3.70 | 6 | -3.3 | 13 | -19.1 | real |
| ECs0105 | - | - | 247 | 2.55 | 3 | -7.4 | - | - | real |
| ECs0232 | - | - | 270 | 1.14 | 7 | -5.1 | - | - | real |
| ECs0239 | - | - | 167 | 4.75 | 9 | -4.8 | 82 | -11.9 | real |
| ECs0243 | - | - | 152 | 2.06 | 4 | -6 | - | - | real |
| ECs0275 | - | - | 173 | 3.94 | 3 | -9.6 | - | - | real |
| ECs0301 | 1.812 | 8E-06 | 127 | 2.42 | 6 | -6 | - | - | real |
| ECs0326 | - | - | 185 | 1.15 | - | - | - | - | real |
| ECs0439 | - | - | 277 | 5.95 | 3 | -4.8 | - | - | real |
| ECs0513 | - | - | 108 | 3.51 | 4 | -3.1 | - | - | real |
| ECs0519 | - | - | 111 | 0.71 | 3 | -2.9 | 9 | -15.2 | real |
| ECs0590 | - | - | 150 | 1.89 | 5 | -4.4 | 9 | -15.2 | real |
| ECs0662 | - | - | 165 | 5.16 | 4 | -3.7 | 20 | -16.3 | real |
| ECs0665 | - | - | 180 | 4.01 | 4 | -5.1 | 35 | -15.7 | real |
| ECs0728 | - | - | 130 | 9.78 | 7 | -8.7 | 24 | -22 | real |
| ECs0790 | - | - | 279 | 6.11 | 4 | -3.7 | 8 | -14.2 | real |
| ECs0805 | - | - | 257 | 4.82 | 8 | -6.2 | - | - | real |
| ECs0808 | - | - | 259 | 1.50 | 8 | -4.2 | - | - | real |
| ECs0818 | - | - | 227 | 6.68 | 6 | -3.8 | - | - | real |
| ECs0822 | - | - | 116 | 1.65 | 12 | -4.1 | 34 | -15.6 | real |
| ECs0826 | - | - | 244 | 0.31 | 5 | -6.1 | - | - | real |
| ECs0849 | - | - | 180 | 6.22 | 19 | -3.4 | - | - | real |
| ECs0966 | - | - | 213 | 4.64 | 6 | -3.7 | 35 | -17.7 | real |
| ECs0969 | - | - | 61 | 4.30 | 1 | -4.3 | 40 | -15.6 | real |
| ECs1000 | - | - | 267 | 2.75 | 3 | -6.1 | - | - | real |
| ECs1037 | - | - | 90 | 5.63 | 6 | -6.9 | 9 | -21.5 | real |
| ECs1058 | - | - | 138 | 4.70 | 8 | -3.7 | - | - | real |
| ECs1059 | - | - | 102 | 1.44 | 4 | -4.6 | - | - | real |
| ECs1061 | - | - | 122 | 4.52 | 4 | -6.3 | - | - | real |
| ECs1065 | - | - | 126 | 4.75 | 11 | -6.4 | - | - | real |
| ECs1068 | - | - | 106 | 2.92 | 5 | -6 | - | - | real |
| ECs1098 | - | - | 218 | 5.11 | 4 | -6.4 | - | - | real |
| ECs1100 | - | - | 100 | 3.89 | 18 | -4.2 | - | - | real |
| ECs1119 | - | - | 101 | 0.85 | - | - | - | - | real |
| ECs1125 | - | - | - | - | 19 | -4.2 | - | - | real |
| ECs1127 | - | - | 280 | 3.84 | - | - | - | - | real |
| ECs1144 | - | - | 112 | 6.03 | 3 | -5.3 | - | - | real |
| ECs1145 | - | - | 244 | 6.71 | 18 | -3.8 | 204 | -14.2 | real |
| ECs1159 | - | - | 278 | 2.33 | 5 | -7.6 | 2 | -22.1 | real |
| ECs1170 | - | - | 257 | 4.82 | 8 | -6.2 | - | - | real |
| ECs1172 | - | - | 175 | 2.13 | 21 | -6 | - | - | real |
| ECs1173 | 0.672 | 8E-05 | 259 | 1.50 | 6 | -4.2 | - | - | real |
| ECs1178 | - | - | 201 | 2.33 | 4 | -6.8 | - | - | real |
| ECs1186 | - | - | 281 | 8.10 | 3 | -7.4 | - | - | real |
| ECs1188 | - | - | 232 | 2.38 | 3 | -5.4 | - | - | real |
| ECs1193 | - | - | 237 | 2.27 | 8 | -9.4 | 205 | -13.6 | real |
| ECs1210 | - | - | 174 | 1.55 | - | - | - | - | real |
| ECs1212 | - | - | 96 | 1.03 | 6 | -4 | - | - | real |
| ECs1248 | - | - | 130 | 2.19 | 9 | -4.9 | - | - | real |
| ECs1357 | - | - | 127 | 0.33 | - | - | - | - | real |
| ECs1359 | - | - | 106 | 3.06 | 20 | -3.5 | - | - | real |
| ECs1367 | - | - | 88 | 5.65 | 17 | -3.4 | - | - | real |
| ECs1379 | - | - | 89 | 2.44 | 18 | -5.3 | - | - | real |
| ECs1392 | - | - | 279 | 2.51 | 2 | -5.5 | - | - | real |
| ECs1436 | - | - | 101 | 3.75 | 4 | -3.3 | - | - | real |
| ECs1467 | - | - | 148 | 1.19 | 6 | -7.2 | - | - | real |
| ECs1505 | - | - | 207 | 4.61 | 5 | -4.9 | - | - | real |
| ECs1517 | - | - | 221 | 3.00 | 4 | -5.1 | - | - | real |
| ECs1530 | - | - | 100 | 0.26 | 6 | -4 | - | - | real |
| ECs1536 | - | - | 280 | 2.03 | - | - | - | - | real |
| ECs1537 | - | - | 99 | 3.33 | - | - | - | - | real |
| ECs1538 | - | - | 248 | 8.00 | 4 | -3.6 | - | - | real |
| ECs1539 | - | - | 273 | 3.84 | 3 | -3.6 | - | - | real |
| ECs1565 | - | - | 180 | 2.08 | - | - | - | - | real |
| ECs1569 | - | - | 247 | 1.86 | 8 | -3.7 | - | - | real |
| ECs1577 | - | - | 206 | 3.21 | 3 | -4.8 | - | - | real |
| ECs1579 | - | - | 252 | 1.87 | 3 | -4.2 | - | - | real |
| ECs1596 | - | - | 96 | 0.74 | - | - | - | - | real |
| ECs1624 | - | - | 207 | 0.89 | 5 | -6.1 | - | - | real |
| ECs1627 | - | - | 267 | 3.40 | 5 | -3.5 | - | - | real |
| ECs1631 | - | - | 279 | 0.97 | 0 | -3.6 | - | - | real |
| ECs1655 | - | - | 122 | 5.35 | 5 | -5 | - | - | real |
| ECs1673 | - | - | 134 | 1.65 | - | - | - | - | real |
| ECs1722 | - | - | 177 | 5.26 | 4 | -6.2 | - | - | real |
| ECs1764 | - | - | 228 | 1.29 | 3 | -5.7 | 26 | -14.1 | real |
| ECs1766 | - | - | 184 | 4.64 | 4 | -6.9 | - | - | real |
| ECs1782 | - | - | 153 | 4.16 | 6 | -3.8 | - | - | real |
| ECs1788 | - | - | 273 | 3.84 | 3 | -3.6 | 132 | -15.6 | real |
| ECs1856 | - | - | 153 | 5.83 | 6 | -4.8 | 80 | -20 | real |
| ECs1882 | - | - | 222 | 2.19 | 3 | -7.4 | - | - | real |
| ECs1884 | - | - | 100 | 1.81 | 2 | -4.6 | - | - | real |
| ECs1939 | - | - | 164 | 1.10 | - | - | - | - | real |
| ECs1940 | - | - | 141 | 9.66 | 14 | -5 | - | - | real |
| ECs1949 | - | - | 281 | 5.14 | 6 | -5.5 | - | - | real |
| ECs1960 | - | - | 130 | 3.03 | 18 | -2.9 | 168 | -15.6 | real |
| ECs1962 | - | - | 153 | 4.16 | 6 | -3.8 | - | - | real |
| ECs1967 | - | - | 273 | 3.84 | 3 | -3.6 | 132 | -15.6 | real |
| ECs1988 | - | - | 78 | 0.76 | - | - | - | - | real |
| ECs2004 | - | - | 97 | 2.56 | 3 | -4.6 | - | - | real |
| ECs2024 | - | - | 177 | 6.01 | 3 | -6.3 | - | - | real |
| ECs2025 | - | - | 126 | 2.63 | 3 | -3.7 | - | - | real |
| ECs2031 | - | - | 216 | 3.19 | 3 | -5.8 | 9 | -14.8 | real |
| ECs2040 | - | - | 161 | 4.13 | 3 | -7.1 | - | - | real |
| ECs2049 | - | - | 197 | 3.14 | - | - | - | - | real |
| ECs2059 | - | - | 264 | 4.81 | - | - | - | - | real |
| ECs2084 | - | - | 217 | 2.71 | 3 | -7 | 24 | -20.3 | real |
| ECs2085 | - | - | 184 | 6.02 | 5 | -2.9 | 271 | -16 | real |
| ECs2105 | - | - | 176 | 5.26 | - | - | - | - | real |
| ECs2139 | - | - | 80 | 1.92 | 1 | -4.7 | - | - | real |
| ECs2178 | - | - | 279 | 0.97 | 0 | -3.6 | - | - | real |
| ECs2181 | - | - | 145 | 3.12 | 5 | -4.8 | 14 | -11.8 | real |
| ECs2188 | - | - | 132 | 3.48 | 18 | -4.2 | - | - | real |
| ECs2192 | - | - | 186 | 4.08 | 5 | -6 | 32 | -13.7 | real |
| ECs2202 | - | - | 174 | 1.40 | 7 | -7.6 | - | - | real |
| ECs2212 | - | - | 213 | 3.09 | 4 | -6.3 | - | - | real |
| ECs2214 | - | - | 79 | 2.52 | 4 | -4.6 | 163 | -13 | real |
| ECs2253 | - | - | 152 | 0.99 | - | - | - | - | real |
| ECs2255 | - | - | 273 | 3.84 | 3 | -3.6 | 132 | -15.6 | real |
| ECs2261 | - | - | 99 | 3.89 | 18 | -4.2 | - | - | real |
| ECs2271 | - | - | 249 | 3.01 | 3 | -8.5 | - | - | real |
| ECs2280 | - | - | 241 | 1.99 | 10 | -3.8 | - | - | real |
| ECs2282 | - | - | 215 | 5.21 | 20 | -6 | - | - | real |
| ECs2284 | - | - | 143 | 3.15 | 4 | -4.6 | - | - | real |
| ECs2333 | 1.745 | 4E-05 | 261 | 3.72 | - | - | - | - | real |
| ECs2334 | - | - | 165 | 4.52 | - | - | - | - | real |
| ECs2382 | - | - | 257 | 6.64 | 7 | -4.8 | 3 | -14.8 | real |
| ECs2424 | - | - | 276 | 1.28 | - | - | - | - | real |
| ECs2497 | - | - | 255 | 4.36 | 20 | -3.5 | - | - | real |
| ECs2505 | - | - | 98 | 1.81 | 6 | -3.8 | - | - | real |
| ECs2520 | 1.602 | 8E-07 | 278 | 4.63 | 7 | -5.3 | 50 | -16.7 | real |
| ECs2526 | - | - | 189 | 4.31 | 6 | -4.8 | - | - | real |
| ECs2533 | 0.653 | 1E-06 | 229 | 2.64 | 6 | -5.3 | 47 | -14.8 | real |
| ECs2534 | - | - | 274 | 2.36 | 12 | -3.7 | 269 | -14.8 | real |
| ECs2536 | - | - | 252 | 3.89 | 16 | -4.8 | - | - | real |
| ECs2622 | - | - | 65 | 1.96 | 7 | -8.2 | - | - | real |
| ECs2627 | - | - | 113 | 1.66 | 3 | -8.7 | - | - | real |
| ECs2653 | - | - | 110 | 5.43 | 6 | -4.6 | - | - | real |
| ECs2691 | 0.67 | 1E-05 | 170 | 2.58 | 5 | -7.6 | - | - | real |
| ECs2695 | - | - | 168 | 2.38 | 4 | -8.5 | - | - | real |
| ECs2734 | - | - | 241 | 0.97 | 1 | -7.8 | - | - | real |
| ECs2743 | - | - | 147 | 3.83 | 18 | -4.2 | - | - | real |
| ECs2748 | - | - | - | - | 18 | -2.9 | 202 | -17.4 | real |
| ECs2755 | - | - | 249 | 3.22 | 3 | -6.4 | - | - | real |
| ECs2758 | - | - | 149 | 1.32 | 5 | -8.5 | - | - | real |
| ECs2760 | - | - | 141 | 1.98 | 7 | -4.8 | - | - | real |
| ECs2765 | - | - | 281 | 5.09 | 3 | -6 | - | - | real |
| ECs2769 | - | - | 230 | 2.84 | - | - | - | - | real |
| ECs2772 | - | - | 199 | 1.58 | 14 | -4.2 | - | - | real |
| ECs2804 | - | - | 241 | 0.92 | 5 | -4.2 | - | - | real |
| ECs2814 | - | - | 212 | 1.29 | 3 | -6 | - | - | real |
| ECs2833 | - | - | 79 | 4.45 | 12 | -7.6 | - | - | real |
| ECs2890 | - | - | 127 | 4.61 | - | - | - | - | real |
| ECs2969 | - | - | 277 | 1.32 | 6 | -4 | - | - | real |
| ECs2971 | - | - | 171 | 1.54 | - | - | - | - | real |
| ECs2978 | 1.584 | 1E-05 | 70 | 2.41 | - | - | - | - | real |
| ECs2980 | - | - | 137 | 2.16 | 4 | -8.2 | - | - | real |
| ECs2989 | - | - | 277 | 5.93 | 5 | -5.1 | - | - | real |
| ECs2997 | - | - | 201 | 2.33 | 4 | -6.8 | - | - | real |
| ECs3003 | - | - | 265 | 1.50 | 2 | -4.2 | - | - | real |
| ECs3004 | - | - | 175 | 2.01 | 2 | -5.8 | - | - | real |
| ECs3006 | - | - | 257 | 3.29 | 8 | -6.2 | - | - | real |
| ECs3079 | - | - | 229 | 4.66 | - | - | - | - | real |
| ECs3087 | - | - | 244 | 0.45 | 5 | -3.7 | - | - | real |
| ECs3239 | - | - | 73 | 3.25 | 5 | -7.9 | - | - | real |
| ECs3366 | - | - | 199 | 7.65 | 4 | -3.7 | - | - | real |
| ECs3372 | - | - | 240 | 5.87 | 18 | -8 | - | - | real |
| ECs3390 | - | - | - | - | 1 | -3.6 | 135 | -18 | real |
| ECs3479 | - | - | 66 | 2.86 | - | - | 20 | -23.3 | real |
| ECs3497 | - | - | 153 | 4.16 | 6 | -3.8 | - | - | real |
| ECs3513 | - | - | 123 | 2.91 | 4 | -3.6 | - | - | real |
| ECs3527 | - | - | 266 | 5.92 | 3 | -5.3 | - | - | real |
| ECs3553 | - | - | 173 | 4.09 | 4 | -5.7 | - | - | real |
| ECs3690 | 1.585 | 1E-04 | 268 | 3.58 | 18 | -6.2 | 2 | -13.9 | real |
| ECs3710 | - | - | 92 | 4.81 | 3 | -6 | 25 | -14.7 | real |
| ECs3815 | - | - | 223 | 2.32 | - | - | - | - | real |
| ECs3852 | - | - | 157 | 1.51 | 5 | -4.1 | - | - | real |
| ECs3854 | - | - | 114 | 4.15 | - | - | - | - | real |
| ECs3864 | - | - | 197 | 1.41 | 7 | -3.3 | - | - | real |
| ECs3891 | - | - | 243 | 2.89 | - | - | 47 | -11.9 | real |
| ECs3931 | - | - | 207 | 6.90 | 21 | -6 | 5 | -15.5 | real |
| ECs3948 | - | - | 165 | 6.31 | 6 | -3.7 | 46 | -19.5 | real |
| ECs3989 | - | - | 280 | 2.28 | - | - | - | - | real |
| ECs4115 | - | - | 194 | 5.57 | 1 | -3.7 | - | - | real |
| ECs4135 | - | - | 269 | 4.29 | 7 | -3.1 | 5 | -16.6 | real |
| ECs4140 | - | - | 124 | 5.22 | 3 | -4.6 | - | - | real |
| ECs4164 | - | - | 87 | 2.13 | 5 | -4 | 29 | -17.1 | real |
| ECs4167 | - | - | 117 | 1.51 | 6 | -3 | - | - | real |
| ECs4177 | - | - | 200 | 2.35 | 6 | -4.1 | 274 | -13.1 | real |
| ECs4199 | - | - | 193 | 3.23 | 5 | -3.7 | 4 | -21.5 | real |
| ECs4205 | - | - | 238 | 0.51 | 6 | -6.1 | - | - | real |
| ECs4220 | - | - | 165 | 5.76 | 4 | -5.1 | 41 | -24.2 | real |
| ECs4250 | - | - | 87 | 4.80 | 4 | -5.6 | - | - | real |
| ECs4381 | - | - | 151 | 3.68 | 0 | -4 | - | - | real |
| ECs4415 | - | - | 121 | 4.26 | 7 | -5.7 | - | - | real |
| ECs4441 | - | - | 140 | 5.09 | 5 | -3.7 | 19 | -21.6 | real |
| ECs4511 | - | - | 196 | 1.04 | 5 | -3.6 | 22 | -13.7 | real |
| ECs4536 | 1.452 | 1E-04 | 237 | 0.26 | - | - | - | - | real |
| ECs4587 | - | - | 71 | 3.39 | - | - | - | - | real |
| ECs4613 | - | - | 97 | 4.96 | 5 | -3.7 | 25 | -18 | pseudo |
| ECs4638 | - | - | 206 | 6.30 | 7 | -3.3 | - | - | real |
| ECs4644 | - | - | 175 | 7.76 | 7 | -3.6 | - | - | real |
| ECs4659 | - | - | 249 | 2.21 | 1 | -6.3 | - | - | real |
| ECs4701 | - | - | 202 | 6.17 | - | - | 21 | -19.5 | real |
| ECs4715 | - | - | 268 | 3.28 | - | - | - | - | real |
| ECs4863 | - | - | 240 | 5.28 | 3 | -4.1 | 16 | -14.1 | real |
| ECs4947 | - | - | 278 | 1.50 | 3 | -5.6 | 134 | -14.3 | real |
| ECs4958 | - | - | 119 | 3.34 | 8 | -6.3 | - | - | real |
| ECs4965 | - |  | - | - | 3 | -4.8 | - | - | real |
| ECs4991 | - | - | 281 | 2.91 | 9 | -5.9 | - | - | real |
| ECs4994 | 0.658 | 3E-06 | 91 | 2.74 | 0 | -4.8 | - | - | real |
| ECs4997 | - | - | 121 | 6.11 | 9 | -7.6 | - | - | real |
| ECs5028 | - | - | 206 | 3.32 | 4 | -5.1 | - | - | real |
| ECs5152 | - | - | 229 | 2.33 | 3 | -4.8 | - | - | real |
| ECs5178 | 0.716 | 1E-05 | 257 | 1.27 | - | - | - | - | real |
| ECs5224 | - | - | 212 | 9.17 | 3 | -5.5 | - | - | real |
| ECs5292 | - | - | 138 | 11.97 | 1 | -3.7 | - | - | real |
| ECs5303 | - | - | 226 | 1.99 | 5 | -9.4 | - | - | real |
| ECs5312 | - | - | 182 | 0.74 | 21 | -2.9 | - | - | real |
| ECs5360 | - | - | 228 | 2.70 | - | - | - | - | real |
| ECs5363 | - | - | 223 | 5.21 | - | - | - | - | real |
| ECs5364 | - | - | 206 | 0.28 | - | - | - | - | real |
| ECs5365 | - | - | 247 | 3.39 | - | - | - | - | real |
| ECs5374 | - | - | 203 | 10.30 | 15 | -3.4 | - | - | real |
| ECs5375 | - | - | 118 | 2.45 | 3 | -8.7 | 52 | -17.9 | real |
| ECs5380 | - | - | 279 | 3.17 | - | - | - | - | real |
| ECs5382 | - | - | 238 | 2.25 | 4 | -5.5 | - | - | real |
| ECs5391 | - | - | 238 | 0.83 | 4 | -6.8 | - | - | real |
| ECs5406 | - | - | 241 | 0.97 | 1 | -7.8 | - | - | real |
| ECs5411 | - | - | 145 | 2.33 | - | - | - | - | real |
| ECs5412 | - | - | 206 | 3.26 | - | - | 146 | -21.9 | real |
| ECs5414 | - | - | 185 | 3.23 | - | - | - | - | real |
| ECs5418 | - | - | - | - | 18 | -6.5 | - | - | real |
| ECs5420 | - | - | 196 | 2.77 | 21 | -5.3 | - | - | real |
| ECs5422 | - | - | 132 | 4.94 | 8 | -5.4 | 206 | -16.9 | real |
| ECs5432 | - | - | 191 | 8.33 | 5 | -4.8 | - | - | real |
| ECs5433 | - | - | 97 | 2.67 | 3 | -7.2 | - | - | real |
| ECs5438 | - | - | - | - | 18 | -6.5 | - | - | real |
| ECs5440 | - | - | 68 | 2.63 | 5 | -5.1 | - | - | real |
| ECs5441 | - | - | 139 | 6.16 | - | - | - | - | real |
| ECs5442 | - | - | 280 | 6.58 | 4 | -6.2 | - | - | real |
| ECs5451 | - | - | - | - | 18 | -6.5 | - | - | real |
| ECs5458 | - | - | 229 | 1.29 | 3 | -5.7 | - | - | real |
| ECs5460 | - | - | 140 | 4.05 | 20 | -3.4 | - | - | real |
| ECs5463 | - | - | 173 | 3.15 | - | - | - | - | real |
| ECs5465 | 1.542 | 1E-04 | 270 | 6.74 | - | - | - | - | real |
| ECs5482 | - | - | 115 | 0.95 | 5 | -6.3 | - | - | real |
| ECs5484 | - | - | 66 | 0.90 | - | - | - | - | real |
| ECs5495 | - | - | 96 | 2.30 | - | - | - | - | real |
| ECs5496 | - | - | 201 | 3.07 | 21 | -4 | - | - | real |
| ECs5498 | - | - | 215 | 2.38 | - | - | - | - | real |
| ECs5530 | - | - | 277 | 3.26 | 3 | -3.1 | - | - | real |
| ECs5531 | - | - | 280 | 5.71 | 4 | -3.8 | - | - | real |
| ECs5532 | - | - | - | - | 20 | -5.3 | - | - | real |
| ECs5556 | - | - | 194 | 4.07 | 20 | -3.2 | - | - | real |
| ECs5575 | - | - | 191 | 1.39 | 4 | -6.5 | - | - | real |
| ECs5580 | - | - | 257 | 2.39 | 7 | -4.9 | - | - | real |
| ECs5586 | - | - | 279 | 4.19 | 4 | -3.7 | 1 | -20.3 | real |
| ECs5591 | - | - | 262 | 2.99 | - | - | - | - | real |
| ECs5595 | - | - | 219 | 0.99 | 4 | -6.1 | - | - | real |
