# Supplementary material for: Discovery of numerous novel small genes in the intergenic regions of the Escherichia coli O157:H7 Sakai genome
Source: PLoS One. 2017 Sep 13;12(9):e0184119. doi: 10.1371/journal.pone.0184119 (PMC5597208; doi:10.1371/journal.pone.0184119)
Supplement: S6 Table — The mean value of the two biological replicates of transcriptome and translatome counts of the BHI control and the LB condition are shown. The log-fold change was calculated and differential gene expression was determined using edgeR. Transcriptional or translational changes are considered significant, when they show a p-value of ≤ 0.05 and a false discovery rate (FDR) of ≤ 0.1. Significant changes in LB compared to BHI control are highlighted in gray. Only genes with significant changes on transcriptional and/or translational level are listed. (DOCX) [file pone.0184119.s008.docx]

| gene name | counts transcriptome BHI^*^ | counts transcriptome LB^*^ | log-fold change | *p*-value | FDR | counts translatome BHI* | counts translatome LB* | log-fold change | *p*-value | FDR |
| --- | --- | --- | --- | --- | --- | --- | --- | --- | --- | --- |
| XECs002 | 11 | 2 | -2,389 | 0,006 | 0,077 | 27 | 7 | -2,030 | 0,002 | 0,024 |
| XECs003 | 5 | 11 | 1,266 | 0,101 | 0,505 | 6 | 31 | 2,446 | 1,65E-04 | 0,004 |
| XECs004 | 4 | 6 | 0,570 | 0,586 | 1,000 | 6 | 19 | 1,705 | 0,011 | 0,091 |
| XECs006 | 77 | 35 | -1,146 | 0,026 | 0,214 | 88 | 26 | -1,778 | 0,001 | 0,012 |
| XECs007 | 140 | 79 | -0,825 | 0,091 | 0,486 | 116 | 275 | 1,227 | 0,011 | 0,086 |
| XECs017 | 39 | 9 | -2,163 | 3,29E-04 | 0,008 | 34 | 13 | -1,381 | 0,019 | 0,127 |
| XECs018 | 161 | 19 | -3,113 | 9,91E-09 | 1,09E-06 | 190 | 66 | -1,547 | 0,002 | 0,023 |
| XECs019 | 20 | 4 | -2,287 | 0,001 | 0,023 | 41 | 16 | -1,374 | 0,014 | 0,103 |
| XECs020 | 38 | 6 | -2,761 | 1,58E-05 | 0,001 | 71 | 30 | -1,253 | 0,015 | 0,107 |
| XECs021 | 32 | 2 | -3,918 | 2,37E-07 | 1,99E-05 | 29 | 7 | -2,135 | 0,001 | 0,015 |
| XECs022 | 35 | 3 | -3,742 | 2,55E-07 | 2,12E-05 | 28 | 6 | -2,196 | 0,001 | 0,013 |
| XECs028 | 3 | 1 | -1,223 | 0,490 | 1,000 | 3 | 41 | 3,694 | 1,18E-07 | 7,83E-06 |
| XECs029 | 27 | 41 | 0,583 | 0,284 | 0,839 | 156 | 64 | -1,319 | 0,007 | 0,067 |
| XECs032 | 169 | 54 | -1,640 | 0,001 | 0,019 | 90 | 61 | -0,588 | 0,242 | 0,612 |
| XECs033 | 83 | 17 | -2,322 | 2,09E-05 | 0,001 | 83 | 54 | -0,643 | 0,201 | 0,544 |
| XECs034 | 35 | 4 | -3,276 | 1,92E-06 | 1,17E-04 | 28 | 14 | -0,991 | 0,100 | 0,362 |
| XECs037 | 3 | 13 | 2,070 | 0,009 | 0,100 | 30 | 98 | 1,697 | 0,001 | 0,015 |
| XECs043 | 6 | 1 | -2,322 | 0,046 | 0,316 | 38 | 4 | -3,232 | 1,46E-06 | 6,75E-05 |
| XECs045 | 26 | 8 | -1,777 | 0,005 | 0,064 | 74 | 46 | -0,714 | 0,158 | 0,458 |
| XECs056 | 32 | 9 | -1,875 | 0,002 | 0,035 | 48 | 44 | -0,166 | 0,756 | 1,000 |
| XECs057 | 22 | 7 | -1,740 | 0,008 | 0,092 | 31 | 31 | 0,000 | 1,000 | 1,000 |
| XECs058 | 191 | 48 | -1,986 | 7,57E-05 | 0,002 | 395 | 83 | -2,280 | 4,25E-06 | 1,67E-04 |
| XECs062 | 45 | 103 | 1,546 | 0,002 | 0,039 | 199 | 194 | -0,064 | 0,901 | 1,000 |
| XECs063 | 16 | 2 | -2,879 | 4,62E-04 | 0,011 | 21 | 18 | -0,245 | 0,743 | 1,000 |
| XECs066 | 39 | 182 | 2,234 | 1,17E-05 | 0,001 | 62 | 503 | 3,005 | 3,81E-09 | 4,04E-07 |
| XECs068 | 42 | 18 | -1,218 | 0,028 | 0,230 | 93 | 35 | -1,422 | 0,005 | 0,052 |
| XECs069 | 11 | 78 | 2,869 | 5,87E-07 | 4,18E-05 | 33 | 205 | 2,628 | 4,32E-07 | 2,35E-05 |
| XECs070 | 11 | 133 | 3,576 | 4,25E-10 | 6,70E-08 | 34 | 401 | 3,550 | 2,38E-11 | 4,99E-09 |
| XECs071 | 20 | 42 | 1,084 | 0,049 | 0,330 | 36 | 90 | 1,296 | 0,011 | 0,090 |
| XECs072 | 18 | 35 | 0,974 | 0,084 | 0,458 | 35 | 89 | 1,321 | 0,010 | 0,081 |
| XECs075 | 53 | 21 | -1,352 | 0,012 | 0,130 | 289 | 76 | -1,956 | 7,36E-05 | 0,002 |
| XECs077 | 96 | 234 | 1,281 | 0,008 | 0,094 | 227 | 675 | 1,548 | 0,001 | 0,017 |
| XECs082 | 62 | 13 | -2,245 | 6,60E-05 | 0,002 | 629 | 491 | -0,380 | 0,414 | 0,808 |
| XECs084 | 48 | 50 | 0,044 | 0,953 | 1,000 | 520 | 164 | -1,687 | 4,42E-04 | 0,008 |
| XECs090 | 16 | 19 | 0,246 | 0,720 | 1,000 | 9 | 34 | 1,961 | 0,001 | 0,018 |
| XECs095 | 1475 | 2913 | 0,982 | 0,035 | 0,272 | 20677 | 9302 | -1,176 | 0,012 | 0,091 |
| XECs098 | 61 | 8 | -2,900 | 1,17E-06 | 7,41E-05 | 55 | 38 | -0,543 | 0,292 | 0,661 |
| XECs100 | 33 | 38 | 0,225 | 0,694 | 1,000 | 25 | 63 | 1,305 | 0,013 | 0,097 |
| XECs106 | 12 | 2 | -2,900 | 0,001 | 0,024 | 24 | 17 | -0,487 | 0,445 | 0,808 |
| XECs107 | 8 | 11 | 0,453 | 0,563 | 1,000 | 3 | 16 | 2,552 | 0,001 | 0,017 |
| XECs108 | 23 | 80 | 1,824 | 0,001 | 0,013 | 38 | 56 | 0,540 | 0,309 | 0,692 |
| XECs111 | 871 | 1417 | 0,701 | 0,130 | 0,608 | 882 | 3560 | 1,989 | 3,08E-05 | 0,001 |
| XECs112 | 313 | 48 | -2,718 | 9,97E-08 | 8,87E-06 | 170 | 44 | -1,971 | 8,91E-05 | 0,002 |
| XECs113 | 57 | 85 | 0,567 | 0,258 | 0,808 | 471 | 195 | -1,294 | 0,007 | 0,060 |
| XECs116 | 69 | 116 | 0,759 | 0,123 | 0,582 | 28 | 101 | 1,841 | 3,73E-04 | 0,007 |
| XECs123 | 58 | 5 | -3,504 | 3,78E-08 | 3,66E-06 | 271 | 55 | -2,335 | 3,37E-06 | 1,35E-04 |
| XECs132 | 14 | 39 | 1,503 | 0,008 | 0,098 | 42 | 72 | 0,743 | 0,147 | 0,455 |
| XECs135 | 10 | 4 | -1,409 | 0,088 | 0,476 | 59 | 5 | -3,540 | 2,99E-08 | 2,41E-06 |
| XECs137 | 93 | 102 | 0,141 | 0,782 | 1,000 | 223 | 812 | 1,843 | 1,23E-04 | 0,003 |
| XECs141 | 1 | 58 | 5,679 | 2,85E-12 | 7,14E-10 | 25 | 111 | 2,121 | 4,80E-05 | 0,001 |
| XECs146 | 77 | 19 | -2,003 | 2,00E-04 | 0,005 | 46 | 18 | -1,371 | 0,013 | 0,100 |
| XECs149 | 9 | 8 | -0,259 | 0,798 | 1,000 | 40 | 10 | -2,010 | 0,001 | 0,011 |
| XECs152 | 7 | 8 | 0,203 | 0,886 | 1,000 | 43 | 13 | -1,740 | 0,002 | 0,030 |
| XECs154 | 11 | 2 | -2,709 | 0,003 | 0,049 | 14 | 6 | -1,228 | 0,089 | 0,339 |
| XECs155 | 42 | 10 | -2,131 | 3,15E-04 | 0,008 | 62 | 25 | -1,348 | 0,012 | 0,092 |
| XECs156 | 14 | 13 | -0,054 | 1,000 | 1,000 | 19 | 69 | 1,830 | 0,001 | 0,011 |
| XECs165 | 14 | 244 | 4,163 | 2,41E-13 | 7,78E-11 | 46 | 267 | 2,510 | 8,07E-07 | 4,10E-05 |
| XECs166 | 277 | 101 | -1,459 | 0,003 | 0,040 | 280 | 315 | 0,146 | 0,758 | 1,000 |
| XECs169 | 20 | 11 | -0,819 | 0,195 | 0,734 | 48 | 19 | -1,378 | 0,012 | 0,096 |
| XECs170 | 23 | 62 | 1,414 | 0,008 | 0,093 | 56 | 551 | 3,283 | 2,09E-10 | 3,47E-08 |
| XECs173 | 41 | 11 | -1,869 | 0,001 | 0,024 | 38 | 34 | -0,165 | 0,756 | 1,000 |
| XECs174 | 69 | 15 | -2,230 | 5,74E-05 | 0,002 | 72 | 70 | -0,054 | 0,934 | 1,000 |
| XECs175 | 13 | 1 | -3,544 | 2,05E-04 | 0,005 | 13 | 7 | -0,849 | 0,253 | 0,614 |
| XECs176 | 27 | 6 | -2,147 | 0,001 | 0,018 | 26 | 24 | -0,110 | 0,915 | 1,000 |
| XECs178 | 5 | 1 | -2,040 | 0,102 | 0,505 | 2 | 11 | 2,298 | 0,008 | 0,070 |
| XECs180 | 52 | 10 | -2,351 | 5,48E-05 | 0,002 | 78 | 25 | -1,689 | 0,001 | 0,020 |
| XECs181 | 34 | 29 | -0,229 | 0,695 | 1,000 | 32 | 98 | 1,603 | 0,002 | 0,026 |
| XECs184 | 13 | 39 | 1,613 | 0,005 | 0,066 | 56 | 32 | -0,816 | 0,114 | 0,398 |
| XECs187 | 21 | 4 | -2,322 | 0,001 | 0,020 | 20 | 15 | -0,484 | 0,467 | 0,808 |
| XECs192 | 45 | 16 | -1,485 | 0,008 | 0,090 | 66 | 46 | -0,543 | 0,286 | 0,657 |
| XECs193 | 33 | 8 | -2,028 | 0,001 | 0,019 | 50 | 38 | -0,404 | 0,435 | 0,808 |
| XECs194 | 24 | 13 | -0,904 | 0,136 | 0,612 | 291 | 98 | -1,597 | 0,001 | 0,015 |
| XECs195 | 46 | 15 | -1,609 | 0,004 | 0,057 | 299 | 105 | -1,532 | 0,002 | 0,021 |
| XECs198 | 58 | 4 | -3,817 | 6,61E-09 | 7,60E-07 | 102 | 10 | -3,424 | 4,21E-09 | 4,34E-07 |
| XECs199 | 1466 | 1434 | -0,033 | 0,944 | 1,000 | 5249 | 2034 | -1,391 | 0,003 | 0,034 |
| XECs203a/b | 90 | 14 | -2,673 | 1,47E-06 | 9,11E-05 | 15 | 16 | 0,117 | 0,873 | 1,000 |
| XECs205 | 15 | 3 | -2,527 | 0,001 | 0,026 | 22 | 13 | -0,777 | 0,219 | 0,577 |
| XECs208 | 9 | 12 | 0,491 | 0,512 | 1,000 | 16 | 46 | 1,477 | 0,008 | 0,068 |
| XECs210 | 20 | 75 | 1,890 | 4,20E-04 | 0,010 | 36 | 60 | 0,731 | 0,163 | 0,470 |
| XECs212 | 24 | 5 | -2,235 | 0,001 | 0,018 | 89 | 46 | -0,974 | 0,053 | 0,246 |
| XECs213 | 16 | 4 | -1,967 | 0,007 | 0,085 | 83 | 45 | -0,912 | 0,071 | 0,293 |
| XECs218 | 215 | 896 | 2,060 | 1,98E-05 | 0,001 | 585 | 617 | 0,187 | 0,688 | 0,979 |
| XECs219 | 72 | 102 | 0,511 | 0,300 | 0,865 | 148 | 551 | 1,872 | 1,06E-04 | 0,002 |
| XECs220 | 91 | 65 | -0,477 | 0,339 | 0,920 | 956 | 416 | -1,226 | 0,009 | 0,076 |
| XECs229 | 23 | 15 | -0,661 | 0,275 | 0,827 | 113 | 17 | -2,741 | 5,32E-07 | 2,82E-05 |
| XECs230 | 1548 | 404 | -1,937 | 5,14E-05 | 0,002 | 8030 | 2649 | -1,624 | 0,001 | 0,010 |
| XECs235 | 162 | 32 | -2,359 | 5,04E-06 | 2,47E-04 | 104 | 22 | -2,291 | 1,77E-05 | 0,001 |
| XECs238 | 10 | 20 | 0,954 | 0,133 | 0,612 | 28 | 169 | 2,559 | 1,06E-06 | 5,17E-05 |
| XECs243 | 24 | 6 | -1,948 | 0,003 | 0,045 | 78 | 16 | -2,300 | 3,00E-05 | 0,001 |
| XECs245 | 56 | 10 | -2,471 | 2,12E-05 | 0,001 | 189 | 75 | -1,365 | 0,005 | 0,052 |
| XECs246 | 49 | 8 | -2,596 | 1,53E-05 | 0,001 | 181 | 73 | -1,328 | 0,007 | 0,061 |
| XECs247 | 39 | 6 | -2,676 | 2,22E-05 | 0,001 | 142 | 63 | -1,206 | 0,014 | 0,103 |
| XECs248 | 72 | 6 | -3,558 | 8,51E-09 | 9,58E-07 | 424 | 34 | -3,680 | 4,50E-12 | 1,22E-09 |
| XECs249 | 36 | 8 | -2,133 | 4,72E-04 | 0,011 | 17 | 11 | -0,645 | 0,343 | 0,726 |
| XECs250 | 40 | 10 | -2,042 | 0,001 | 0,013 | 22 | 10 | -1,152 | 0,070 | 0,292 |
| XECs253 | 51 | 49 | -0,058 | 0,931 | 1,000 | 278 | 103 | -1,455 | 0,003 | 0,032 |
| XECs254 | 45 | 50 | 0,137 | 0,809 | 1,000 | 260 | 84 | -1,652 | 0,001 | 0,012 |
| XECs257 | 269 | 157 | -0,777 | 0,102 | 0,505 | 88 | 464 | 2,381 | 1,49E-06 | 6,82E-05 |
| XECs265 | 40 | 89 | 1,169 | 0,021 | 0,189 | 69 | 193 | 1,469 | 0,003 | 0,033 |
| XECs267 | 70 | 42 | -0,753 | 0,141 | 0,612 | 208 | 86 | -1,296 | 0,007 | 0,067 |
| XECs269 | 5 | 22 | 2,225 | 0,001 | 0,023 | 16 | 27 | 0,744 | 0,191 | 0,525 |
| XECs276 | 123 | 157 | 0,351 | 0,466 | 1,000 | 450 | 153 | -1,583 | 0,001 | 0,015 |
| XECs277 | 17 | 4 | -2,197 | 0,003 | 0,046 | 19 | 7 | -1,448 | 0,031 | 0,179 |
| XECs278 | 94 | 331 | 1,822 | 1,86E-04 | 0,005 | 172 | 481 | 1,457 | 0,002 | 0,029 |
| XECs279 | 75 | 248 | 1,720 | 4,48E-04 | 0,010 | 878 | 447 | -0,998 | 0,033 | 0,186 |
| XECs280 | 16 | 6 | -1,351 | 0,052 | 0,345 | 67 | 25 | -1,442 | 0,007 | 0,061 |
| XECs283 | 52 | 187 | 1,857 | 1,97E-04 | 0,005 | 150 | 678 | 2,151 | 9,70E-06 | 3,30E-04 |
| XECs286 | 20 | 24 | 0,298 | 0,632 | 1,000 | 41 | 121 | 1,552 | 0,002 | 0,028 |
| XECs294 | 40 | 61 | 0,625 | 0,226 | 0,794 | 57 | 168 | 1,530 | 0,002 | 0,026 |
| XECs296 | 13044 | 12215 | -0,095 | 0,836 | 1,000 | 44729 | 119281 | 1,391 | 0,003 | 0,034 |
| XECs301 | 43 | 7 | -2,686 | 1,52E-05 | 0,001 | 19 | 14 | -0,513 | 0,445 | 0,808 |
| XECs302 | 31 | 17 | -0,882 | 0,125 | 0,591 | 44 | 15 | -1,617 | 0,004 | 0,045 |
| XECs305 | 100 | 494 | 2,302 | 2,99E-06 | 1,71E-04 | 277 | 1449 | 2,364 | 1,17E-06 | 5,57E-05 |
| XECs306 | 8 | 23 | 1,477 | 0,020 | 0,185 | 45 | 318 | 2,791 | 5,26E-08 | 3,97E-06 |
| XECs307 | 6 | 6 | 0,000 | 1,000 | 1,000 | 18 | 60 | 1,706 | 0,002 | 0,022 |
| XECs309 | 9 | 18 | 1,071 | 0,101 | 0,505 | 16 | 53 | 1,683 | 0,002 | 0,028 |
| XECs310 | 9 | 13 | 0,606 | 0,398 | 0,982 | 21 | 82 | 1,935 | 2,54E-04 | 0,005 |
| XECs311 | 14 | 23 | 0,711 | 0,241 | 0,808 | 39 | 144 | 1,852 | 2,74E-04 | 0,006 |
| XECs312 | 10 | 19 | 0,990 | 0,122 | 0,581 | 22 | 111 | 2,298 | 0,122 | 0,581 |
| XECs313 | 21 | 58 | 1,482 | 0,006 | 0,075 | 97 | 119 | 0,271 | 0,576 | 0,878 |
| XECs314 | 5 | 38 | 3,043 | 3,84E-06 | 2,06E-04 | 51 | 59 | 0,200 | 0,712 | 0,983 |
| XECs316 | 73 | 177 | 1,281 | 0,009 | 0,099 | 353 | 797 | 1,226 | 0,009 | 0,076 |
| XECs317 | 101 | 959 | 3,252 | 1,69E-10 | 2,82E-08 | 120 | 1355 | 3,476 | 1,18E-11 | 2,73E-09 |
| XECs318 | 341 | 2808 | 3,044 | 1,05E-09 | 1,52E-07 | 898 | 3284 | 1,846 | 1,02E-04 | 0,002 |
| XECs320 | 31 | 5 | -2,580 | 8,86E-05 | 0,003 | 50 | 14 | -1,889 | 0,001 | 0,014 |
| XECs322 | 116 | 40 | -1,534 | 0,002 | 0,039 | 264 | 146 | -0,880 | 0,064 | 0,276 |
| XECs328 | 51 | 5 | -3,467 | 9,46E-08 | 8,55E-06 | 15 | 16 | 0,072 | 0,935 | 1,000 |
| XECs331 | 17 | 3 | -2,663 | 0,001 | 0,015 | 26 | 17 | -0,676 | 0,269 | 0,633 |
| XECs332 | 5 | 10 | 1,057 | 0,190 | 0,717 | 48 | 16 | -1,631 | 0,004 | 0,040 |
| XECs334 | 6 | 8 | 0,530 | 0,554 | 1,000 | 29 | 9 | -1,673 | 0,007 | 0,063 |
| XECs336 | 87 | 236 | 1,447 | 0,003 | 0,044 | 105 | 215 | 1,006 | 0,037 | 0,203 |
| XECs337 | 10 | 2 | -2,566 | 0,006 | 0,080 | 33 | 55 | 0,720 | 0,177 | 0,497 |
| XECs343 | 500 | 162 | -1,626 | 0,001 | 0,015 | 317 | 184 | -0,812 | 0,086 | 0,330 |
| XECs345 | 70 | 11 | -2,646 | 3,71E-06 | 2,01E-04 | 51 | 10 | -2,419 | 3,99E-05 | 0,001 |
| XECs348 | 25 | 116 | 2,237 | 2,07E-05 | 0,001 | 216 | 713 | 1,698 | 3,76E-04 | 0,007 |
| XECs350 | 21 | 7 | -1,639 | 0,013 | 0,134 | 62 | 5 | -3,771 | 5,71E-09 | 5,62E-07 |
| XECs360 | 27 | 34 | 0,337 | 0,555 | 1,000 | 76 | 181 | 1,232 | 0,011 | 0,090 |
| XECs361 | 28 | 35 | 0,347 | 0,539 | 1,000 | 76 | 181 | 1,236 | 0,011 | 0,088 |
| XECs363 | 0 | 4 | 5,044 | 0,014 | 0,142 | 4 | 38 | 3,184 | 2,01E-06 | 8,72E-05 |
| XECs365 | 41 | 10 | -2,078 | 4,53E-04 | 0,010 | 63 | 28 | -1,216 | 0,019 | 0,127 |
| XECs371 | 22 | 16 | -0,457 | 0,460 | 1,000 | 118 | 40 | -1,594 | 0,002 | 0,022 |
| XECs372 | 11 | 6 | -0,795 | 0,302 | 0,869 | 88 | 33 | -1,436 | 0,005 | 0,050 |
| XECs377 | 81 | 6 | -3,719 | 1,67E-09 | 2,34E-07 | 247 | 33 | -2,920 | 1,96E-08 | 1,65E-06 |
| XECs383 | 31 | 7 | -2,104 | 0,001 | 0,017 | 69 | 91 | 0,375 | 0,456 | 0,808 |
| XECs384 | 30 | 6 | -2,298 | 3,41E-04 | 0,008 | 69 | 91 | 0,385 | 0,443 | 0,808 |
| XECs390 | 186 | 120 | -0,629 | 0,190 | 0,720 | 407 | 182 | -1,185 | 0,013 | 0,097 |
| XECs391 | 122 | 72 | -0,765 | 0,119 | 0,571 | 313 | 117 | -1,449 | 0,003 | 0,032 |
| XECs393 | 838 | 321 | -1,385 | 0,003 | 0,049 | 246 | 198 | -0,334 | 0,478 | 0,808 |
| XECs394 | 1324 | 507 | -1,387 | 0,003 | 0,047 | 1358 | 644 | -1,100 | 0,018 | 0,124 |
| XECs395 | 271 | 70 | -1,952 | 7,71E-05 | 0,002 | 1973 | 558 | -1,847 | 1,04E-04 | 0,002 |
| XECs396 | 49 | 16 | -1,638 | 0,003 | 0,048 | 241 | 12 | -4,395 | 3,51E-14 | 1,50E-11 |
| XECs397 | 1356 | 336 | -2,013 | 2,76E-05 | 0,001 | 756 | 329 | -1,225 | 0,009 | 0,077 |
| XECs399 | 19 | 2 | -3,132 | 9,48E-05 | 0,003 | 63 | 12 | -2,464 | 1,67E-05 | 0,001 |
| XECs402 | 246 | 57 | -2,118 | 2,23E-05 | 0,001 | 113 | 84 | -0,451 | 0,360 | 0,758 |
| XECs406 | 57 | 259 | 2,191 | 1,17E-05 | 0,001 | 245 | 836 | 1,748 | 2,56E-04 | 0,005 |
| XECs410 | 96 | 31 | -1,644 | 0,001 | 0,026 | 210 | 76 | -1,489 | 0,002 | 0,030 |
| XECs411 | 38 | 11 | -1,777 | 0,002 | 0,038 | 34 | 70 | 1,027 | 0,048 | 0,234 |
| XECs412 | 39 | 14 | -1,503 | 0,008 | 0,098 | 38 | 74 | 0,955 | 0,063 | 0,274 |
| XECs415 | 28 | 7 | -1,981 | 0,002 | 0,030 | 21 | 34 | 0,668 | 0,232 | 0,603 |
| XECs416 | 23 | 6 | -2,040 | 0,002 | 0,036 | 38 | 51 | 0,418 | 0,440 | 0,808 |
| XECs423 | 49 | 14 | -1,836 | 0,001 | 0,022 | 141 | 59 | -1,274 | 0,010 | 0,081 |
| XECs424 | 16 | 3 | -2,619 | 0,001 | 0,018 | 51 | 27 | -0,951 | 0,077 | 0,305 |
| XECs426 | 476 | 764 | 0,681 | 0,144 | 0,612 | 377 | 866 | 1,177 | 0,012 | 0,096 |
| XECs429 | 56 | 31 | -0,875 | 0,096 | 0,501 | 57 | 17 | -1,804 | 0,001 | 0,016 |
| XECs430 | 22 | 6 | -1,853 | 0,005 | 0,066 | 22 | 9 | -1,302 | 0,042 | 0,217 |
| XECs432 | 6 | 3 | -0,848 | 0,418 | 1,000 | 24 | 8 | -1,654 | 0,010 | 0,084 |
| XECs435 | 765 | 42 | -4,202 | 6,42E-15 | 2,95E-12 | 2201 | 344 | -2,702 | 3,93E-08 | 3,10E-06 |
| XECs436 | 113 | 869 | 2,948 | 4,39E-09 | 5,21E-07 | 307 | 1132 | 1,861 | 1,01E-04 | 0,002 |
| XECs437 | 31 | 3 | -3,568 | 1,12E-06 | 7,25E-05 | 62 | 30 | -1,092 | 0,035 | 0,195 |
| XECs438 | 30 | 3 | -3,497 | 2,01E-06 | 1,22E-04 | 61 | 29 | -1,118 | 0,031 | 0,179 |
| XECs439 | 78 | 5 | -3,930 | 5,33E-10 | 8,16E-08 | 97 | 52 | -0,921 | 0,066 | 0,282 |
| XECs442 | 80 | 40 | -1,007 | 0,048 | 0,326 | 232 | 62 | -1,937 | 9,37E-05 | 0,002 |
| XECs443 | 6 | 9 | 0,575 | 0,492 | 1,000 | 29 | 139 | 2,257 | 1,25E-05 | 4,14E-04 |
| XECs446 | 38 | 24 | -0,661 | 0,230 | 0,803 | 76 | 27 | -1,530 | 0,004 | 0,041 |
| XECs447 | 199 | 35 | -2,524 | 9,75E-07 | 6,40E-05 | 98 | 62 | -0,676 | 0,171 | 0,485 |
| XECs448 | 21 | 94 | 2,190 | 4,09E-05 | 0,001 | 19 | 105 | 2,465 | 4,42E-06 | 1,70E-04 |
| XECs455 | 10 | 5 | -1,058 | 0,190 | 0,717 | 32 | 6 | -2,515 | 1,19E-04 | 0,003 |
| XECs457 | 31 | 25 | -0,315 | 0,590 | 1,000 | 124 | 28 | -2,160 | 2,98E-05 | 0,001 |
| XECs459 | 6 | 8 | 0,438 | 0,649 | 1,000 | 83 | 30 | -1,504 | 0,004 | 0,039 |
| ECs0001 | 151 | 56 | -1,424 | 0,004 | 0,027 | 111 | 206 | 0,888 | 0,064 | 0,264 |
| ECs0239 | 39 | 6 | -2,657 | 2,56E-05 | 4,14E-04 | 51 | 12 | -2,076 | 2,73E-04 | 0,004 |
| ECs0275 | 12 | 33 | 1,450 | 0,013 | 0,067 | 44 | 38 | -0,213 | 0,702 | 0,897 |
| ECs0439 | 75 | 18 | -2,092 | 1,17E-04 | 0,002 | 119 | 65 | -0,871 | 0,077 | 0,292 |
| ECs0513 | 248 | 50 | -2,322 | 4,12E-06 | 8,52E-05 | 420 | 177 | -1,246 | 0,009 | 0,066 |
| ECs0665 | 238 | 658 | 1,467 | 0,002 | 0,016 | 1385 | 2281 | 0,720 | 0,120 | 0,376 |
| ECs0728 | 1040 | 1712 | 0,720 | 0,120 | 0,318 | 5268 | 12316 | 1,225 | 0,009 | 0,065 |
| ECs0805 | 6 | 10 | 0,725 | 0,357 | 0,616 | 7 | 36 | 2,447 | 9,41E-05 | 0,002 |
| ECs0966 | 41 | 91 | 1,158 | 0,022 | 0,099 | 106 | 483 | 2,187 | 8,26E-06 | 2,46E-04 |
| ECs1000 | 125 | 292 | 1,227 | 0,010 | 0,057 | 260 | 309 | 0,252 | 0,594 | 0,835 |
| ECs1037 | 35 | 102 | 1,561 | 0,002 | 0,017 | 29 | 214 | 2,878 | 4,05E-08 | 2,54E-06 |
| ECs1100 | 6 | 7 | 0,218 | 0,880 | 0,980 | 8 | 31 | 2,029 | 0,001 | 0,013 |
| ECs1127 | 187 | 30 | -2,659 | 3,41E-07 | 9,70E-06 | 163 | 95 | -0,781 | 0,106 | 0,350 |
| ECs1144 | 43 | 3 | -4,038 | 1,75E-08 | 6,52E-07 | 31 | 13 | -1,278 | 0,029 | 0,156 |
| ECs1145 | 699 | 77 | -3,179 | 4,67E-10 | 2,65E-08 | 3696 | 532 | -2,796 | 1,33E-08 | 9,88E-07 |
| ECs1170 | 7 | 20 | 1,567 | 0,018 | 0,085 | 8 | 55 | 2,762 | 3,85E-06 | 1,29E-04 |
| ECs1172 | 11 | 15 | 0,509 | 0,459 | 0,715 | 17 | 44 | 1,392 | 0,012 | 0,084 |
| ECs1186 | 79 | 184 | 1,228 | 0,012 | 0,062 | 278 | 204 | -0,444 | 0,348 | 0,656 |
| ECs1569 | 6 | 3 | -1,222 | 0,221 | 0,463 | 36 | 4 | -3,110 | 3,86E-06 | 1,29E-04 |
| ECs1624 | 14 | 2 | -2,733 | 0,001 | 0,010 | 15 | 7 | -1,142 | 0,102 | 0,341 |
| ECs1673 | 21 | 21 | 0,035 | 1,000 | 1,000 | 6 | 21 | 1,909 | 0,005 | 0,040 |
| ECs1856 | 110 | 49 | -1,158 | 0,020 | 0,092 | 1424 | 386 | -1,952 | 4,60E-05 | 0,001 |
| ECs1960 | 14 | 4 | -1,910 | 0,013 | 0,065 | 13 | 16 | 0,308 | 0,668 | 0,881 |
| ECs2024 | 7 | 22 | 1,707 | 0,009 | 0,052 | 23 | 58 | 1,361 | 0,011 | 0,077 |
| ECs2025 | 8 | 48 | 2,643 | 1,30E-05 | 2,34E-04 | 14 | 58 | 2,093 | 1,89E-04 | 0,003 |
| ECs2031 | 13 | 23 | 0,786 | 0,198 | 0,433 | 16 | 101 | 2,687 | 9,99E-07 | 4,06E-05 |
| ECs2049 | 19 | 5 | -1,900 | 0,006 | 0,037 | 19 | 21 | 0,109 | 0,897 | 0,981 |
| ECs2085 | 6 | 4 | -0,570 | 0,586 | 0,812 | 21 | 3 | -2,974 | 9,66E-05 | 0,002 |
| ECs2139 | 96 | 35 | -1,473 | 0,004 | 0,027 | 117 | 89 | -0,396 | 0,419 | 0,716 |
| ECs2192 | 22 | 7 | -1,635 | 0,012 | 0,061 | 13 | 9 | -0,524 | 0,468 | 0,749 |
| ECs2253 | 5 | 3 | -0,565 | 0,663 | 0,855 | 11 | 31 | 1,461 | 0,014 | 0,093 |
| ECs2282 | 19 | 3 | -2,575 | 0,001 | 0,006 | 11 | 7 | -0,681 | 0,378 | 0,683 |
| ECs2333 | 258 | 132 | -0,972 | 0,042 | 0,155 | 338 | 121 | -1,479 | 0,002 | 0,023 |
| ECs2334 | 302 | 119 | -1,343 | 0,005 | 0,033 | 443 | 146 | -1,605 | 0,001 | 0,011 |
| ECs2497 | 8 | 17 | 1,076 | 0,105 | 0,289 | 17 | 53 | 1,663 | 0,003 | 0,026 |
| ECs2505 | 93 | 11 | -3,132 | 4,86E-08 | 1,65E-06 | 419 | 102 | -2,044 | 2,92E-05 | 0,001 |
| ECs2533 | 337 | 1185 | 1,816 | 1,42E-04 | 0,002 | 979 | 2155 | 1,138 | 0,015 | 0,097 |
| ECs2534 | 162 | 422 | 1,379 | 0,004 | 0,026 | 149 | 645 | 2,118 | 1,33E-05 | 3,70E-04 |
| ECs2536 | 221 | 167 | -0,405 | 0,395 | 0,655 | 1146 | 369 | -1,636 | 0,001 | 0,008 |
| ECs2622 | 23 | 10 | -1,264 | 0,042 | 0,155 | 11 | 45 | 2,020 | 4,86E-04 | 0,007 |
| ECs2653 | 151 | 57 | -1,403 | 0,005 | 0,030 | 720 | 794 | 0,141 | 0,761 | 0,925 |
| ECs2743 | 3 | 4 | 0,466 | 0,802 | 0,938 | 3 | 14 | 2,428 | 0,003 | 0,026 |
| ECs2748 | 59 | 7 | -3,040 | 5,61E-07 | 1,49E-05 | 46 | 24 | -0,935 | 0,083 | 0,305 |
| ECs2755 | 36 | 26 | -0,467 | 0,400 | 0,659 | 137 | 493 | 1,850 | 1,30E-04 | 0,002 |
| ECs2814 | 20 | 3 | -2,938 | 1,22E-04 | 0,002 | 40 | 13 | -1,594 | 0,005 | 0,046 |
| ECs2890 | 6 | 3 | -1,099 | 0,290 | 0,545 | 27 | 8 | -1,804 | 0,004 | 0,038 |
| ECs3003 | 17 | 58 | 1,751 | 0,001 | 0,012 | 131 | 186 | 0,505 | 0,291 | 0,598 |
| ECs3004 | 27 | 63 | 1,207 | 0,022 | 0,096 | 142 | 197 | 0,477 | 0,318 | 0,626 |
| ECs3006 | 19 | 75 | 1,964 | 2,66E-04 | 0,003 | 37 | 88 | 1,247 | 0,014 | 0,095 |
| ECs3087 | 20 | 16 | -0,365 | 0,570 | 0,799 | 64 | 19 | -1,745 | 0,001 | 0,015 |
| ECs3497 | 3 | 4 | 0,652 | 0,633 | 0,838 | 2 | 9 | 2,489 | 0,009 | 0,068 |
| ECs3527 | 22 | 15 | -0,564 | 0,360 | 0,620 | 252 | 69 | -1,864 | 1,56E-04 | 0,003 |
| ECs3690 | 196 | 222 | 0,180 | 0,707 | 0,883 | 3175 | 1422 | -1,159 | 0,013 | 0,088 |
| ECs3891 | 42 | 12 | -1,779 | 0,002 | 0,016 | 16 | 14 | -0,146 | 0,869 | 0,971 |
| ECs3931 | 52 | 96 | 0,876 | 0,080 | 0,242 | 102 | 462 | 2,184 | 8,67E-06 | 2,54E-04 |
| ECs3948 | 3123 | 2010 | -0,636 | 0,169 | 0,392 | 9026 | 3987 | -1,179 | 0,011 | 0,079 |
| ECs4140 | 28 | 114 | 2,040 | 8,77E-05 | 0,001 | 95 | 262 | 1,460 | 0,003 | 0,026 |
| ECs4177 | 7204 | 4006 | -0,846 | 0,067 | 0,218 | 8743 | 20517 | 1,231 | 0,008 | 0,063 |
| ECs4415 | 88 | 56 | -0,664 | 0,184 | 0,414 | 92 | 218 | 1,240 | 0,010 | 0,075 |
| ECs4441 | 7149 | 1384 | -2,369 | 9,44E-07 | 2,33E-05 | 52365 | 9014 | -2,538 | 1,74E-07 | 8,60E-06 |
| ECs4511 | 1771 | 841 | -1,074 | 0,021 | 0,095 | 5213 | 2272 | -1,198 | 0,010 | 0,074 |
| ECs4587 | 38 | 6 | -2,761 | 1,58E-05 | 2,72E-04 | 47 | 20 | -1,264 | 0,021 | 0,122 |
| ECs4638 | 2551 | 1231 | -1,052 | 0,024 | 0,103 | 32550 | 14486 | -1,168 | 0,012 | 0,083 |
| ECs4644 | 32 | 72 | 1,180 | 0,023 | 0,100 | 13 | 215 | 4,032 | 1,31E-12 | 2,80E-10 |
| ECs4958 | 14 | 3 | -2,124 | 0,007 | 0,041 | 23 | 11 | -1,276 | 0,077 | 0,292 |
| ECs4997 | 74 | 31 | -1,242 | 0,016 | 0,079 | 939 | 774 | -0,279 | 0,548 | 0,808 |
| ECs5224 | 27 | 47 | 0,808 | 0,131 | 0,336 | 49 | 153 | 1,636 | 0,001 | 0,013 |
| ECs5360 | 16 | 5 | -1,802 | 0,012 | 0,063 | 6 | 5 | -0,282 | 0,856 | 0,968 |
| ECs5391 | 93 | 223 | 1,261 | 0,009 | 0,052 | 292 | 276 | -0,081 | 0,866 | 0,971 |
| ECs5406 | 3 | 3 | 0,000 | 1,000 | 1,000 | 6 | 23 | 2,040 | 0,002 | 0,023 |
| ECs5411 | 7 | 4 | -0,870 | 0,362 | 0,621 | 76 | 14 | -2,482 | 8,64E-06 | 2,54E-04 |
| ECs5440 | 19 | 79 | 2,078 | 1,18E-04 | 0,002 | 13 | 65 | 2,356 | 2,97E-05 | 0,001 |
| ECs5441 | 29 | 7 | -2,111 | 0,001 | 0,009 | 19 | 10 | -0,879 | 0,171 | 0,451 |
| ECs5442 | 15 | 3 | -2,478 | 0,002 | 0,015 | 19 | 2 | -3,132 | 9,48E-05 | 0,002 |
| ECs5458 | 18 | 5 | -1,782 | 0,011 | 0,058 | 57 | 21 | -1,457 | 0,007 | 0,055 |
| ECs5463 | 15 | 14 | -0,150 | 0,866 | 0,974 | 46 | 18 | -1,372 | 0,013 | 0,088 |
| ECs5465 | 49 | 14 | -1,783 | 0,002 | 0,013 | 34 | 62 | 0,864 | 0,096 | 0,331 |
| ECs5495 | 16 | 9 | -0,903 | 0,177 | 0,405 | 31 | 86 | 1,468 | 0,004 | 0,040 |
| ECs5530 | 459 | 583 | 0,344 | 0,460 | 0,716 | 1693 | 4919 | 1,539 | 0,001 | 0,013 |
| ECs5531 | 36 | 8 | -2,224 | 2,99E-04 | 0,003 | 34 | 37 | 0,102 | 0,875 | 0,973 |
| ECs5556 | 89 | 191 | 1,105 | 0,022 | 0,099 | 312 | 413 | 0,403 | 0,390 | 0,694 |
| ECs5586 | 27 | 24 | -0,199 | 0,752 | 0,914 | 66 | 17 | -1,981 | 2,96E-04 | 0,005 |

*Mean counts of two biological replicates normalized to the smallest library are shown.
