# Supplementary material for: Discovery of numerous novel small genes in the intergenic regions of the Escherichia coli O157:H7 Sakai genome
Source: PLoS One. 2017 Sep 13;12(9):e0184119. doi: 10.1371/journal.pone.0184119 (PMC5597208; doi:10.1371/journal.pone.0184119)
Supplement: S7 Table — The mean value of the two biological replicates of transcriptome and translatome counts of the BHI control and the stress condition COS are shown. The log-fold change was calculated and differential gene expression was determined using the software edgeR. Transcriptional or translational changes are considered significant, when they show a p-value of ≤ 0.05 and an FDR of ≤ 0.1. Significant changes in BHI COS compared to control are highlighted in gray. Only genes with significant changes on transcriptional and/or translational level are listed. (DOCX) [file pone.0184119.s009.docx]

| gene name | counts transcriptome control^*^ | counts transcriptome stress^*^ | log-fold change | *p*-value | FDR | counts translatome control* | counts translatome stress* | log-fold change | *p*-value | FDR |
| --- | --- | --- | --- | --- | --- | --- | --- | --- | --- | --- |
| XECs002 | 7 | 5 | -0,474 | 0,631 | 1,000 | 18 | 1 | -4,816 | 2,42E-06 | 2,51E-05 |
| XECs003 | 3 | 2 | -0,942 | 0,551 | 1,000 | 6 | 1 | -3,169 | 0,016 | 0,067 |
| XECs004 | 2 | 2 | -0,386 | 1,000 | 1,000 | 5 | 1 | -3,034 | 0,024 | 0,098 |
| XECs005 | 10 | 4 | -1,408 | 0,088 | 0,608 | 8 | 1 | -3,608 | 0,003 | 0,015 |
| XECs006 | 48 | 30 | -0,659 | 0,215 | 1,000 | 50 | 1 | -5,462 | 2,77E-11 | 6,71E-10 |
| XECs007 | 87 | 163 | 0,914 | 0,060 | 0,486 | 56 | 18 | -1,670 | 0,002 | 0,013 |
| XECs008 | 49 | 116 | 1,236 | 0,013 | 0,194 | 1001 | 123 | -3,022 | 1,87E-09 | 3,30E-08 |
| XECs010 | 6 | 4 | -0,446 | 0,708 | 1,000 | 13 | 1 | -3,543 | 2,05E-04 | 0,001 |
| XECs011 | 10 | 18 | 0,801 | 0,219 | 1,000 | 19 | 2 | -3,169 | 7,35E-05 | 0,001 |
| XECs012 | 654 | 658 | 0,010 | 0,985 | 1,000 | 625 | 50 | -3,653 | 3,52E-12 | 9,53E-11 |
| XECs013 | 256 | 318 | 0,316 | 0,503 | 1,000 | 275 | 20 | -3,769 | 6,03E-12 | 1,61E-10 |
| XECs014 | 2431 | 3782 | 0,639 | 0,167 | 0,845 | 4856 | 346 | -3,809 | 1,47E-13 | 5,17E-12 |
| XECs015 | 24 | 14 | -0,741 | 0,219 | 1,000 | 18 | 1 | -4,009 | 1,00E-05 | 9,42E-05 |
| XECs017 | 24 | 6 | -1,977 | 0,003 | 0,056 | 71 | 0 | -9,151 | 7,00E-16 | 3,53E-14 |
| XECs018 | 100 | 20 | -2,313 | 1,52E-05 | 0,001 | 217 | 4 | -5,900 | 8,30E-19 | 6,45E-17 |
| XECs019 | 13 | 3 | -2,265 | 0,006 | 0,105 | 63 | 1 | -6,657 | 8,05E-14 | 2,96E-12 |
| XECs020 | 24 | 4 | -2,703 | 1,43E-04 | 0,006 | 89 | 1 | -6,306 | 2,43E-15 | 1,13E-13 |
| XECs021 | 20 | 2 | -3,629 | 1,37E-05 | 0,001 | 23 | 1 | -4,360 | 7,11E-07 | 8,16E-06 |
| XECs022 | 22 | 2 | -3,733 | 6,43E-06 | 4,94E-04 | 23 | 1 | -4,360 | 7,11E-07 | 8,16E-06 |
| XECs023 | 3 | 6 | 0,972 | 0,328 | 1,000 | 22 | 1 | -4,296 | 1,17E-06 | 1,29E-05 |
| XECs025 | 2 | 2 | 0,001 | 1,000 | 1,000 | 48 | 2 | -4,887 | 2,43E-10 | 4,99E-09 |
| XECs026 | 281 | 191 | -0,553 | 0,241 | 1,000 | 319 | 8 | -5,294 | 3,19E-18 | 2,29E-16 |
| XECs027 | 36 | 37 | 0,061 | 0,939 | 1,000 | 68 | 9 | -2,888 | 8,28E-07 | 9,40E-06 |
| XECs029 | 17 | 34 | 1,039 | 0,067 | 0,527 | 54 | 24 | -1,195 | 0,025 | 0,099 |
| XECs030 | 3 | 1 | -1,473 | 0,334 | 1,000 | 12 | 1 | -3,429 | 3,96E-04 | 0,003 |
| XECs031 | 135 | 11 | -3,662 | 1,99E-10 | 5,86E-08 | 137 | 11 | -3,683 | 1,58E-10 | 3,38E-09 |
| XECs032 | 104 | 7 | -3,973 | 7,19E-11 | 2,28E-08 | 170 | 10 | -4,069 | 2,63E-12 | 7,28E-11 |
| XECs033 | 52 | 5 | -3,331 | 1,87E-07 | 2,28E-05 | 179 | 1 | -8,162 | 1,10E-21 | 1,54E-19 |
| XECs034 | 22 | 2 | -3,346 | 2,15E-05 | 0,001 | 66 | 1 | -6,724 | 3,81E-14 | 1,49E-12 |
| XECs036 | 6 | 6 | -0,122 | 1,000 | 1,000 | 16 | 3 | -2,618 | 0,001 | 0,006 |
| XECs037 | 2 | 1 | -0,530 | 1,000 | 1,000 | 15 | 2 | -3,217 | 2,11E-04 | 0,002 |
| XECs038 | 8 | 3 | -1,537 | 0,095 | 0,639 | 44 | 2 | -4,745 | 9,95E-10 | 1,85E-08 |
| XECs039 | 8 | 3 | -1,537 | 0,095 | 0,639 | 44 | 3 | -4,070 | 1,28E-08 | 1,99E-07 |
| XECs040 | 20 | 9 | -1,140 | 0,074 | 0,547 | 284 | 10 | -4,807 | 3,98E-16 | 2,08E-14 |
| XECs041 | 14 | 7 | -0,934 | 0,185 | 0,913 | 43 | 3 | -4,020 | 2,05E-08 | 3,05E-07 |
| XECs042 | 1 | 1 | 0,001 | 1,000 | 1,000 | 17 | 2 | -2,967 | 2,69E-04 | 0,002 |
| XECs043 | 3 | 1 | -2,321 | 0,157 | 0,807 | 41 | 1 | -6,039 | 5,64E-11 | 1,29E-09 |
| XECs044 | 83 | 70 | -0,255 | 0,614 | 1,000 | 85 | 6 | -3,910 | 3,68E-10 | 7,36E-09 |
| XECs045 | 16 | 15 | -0,140 | 0,873 | 1,000 | 45 | 4 | -3,621 | 9,46E-08 | 1,28E-06 |
| XECs046 | 33 | 18 | -0,847 | 0,135 | 0,779 | 100 | 9 | -3,455 | 3,46E-09 | 5,86E-08 |
| XECs047 | 349 | 310 | -0,172 | 0,714 | 1,000 | 219 | 48 | -2,186 | 1,40E-05 | 1,28E-04 |
| XECs048 | 196 | 261 | 0,414 | 0,383 | 1,000 | 165 | 40 | -2,040 | 5,70E-05 | 4,64E-04 |
| XECs049 | 191 | 255 | 0,418 | 0,379 | 1,000 | 136 | 39 | -1,811 | 3,53E-04 | 0,002 |
| XECs050 | 36 | 23 | -0,622 | 0,262 | 1,000 | 112 | 7 | -4,080 | 2,23E-11 | 5,49E-10 |
| XECs051 | 11 | 19 | 0,783 | 0,219 | 1,000 | 18 | 6 | -1,564 | 0,021 | 0,089 |
| XECs054 | 2 | 2 | 0,001 | 1,000 | 1,000 | 21 | 2 | -3,665 | 1,06E-05 | 9,91E-05 |
| XECs055 | 22 | 15 | -0,563 | 0,360 | 1,000 | 262 | 49 | -2,427 | 1,59E-06 | 1,72E-05 |
| XECs056 | 20 | 5 | -1,936 | 0,005 | 0,093 | 106 | 4 | -4,863 | 2,29E-13 | 7,68E-12 |
| XECs057 | 14 | 4 | -1,723 | 0,022 | 0,261 | 40 | 4 | -3,449 | 4,38E-07 | 5,23E-06 |
| XECs058 | 118 | 61 | -0,949 | 0,054 | 0,447 | 176 | 3 | -5,815 | 9,87E-18 | 6,45E-16 |
| XECs059 | 6 | 18 | 1,689 | 0,014 | 0,199 | 59 | 6 | -3,257 | 1,51E-07 | 1,97E-06 |
| XECs060 | 21 | 30 | 0,489 | 0,397 | 1,000 | 241 | 40 | -2,583 | 4,51E-07 | 5,37E-06 |
| XECs061 | 13 | 40 | 1,595 | 0,005 | 0,101 | 37 | 3 | -3,569 | 3,73E-07 | 4,53E-06 |
| XECs062 | 22 | 18 | -0,254 | 0,697 | 1,000 | 142 | 11 | -3,735 | 8,98E-11 | 2,00E-09 |
| XECs064 | 44 | 34 | -0,375 | 0,488 | 1,000 | 117 | 1 | -6,701 | 2,22E-17 | 1,38E-15 |
| XECs066 | 24 | 8 | -1,539 | 0,015 | 0,207 | 94 | 6 | -3,940 | 1,61E-10 | 3,42E-09 |
| XECs067 | 184 | 379 | 1,045 | 0,028 | 0,304 | 248 | 74 | -1,749 | 3,63E-04 | 0,003 |
| XECs068 | 26 | 75 | 1,515 | 0,004 | 0,079 | 47 | 20 | -1,211 | 0,026 | 0,103 |
| XECs069 | 7 | 2 | -1,639 | 0,098 | 0,640 | 51 | 1 | -5,491 | 2,06E-11 | 5,09E-10 |
| XECs070 | 7 | 1 | -3,405 | 0,006 | 0,116 | 146 | 0 | -10,190 | 2,42E-21 | 3,07E-19 |
| XECs071 | 12 | 47 | 1,960 | 0,001 | 0,019 | 30 | 38 | 0,346 | 0,536 | 0,988 |
| XECs072 | 11 | 42 | 1,971 | 0,001 | 0,022 | 30 | 38 | 0,346 | 0,536 | 0,988 |
| XECs073 | 17 | 64 | 1,906 | 4,86E-04 | 0,015 | 31 | 40 | 0,373 | 0,500 | 0,947 |
| XECs075 | 33 | 21 | -0,660 | 0,241 | 1,000 | 272 | 5 | -5,727 | 3,61E-19 | 3,04E-17 |
| XECs077 | 59 | 11 | -2,475 | 1,76E-05 | 0,001 | 400 | 10 | -5,374 | 4,16E-19 | 3,41E-17 |
| XECs080 | 11 | 15 | 0,462 | 0,511 | 1,000 | 26 | 10 | -1,366 | 0,025 | 0,099 |
| XECs082 | 39 | 48 | 0,303 | 0,575 | 1,000 | 1083 | 127 | -3,089 | 8,87E-10 | 1,65E-08 |
| XECs084 | 30 | 25 | -0,236 | 0,693 | 1,000 | 152 | 18 | -3,103 | 1,25E-08 | 1,95E-07 |
| XECs085 | 42 | 48 | 0,193 | 0,728 | 1,000 | 183 | 3 | -6,119 | 1,31E-18 | 1,01E-16 |
| XECs088 | 20 | 9 | -1,185 | 0,066 | 0,516 | 19 | 5 | -1,899 | 0,006 | 0,030 |
| XECs089 | 10 | 23 | 1,161 | 0,062 | 0,490 | 42 | 4 | -3,351 | 4,99E-07 | 5,91E-06 |
| XECs090 | 10 | 19 | 0,919 | 0,151 | 0,806 | 8 | 1 | -3,608 | 0,003 | 0,015 |
| XECs092 | 31 | 33 | 0,092 | 0,896 | 1,000 | 100 | 13 | -2,979 | 1,17E-07 | 1,56E-06 |
| XECs093 | 20 | 22 | 0,105 | 0,901 | 1,000 | 349 | 34 | -3,353 | 1,92E-10 | 4,02E-09 |
| XECs094 | 92 | 110 | 0,260 | 0,601 | 1,000 | 190 | 7 | -4,737 | 7,18E-15 | 3,12E-13 |
| XECs095 | 913 | 983 | 0,108 | 0,816 | 1,000 | 7267 | 230 | -4,980 | 6,52E-20 | 6,14E-18 |
| XECs097 | 2 | 2 | 0,001 | 1,000 | 1,000 | 10 | 1 | -4,017 | 3,78E-04 | 0,003 |
| XECs098 | 38 | 6 | -2,741 | 1,84E-05 | 0,001 | 68 | 4 | -4,034 | 7,36E-10 | 1,40E-08 |
| XECs099 | 6 | 2 | -1,913 | 0,071 | 0,534 | 23 | 1 | -5,177 | 1,49E-07 | 1,95E-06 |
| XECs100 | 20 | 8 | -1,399 | 0,031 | 0,327 | 15 | 1 | -3,699 | 7,93E-05 | 0,001 |
| XECs101 | 42 | 27 | -0,634 | 0,241 | 1,000 | 57 | 2 | -4,747 | 8,59E-11 | 1,93E-09 |
| XECs102 | 16 | 19 | 0,293 | 0,663 | 1,000 | 9 | 1 | -2,937 | 0,005 | 0,024 |
| XECs103 | 24 | 9 | -1,453 | 0,021 | 0,253 | 76 | 1 | -6,070 | 3,73E-14 | 1,47E-12 |
| XECs104 | 70 | 20 | -1,826 | 0,001 | 0,020 | 853 | 3 | -8,091 | 1,11E-30 | 8,95E-28 |
| XECs105 | 42 | 10 | -2,111 | 3,55E-04 | 0,012 | 718 | 2 | -8,398 | 1,58E-30 | 1,12E-27 |
| XECs106 | 8 | 2 | -1,842 | 0,053 | 0,446 | 22 | 2 | -3,766 | 5,02E-06 | 4,98E-05 |
| XECs108 | 14 | 31 | 1,118 | 0,055 | 0,450 | 37 | 11 | -1,737 | 0,003 | 0,016 |
| XECs109 | 3 | 2 | -0,304 | 1,000 | 1,000 | 34 | 1 | -4,921 | 5,61E-09 | 9,20E-08 |
| XECs110 | 2 | 2 | 0,388 | 1,000 | 1,000 | 32 | 1 | -4,812 | 1,53E-08 | 2,35E-07 |
| XECs111 | 540 | 84 | -2,689 | 7,80E-08 | 1,05E-05 | 998 | 35 | -4,847 | 2,79E-18 | 2,01E-16 |
| XECs112 | 194 | 95 | -1,024 | 0,034 | 0,345 | 76 | 29 | -1,385 | 0,008 | 0,037 |
| XECs113 | 36 | 21 | -0,787 | 0,157 | 0,807 | 923 | 14 | -6,081 | 6,60E-24 | 1,28E-21 |
| XECs114 | 104 | 33 | -1,666 | 0,001 | 0,031 | 129 | 2 | -5,918 | 1,49E-16 | 8,22E-15 |
| XECs117 | 3 | 4 | 0,653 | 0,633 | 1,000 | 23 | 2 | -3,411 | 1,34E-05 | 1,23E-04 |
| XECs118 | 32 | 18 | -0,865 | 0,128 | 0,761 | 159 | 1 | -7,143 | 9,41E-20 | 8,73E-18 |
| XECs119 | 10 | 7 | -0,506 | 0,530 | 1,000 | 62 | 2 | -4,857 | 2,72E-11 | 6,64E-10 |
| XECs120 | 10 | 8 | -0,335 | 0,707 | 1,000 | 64 | 2 | -4,903 | 1,67E-11 | 4,18E-10 |
| XECs121 | 927 | 483 | -0,940 | 0,044 | 0,395 | 728 | 45 | -4,027 | 4,81E-14 | 1,86E-12 |
| XECs122 | 879 | 535 | -0,716 | 0,124 | 0,749 | 688 | 48 | -3,851 | 3,62E-13 | 1,17E-11 |
| XECs123 | 36 | 43 | 0,260 | 0,640 | 1,000 | 126 | 3 | -5,334 | 3,46E-15 | 1,57E-13 |
| XECs124 | 13 | 10 | -0,446 | 0,541 | 1,000 | 26 | 5 | -2,469 | 2,72E-04 | 0,002 |
| XECs125 | 26 | 55 | 1,106 | 0,037 | 0,367 | 71 | 10 | -2,811 | 1,15E-06 | 1,27E-05 |
| XECs126 | 25 | 51 | 1,041 | 0,052 | 0,445 | 56 | 6 | -3,194 | 2,70E-07 | 3,36E-06 |
| XECs127 | 3 | 4 | 0,215 | 1,000 | 1,000 | 22 | 1 | -5,144 | 1,93E-07 | 2,46E-06 |
| XECs128 | 22 | 11 | -0,991 | 0,108 | 0,678 | 193 | 15 | -3,721 | 2,93E-11 | 7,04E-10 |
| XECs129 | 6 | 3 | -1,099 | 0,290 | 1,000 | 89 | 6 | -3,976 | 1,82E-10 | 3,84E-09 |
| XECs130 | 30 | 20 | -0,593 | 0,301 | 1,000 | 28 | 3 | -3,143 | 1,21E-05 | 1,12E-04 |
| XECs131 | 10 | 3 | -1,622 | 0,054 | 0,447 | 8 | 1 | -2,760 | 0,010 | 0,046 |
| XECs132 | 8 | 12 | 0,579 | 0,435 | 1,000 | 64 | 12 | -2,451 | 1,67E-05 | 1,51E-04 |
| XECs133 | 12 | 3 | -2,207 | 0,007 | 0,129 | 34 | 2 | -4,370 | 3,41E-08 | 4,94E-07 |
| XECs135 | 6 | 2 | -1,913 | 0,071 | 0,534 | 27 | 1 | -4,564 | 1,34E-07 | 1,77E-06 |
| XECs136 | 5 | 1 | -2,039 | 0,102 | 0,647 | 17 | 1 | -4,732 | 4,44E-06 | 4,42E-05 |
| XECs138 | 77 | 37 | -1,064 | 0,037 | 0,368 | 409 | 6 | -6,059 | 1,23E-21 | 1,69E-19 |
| XECs139 | 16 | 2 | -3,309 | 1,19E-04 | 0,005 | 6 | 1 | -3,169 | 0,016 | 0,067 |
| XECs140 | 8 | 5 | -0,812 | 0,347 | 1,000 | 26 | 5 | -2,469 | 2,72E-04 | 0,002 |
| XECs141 | 1 | 0 | -2,321 | 1,000 | 1,000 | 21 | 1 | -4,230 | 1,97E-06 | 2,07E-05 |
| XECs143 | 37 | 59 | 0,660 | 0,204 | 0,967 | 192 | 3 | -6,188 | 5,49E-19 | 4,38E-17 |
| XECs145 | 23 | 16 | -0,565 | 0,351 | 1,000 | 316 | 1 | -8,133 | 2,68E-25 | 6,46E-23 |
| XECs146 | 48 | 36 | -0,418 | 0,433 | 1,000 | 25 | 5 | -2,403 | 2,97E-04 | 0,002 |
| XECs147 | 10 | 5 | -1,056 | 0,190 | 0,925 | 12 | 2 | -2,837 | 0,002 | 0,010 |
| XECs148 | 9 | 4 | -1,331 | 0,112 | 0,692 | 8 | 1 | -3,608 | 0,003 | 0,015 |
| XECs149 | 6 | 4 | -0,446 | 0,708 | 1,000 | 14 | 4 | -1,909 | 0,013 | 0,058 |
| XECs150 | 9 | 1 | -2,937 | 0,005 | 0,090 | 5 | 0 | -5,209 | 0,008 | 0,039 |
| XECs151 | 7 | 3 | -1,440 | 0,126 | 0,753 | 29 | 1 | -4,668 | 5,47E-08 | 7,67E-07 |
| XECs152 | 4 | 3 | -0,651 | 0,633 | 1,000 | 16 | 3 | -2,366 | 0,002 | 0,011 |
| XECs153 | 4 | 2 | -1,343 | 0,285 | 1,000 | 5 | 0 | -5,357 | 0,005 | 0,025 |
| XECs154 | 7 | 0 | -5,832 | 0,001 | 0,020 | 15 | 0 | -6,918 | 1,19E-06 | 1,30E-05 |
| XECs155 | 26 | 15 | -0,836 | 0,157 | 0,807 | 65 | 4 | -3,979 | 1,28E-09 | 2,33E-08 |
| XECs156 | 8 | 4 | -1,163 | 0,181 | 0,901 | 9 | 1 | -3,019 | 0,003 | 0,017 |
| XECs160 | 64 | 172 | 1,421 | 0,004 | 0,079 | 98 | 41 | -1,263 | 0,012 | 0,057 |
| XECs161 | 6 | 20 | 1,681 | 0,012 | 0,183 | 14 | 3 | -2,375 | 0,003 | 0,018 |
| XECs162 | 4 | 20 | 2,438 | 0,001 | 0,023 | 9 | 3 | -1,545 | 0,070 | 0,240 |
| XECs163 | 106 | 49 | -1,103 | 0,027 | 0,301 | 494 | 6 | -6,333 | 3,48E-23 | 5,99E-21 |
| XECs164 | 5 | 4 | -0,499 | 0,687 | 1,000 | 74 | 0 | -9,201 | 3,94E-16 | 2,06E-14 |
| XECs165 | 9 | 1 | -3,786 | 0,001 | 0,033 | 38 | 1 | -5,081 | 1,24E-09 | 2,26E-08 |
| XECs166 | 172 | 219 | 0,354 | 0,459 | 1,000 | 100 | 18 | -2,498 | 4,07E-06 | 4,09E-05 |
| XECs167 | 7 | 4 | -0,974 | 0,289 | 1,000 | 29 | 5 | -2,653 | 7,62E-05 | 0,001 |
| XECs168 | 16 | 7 | -1,177 | 0,083 | 0,583 | 117 | 2 | -6,164 | 1,58E-16 | 8,61E-15 |
| XECs169 | 12 | 6 | -1,107 | 0,135 | 0,779 | 107 | 2 | -6,035 | 7,42E-16 | 3,70E-14 |
| XECs170 | 14 | 7 | -0,986 | 0,158 | 0,812 | 31 | 2 | -3,871 | 3,50E-07 | 4,27E-06 |
| XECs172 | 11 | 4 | -1,617 | 0,042 | 0,391 | 16 | 0 | -7,011 | 6,12E-07 | 7,09E-06 |
| XECs173 | 25 | 5 | -2,292 | 0,001 | 0,019 | 31 | 1 | -5,613 | 3,36E-09 | 5,70E-08 |
| XECs174 | 42 | 6 | -2,904 | 4,97E-06 | 4,05E-04 | 42 | 2 | -4,291 | 8,06E-09 | 1,29E-07 |
| XECs175 | 8 | 1 | -3,699 | 0,002 | 0,045 | 6 | 1 | -3,169 | 0,016 | 0,067 |
| XECs176 | 17 | 3 | -2,705 | 0,001 | 0,017 | 13 | 0 | -6,714 | 4,86E-06 | 4,82E-05 |
| XECs177 | 3 | 2 | -0,691 | 0,751 | 1,000 | 162 | 15 | -3,421 | 7,11E-10 | 1,36E-08 |
| XECs187 | 13 | 42 | 1,739 | 0,002 | 0,055 | 25 | 4 | -2,605 | 1,66E-04 | 0,001 |
| XECs188a/b | 10 | 3 | -1,695 | 0,042 | 0,390 | 22 | 2 | -3,733 | 6,43E-06 | 6,27E-05 |
| XECs189 | 8 | 2 | -2,229 | 0,025 | 0,285 | 16 | 2 | -3,264 | 1,58E-04 | 0,001 |
| XECs192 | 28 | 11 | -1,403 | 0,020 | 0,245 | 79 | 3 | -4,652 | 8,44E-12 | 2,21E-10 |
| XECs193 | 21 | 7 | -1,532 | 0,019 | 0,239 | 70 | 3 | -4,728 | 1,63E-11 | 4,10E-10 |
| XECs194 | 15 | 8 | -0,939 | 0,174 | 0,875 | 191 | 4 | -5,529 | 2,67E-17 | 1,65E-15 |
| XECs195 | 28 | 16 | -0,847 | 0,146 | 0,788 | 199 | 5 | -5,279 | 9,88E-17 | 5,56E-15 |
| XECs196 | 7 | 4 | -0,974 | 0,289 | 1,000 | 18 | 2 | -3,438 | 5,16E-05 | 4,22E-04 |
| XECs197 | 22 | 4 | -2,608 | 2,68E-04 | 0,010 | 129 | 1 | -6,841 | 3,98E-18 | 2,84E-16 |
| XECs198 | 36 | 9 | -1,964 | 0,001 | 0,030 | 85 | 1 | -7,088 | 5,83E-16 | 2,99E-14 |
| XECs199 | 907 | 349 | -1,376 | 0,004 | 0,073 | 5032 | 131 | -5,261 | 2,21E-21 | 2,86E-19 |
| XECs200 | 476 | 242 | -0,976 | 0,038 | 0,368 | 2667 | 140 | -4,254 | 8,84E-16 | 4,32E-14 |
| XECs201 | 51 | 85 | 0,743 | 0,140 | 0,779 | 98 | 20 | -2,277 | 2,07E-05 | 1,85E-04 |
| XECs202 | 63 | 110 | 0,798 | 0,107 | 0,670 | 122 | 22 | -2,496 | 2,70E-06 | 2,78E-05 |
| XECs203a/b | 56 | 6 | -3,194 | 2,70E-07 | 3,19E-05 | 9 | 7 | -0,356 | 0,695 | 1,000 |
| XECs204 | 7 | 8 | 0,204 | 0,886 | 1,000 | 16 | 2 | -2,922 | 3,52E-04 | 0,002 |
| XECs205 | 9 | 4 | -1,330 | 0,112 | 0,692 | 12 | 2 | -2,898 | 0,001 | 0,008 |
| XECs206 | 7 | 2 | -1,639 | 0,098 | 0,640 | 22 | 2 | -3,733 | 6,43E-06 | 6,27E-05 |
| XECs207 | 66 | 62 | -0,078 | 0,891 | 1,000 | 237 | 2 | -6,798 | 2,49E-21 | 3,09E-19 |
| XECs208 | 5 | 4 | -4,617 | 0,846 | 1,000 | 28 | 1 | -4,617 | 8,52E-08 | 1,16E-06 |
| XECs210 | 13 | 36 | 1,497 | 0,010 | 0,156 | 31 | 12 | -1,419 | 0,016 | 0,069 |
| XECs212 | 15 | 8 | -0,895 | 0,189 | 0,925 | 41 | 4 | -3,299 | 7,86E-07 | 8,94E-06 |
| XECs213 | 10 | 5 | -0,908 | 0,257 | 1,000 | 38 | 4 | -3,207 | 1,72E-06 | 1,83E-05 |
| XECs214 | 18 | 15 | -0,268 | 0,700 | 1,000 | 127 | 4 | -4,944 | 3,11E-14 | 1,24E-12 |
| XECs215 | 4 | 14 | 1,725 | 0,022 | 0,261 | 41 | 12 | -1,804 | 0,002 | 0,010 |
| XECs216 | 65 | 22 | -1,556 | 0,003 | 0,072 | 504 | 7 | -6,143 | 1,36E-22 | 2,22E-20 |
| XECs218 | 133 | 38 | -1,822 | 3,33E-04 | 0,012 | 504 | 16 | -5,010 | 4,09E-18 | 2,90E-16 |
| XECs220 | 56 | 21 | -1,408 | 0,009 | 0,144 | 2527 | 9 | -8,193 | 3,59E-35 | 8,66E-32 |
| XECs221 | 4 | 15 | 2,062 | 0,006 | 0,113 | 101 | 12 | -3,119 | 3,91E-08 | 5,61E-07 |
| XECs222 | 3 | 13 | 2,323 | 0,004 | 0,085 | 42 | 8 | -2,447 | 5,92E-05 | 4,78E-04 |
| XECs223 | 47 | 34 | -0,464 | 0,383 | 1,000 | 60 | 3 | -4,253 | 5,74E-10 | 1,11E-08 |
| XECs224 | 24 | 20 | -0,261 | 0,678 | 1,000 | 18 | 2 | -3,478 | 3,93E-05 | 3,31E-04 |
| XECs225 | 5 | 5 | 0,001 | 1,000 | 1,000 | 12 | 0 | -6,538 | 1,50E-05 | 1,36E-04 |
| XECs227 | 18 | 8 | -1,208 | 0,069 | 0,530 | 139 | 7 | -4,391 | 6,65E-13 | 2,03E-11 |
| XECs228 | 20 | 10 | -0,990 | 0,117 | 0,719 | 167 | 8 | -4,357 | 2,96E-13 | 9,74E-12 |
| XECs229 | 14 | 17 | 0,236 | 0,749 | 1,000 | 44 | 11 | -2,037 | 4,75E-04 | 0,003 |
| XECs230 | 958 | 103 | -3,221 | 2,35E-10 | 6,74E-08 | 1992 | 61 | -5,026 | 1,03E-19 | 9,44E-18 |
| XECs232 | 10 | 31 | 1,694 | 0,005 | 0,095 | 7 | 2 | -2,131 | 0,036 | 0,137 |
| XECs233 | 23 | 99 | 2,101 | 7,02E-05 | 0,003 | 10 | 4 | -1,408 | 0,088 | 0,284 |
| XECs234 | 17 | 57 | 1,782 | 0,001 | 0,032 | 7 | 4 | -0,869 | 0,362 | 0,824 |
| XECs235 | 35 | 26 | -0,405 | 0,470 | 1,000 | 67 | 4 | -4,012 | 9,17E-10 | 1,70E-08 |
| XECs236 | 47 | 40 | -0,231 | 0,672 | 1,000 | 94 | 10 | -3,208 | 2,73E-08 | 3,99E-07 |
| XECs237 | 58 | 47 | -0,301 | 0,564 | 1,000 | 177 | 14 | -3,699 | 4,90E-11 | 1,13E-09 |
| XECs238 | 7 | 1 | -3,405 | 0,006 | 0,116 | 45 | 1 | -5,325 | 1,13E-10 | 2,47E-09 |
| XECs239 | 9 | 1 | -3,019 | 0,003 | 0,068 | 20 | 0 | -7,330 | 5,20E-08 | 7,33E-07 |
| XECs240 | 14 | 12 | -0,219 | 0,785 | 1,000 | 96 | 2 | -5,878 | 4,73E-15 | 2,09E-13 |
| XECs241 | 6 | 4 | -0,756 | 0,452 | 1,000 | 66 | 1 | -6,724 | 3,81E-14 | 1,49E-12 |
| XECs242 | 34 | 22 | -0,651 | 0,633 | 1,000 | 653 | 20 | -4,277 | 8,69E-05 | 0,001 |
| XECs243 | 15 | 9 | -0,761 | 0,268 | 1,000 | 59 | 1 | -6,563 | 2,28E-13 | 7,67E-12 |
| XECs245 | 35 | 17 | -1,078 | 0,056 | 0,462 | 95 | 11 | -3,087 | 6,28E-08 | 8,73E-07 |
| XECs246 | 31 | 14 | -1,115 | 0,055 | 0,450 | 92 | 11 | -3,115 | 5,66E-08 | 7,92E-07 |
| XECs247 | 25 | 12 | -1,021 | 0,091 | 0,622 | 68 | 8 | -3,056 | 2,80E-07 | 3,47E-06 |
| XECs248 | 45 | 6 | -2,987 | 2,48E-06 | 2,26E-04 | 202 | 1 | -7,488 | 1,18E-21 | 1,64E-19 |
| XECs249 | 22 | 4 | -2,609 | 2,68E-04 | 0,010 | 16 | 0 | -7,011 | 6,12E-07 | 7,09E-06 |
| XECs250 | 24 | 4 | -2,547 | 2,46E-04 | 0,009 | 13 | 1 | -4,391 | 4,32E-05 | 3,60E-04 |
| XECs251 | 37 | 73 | 0,969 | 0,059 | 0,481 | 248 | 2 | -6,863 | 1,07E-21 | 1,52E-19 |
| XECs252 | 51 | 92 | 0,851 | 0,090 | 0,617 | 133 | 24 | -2,493 | 2,36E-06 | 2,47E-05 |
| XECs253 | 32 | 52 | 0,722 | 0,171 | 0,864 | 588 | 19 | -4,940 | 4,63E-18 | 3,24E-16 |
| XECs254 | 28 | 55 | 0,959 | 0,070 | 0,534 | 554 | 10 | -5,773 | 1,83E-21 | 2,43E-19 |
| XECs255 | 9 | 340 | 5,303 | 2,05E-18 | 1,24E-14 | 29 | 14 | -1,017 | 0,082 | 0,265 |
| XECs257 | 167 | 57 | -1,556 | 0,002 | 0,042 | 96 | 3 | -4,942 | 3,32E-13 | 1,08E-11 |
| XECs258 | 36 | 7 | -2,446 | 9,41E-05 | 0,004 | 25 | 1 | -4,451 | 3,42E-07 | 4,17E-06 |
| XECs259 | 107 | 27 | -1,974 | 1,50E-04 | 0,006 | 145 | 6 | -4,688 | 6,15E-14 | 2,32E-12 |
| XECs260 | 93 | 24 | -1,978 | 1,74E-04 | 0,007 | 131 | 6 | -4,542 | 3,39E-13 | 1,10E-11 |
| XECs261 | 70 | 38 | -0,887 | 0,083 | 0,586 | 43 | 5 | -3,072 | 1,85E-06 | 1,96E-05 |
| XECs262 | 19 | 14 | -0,488 | 0,445 | 1,000 | 21 | 1 | -4,195 | 2,56E-06 | 2,64E-05 |
| XECs263 | 78 | 26 | -1,607 | 0,002 | 0,051 | 230 | 6 | -5,353 | 1,92E-17 | 1,20E-15 |
| XECs264 | 14 | 5 | -1,409 | 0,053 | 0,447 | 33 | 3 | -3,634 | 6,37E-07 | 7,37E-06 |
| XECs265 | 24 | 17 | -0,536 | 0,372 | 1,000 | 118 | 4 | -4,832 | 1,15E-13 | 4,13E-12 |
| XECs266 | 36 | 18 | -0,994 | 0,076 | 0,556 | 136 | 6 | -4,596 | 1,81E-13 | 6,26E-12 |
| XECs267 | 44 | 69 | 0,655 | 0,201 | 0,961 | 119 | 30 | -2,660 | 3,24E-07 | 3,98E-06 |
| XECs268 | 7 | 27 | 2,035 | 0,001 | 0,039 | 20 | 7 | -1,461 | 0,026 | 0,104 |
| XECs270 | 62 | 97 | 0,638 | 0,199 | 0,960 | 353 | 13 | -4,749 | 2,30E-16 | 1,24E-14 |
| XECs271 | 3 | 4 | 0,653 | 0,633 | 1,000 | 9 | 2 | -2,488 | 0,009 | 0,043 |
| XECs273 | 30 | 19 | -0,692 | 0,226 | 1,000 | 56 | 2 | -5,096 | 2,83E-11 | 6,84E-10 |
| XECs274 | 28 | 38 | 0,441 | 0,426 | 1,000 | 27 | 4 | -2,716 | 7,72E-05 | 0,001 |
| XECs275 | 35 | 66 | 0,924 | 0,074 | 0,550 | 37 | 3 | -3,550 | 4,42E-07 | 5,27E-06 |
| XECs276 | 76 | 133 | 0,802 | 0,101 | 0,647 | 203 | 66 | -1,625 | 0,001 | 0,006 |
| XECs277 | 11 | 2 | -2,708 | 0,003 | 0,070 | 16 | 1 | -3,795 | 4,30E-05 | 3,60E-04 |
| XECs278 | 58 | 14 | -2,092 | 1,89E-04 | 0,007 | 346 | 4 | -6,576 | 1,41E-22 | 2,26E-20 |
| XECs279 | 47 | 58 | 0,319 | 0,544 | 1,000 | 982 | 66 | -3,891 | 1,45E-13 | 5,10E-12 |
| XECs280 | 10 | 15 | 0,605 | 0,382 | 1,000 | 56 | 1 | -5,639 | 4,28E-12 | 1,15E-10 |
| XECs281 | 212 | 82 | -1,374 | 0,005 | 0,091 | 401 | 3 | -7,003 | 2,35E-24 | 4,71E-22 |
| XECs282 | 206 | 72 | -1,524 | 0,002 | 0,045 | 401 | 3 | -7,003 | 2,35E-24 | 4,71E-22 |
| XECs283 | 32 | 118 | 1,874 | 2,68E-04 | 0,010 | 89 | 43 | -1,038 | 0,040 | 0,149 |
| XECs284 | 3 | 4 | 0,653 | 0,633 | 1,000 | 30 | 2 | -4,187 | 1,69E-07 | 2,18E-06 |
| XECs285 | 21 | 20 | -0,069 | 0,950 | 1,000 | 39 | 1 | -5,119 | 8,64E-10 | 1,61E-08 |
| XECs286 | 12 | 59 | 2,287 | 5,69E-05 | 0,003 | 25 | 23 | -0,118 | 0,869 | 1,000 |
| XECs287 | 14 | 62 | 2,190 | 9,21E-05 | 0,004 | 22 | 53 | 1,298 | 0,016 | 0,069 |
| XECs290 | 5 | 2 | -1,508 | 0,203 | 0,961 | 9 | 2 | -2,488 | 0,009 | 0,043 |
| XECs291 | 89 | 31 | -1,540 | 0,003 | 0,062 | 110 | 2 | -6,075 | 4,60E-16 | 2,39E-14 |
| XECs292 | 46 | 22 | -1,092 | 0,044 | 0,395 | 90 | 1 | -6,323 | 2,01E-15 | 9,45E-14 |
| XECs293 | 5 | 11 | 1,053 | 0,175 | 0,879 | 6 | 1 | -3,169 | 0,016 | 0,067 |
| XECs294 | 25 | 38 | 0,632 | 0,251 | 1,000 | 36 | 12 | -1,554 | 0,007 | 0,037 |
| XECs296 | 8070 | 20762 | 1,365 | 0,004 | 0,072 | 40382 | 9455 | -2,093 | 1,18E-05 | 1,09E-04 |
| XECs297 | 46 | 25 | -0,888 | 0,099 | 0,642 | 62 | 3 | -4,312 | 3,13E-10 | 6,32E-09 |
| XECs298 | 36 | 20 | -0,859 | 0,124 | 0,748 | 47 | 3 | -4,149 | 6,00E-09 | 9,80E-08 |
| XECs299 | 30 | 17 | -0,856 | 0,136 | 0,779 | 60 | 3 | -4,504 | 1,73E-10 | 3,66E-09 |
| XECs300 | 69 | 27 | -1,365 | 0,009 | 0,149 | 105 | 4 | -4,857 | 2,48E-13 | 8,26E-12 |
| XECs301 | 26 | 7 | -1,978 | 0,002 | 0,049 | 14 | 2 | -3,118 | 3,80E-04 | 0,003 |
| XECs302 | 19 | 18 | -0,076 | 0,945 | 1,000 | 23 | 1 | -5,208 | 1,15E-07 | 1,54E-06 |
| XECs304 | 108 | 44 | -1,285 | 0,010 | 0,162 | 491 | 5 | -6,728 | 1,98E-24 | 4,11E-22 |
| XECs305 | 62 | 18 | -1,816 | 0,001 | 0,024 | 754 | 2 | -8,470 | 6,00E-31 | 5,17E-28 |
| XECs306 | 6 | 4 | -0,633 | 0,560 | 1,000 | 48 | 3 | -3,943 | 1,22E-08 | 1,91E-07 |
| XECs307 | 4 | 1 | -1,873 | 0,152 | 0,806 | 23 | 1 | -5,177 | 1,49E-07 | 1,95E-06 |
| XECs308 | 10 | 5 | -0,981 | 0,212 | 0,995 | 34 | 4 | -3,047 | 6,36E-06 | 6,22E-05 |
| XECs310 | 6 | 5 | -0,281 | 0,856 | 1,000 | 24 | 1 | -4,421 | 4,35E-07 | 5,20E-06 |
| XECs311 | 9 | 10 | 0,159 | 0,904 | 1,000 | 44 | 3 | -4,053 | 1,50E-08 | 2,31E-07 |
| XECs312 | 6 | 9 | 0,495 | 0,574 | 1,000 | 18 | 2 | -3,478 | 3,93E-05 | 3,31E-04 |
| XECs313 | 13 | 15 | 0,213 | 0,791 | 1,000 | 40 | 10 | -2,040 | 0,001 | 0,004 |
| XECs316 | 45 | 33 | -0,445 | 0,406 | 1,000 | 161 | 11 | -3,921 | 1,13E-11 | 2,91E-10 |
| XECs317 | 62 | 48 | -0,367 | 0,477 | 1,000 | 297 | 37 | -3,016 | 6,52E-09 | 1,06E-07 |
| XECs318 | 211 | 163 | -0,372 | 0,434 | 1,000 | 2236 | 81 | -4,792 | 1,50E-18 | 1,13E-16 |
| XECs319 | 13 | 10 | -0,373 | 0,617 | 1,000 | 21 | 6 | -1,750 | 0,009 | 0,041 |
| XECs320 | 19 | 7 | -1,424 | 0,031 | 0,326 | 32 | 1 | -4,834 | 1,25E-08 | 1,95E-07 |
| XECs321 | 5 | 3 | -0,713 | 0,529 | 1,000 | 58 | 1 | -5,677 | 2,85E-12 | 7,77E-11 |
| XECs322 | 72 | 61 | -0,250 | 0,626 | 1,000 | 119 | 28 | -2,789 | 1,07E-07 | 1,44E-06 |
| XECs323 | 8 | 2 | -1,842 | 0,053 | 0,446 | 58 | 2 | -5,147 | 1,65E-11 | 4,16E-10 |
| XECs324 | 36 | 44 | 0,274 | 0,620 | 1,000 | 27 | 8 | -1,803 | 0,004 | 0,022 |
| XECs325 | 32 | 31 | -0,044 | 0,965 | 1,000 | 31 | 11 | -1,483 | 0,012 | 0,056 |
| XECs326 | 50 | 30 | -0,743 | 0,161 | 0,822 | 290 | 38 | -2,926 | 1,56E-08 | 2,40E-07 |
| XECs327 | 40 | 25 | -0,674 | 0,216 | 1,000 | 281 | 37 | -2,919 | 1,75E-08 | 2,64E-07 |
| XECs328 | 32 | 25 | -0,331 | 0,565 | 1,000 | 18 | 3 | -2,535 | 0,001 | 0,005 |
| XECs329 | 7 | 42 | 2,548 | 3,50E-05 | 0,002 | 20 | 19 | -0,074 | 0,947 | 1,000 |
| XECs330 | 74 | 39 | -0,929 | 0,069 | 0,530 | 106 | 4 | -4,677 | 6,84E-13 | 2,07E-11 |
| XECs331 | 11 | 2 | -2,321 | 0,008 | 0,138 | 11 | 1 | -3,305 | 0,001 | 0,005 |
| XECs332 | 3 | 9 | 1,717 | 0,054 | 0,447 | 53 | 0 | -8,717 | 9,10E-14 | 3,33E-12 |
| XECs333 | 15 | 7 | -1,190 | 0,086 | 0,598 | 13 | 1 | -4,335 | 6,11E-05 | 4,90E-04 |
| XECs334 | 4 | 3 | -0,213 | 1,000 | 1,000 | 14 | 3 | -2,427 | 0,003 | 0,014 |
| XECs336 | 54 | 16 | -1,777 | 0,001 | 0,035 | 199 | 3 | -6,240 | 2,86E-19 | 2,46E-17 |
| XECs337 | 6 | 7 | 0,219 | 0,880 | 1,000 | 26 | 4 | -2,848 | 5,23E-05 | 4,27E-04 |
| XECs338 | 14 | 12 | -0,167 | 0,854 | 1,000 | 21 | 5 | -2,155 | 0,002 | 0,011 |
| XECs339 | 3 | 1 | -1,473 | 0,334 | 1,000 | 14 | 1 | -4,445 | 3,07E-05 | 2,65E-04 |
| XECs341 | 56 | 49 | -0,191 | 0,721 | 1,000 | 86 | 7 | -3,699 | 1,31E-09 | 2,37E-08 |
| XECs343 | 310 | 150 | -1,048 | 0,028 | 0,304 | 916 | 8 | -6,816 | 7,15E-27 | 2,40E-24 |
| XECs344 | 433 | 159 | -1,448 | 0,002 | 0,055 | 462 | 44 | -3,402 | 7,42E-11 | 1,68E-09 |
| XECs345 | 43 | 37 | -0,234 | 0,671 | 1,000 | 22 | 4 | -2,422 | 0,001 | 0,004 |
| XECs346 | 137 | 120 | -0,185 | 0,705 | 1,000 | 731 | 31 | -4,575 | 1,17E-16 | 6,54E-15 |
| XECs348 | 16 | 20 | 0,366 | 0,570 | 1,000 | 98 | 22 | -2,141 | 5,39E-05 | 4,39E-04 |
| XECs349 | 46 | 27 | -0,792 | 0,139 | 0,779 | 118 | 3 | -5,484 | 3,04E-15 | 1,39E-13 |
| XECs350 | 13 | 4 | -1,855 | 0,016 | 0,216 | 44 | 2 | -4,358 | 4,22E-09 | 7,05E-08 |
| XECs351 | 445 | 319 | -0,480 | 0,304 | 1,000 | 1939 | 111 | -4,130 | 4,84E-15 | 2,13E-13 |
| XECs352 | 230 | 172 | -0,419 | 0,377 | 1,000 | 315 | 55 | -2,525 | 5,60E-07 | 6,58E-06 |
| XECs353 | 98 | 37 | -1,413 | 0,005 | 0,102 | 90 | 4 | -4,635 | 3,05E-12 | 8,30E-11 |
| XECs354 | 10 | 24 | 1,224 | 0,048 | 0,420 | 17 | 6 | -1,605 | 0,020 | 0,086 |
| XECs355 | 40 | 34 | -0,236 | 0,675 | 1,000 | 31 | 4 | -3,077 | 9,43E-06 | 8,93E-05 |
| XECs356 | 39 | 59 | 0,585 | 0,261 | 1,000 | 181 | 17 | -3,440 | 4,26E-10 | 8,45E-09 |
| XECs359 | 35 | 16 | -1,122 | 0,047 | 0,419 | 46 | 3 | -4,134 | 6,97E-09 | 1,13E-07 |
| XECs360 | 17 | 4 | -2,009 | 0,006 | 0,105 | 82 | 2 | -5,658 | 5,97E-14 | 2,26E-12 |
| XECs361 | 18 | 5 | -1,781 | 0,011 | 0,163 | 82 | 2 | -5,271 | 2,92E-13 | 9,63E-12 |
| XECs362 | 10 | 9 | -0,230 | 0,814 | 1,000 | 10 | 2 | -2,638 | 0,005 | 0,024 |
| XECs363 | 0 | 1 | 2,322 | 1,000 | 1,000 | 7 | 0 | -5,832 | 0,001 | 0,004 |
| XECs364 | 5 | 5 | -0,147 | 1,000 | 1,000 | 20 | 1 | -4,160 | 3,33E-06 | 3,38E-05 |
| XECs366 | 34 | 17 | -1,015 | 0,074 | 0,546 | 54 | 4 | -3,699 | 2,04E-08 | 3,05E-07 |
| XECs367 | 36 | 13 | -1,439 | 0,012 | 0,186 | 112 | 4 | -4,943 | 9,15E-14 | 3,34E-12 |
| XECs368 | 10 | 4 | -1,408 | 0,088 | 0,608 | 62 | 6 | -3,341 | 6,90E-08 | 9,52E-07 |
| XECs369 | 98 | 17 | -2,552 | 2,86E-06 | 2,57E-04 | 88 | 4 | -4,408 | 1,39E-11 | 3,55E-10 |
| XECs370 | 26 | 29 | 0,161 | 0,804 | 1,000 | 25 | 1 | -4,451 | 3,42E-07 | 4,17E-06 |
| XECs371 | 14 | 7 | -0,986 | 0,158 | 0,812 | 231 | 7 | -5,120 | 1,07E-16 | 6,02E-15 |
| XECs372 | 7 | 3 | -1,335 | 0,167 | 0,845 | 216 | 2 | -7,054 | 2,41E-21 | 3,07E-19 |
| XECs374 | 30 | 33 | 0,162 | 0,793 | 1,000 | 53 | 13 | -2,072 | 2,56E-04 | 0,002 |
| XECs377 | 50 | 10 | -2,292 | 8,47E-05 | 0,004 | 133 | 4 | -5,011 | 1,42E-14 | 5,97E-13 |
| XECs379 | 17 | 10 | -0,787 | 0,233 | 1,000 | 196 | 5 | -5,405 | 4,72E-17 | 2,80E-15 |
| XECs380 | 7 | 7 | 0,106 | 1,000 | 1,000 | 14 | 4 | -1,961 | 9,93E-03 | 0,046 |
| XECs383 | 19 | 10 | -0,989 | 0,122 | 0,741 | 40 | 3 | -3,681 | 1,39E-07 | 1,83E-06 |
| XECs384 | 19 | 9 | -1,148 | 0,076 | 0,556 | 40 | 3 | -3,933 | 4,60E-08 | 6,53E-07 |
| XECs386 | 5 | 1 | -2,186 | 0,069 | 0,530 | 9 | 0 | -6,108 | 1,75E-04 | 0,001 |
| XECs387 | 23 | 23 | 0,032 | 1,000 | 1,000 | 18 | 3 | -2,786 | 3,22E-04 | 0,002 |
| XECs388 | 20 | 19 | -0,073 | 0,948 | 1,000 | 32 | 3 | -3,612 | 7,68E-07 | 8,77E-06 |
| XECs390 | 115 | 98 | -0,237 | 0,630 | 1,000 | 338 | 21 | -3,997 | 3,66E-13 | 1,18E-11 |
| XECs391 | 75 | 61 | -0,297 | 0,559 | 1,000 | 250 | 6 | -5,348 | 1,10E-17 | 7,12E-16 |
| XECs392 | 19 | 16 | -0,252 | 0,714 | 1,000 | 33 | 1 | -4,879 | 8,33E-09 | 1,33E-07 |
| XECs393 | 519 | 363 | -0,515 | 0,271 | 1,000 | 204 | 22 | -3,234 | 1,88E-09 | 3,30E-08 |
| XECs394 | 820 | 496 | -0,723 | 0,120 | 0,732 | 1486 | 22 | -5,122 | 5,24E-20 | 5,05E-18 |
| XECs395 | 168 | 265 | 0,655 | 0,167 | 0,846 | 653 | 222 | -1,554 | 0,001 | 0,007 |
| XECs397 | 839 | 586 | -0,518 | 0,265 | 1,000 | 415 | 62 | -2,739 | 5,97E-08 | 8,34E-07 |
| XECs398 | 6417 | 3988 | -0,685 | 0,138 | 0,779 | 3172 | 386 | -3,039 | 1,06E-09 | 1,95E-08 |
| XECs399 | 12 | 9 | -0,348 | 0,663 | 1,000 | 30 | 2 | -4,211 | 1,38E-07 | 1,81E-06 |
| XECs400 | 35 | 26 | -0,405 | 0,470 | 1,000 | 67 | 4 | -4,012 | 9,17E-10 | 1,70E-08 |
| XECs401 | 23 | 37 | 0,696 | 0,209 | 0,984 | 59 | 13 | -2,214 | 8,92E-05 | 0,001 |
| XECs402 | 152 | 196 | 0,364 | 0,448 | 1,000 | 40 | 3 | -3,933 | 4,60E-08 | 6,53E-07 |
| XECs406 | 35 | 34 | -0,062 | 0,936 | 1,000 | 241 | 20 | -3,618 | 3,63E-11 | 8,59E-10 |
| XECs407 | 32 | 19 | -0,763 | 0,179 | 0,898 | 60 | 5 | -3,539 | 2,66E-08 | 3,90E-07 |
| XECs408 | 21 | 11 | -0,924 | 0,138 | 0,779 | 59 | 2 | -5,172 | 1,27E-11 | 3,26E-10 |
| XECs409 | 6 | 5 | -0,256 | 0,865 | 1,000 | 6 | 1 | -3,292 | 0,010 | 0,046 |
| XECs410 | 59 | 50 | -0,237 | 0,652 | 1,000 | 100 | 21 | -2,271 | 2,08E-05 | 1,86E-04 |
| XECs411 | 24 | 13 | -0,903 | 0,136 | 0,779 | 36 | 6 | -2,682 | 2,89E-05 | 2,50E-04 |
| XECs412 | 24 | 13 | -0,903 | 0,136 | 0,779 | 40 | 7 | -2,579 | 3,47E-05 | 2,96E-04 |
| XECs413 | 18 | 6 | -1,687 | 0,014 | 0,199 | 228 | 4 | -5,971 | 3,36E-19 | 2,86E-17 |
| XECs415 | 18 | 20 | 0,156 | 0,837 | 1,000 | 22 | 3 | -2,789 | 1,50E-04 | 0,001 |
| XECs416 | 14 | 20 | 0,512 | 0,418 | 1,000 | 15 | 4 | -1,873 | 0,011 | 0,051 |
| XECs417 | 151 | 90 | -0,748 | 0,122 | 0,741 | 1145 | 3 | -8,516 | 3,47E-33 | 5,83E-30 |
| XECs418 | 3901 | 5250 | 0,429 | 0,352 | 1,000 | 2324 | 610 | -1,929 | 5,21E-05 | 4,27E-04 |
| XECs419 | 130 | 410 | 1,663 | 0,001 | 0,017 | 445 | 285 | -0,644 | 0,169 | 0,465 |
| XECs420 | 865 | 461 | -0,906 | 0,052 | 0,446 | 1612 | 81 | -4,320 | 6,02E-16 | 3,08E-14 |
| XECs421 | 348 | 287 | -0,279 | 0,551 | 1,000 | 928 | 58 | -3,996 | 4,75E-14 | 1,84E-12 |
| XECs422 | 12 | 23 | 0,994 | 0,105 | 0,662 | 102 | 16 | -2,662 | 1,15E-06 | 1,27E-05 |
| XECs426 | 294 | 31 | -3,239 | 8,02E-10 | 1,86E-07 | 643 | 4 | -7,470 | 1,02E-27 | 4,34E-25 |
| XECs429 | 35 | 30 | -0,224 | 0,699 | 1,000 | 47 | 8 | -2,611 | 1,66E-05 | 1,50E-04 |
| XECs430 | 14 | 9 | -0,710 | 0,306 | 1,000 | 18 | 1 | -4,009 | 1,00E-05 | 9,42E-05 |
| XECs431 | 9 | 11 | 0,287 | 0,739 | 1,000 | 19 | 4 | -2,212 | 0,002 | 0,011 |
| XECs432 | 4 | 2 | -1,156 | 0,398 | 1,000 | 11 | 0 | -6,475 | 2,21E-05 | 1,94E-04 |
| XECs433 | 63 | 5 | -3,621 | 1,20E-08 | 2,00E-06 | 44 | 2 | -4,762 | 8,47E-10 | 1,59E-08 |
| XECs434 | 54 | 12 | -2,205 | 1,16E-04 | 0,005 | 22 | 3 | -3,041 | 6,07E-05 | 4,89E-04 |
| XECs435 | 474 | 150 | -1,662 | 0,001 | 0,017 | 1085 | 74 | -3,880 | 1,47E-13 | 5,16E-12 |
| XECs436 | 70 | 50 | -0,497 | 0,327 | 1,000 | 172 | 18 | -3,282 | 1,94E-09 | 3,41E-08 |
| XECs437 | 20 | 10 | -0,954 | 0,133 | 0,779 | 49 | 3 | -3,958 | 1,06E-08 | 1,67E-07 |
| XECs438 | 19 | 10 | -0,952 | 0,139 | 0,779 | 47 | 3 | -3,913 | 1,63E-08 | 2,47E-07 |
| XECs439 | 49 | 67 | 0,467 | 0,363 | 1,000 | 87 | 4 | -4,391 | 1,66E-11 | 4,18E-10 |
| XECs440 | 68 | 40 | -0,752 | 0,142 | 0,779 | 173 | 36 | -2,255 | 1,04E-05 | 9,69E-05 |
| XECs441 | 38 | 13 | -1,538 | 0,007 | 0,128 | 256 | 16 | -4,034 | 6,43E-13 | 1,98E-11 |
| XECs442 | 49 | 120 | 1,291 | 0,010 | 0,156 | 131 | 34 | -1,962 | 1,27E-04 | 9,55E-04 |
| XECs443 | 4 | 1 | -1,873 | 0,152 | 0,806 | 16 | 2 | -2,877 | 4,62E-04 | 0,003 |
| XECs444 | 275 | 206 | -0,416 | 0,378 | 1,000 | 1054 | 210 | -2,329 | 1,75E-06 | 1,87E-05 |
| XECs445 | 5 | 27 | 2,553 | 1,57E-04 | 0,006 | 8 | 1 | -3,608 | 0,003 | 0,015 |
| XECs446 | 24 | 17 | -0,463 | 0,445 | 1,000 | 60 | 4 | -3,852 | 4,60E-09 | 7,62E-08 |
| XECs447 | 123 | 136 | 0,141 | 0,779 | 1,000 | 67 | 22 | -1,622 | 0,002 | 0,013 |
| XECs448 | 13 | 4 | -1,613 | 0,034 | 0,347 | 32 | 0 | -8,005 | 1,34E-10 | 2,88E-09 |
| XECs449 | 633 | 373 | -0,762 | 0,103 | 0,650 | 3441 | 65 | -5,722 | 9,81E-24 | 1,85E-21 |
| XECs450 | 50 | 32 | -0,649 | 0,220 | 1,000 | 189 | 9 | -4,368 | 1,38E-13 | 4,88E-12 |
| XECs451 | 43 | 9 | -2,321 | 1,06E-04 | 0,005 | 337 | 6 | -5,902 | 1,82E-20 | 2,00E-18 |
| XECs452 | 2 | 2 | 0,001 | 1,000 | 1,000 | 11 | 3 | -2,082 | 0,013 | 0,059 |
| XECs453 | 17 | 21 | 0,312 | 0,632 | 1,000 | 13 | 2 | -3,013 | 0,001 | 0,004 |
| XECs454 | 16 | 8 | -0,988 | 0,141 | 0,779 | 20 | 3 | -2,937 | 1,22E-04 | 0,001 |
| XECs455 | 275 | 206 | -0,416 | 0,378 | 1,000 | 1054 | 210 | -2,329 | 1,75E-06 | 1,87E-05 |
| XECs456 | 13 | 9 | -0,548 | 0,452 | 1,000 | 16 | 2 | -3,264 | 1,58E-04 | 0,001 |
| XECs457 | 19 | 3 | -2,612 | 4,65E-04 | 0,015 | 80 | 3 | -4,922 | 1,96E-12 | 5,49E-11 |
| XECs458 | 3 | 8 | 1,288 | 0,154 | 0,807 | 35 | 8 | -2,111 | 0,001 | 0,004 |
| XECs459 | 4 | 8 | 1,165 | 0,181 | 0,901 | 53 | 11 | -2,254 | 8,86E-05 | 0,001 |
| XECs460 | 2 | 2 | 0,001 | 1,000 | 1,000 | 20 | 2 | -3,206 | 5,72E-05 | 4,65E-04 |
| XECs461 | 6 | 1 | -2,321 | 0,046 | 0,408 | 64 | 1 | -6,680 | 6,26E-14 | 2,33E-12 |
| XECs462 | 8 | 2 | -2,321 | 0,018 | 0,229 | 86 | 1 | -6,257 | 4,32E-15 | 1,93E-13 |
| XECs463 | 3 | 1 | -1,221 | 0,490 | 1,000 | 18 | 1 | -3,968 | 1,33E-05 | 1,22E-04 |
| XECs464 | 9 | 15 | 0,763 | 0,268 | 1,000 | 27 | 6 | -2,242 | 0,001 | 0,004 |
| XECs465 | 271 | 69 | -1,968 | 6,71E-05 | 0,003 | 349 | 11 | -4,970 | 3,17E-17 | 1,93E-15 |
| ECs0239 | 27 | 7 | -2,007 | 0,002 | 0,017 | 16 | 2 | -3,265 | 1,58E-04 | 0,001 |
| ECs0275 | 8 | 2 | -2,322 | 0,018 | 0,100 | 14 | 2 | -3,067 | 0,001 | 0,003 |
| ECs0326 | 15 | 17 | 0,179 | 0,817 | 0,983 | 12 | 3 | -1,895 | 0,019 | 0,066 |
| ECs0439 | 52 | 157 | 1,601 | 0,001 | 0,014 | 37 | 54 | 0,564 | 0,282 | 0,472 |
| ECs0513 | 171 | 88 | -0,953 | 0,049 | 0,201 | 131 | 52 | -1,325 | 0,008 | 0,032 |
| ECs0519 | 9 | 3 | -1,546 | 0,070 | 0,253 | 26 | 10 | -1,412 | 0,021 | 0,072 |
| ECs0662 | 438 | 1618 | 1,886 | 7,77E-05 | 0,001 | 889 | 1847 | 1,056 | 0,023 | 0,077 |
| ECs0790 | 73 | 131 | 0,842 | 0,085 | 0,286 | 195 | 411 | 1,076 | 0,023 | 0,076 |
| ECs0966 | 28 | 12 | -1,214 | 0,041 | 0,180 | 33 | 9 | -1,941 | 0,001 | 0,008 |
| ECs1037 | 24 | 203 | 3,074 | 7,94E-09 | 4,49E-07 | 9 | 65 | 2,825 | 1,49E-06 | 2,15E-05 |
| ECs1058 | 12 | 4 | -1,556 | 0,043 | 0,184 | 27 | 6 | -2,269 | 0,001 | 0,003 |
| ECs1059 | 13 | 5 | -1,301 | 0,079 | 0,273 | 17 | 2 | -3,010 | 2,06E-04 | 0,002 |
| ECs1061 | 9 | 3 | -1,716 | 0,054 | 0,212 | 19 | 3 | -2,865 | 1,98E-04 | 0,002 |
| ECs1098 | 9 | 2 | -2,408 | 0,013 | 0,078 | 3 | 4 | 0,214 | 1,000 | 1,000 |
| ECs1144 | 30 | 7 | -2,056 | 0,001 | 0,012 | 9 | 5 | -1,057 | 0,190 | 0,356 |
| ECs1145 | 480 | 240 | -1,000 | 0,034 | 0,159 | 1149 | 232 | -2,310 | 2,05E-06 | 2,85E-05 |
| ECs1159 | 9 | 26 | 1,571 | 0,012 | 0,073 | 22 | 56 | 1,363 | 0,011 | 0,043 |
| ECs1178 | 42 | 18 | -1,200 | 0,030 | 0,147 | 13 | 2 | -3,013 | 0,001 | 0,004 |
| ECs1186 | 54 | 12 | -2,158 | 1,50E-04 | 0,002 | 87 | 10 | -3,096 | 8,16E-08 | 1,50E-06 |
| ECs1188 | 24 | 6 | -2,070 | 0,002 | 0,018 | 8 | 2 | -2,322 | 0,018 | 0,063 |
| ECs1193 | 10 | 1 | -3,097 | 0,002 | 0,021 | 12 | 1 | -4,217 | 1,24E-04 | 0,001 |
| ECs1436 | 1 | 2 | 0,530 | 1,000 | 1,000 | 1 | 5 | 3,036 | 0,024 | 0,079 |
| ECs1538 | 7 | 1 | -3,406 | 0,006 | 0,047 | 4 | 2 | -1,157 | 0,398 | 0,593 |
| ECs1569 | 4 | 5 | 0,165 | 1,000 | 1,000 | 11 | 3 | -2,083 | 0,013 | 0,049 |
| ECs1624 | 10 | 1 | -3,097 | 0,002 | 0,021 | 5 | 2 | -1,509 | 0,203 | 0,372 |
| ECs1627 | 57 | 3 | -4,444 | 3,26E-10 | 2,97E-08 | 61 | 1 | -6,600 | 1,54E-13 | 6,34E-12 |
| ECs1655 | 3 | 11 | 1,832 | 0,025 | 0,126 | 2 | 16 | 2,924 | 3,52E-04 | 0,002 |
| ECs1856 | 75 | 472 | 2,650 | 1,26E-07 | 4,99E-06 | 443 | 2636 | 2,576 | 1,37E-07 | 2,40E-06 |
| ECs1967 | 10 | 2 | -2,566 | 0,006 | 0,047 | 5 | 1 | -3,035 | 0,024 | 0,079 |
| ECs2040 | 11 | 9 | -0,367 | 0,650 | 0,897 | 21 | 1 | -5,079 | 3,30E-07 | 5,32E-06 |
| ECs2049 | 13 | 27 | 1,047 | 0,077 | 0,269 | 6 | 35 | 2,499 | 8,37E-05 | 0,001 |
| ECs2084 | 15 | 168 | 3,523 | 2,68E-10 | 2,48E-08 | 33 | 373 | 3,514 | 3,52E-11 | 1,05E-09 |
| ECs2085 | 4 | 71 | 4,098 | 3,85E-10 | 3,45E-08 | 7 | 97 | 3,867 | 2,30E-10 | 6,09E-09 |
| ECs2139 | 66 | 20 | -1,753 | 0,001 | 0,012 | 36 | 20 | -0,880 | 0,114 | 0,250 |
| ECs2181 | 30 | 4 | -2,869 | 2,56E-05 | 0,001 | 13 | 3 | -2,265 | 0,006 | 0,025 |
| ECs2192 | 16 | 4 | -1,921 | 0,009 | 0,059 | 4 | 1 | -2,722 | 0,061 | 0,158 |
| ECs2212 | 7 | 4 | -0,789 | 0,393 | 0,699 | 16 | 2 | -3,310 | 1,19E-04 | 0,001 |
| ECs2255 | 8 | 2 | -2,322 | 0,018 | 0,100 | 7 | 1 | -3,406 | 0,006 | 0,028 |
| ECs2271 | 23 | 10 | -1,233 | 0,048 | 0,198 | 84 | 3 | -4,750 | 2,88E-12 | 1,04E-10 |
| ECs2280 | 275 | 77 | -1,842 | 1,76E-04 | 0,003 | 849 | 37 | -4,515 | 1,63E-16 | 1,02E-14 |
| ECs2333 | 177 | 62 | -1,523 | 0,002 | 0,019 | 105 | 27 | -1,981 | 1,46E-04 | 0,001 |
| ECs2334 | 207 | 66 | -1,658 | 0,001 | 0,009 | 138 | 46 | -1,592 | 0,001 | 0,008 |
| ECs2382 | 31 | 20 | -0,665 | 0,242 | 0,539 | 25 | 6 | -2,036 | 0,002 | 0,009 |
| ECs2533 | 232 | 223 | -0,054 | 0,914 | 1,000 | 305 | 144 | -1,084 | 0,023 | 0,075 |
| ECs2534 | 111 | 144 | 0,370 | 0,445 | 0,745 | 46 | 240 | 2,378 | 2,68E-06 | 3,61E-05 |
| ECs2536 | 152 | 90 | -0,751 | 0,121 | 0,360 | 356 | 28 | -3,688 | 6,79E-12 | 2,31E-10 |
| ECs2653 | 104 | 44 | -1,248 | 0,013 | 0,079 | 224 | 44 | -2,361 | 3,30E-06 | 4,27E-05 |
| ECs2695 | 14 | 16 | 0,243 | 0,742 | 0,949 | 51 | 13 | -2,003 | 4,20E-04 | 0,003 |
| ECs2748 | 40 | 13 | -1,612 | 0,005 | 0,038 | 15 | 7 | -1,142 | 0,102 | 0,229 |
| ECs2755 | 25 | 23 | -0,120 | 0,869 | 1,000 | 43 | 8 | -2,483 | 4,56E-05 | 4,31E-04 |
| ECs2765 | 5 | 4 | -0,500 | 0,687 | 0,922 | 26 | 9 | -1,517 | 0,014 | 0,051 |
| ECs2971 | 13 | 3 | -2,266 | 0,006 | 0,043 | 8 | 3 | -1,630 | 0,071 | 0,176 |
| ECs2989 | 110 | 68 | -0,697 | 0,157 | 0,418 | 112 | 36 | -1,634 | 0,001 | 0,007 |
| ECs2997 | 35 | 18 | -0,955 | 0,089 | 0,296 | 15 | 2 | -2,782 | 0,001 | 0,005 |
| ECs3003 | 12 | 15 | 0,331 | 0,649 | 0,896 | 41 | 14 | -1,541 | 0,007 | 0,028 |
| ECs3004 | 19 | 19 | 0,000 | 1,000 | 1,000 | 44 | 17 | -1,408 | 0,011 | 0,043 |
| ECs3079 | 28 | 36 | 0,367 | 0,513 | 0,802 | 24 | 65 | 1,433 | 0,007 | 0,029 |
| ECs3239 | 94 | 39 | -1,285 | 0,011 | 0,072 | 83 | 39 | -1,078 | 0,034 | 0,102 |
| ECs3366 | 32 | 17 | -0,885 | 0,121 | 0,360 | 30 | 5 | -2,679 | 6,39E-05 | 0,001 |
| ECs3390 | 98 | 478 | 2,290 | 3,36E-06 | 9,19E-05 | 49 | 170 | 1,788 | 3,49E-04 | 0,002 |
| ECs3553 | 459 | 390 | -0,237 | 0,613 | 0,878 | 615 | 258 | -1,254 | 0,008 | 0,033 |
| ECs3690 | 134 | 298 | 1,106 | 0,021 | 0,111 | 987 | 315 | -1,649 | 0,001 | 0,003 |
| ECs3815 | 38 | 22 | -0,799 | 0,148 | 0,407 | 63 | 18 | -1,840 | 0,001 | 0,004 |
| ECs3891 | 29 | 286 | 3,321 | 3,91E-10 | 3,45E-08 | 5 | 14 | 1,559 | 0,035 | 0,104 |
| ECs4140 | 19 | 69 | 1,843 | 0,001 | 0,008 | 30 | 61 | 1,033 | 0,048 | 0,133 |
| ECs4199 | 128 | 106 | -0,266 | 0,587 | 0,860 | 469 | 137 | -1,774 | 2,36E-04 | 0,002 |
| ECs4415 | 61 | 72 | 0,240 | 0,641 | 0,895 | 29 | 142 | 2,287 | 1,06E-05 | 1,18E-04 |
| ECs4441 | 4909 | 3076 | -0,674 | 0,144 | 0,398 | 16280 | 3243 | -2,327 | 1,38E-06 | 2,01E-05 |
| ECs4511 | 1216 | 1121 | -0,117 | 0,800 | 0,977 | 1621 | 696 | -1,220 | 0,009 | 0,037 |
| ECs4587 | 26 | 5 | -2,498 | 2,26E-04 | 0,003 | 15 | 1 | -4,597 | 1,14E-05 | 1,27E-04 |
| ECs4638 | 1752 | 880 | -0,993 | 0,033 | 0,155 | 10120 | 3516 | -1,525 | 0,001 | 0,007 |
| ECs4644 | 22 | 3 | -3,075 | 4,83E-05 | 0,001 | 4 | 3 | -0,400 | 0,820 | 0,938 |
| ECs4659 | 9 | 38 | 2,063 | 0,001 | 0,007 | 3 | 9 | 1,465 | 0,091 | 0,213 |
| ECs4701 | 195 | 70 | -1,476 | 0,003 | 0,023 | 58 | 41 | -0,499 | 0,337 | 0,529 |
| ECs4958 | 10 | 3 | -1,875 | 0,030 | 0,147 | 7 | 1 | -2,663 | 0,014 | 0,053 |
| ECs4997 | 51 | 39 | -0,372 | 0,484 | 0,780 | 292 | 32 | -3,205 | 1,14E-09 | 2,78E-08 |
| ECs5028 | 24 | 30 | 0,326 | 0,578 | 0,854 | 28 | 11 | -1,378 | 0,022 | 0,074 |
| ECs5382 | 8 | 27 | 1,712 | 0,006 | 0,045 | 22 | 30 | 0,454 | 0,432 | 0,626 |
| ECs5411 | 5 | 5 | 0,148 | 1,000 | 1,000 | 24 | 1 | -5,240 | 8,89E-08 | 1,62E-06 |
| ECs5420 | 22 | 42 | 0,962 | 0,078 | 0,271 | 31 | 12 | -1,360 | 0,021 | 0,070 |
| ECs5433 | 2 | 1 | -0,918 | 0,709 | 0,930 | 1 | 11 | 3,306 | 0,001 | 0,005 |
| ECs5442 | 10 | 3 | -1,948 | 0,023 | 0,119 | 6 | 1 | -3,292 | 0,010 | 0,039 |
| ECs5463 | 11 | 21 | 0,991 | 0,113 | 0,344 | 14 | 36 | 1,335 | 0,020 | 0,068 |
| ECs5465 | 33 | 11 | -1,574 | 0,008 | 0,053 | 11 | 6 | -0,917 | 0,234 | 0,416 |
| ECs5495 | 11 | 12 | 0,124 | 0,920 | 1,000 | 10 | 36 | 1,888 | 0,002 | 0,008 |
| ECs5496 | 123 | 93 | -0,405 | 0,407 | 0,707 | 260 | 80 | -1,696 | 0,001 | 0,003 |
| ECs5530 | 315 | 138 | -1,190 | 0,013 | 0,078 | 526 | 33 | -4,011 | 1,05E-13 | 4,54E-12 |
| ECs5531 | 24 | 197 | 3,027 | 1,29E-08 | 6,84E-07 | 11 | 352 | 5,051 | 1,50E-17 | 1,08E-15 |
| ECs5575 | 2 | 2 | -0,387 | 1,000 | 1,000 | 5 | 30 | 2,704 | 5,36E-05 | 4,93E-04 |
| ECs5586 | 19 | 10 | -0,952 | 0,139 | 0,393 | 21 | 5 | -2,008 | 0,003 | 0,016 |

*Mean counts of two biological replicates normalized to the smallest library are shown.
